# CD44a functions as a regulator of p53 signaling, apoptosis and autophagy in the antibacterial immune response

**DOI:** 10.1038/s42003-022-03856-1

**Published:** 2022-08-30

**Authors:** Lu Cao, Hong Fang, Dong Yan, Xiao Man Wu, Jie Zhang, Ming Xian Chang

**Affiliations:** 1 State Key Laboratory of Freshwater Ecology and Biotechnology, Institute of Hydrobiology, Chinese Academy of Sciences, Wuhan, 430072 China; 2 College of Advanced Agricultural Sciences, University of Chinese Academy of Sciences, 100049 Beijing, China; 3 Innovation Academy for Seed Design, Chinese Academy of Sciences, Wuhan, 430072 China

**Keywords:** Immunology, Innate immunity

## Abstract

The cell adhesion molecule CD44 has been implicated in diverse biological functions including the pathological responses to infections and inflammatory diseases. The variable forms of CD44 contribute to functional variations, which are not yet defined in teleost. Here, we show that zebrafish CD44a plays a protective role in the host defense against *Edwardsiella piscicida* infection. Zebrafish CD44a deficiency inhibits cell growth and proliferation, impairs cell growth and death pathways, and regulates the expression levels of many genes involved in p53 signaling, apoptosis and autophagy. In addition, CD44a gene disruption in zebrafish leads to inhibition of apoptosis and induction of autophagy, with the increased susceptibility to *E. piscicida* infection. Furthermore, we show that zebrafish CD44a variants including CD44a_tv1 and CD44a_tv2 promote the translocation of p53 from the nucleus to the cytoplasm and interact with p53 in the cytoplasm. Mechanistically, zebrafish CD44a_tv1 mediates the beneficial effect for larvae survival infected with *E. piscicida* is depending on the CASP8-mediated apoptosis. However, the antibacterial effect of zebrafish CD44a_tv2 depends on the cytoplasmic p53-mediated inhibition of autophagy. Collectively, our results identify that different mechanisms regulate CD44a variants-mediated antibacterial responses.

## Abstract (web summary)

The adhesion molecule CD44 drives host defence in zebrafish by regulating apoptosis and autophagy.

## Introduction

CD44 proteins are well characterized members of cell adhesion molecules, which have large extracellular domains, a membrane spanning region, and a cytoplasmic functional domain^1^. Among them, the N-terminal extracellular domains can bind various ligands, which include hyaluronic acid, osteopontin, collagen, laminin, proteoglycan, matrix metalloproteinases, as well as growth factors and cytokines^2–4^. The cytoplasmic domain of CD44 proteins is responsible for signal transduction through binding to different molecules, including cytoskeleton elements, Src family kinases such as Src, Lck, Fyn and Lyn, Rho-family GTPases and associated molecules^5,6^. The function of CD44 has been implicated in numerous biological processes. As a lymphocyte homing receptor, CD44 is involved in lymphocyte activation, recirculation and homing^7,8^. As a cell adhesion molecule, the major physiological role of CD44 is to maintain organ and tissue structure via cell-cell and cell-matrix interaction^1^. Furthermore, CD44 also plays a critical role in leukocyte recruitment, T-cell activation, tumor progression and metastasis, as well as in transmission of signals mediating hematopoiesis and apoptosis^8–10^.

CD44 glycoproteins are encoded by a single gene, which contains at least 19 exons and 10 alternatively spliced exons within the extracellular domain^11^. Mammalian CD44 exists in a variety of isoforms generated by alternative splicing of the pre-mRNA. In theory, more than 1000 different CD44 variants (CD44v) may be generated by alternative splicing^1^. At least 20 different isoforms of human CD44 have been described^12^. The standard form of CD44 (CD44s) is encoded by the ten constant exons (exons 1–5 and 16–20), CD44v are generated within the standard exons at an insertion site between exons 5 and 16^13,14^. The functions of multiple CD44v in tumor initiation and metastasis were established^15,16^. In normal cells, CD44v is reported to participate in cell proliferation, activation, apoptosis, differentiation, migration and adhesion^17^. For example during T-cell development and activation, CD44 splicing variant 6 (CD44v6) plays a pivotal role, whose expression provides a proliferative stimulus for T cells^18^.

In teleost, two CD44 genes CD44a and CD44b exist in zebrafish database. Bioinformatic analysis show that zebrafish CD44a is not found to undergo alternative splicing, but zebrafish CD44b does. Five CD44b antigen isoforms, namely CD44b antigen isoform X1-isoform X5, are found in zebrafish database. Furthermore, a novel CD44 gene (named as CD44c), which was more similar to CD44b antigen isoforms rather than CD44a, was cloned and characterized. The negative regulation of zebrafish CD44c in viral and bacterial infection was revealed, which showed that the overexpression of zebrafish CD44c facilitated pathogenic proliferation with the impaired production of inflammatory cytokines and MHC genes in the case of SVCV infection, and a decreased production of antibacterial molecules in the case of *Edwardsiella piscicida* infection^19^. Although previous study has shown that NOD1 impacts larvae survival via CD44a^20^, the functions of piscine CD44a and the correlation between NOD1 and CD44a receptors in pathogen infection are unreported.

In the present study, two variants of zebrafish CD44a were identified. The antibacterial effects of CD44a variants were examined in CD44a variants-overexpressing zebrafish, CD44a-knockout and NOD1-knockout zebrafish, which was accompanied by studying the expression regulation of CD44a on many genes involved in NOD-like receptor (NLR) signaling pathway. After confirming that CD44a receptor has a functional linkage with NOD1 in antibacterial immune response, we next determine whether zebrafish CD44a is involved in the other biological process. Our data demonstrate that zebrafish CD44a also functions as a regulator of p53 signaling, apoptosis and autophagy in the antibacterial immune response. Finally, the present study reveals similar and different molecular mechanisms of CD44a variants in the defense against *E. piscicida* infection.

## Results

### Identification of zebrafish CD44a variants

The CD44a variants were screened by using primers that amplify the whole open reading frame (ORF). The amplicons were cloned and sequenced to identify potential transcripts or variants. This approach revealed the existence of two CD44a transcript variants, which may be generated not by alternative splicing but by gene replication. The longer CD44a transcript variant was named as CD44a_tv1 (GenBank accession number: MW674927), and encoded 392 amino acids. The shorter CD44a transcript variant was named as CD44a_tv2 (GenBank accession number: MW674928), and encoded 389 amino acids. The CD44a_tv1 corresponds to the CD44a previously reported. In addition to the length difference of 3 amino acids, there are also obvious differences in the composition of 34 amino acids between CD44a_tv1 and CD44a_tv2 (Supplementary Fig. 1a).

Mammalian CD44 is a transmembrane and highly glycosylated protein with several isoforms, which result from gene alternative splicing^6,14^. Analysis of zebrafish CD44a_tv1 and CD44a_tv2 transcript variants revealed the existences of 1 N-glycosylated site for CD44a_tv1 and 2 N-glycosylated sites for CD44a_tv2. Furthermore, two strong transmembrane helices were identified both for CD44a_tv1 and CD44a_tv2 (Supplementary Fig. 1a).

To further confirm the identities of zebrafish CD44a transcript variants, a phylogenetic tree was constructed using the neighbor-joining (N-J) method based on the alignments of vertebrate CD44 or CD44-like proteins. Zebrafish CD44a transcript variants and common carp CD44-like protein formed a branch, which is away from the big branch formed by zebrafish CD44b isoforms and CD44 proteins from amphibian, avian and mammalian animals (Supplementary Fig. 1b).

### Zebrafish CD44a variants function as positive regulators in antibacterial response

**Fig. 1:**
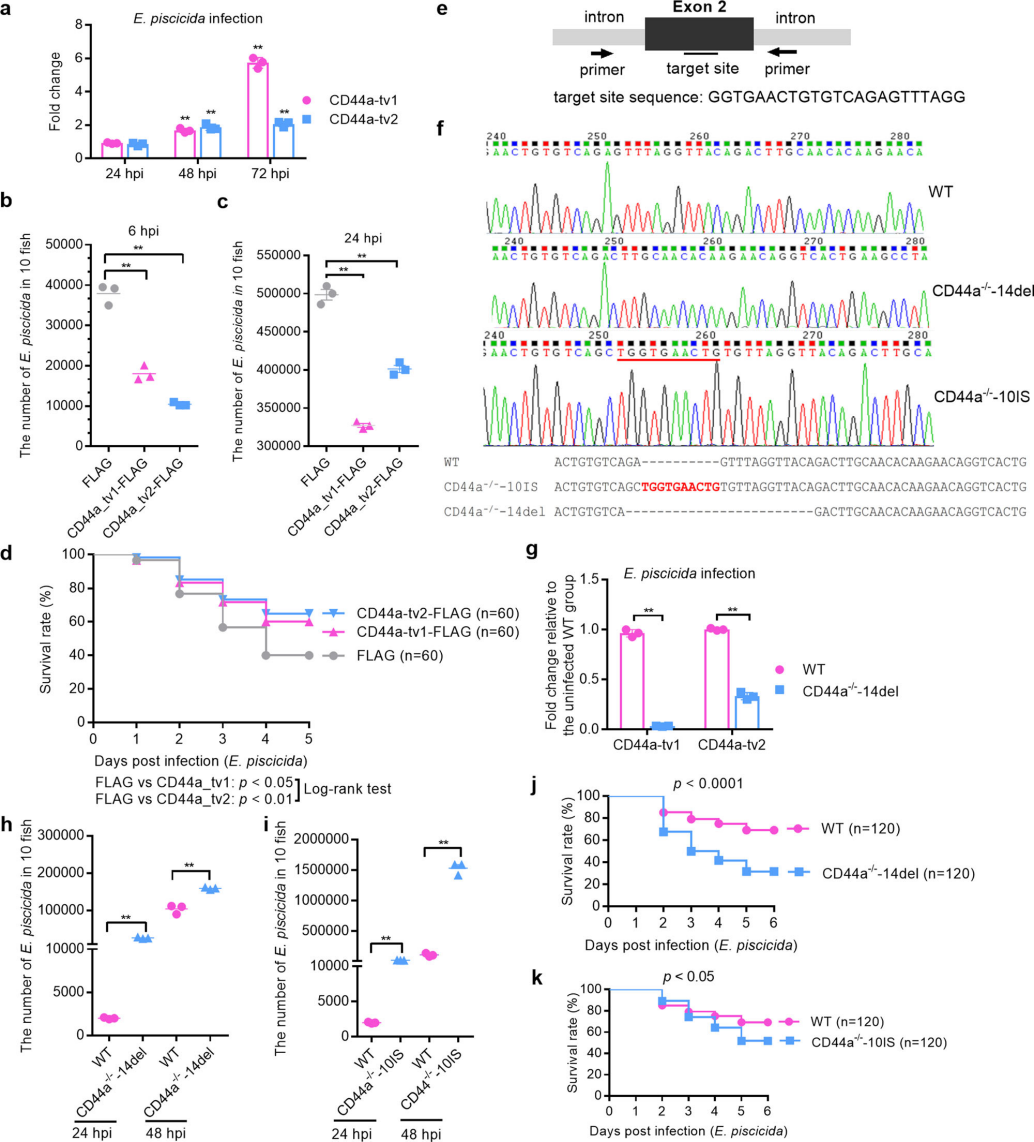
Zebrafish CD44a plays a critical role in protecting against *E. piscicida* infection. **a** The effect of *E. piscicida* infection on the expression of CD44a variants in zebrafish larvae. **b**, **c** The antibacterial effects of zebrafish CD44a variants at 6 and 24 hpi. **d** Larval survival analysis in the WT zebrafish microinfected with FLAG, CD44a_tv1-FLAG or CD44a_tv2-FLAG (*n* = 60 for each group). **e** Cartoon showing the position of the target site and its sequence in the zebrafish CD44a locus. **f** Representative Sanger sequencing results of the PCR amplicons from homozygous mutations with 14 bp deletions (CD44a^−/−^-14del), 10 bp insertions (CD44a^−/−^-10IS) or WT. **g** The expression of CD44a variants in the WT and CD44a^−/−^-14del zebrafish larvae. **h** Zebrafish larvae from the WT and CD44a^−/−^-14del mutants were collected at the indicated post-infection time points, and homogenates were made for CFU counts. **i** Zebrafish larvae from the WT and CD44a^−/−^-10IS mutants were collected at the indicated post-infection time points, and homogenates were made for CFU counts. **j** Larval survival analysis in the WT and CD44a^−/−^-14del zebrafish larvae infected with *E. piscicida* (*n* = 120 for each group). **k** Larval survival analysis in the WT and CD44a^−/−^-10IS zebrafish larvae infected with *E. piscicida* (*n* = 120 for each group). For **b**, **c**, **h** and **i**, each symbol represents the average counts of ten larvae. For **a**–**c** and **g**–**i**, the data are presented as means ± SD (*n* = 3). **p < 0.01.

To clarify the expression pattern of CD44a variants in response to *E. piscicida* infection, the mRNA expression levels of CD44a variants were examined in zebrafish larvae infected with *E. piscicida* for 24, 48 and 72 h. *E. piscicida* infection significantly induced the expressions of zebrafish CD44a_tv1 and CD44a_tv2 both at 48 hpi and 72 hpi, but not at 24 hpi. The expression of zebrafish CD44a_tv1 was increased continuously from 24 to 72 hpi, whereas slightly increased for zebrafish CD44a_tv2 (Fig. 1a). To determine the possible effect of CD44a variants in bacterial infection, zebrafish larvae microinjected with the empty plasmid, CD44a_tv1-FLAG and CD44a_tv2-FLAG were infected separately with *E. piscicida* by static immersion. The overexpression of zebrafish CD44a_tv1 or CD44a_tv2 significantly inhibited the proliferation of *E. piscicida* in vivo at 6 and 24 hpi (Fig. 1b, c), and increased the larval survival infected with *E. piscicida* (Fig. 1d).

To investigate the effect of CD44a knockout in bacterial infection, two homozygotic CD44a^−/−^ mutants, including insertion of 10 base-pairs (CD44a^−/−^-10IS) and deletion of 14 bp (CD44a^−/−^-14del), were screened and produced by Cas9/gRNA system (Fig. 1e, f). The impacts of CD44a knockout on the two transcripts were examined by qRT-PCR. CD44a deficiency significantly impaired the expressions of CD44a variants in zebrafish larvae, especially for zebrafish CD44a_tv1 (Fig. 1g). To confirm the impact of CD44a knockout on the derived proteins, the protein lysates from the WT and CD44a^−/−^-14del zebrafish collected at 5 dpf were examined by Western blotting using the anti-CD44a monoclonal antibody. In the WT zebrafish larvae, the protein expression of CD44a_tv1 was stronger than CD44a_tv2. The bands corresponding to the WT CD44a variants were not detected in the homozygous CD44a^−/−^-14del zebrafish larvae (Supplementary Fig. 2a). Compared with WT zebrafish, CD44a^−/−^-14del and CD44a^−/−^-10IS mutants demonstrated the higher bacterial load (Fig. 1h, i) and the decreased survival rates (Fig. 1j, k). Therefore, CD44a_tv1 or CD44a_tv2 are effective in protecting against *E. piscicida* infection.

### The antibacterial effect of zebrafish CD44a is not dependent on antibacterial pattern recognition receptor NOD1

**Fig. 2:**
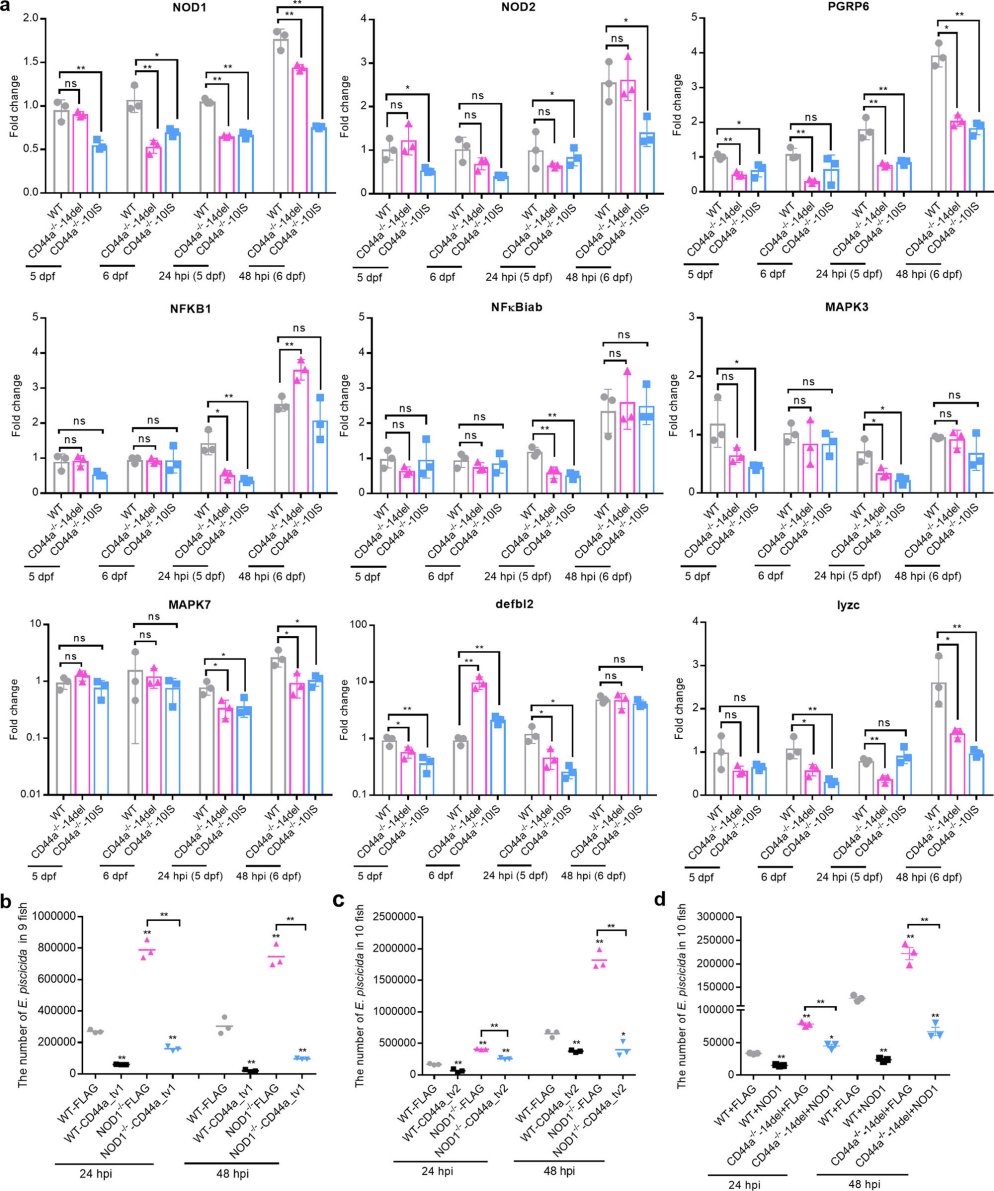
The antibacterial effects of zebrafish CD44a and NOD1 are independent of each other. **a** The quantification analysis of mRNA levels of key genes involved in the NOD1-RIPK2 signaling pathway in the WT, CD44a^−/−^-14del and CD44a^−/−^-10IS zebrafish larvae, as determined by qRT-PCR. Quantification data represent the expression level of genes compared with those in the WT zebrafish. **b** Zebrafish larvae from the WT and NOD1^−/−^ mutants microinjected with p3×FLAG or CD44a_tv1-FLAG were collected at the indicated post-infection time points, and homogenates were made for CFU counts. **c** Zebrafish larvae from the WT and NOD1^−/−^ mutants microinjected with p3×FLAG or CD44a_tv2-FLAG were collected at the indicated post-infection time points, and homogenates were made for CFU counts. **d** Zebrafish larvae from the WT and CD44a^−/−^-14del mutants microinjected with p3×FLAG or NOD1-FLAG were collected at the indicated post-infection time points, and homogenates were made for CFU counts. For **a**–**d**, the data are presented as means ± SD (*n* = 3). *p < 0.05, **p < 0.01; ns not significant.

NOD1 is the best-characterized antibacterial pattern recognition receptor (PRR). Our previous studies have revealed that both NOD1 and its adapter protein RIPK2 influence the expression of CD44a enormously, but not for CD44c^19–21^. Furthermore, NOD1 plays a protective role in larval survival through CD44a-mediated PI3K-Akt signaling^20^. We firstly investigate whether the antibacterial effect of CD44a is associated with the NOD1-RIPK2 signaling pathway. In two different homozygous mutants, we found that CD44a deficiency significantly inhibited the expressions of key genes involved in the NOD1-RIPK2 signaling pathway (Fig. 2a). Since the expression trends of most genes were consistent both in CD44a^−/−^-10IS and CD44a^−/−^-14del, only CD44a^−/−^-14del was chosen for the follow-up research. Next, we examined the PI3K-Akt signaling cascade from the WT and CD44a^−/−^-14del zebrafish by Western blotting. In the absence of infection and in the case of *E. piscicida* infection, CD44a deficiency decreased the levels of phosphorylated and total proteins of Akt and GSK3β (Supplementary Fig. 2b, c).

In order to confirm our speculation, rescue experiments were performed in CD44a^−/−^-14del or NOD1^−/−^ mutants. When zebrafish CD44a transcript variants were overexpressed in NOD1 mutants, NOD1 deficiency failed to abolish the antibacterial effect of CD44a_tv1 (Fig. 2b) and CD44a_tv2 (Fig. 2c). When NOD1 was overexpressed in CD44a^−/−^-14del mutants, CD44a deficiency failed to abolish the antibacterial effect of NOD1 (Fig. 2d).

Collectively, these results suggest that the antibacterial activity mediated by zebrafish CD44a and NOD1 was not dependent on each other, although there was mutual regulation existed between zebrafish CD44a and NOD1.

### CD44a deficiency impairs cell growth and death pathways

**Fig. 3:**
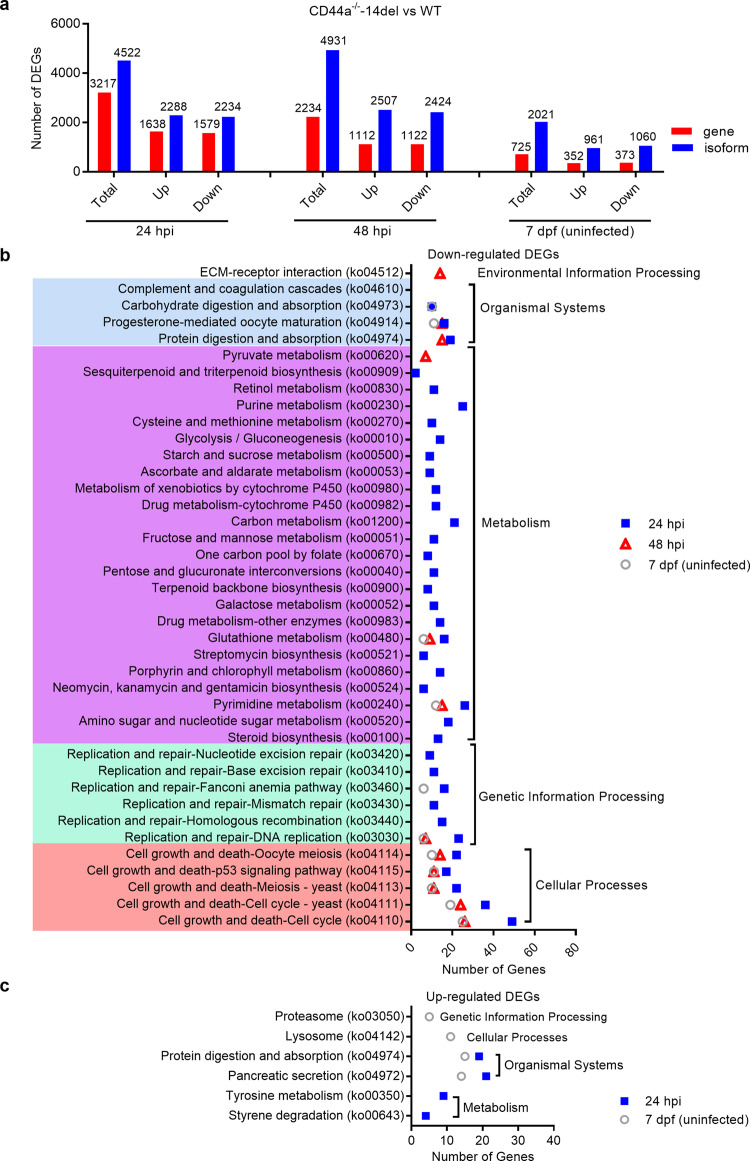
CD44a deficiency impairs cell growth and death pathways. **a** The numbers of differentially expressed genes and isoforms at 24 hpi, 48 hpi and 7 dpf from the WT and CD44a^−/−^-14del zebrafish larvae with or without the infection of *E. piscicida*. **b** KEGG enrichment analysis of the down-regulated DEGs at 24 hpi, 48 hpi and 7 dpf from the WT and CD44a^−/−^-14del zebrafish larvae with or without the infection of *E. piscicida*. **c** KEGG enrichment analysis of the up-regulated DEGs at 24 hpi, 48 hpi and 7 dpf from the WT and CD44a^−/−^-14del zebrafish larvae with or without the infection of *E. piscicida*.

In order to reveal the possible antibacterial mechanism of CD44a, zebrafish larvae infected with *E. piscicida* collected at 24 and 48 hpi were used for transcriptome sequencing, together with the uninfected larvae collected at 7 days post-fertilization (dpf). A total of 3217, 2234 and 725 genes were considered to be differentially expressed at 24 hpi, 48 hpi and 7 dpf, respectively. Among them, 1638 up-regulated and 1579 down-regulated genes were identified at 24 hpi, 1112 up-regulated and 1122 down-regulated genes identified at 48 hpi, 352 up-regulated and 373 down-regulated genes identified at 7 dpf (Fig. 3a). For down-regulated differentially expressed genes (DEGs), the significantly enriched signaling pathways are mainly involved in metabolism (24 pathways), and cell growth and death signaling pathways (5 pathways). Among cell growth and death signaling pathways, p53 and two cell cycle signaling pathways were enriched at 24 hpi, 48 hpi and 7 dpf (Fig. 3b). For up-regulated DEGs, only four pathways were significantly enriched at 24 hpi and 7 dpf respectively, and no significantly enriched signaling pathways at 48 hpi (Fig. 3c). Collectively, these results suggest that CD44a deficiency impairs cell growth and death pathways both in the absence of infection and in the case of *E. piscicida* infection.

### The antibacterial effects of zebrafish CD44a variants are dependent on the inhibition of autophagy

**Fig. 4:**
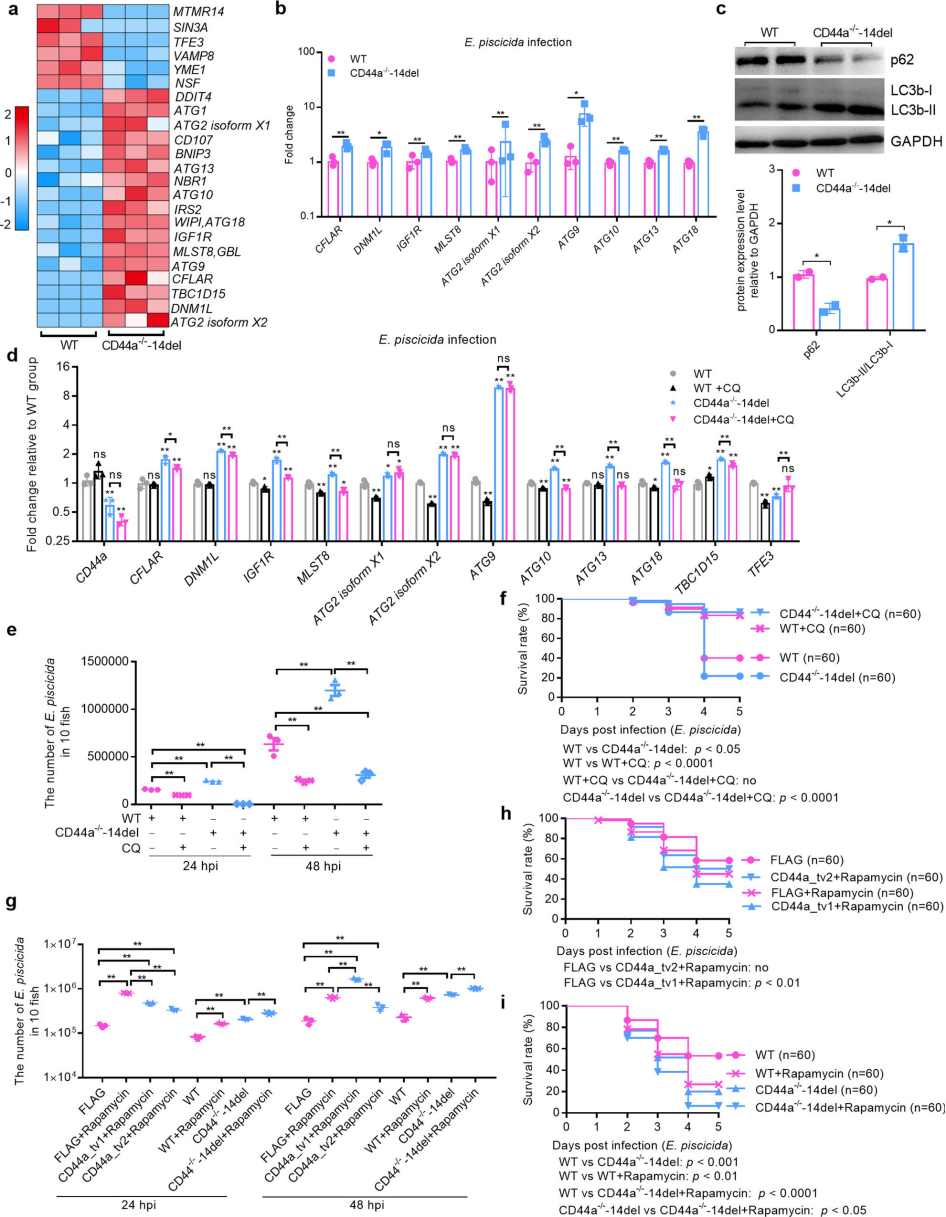
The antibacterial effects of zebrafish CD44a variants are dependent on the inhibition of autophagy. **a** DEGs involved in autophagy at 24 hpi by RNAseq analysis of mRNA expression levels in the WT and CD44a^−/−^-14del zebrafish larvae infected with *E. piscicida*. **b** Validation of DEGs involved in autophagy at 24 hpi by qRT-PCR. **c** Immunoblotting analysis and quantification of p62 and LC3b levels in the WT and CD44a^−/−^-14del zebrafish larvae infected with *E. piscicida* collected at 24 hpi. WB were repeated at least three times, and shown were the representative data. **d** mRNA levels of CD44a and autophagy-related genes in the WT and CD44a^−/−^-14del zebrafish larvae without or with the treatment of the autophagy inhibitor chloroquine (CQ). **e** Zebrafish larvae from the WT and CD44a^−/−^-14del mutants without or with the treatment of CQ were collected at the indicated post-infection time points, and homogenates were made for CFU counts. **f** Larval survival analysis in the WT and CD44a^−/−^-14del zebrafish larvae without or with the treatment of CQ (*n* = 60 for each group). **g** Zebrafish larvae microinjected with FLAG, CD44a_tv1 or CD44a_tv2 without or with the treatment of rapamycin or zebrafish larvae from the WT and CD44a^−/−^-14del mutants without or with the treatment of rapamycin were collected at the indicated post-infection time points, and homogenates were made for CFU counts. **h** Larval survival analysis for zebrafish larvae microinjected with FLAG, CD44a_tv1 or CD44a_tv2 without or with the treatment of rapamycin (*n* = 60 for each group). **i** Larval survival analysis for zebrafish larvae from the WT and CD44a^−/−^-14del mutants without or with the treatment of rapamycin (*n* = 60 for each group). Data are presented as mean values ± SD (*n* = 3) and *p* values by Student’s *t* test are shown in (**b**–**e**, **g**). *p < 0.05, **p < 0.01; ns not significant.

The results of transcriptome sequencing showed that zebrafish CD44a deficiency regulated the transcriptions of 23 autophagy-related genes (Fig. 4a). The results from qRT-PCR confirmed the induced expression of *CFLAR*, *DNM1L*, *IGF1R*, *MLST8*, *ATG2* isoform X1, *ATG2* isoform X2, *ATG9*, *ATG10*, *ATG13* and *ATG18*, which is 100% consistent with the results of transcriptome sequencing (Fig. 4a, b). Similar to CD44a deficiency, blocking p53 transactivation using pifithrin-α in zebrafish also induced the transcription of autophagy-related genes including *CFLAR*, *MLST8*, *ATG2* isoform X1, *ATG2* isoform X2, *ATG9*, *ATG10*, *ATG13* and *ATG18* (Supplementary Fig. 3).

To confirm the effect of zebrafish CD44a deficiency on autophagy, we performed LC3b and p62 Western blotting detection on larvae extracts in vivo. Compared with WT, zebrafish CD44a deficiency increased the LC3b-II/LC3b-I ratio (a marker of autophagosome formation) and decreased p62 levels, thereby enhancing the formation of autophagosomes (Fig. 4c).

Previous studies have shown that the transcription levels of autophagy genes can be altered in some chloroquine (CQ)-treated cells or animals^22,23^. Since zebrafish CD44a deficiency regulated the transcriptions of autophagy-related genes and induced autophagy, we would like to know whether the induced transcriptions of autophagy-related genes by CD44a deficiency were weakened by CQ, an autophagy inhibitor. In WT zebrafish, treatment with CQ inhibited the expression of autophagy-related genes including *IGF1R*, *MLST8*, *ATG2* isoform X1, *ATG2* isoform X2, *ATG9*, *ATG10*, *ATG18* and *TFE3* (Fig. 4d). The treatment with CQ significantly inhibited the induced expression of *CFLAR*, *DNM1L*, *IGF1R*, *MLST8*, *ATG10*, *ATG13*, *ATG18, TBC1D15*, and increased the impaired expression of *TFE3* regulated by CD44a deficiency (Fig. 4d). In WT zebrafish, the treatment with CQ significantly inhibited the bacterial proliferation and increased the survival rate of zebrafish larvae infected with *E. piscicida* (Fig. 4e, f). Zebrafish CD44a deficiency did not affect the survival of zebrafish larvae infected with *E. piscicida* in the case of autophagy inhibition. Most of all, the treatment with CQ completely rescued the impaired survival rate of zebrafish larvae infected with *E. piscicida* caused by CD44a deficiency (Fig. 4f). Consistent with the results of CQ treatment, the autophagy inductor (rapamycin) promoted the bacterial proliferation and decreased the survival rate of zebrafish larvae infected with *E. piscicida* both in the WT and in the CD44a^−/−^-14del zebrafish (Fig. 4g, h). Compared with the WT zebrafish or WT zebrafish microinjected with FLAG, the antibacterial effects of CD44a_tv1 and CD44a_tv2 were completely abolished by rapamycin treatment (Fig. 4h).

Collectively, these results suggest that the antibacterial effects of zebrafish CD44a variants are dependent on the inhibition of autophagy.

### CD44a deficiency inhibits cell growth and proliferation via p53-mediated ccnb1 and ccnb3

**Fig. 5:**
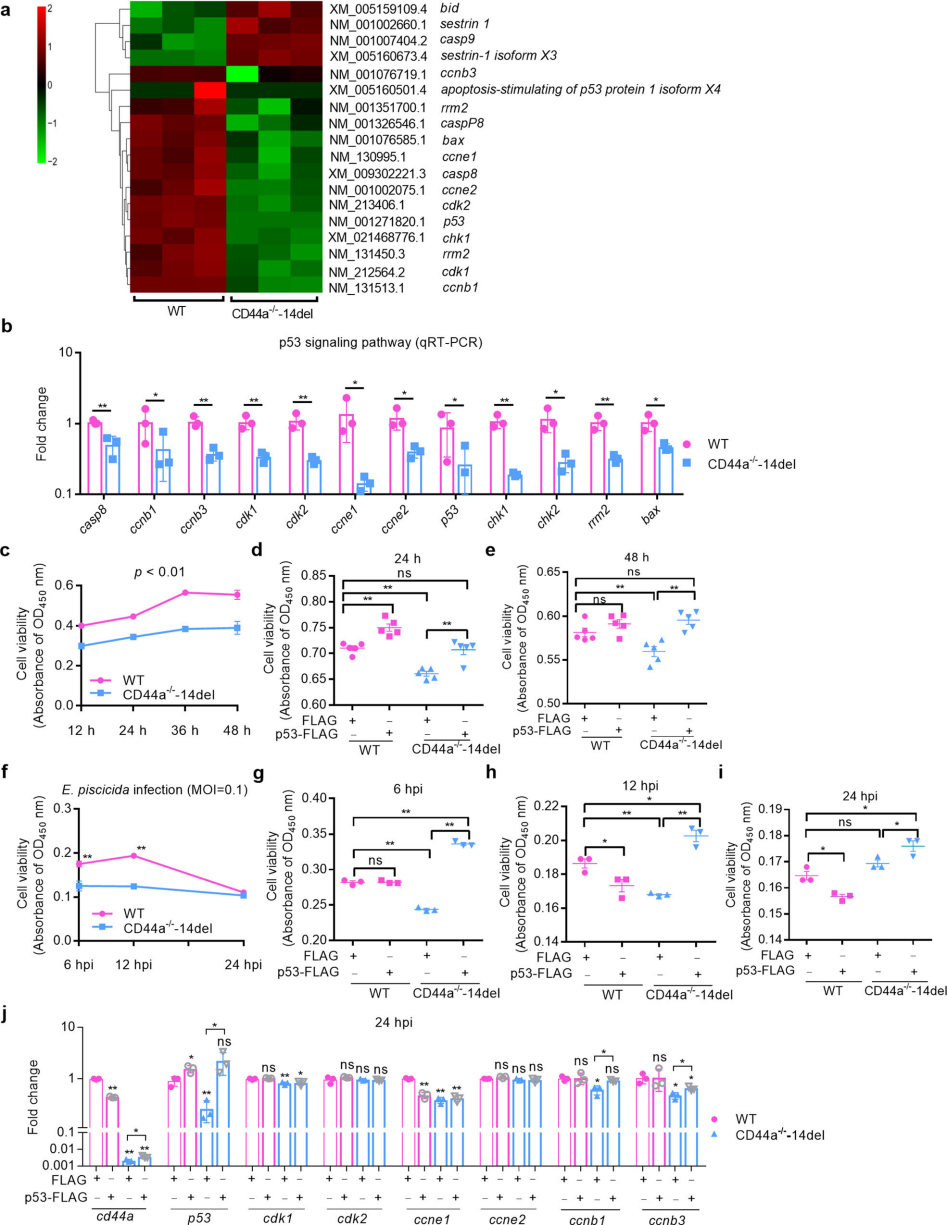
CD44a deficiency inhibits cell growth and survival. **a** DEGs involved in p53 signaling pathway at 24 hpi by RNAseq analysis of mRNA expression levels in the WT and CD44a^−/−^-14del zebrafish larvae infected with *E. piscicida*. **b** Validation of DEGs involved in p53 signaling pathway at 24 hpi by qRT-PCR. **c** Cell viability analysis of the WT and CD44a^−/−^-14del cells in the absence of infection. The cells were collected at 12, 24, 36 and 48 h. **d**, **e** Cell viability analysis of the WT and CD44a^−/−^-14del cells with or without the overexpression of p53. The cells were collected at 24 h (**d**) and 48 h (**e**). **f** Cell viability analysis of the WT and CD44a^−/−^-14del cells infected with *E. piscicida*. The cells were collected at 6, 12 and 24 hpi. **g**–**i** Cell viability analysis of the WT and CD44a^−/−^-14del cells with or without the overexpression of p53 following the *E. piscicida* infection. The cells were collected at 6 hpi (**g**), 12 hpi (**h**) and 24 hpi (**i**). **j** mRNA levels of CD44a and many genes involved in p53 signaling pathway in the WT and CD44a^−/−^-14del zebrafish with or without the overexpression of p53 following the *E. piscicida* infection. The cells were collected at 24 hpi. Data are presented as mean values ± SD (*n* = 3) and *p* values by Student’s *t* test are shown in **b**–**j**. *p < 0.05, **p < 0.01; ns not significant.

The regulation of CD44a deficiency on p53 signaling pathway was also verified by qRT-PCR. The results showed that zebrafish CD44a deficiency indeed impaired the expression of those genes involved in p53 signaling pathway including *casp8, ccnb1, ccnb3, cdk1, cdk2, ccne1, ccne2, p53, chk1, chk2, rrm2* and *bax*, which is 100% consistent with the results of transcriptome sequencing (Fig. 5a, b).

Mammalian p53 can affect cell cycle through transcriptional regulation. Since zebrafish CD44a deficiency significantly impairs p53 and cell cycle signaling pathways, we want to know whether zebrafish CD44a influences cell growth and proliferation through p53. The WT and CD44a^−/−^-14del cells from caudal fins of the WT and CD44a^−/−^-14del zebrafish at the age of 1 month (Supplementary Fig. 4a) were successfully subcultured as the stable cell lines, and majority of cells were in spindle-shaped forms. In uninfected WT cells, CD44a knockout significantly inhibited growth and cell viability (Fig. 5c). The overexpression of p53 in CD44a^−/−^-14del cell lines was beneficial to growth and cell viability, and even completely rescue the inhibition of CD44a on growth and cell viability (Fig. 5d, e). In zebrafish cells infected with *E. piscicida*, CD44a knockout could inhibit growth and cell viability at 6 and 12 hpi, but not at 24 hpi (Fig. 5f). Interestingly, overexpression of p53 in WT cells infected with *E. piscicida* inhibited cell growth and survival, however overexpression of p53 in CD44a^−/−^-14del cell lines promoted cell growth and survival (Fig. 5g–i). In all, the overexpression of p53 in CD44a^−/−^-14del cell lines can completely rescue the inhibition of CD44a on growth and cell viability in the case of the uninfected and infected conditions.

The effect of zebrafish CD44a on cell proliferation was performed in early developing embryos (2 dpf) from the WT and CD44a-deficient zebrafish using pH3 marker. Compared with the WT, mitotic (pH3+) cells were significantly lower in the CD44a-deficient zebrafish (Supplementary Fig. 4b, c), suggesting that the constitutive proliferative activity was impaired by CD44a deficiency during the early developmental stage in zebrafish. Compared with WT zebrafish microinjected with FLAG empty plasmid, the overexpression of p53 had no obvious effect on the proliferative activity. However the overexpression of p53 in CD44a^−/−^-14del zebrafish embryos partially rescued the impaired proliferative activity caused by CD44a deficiency (Supplementary Fig. 4d, e).

The target genes regulated by CD44a were further investigated through rescue experiments and qRT-PCR. Overexpression of p53 in CD44a^−/−^-14del zebrafish could restore the impaired expression of p53 in CD44a^−/−^-14del zebrafish. Overexpression of p53 in WT zebrafish had no significant effect for the expression of all tested genes including *cdk1, cdk2, ccne2, ccnb1* and *ccnb3*, except the decreased expression of *ccne1*. However, overexpression of p53 in CD44a^−/−^-14del zebrafish could increase the expressions of *ccnb1* and *ccnb3*, but not for *cdk1, cdk2, ccne1* and *ccne2* (Fig. 5j). The impaired expression of *ccnb1* was completely rescued by p53 overexpression, and the expression of *ccnb3* was partially rescued by p53 overexpression (Fig. 5j).

Collectively, these results suggest that zebrafish CD44a influences cell growth and proliferation via p53-mediated *ccnb1* and *ccnb3*.

### Zebrafish CD44a variants promote the translocation of p53 from the nucleus to the cytoplasm and interact with p53 in the cytoplasm

**Fig. 6:**
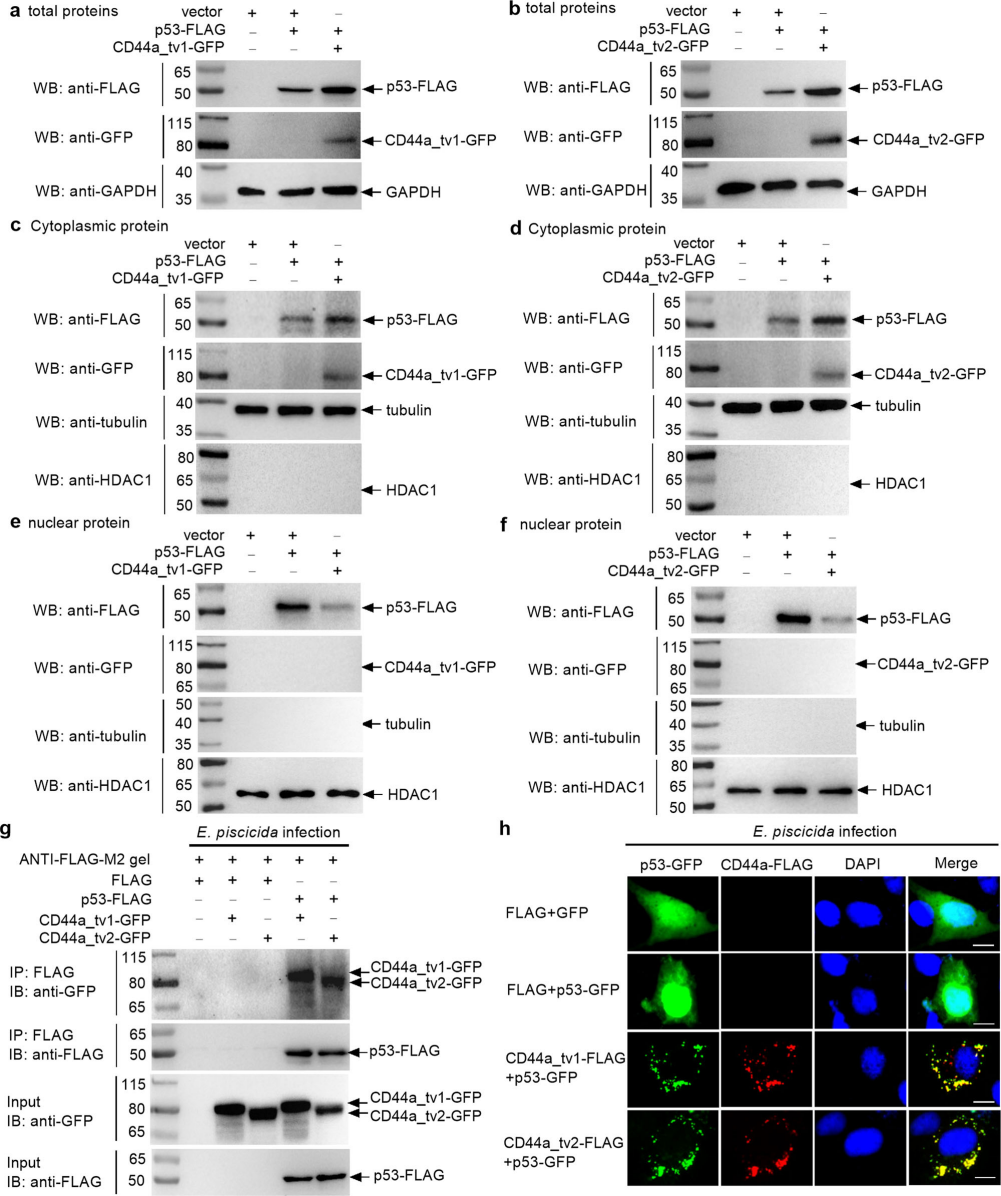
Zebrafish CD44a variants promote the translocation of p53 from the nucleus to the cytoplasm and interact with p53 in the cytoplasm. The effects of zebrafish CD44a_tv1 (**a**) and CD44a_tv2 (**b**) on the expression of total p53 proteins. The effects of zebrafish CD44a_tv1 (**c**) and CD44a_tv2 (**d**) on the expression of cytoplasmic p53 proteins. The effects of zebrafish CD44a_tv1 (**e**) and CD44a_tv2 (**f**) on the expression of nuclear p53 proteins. **g** The interaction between zebrafish CD44a variants and p53. For **a**–**g**, WB were repeated at least two times, and shown were the representative data. **h** The colocalization between zebrafish CD44a variants and p53 in the CD44a^−/−^-14del cells following the *E. piscicida* infection. Scale bar, 10 µm.

Many studies have shown that p53 has a dual role in modulating autophagy based on its localization. Since that the antibacterial effects of zebrafish CD44a variants are dependent on the inhibition of autophagy, and that CD44a deficiency impairs p53 and p53 signaling pathway, we are interested to know the correlation between zebrafish CD44a variants and nuclear or cytoplasmic p53. Cell fractionation experiments were firstly performed in the CD44a^−/−^-14del cell lines transfected with p53-FLAG with or without the cotransfection of CD44a_tv1 or CD44a_tv2 in the case of *E. piscicida* infection. Compared with the control group transfected with empty plasmid and p53-FLAG, addition of CD44a_tv1 or CD44a_tv2 significantly increased the expression levels of total p53 protein (Fig. 6a, b) and cytoplasmic p53 protein (Fig. 6c, d). However, the expression levels of nuclear p53 protein were enormously decreased by CD44a_tv1 or CD44a_tv2 (Fig. 6e, f).

To check whether zebrafish CD44a variants physically interact with p53, co-immunoprecipitation was performed in the CD44a^−/−^-14del cell lines transfected with the indicated various plasmids. No CD44a_tv1-GFP or CD44a_tv2-GFP band was observed, which confirmed that CD44a variants were not pull-downed by FLAG. However, the pull-downed CD44a_tv1-GFP or CD44a_tv2-GFP band by p53-FLAG was readily detected by immunoprecipitation analysis, which proved the interaction between zebrafish CD44a variants and p53 in the case of *E. piscicida* infection (Fig. 6g). The endogenous interaction between CD44a and p53 was also confirmed in zebrafish larvae without or with the infection of *E. piscicida* (Supplementary Fig. 5a, b).

To further explore the translocation of p53 from the nucleus to the cytoplasm by zebrafish CD44a variants, we examined the CD44a^−/−^14del cell lines transfected with the FLAG, GFP empty plasmid, p53-GFP, CD44a_tv1-FLAG or CD44a_tv2-FLAG with the indicated combination of two plasmids. The p53-GFP signal in the transfected cells was mainly in the nucleus, and weakly in the cytoplasm in the case of the uninfected and infected conditions (Supplementary Fig. 5c and Fig. 6h). However in the existence of CD44a_tv1 or CD44a_tv2, the p53-GFP signal in the transfected cells was mainly in the cytoplasm, with the sign of specific granular localization in the case of the uninfected and infected conditions (Supplementary Fig. 5c and Fig. 6h). Both CD44a_tv1 and CD44a_tv2 exhibited obvious co-localization with p53 in the cytoplasm in the case of the uninfected and infected conditions (Supplementary Fig. 5c and Fig. 6h). The co-staining between the endogenous p53 protein and mitochondria with or without the expression of zebrafish CD44a_tv1 or CD44a_tv2 were performed in the CD44a^−/−^-14del cell lines using zebrafish p53 antibody and MitoTracker Red. Similar to exogenous p53, the endogenous p53 failed to be observed in the nucleus in the existence of CD44a_tv1 or CD44a_tv2. However, the co-staining between the endogenous p53 protein and mitochondria was obviously enhanced by *E. piscicida* infection but not by zebrafish CD44a_tv1 or CD44a_tv2 (Supplementary Fig. 5d, e).

Collectively, these results suggest that zebrafish CD44a variants can translocate the nuclear p53 to the cytoplasm, and interact with p53 in the cytoplasm.

### The antibacterial effect of zebrafish CD44a_tv1 is depending on the CASP8-mediated apoptosis but not on the p53-mediated apoptosis

**Fig. 7:**
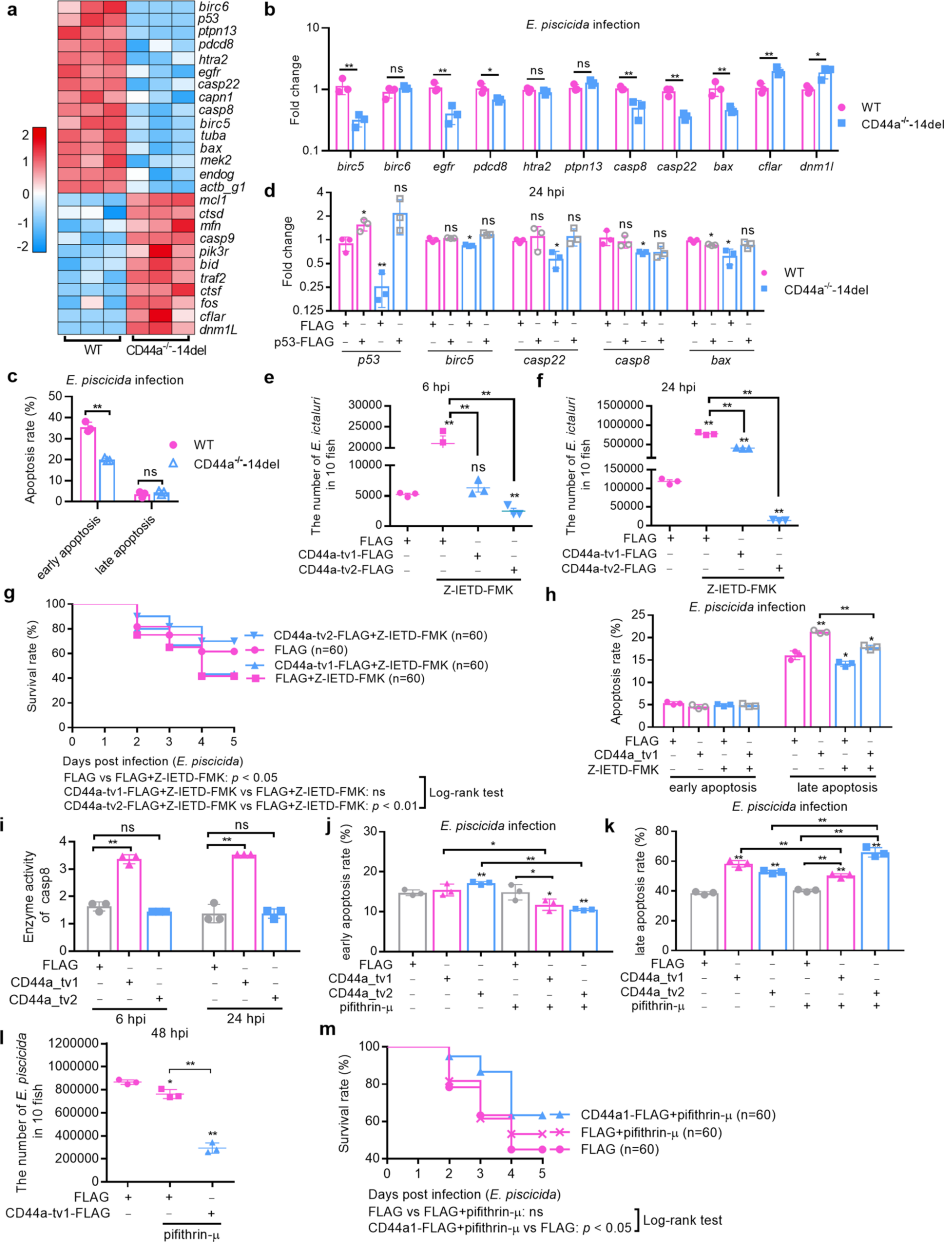
The antibacterial effect of zebrafish CD44a_tv1 depends on the CASP8-mediated apoptosis. **a** DEGs involved in apoptosis at 24 hpi by RNAseq analysis of mRNA expression levels in the WT and CD44a^−/−^-14del zebrafish larvae infected with *E. piscicida*. **b** Validation of DEGs involved in apoptosis at 24 hpi by qRT-PCR. **c** The effect of zebrafish CD44a deficiency on the early and late apoptosis rates at 6 hpi. **d** mRNA levels of many genes involved in apoptosis in the WT and CD44a^−/−^-14del zebrafish with or without the overexpression of p53 following the *E. piscicida* infection. The larvae were collected at 24 hpi. **e**, **f** Zebrafish larvae microinjected with FLAG, CD44a_tv1 or CD44a_tv2 without or with the treatment of Z-IETD-FMK were collected at the indicated post-infection time points, and homogenates were made for CFU counts. **g** Larval survival analysis in the WT zebrafish larvae microinjected with FLAG, CD44a_tv1 or CD44a_tv2 without or with the treatment of Z-IETD-FMK (*n* = 60 for each group). **h** The effect of Z-IETD-FMK on the CD44a_tv1-mediated apoptosis rates at 6 hpi. **i** The effect of zebrafish CD44a variants on the casp8 activity. **j** The effect of pifithrin-μ on the early apoptosis regulated by zebrafish CD44a variants at 6 hpi. **k** The effect of pifithrin-μ on the late apoptosis regulated by zebrafish CD44a variants at 6 hpi. **l** The effect of pifithrin-μ on the CD44a_tv1-mediated antibacterial activity. **m** The effect of pifithrin-μ on the CD44a_tv1-mediated larval survival rate (*n* = 60 for each group). Data are presented as mean values ± SD (*n* = 3) and *p* values by Student’s *t* test are shown in **b**–**f**, **h**–**l**. *p < 0.05, **p < 0.01; ns not significant.

The exact antibacterial mechanisms of zebrafish CD44a variants remain unclear. Next, whether zebrafish CD44a regulates apoptosis or apoptosis-related genes via p53 was further determined. The results from qRT-PCR showed that zebrafish CD44a deficiency regulated many apoptosis-related genes including *birc5*, *egfr*, *pdcd8*, *casp8*, *casp22*, *bax*, *cflar* and *dnm1l*, which is 72.7% consistent with the results of transcriptome sequencing (Fig. 7a, b). The results measured by flow cytometry showed that CD44a deficiency significantly inhibited the early apoptosis rate in the case of *E. piscicida* infection (Fig. 7c and Supplementary Fig. 6a). In the rescue experiments, we found that overexpression of p53 in CD44a^−/−^-14del zebrafish could completely restore the impaired expression of apoptosis-related genes such as *birc5*, *casp22*, *casp8* and *bax* (Fig. 7d).

Based on that CD44a deficiency inhibited the expressions of p53 and caspase genes, and that overexpression of p53 in CD44a^−/−^-14del zebrafish could restore the impaired expression of caspase genes, we speculated that the antibacterial mechanisms of zebrafish CD44a variants might be associated with the p53 and caspases. Z-IETD-FMK is a specific caspase-8 inhibitor. In wild zebrafish, treatment with Z-IETD-FMK decreased the survival rate of zebrafish larvae infected with *E. piscicida* (Supplementary Fig. 6b) with the increased bacterial loads (Fig. 7e, f), compared with the control group microinjected with the FLAG plasmid but in the absence of treatment. Unexpectedly, treatment with Z-IETD-FMK completely abolished the antibacterial effect of zebrafish CD44a_tv1. However, the antibacterial effect of zebrafish CD44a_tv2 failed to be abolished by Z-IETD-FMK treatment (Fig. 7e, f). Consistent with the bacterial loads in zebrafish larvae, CD44a_tv1-mediated beneficial effect for larvae survival were abolished by Z-IETD-FMK treatment, but not for zebrafish CD44a_tv2 (Figs. 1d and 7g). Furthermore, overexpression of zebrafish CD44a_tv1 increased levels of late apoptosis, and the induced late apoptosis by zebrafish CD44a_tv1 was significantly weakened by the treatment of Z-IETD-FMK (Fig. 7h and Supplementary Fig. 6c).

The regulations of zebrafish CD44a variants on the expression and activity of *casp8* were further investigated. In WT zebrafish, overexpression of CD44a_tv1 significantly increased the expression of *casp8*, but not for CD44a_tv2 (Supplementary Fig. 6d). Similar to the effect of zebrafish CD44a variants on the expression of *casp8*, overexpression of CD44a_tv1 in EPC cells significantly increased the casp8 activity both at 6 hpi and 24 hpi. No alterations were observed in EPC cells transfected with CD44a_tv2 when compared with the control cells transfected with the FLAG empty plasmid (Fig. 7i).

To distinguish the role of cytoplasmic and nuclear p53 on the expression of *casp8*, pifithrin-μ (an inhibitor of cytoplasmic p53) and pifithrin-α (an inhibitor of p53 transactivation) were used. Treatment of pifithrin-α significantly inhibited the expression of *casp8*. Different from pifithrin-α, pifithrin-μ had no significant effect on the expression of *casp8* (Supplementary Fig. 6e). The regulation of pifithrin-α on the expression of zebrafish CD44a variants was also examined. The mRNA levels of CD44a_tv1 but not for CD44a_tv2 were increased by the treatment of pifithrin-α (Supplementary Fig. 6f).

Since pifithrin-μ could block the interaction of p53 with antiapoptotic Bcl2/Bclxl^24^, the effects of pifithrin-μ on the CD44a-mediated apoptosis were next investigated. Overexpression of zebrafish CD44a_tv1 only increased levels of late apoptosis, however both early and late apoptosis increased by zebrafish CD44a_tv2. The treatments with pifithrin-μ significantly weakened the late apoptosis induced by zebrafish CD44a_tv1. Different from zebrafish CD44a_tv1, the treatments with pifithrin-μ significantly weakened the early apoptosis mediated by zebrafish CD44a_tv2, whereas enhanced the late apoptosis mediated by zebrafish CD44a_tv2 (Fig. 7j, k). These data suggest that the cytoplasmic p53 is involved in the increased apoptosis induced by zebrafish CD44a_tv1.

The effects of pifithrin-μ on the zebrafish CD44a_tv1-mediated antibacterial activity were next investigated. Compared with the control group microinjected with empty plasmid without pifithrin-μ treatment or the control group microinjected with empty plasmid with pifithrin-μ treatment, the antibacterial effect of zebrafish CD44a_tv1 was observed. Similar to previous study^25^, pifithrin-μ treatment slightly inhibited the proliferation of *E. piscicida*, but no significant differences observed for the survival rates of zebrafish larvae compared with the untreated control group (Fig. 7l, m). Furthermore, zebrafish CD44a_tv1-mediated beneficial effect for larvae survival were not affected by pifithrin-μ treatment (Fig. 7m).

Taken together, these results suggest that the antibacterial effect of zebrafish CD44a_tv1 is depending on the CASP8-mediated apoptosis but not on the cytoplasmic p53-mediated apoptosis.

### Zebrafish CD44a_tv2 increases p53 transcription and its antibacterial effect depends on the cytoplasmic p53-mediated inhibition of autophagy

**Fig. 8:**
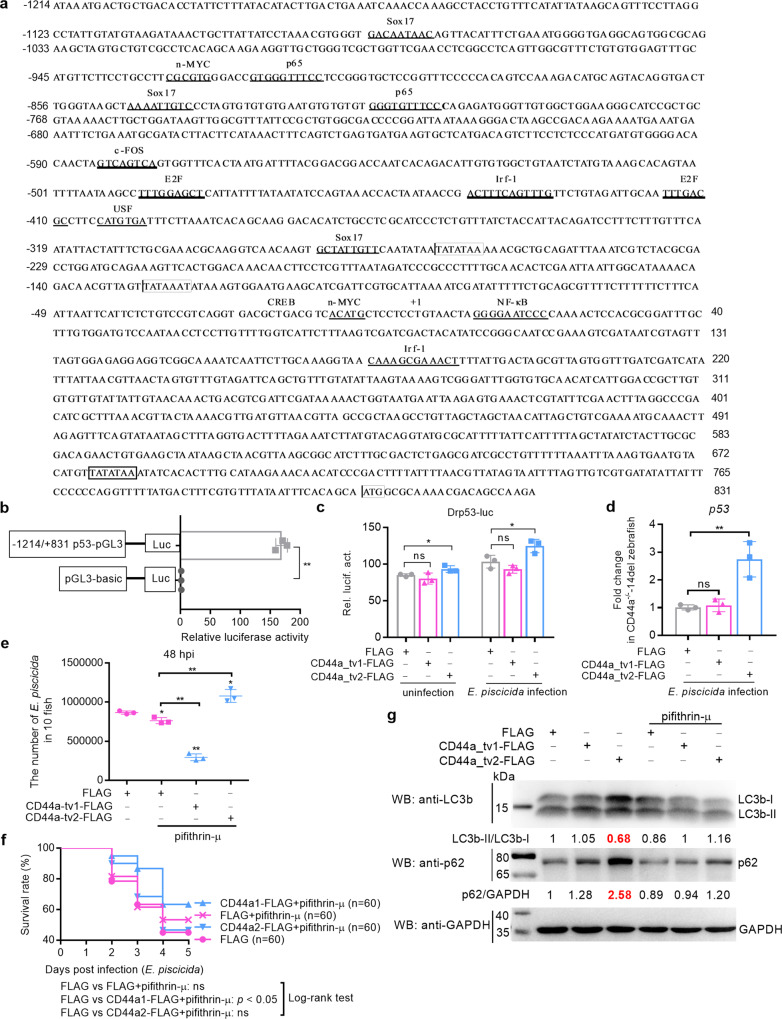
The antibacterial effect of zebrafish CD44a_tv2 depends on the cytoplasmic p53-mediated inhibition of autophagy. **a** The 5′ flanking regulatory sequence of zebrafish p53. The transcription factor binding sites were underlined. **b** The relative luciferase activity of p53-pGL3 promoter. **c** The effects of zebrafish CD44a variants on the promoter activity of p53 without or with the infection of *E. piscicida*. **d** The effects of zebrafish CD44a variants on the transcription of p53 in the CD44a^−/−^-14del cells. **e** Zebrafish larvae microinjected with FLAG, CD44a_tv1 or CD44a_tv2 without or with the treatment of pifithrin-μ were collected at 48 hpi, and homogenates were made for CFU counts. **f** Larval survival analysis in the WT zebrafish larvae microinjected with FLAG, CD44a_tv1 or CD44a_tv2 without or with the treatment of pifithrin-μ (*n* = 60 for each group). **g** Immunoblotting analysis and quantification of p62 and LC3b levels in the WT zebrafish microinjected with FLAG, CD44a_tv1 or CD44a_tv2 without or with the treatment of pifithrin-μ. WB were repeated at least two times, and shown were the representative data. Data are presented as mean values ± SD (*n* = 3) and *p* values by Student’s *t* test are shown in **b**–**e**. *p < 0.05, **p < 0.01; ns not significant.

p53 mRNA levels are tightly regulated. In zebrafish, the transcription of p53 was decreased by zebrafish CD44a deficiency (Fig. 5b). To make clear whether p53 is regulated by zebrafish CD44a_tv1 or/and CD44a_tv2, p53 promoter was constructed (Fig. 8a, b). To eliminate the interference of CD44a variants with each other, CD44a^−/−^-14del cell lines were used. Zebrafish CD44a_tv2, but not for zebrafish CD44a_tv1, significantly increased the p53 promoter activity in the case of the uninfected and infected conditions (Fig. 8c). Consistent with this, the transcription of p53 was increased only by zebrafish CD44a_tv2 (Fig. 8d).

Since zebrafish CD44a variants promoted the translocation of p53 from the nucleus to the cytoplasm, we further investigated whether the inhibition of cytoplasmic p53 affected CD44a variants-mediated antibacterial activity. The p53 inhibitor pifithrin-μ was used for blocking cytoplasmic p53 effects. Compared with the control group microinjected with empty plasmid without pifithrin-μ treatment or the control group microinjected with empty plasmid with pifithrin-μ treatment, the antibacterial effect of zebrafish CD44a_tv1 was observed. Different from zebrafish CD44a_tv1, the antibacterial effect of zebrafish CD44a_tv2 was completely abolished by pifithrin-μ treatment (Fig. 8e). Furthermore, zebrafish CD44a_tv1-mediated beneficial effect for larvae survival were not affected by pifithrin-μ treatment, but zebrafish CD44a_tv2 did. Compared with the control group microinjected with empty plasmid without pifithrin-μ treatment, no significant differences were observed for the survival rate of zebrafish larvae microinjected with zebrafish CD44a_tv2 with pifithrin-μ treatment (Fig. 8f).

Cytoplasmic p53 inhibits autophagy via protein-protein interactions in the mitochondria. To further confirm whether the antibacterial effects of zebrafish CD44a variants were related with the cytoplasmic p53-mediated inhibition of autophagy, Western blotting detection of LC3b and p62 proteins were performed on larvae extracts microinjected with FLAG empty plasmid, CD44a_tv1-FLAG or CD44a_tv2-FLAG. Compared with the control group microinjected with FLAG empty plasmid, overexpression of zebrafish CD44a_tv2-FLAG increased the p62 levels and decreased the LC3b-II/LC3b-I ratio, and the altered protein levels of p62 and LC3b-II/LC3b-I ratio are restored to normal by pifithrin-μ treatment (Fig. 8g). No differences in the protein levels of p62 and LC3b-II/LC3b-I ratio were observed between the control group microinjected with FLAG empty plasmid and the group microinjected with zebrafish CD44a_tv1-FLAG (Fig. 8g).

Collectively, these data suggest that the antibacterial effect of zebrafish CD44a_tv2 but not for CD44a_tv1 depends on the cytoplasmic p53-mediated inhibition of autophagy.

## Discussion

Most previous studies concerning CD44 variants have focused on the functions of mammalian CD44 standard (CD44s) and specific CD44 variant (CD44v) isoforms in cancer progression^16,26–28^. Although many studies have implicated that mammalian CD44 is also involved in host defense against invading pathogens^29^, the detrimental role of CD44 in host defense has been observed for some bacterial pathogens such as *Listeria monocytogenes*, *Escherichia coli*, *Streptococcus pneumoniae*, *Klebsiella pneumoniae, E. piscicida*^19,30–33^. In the present study, our data suggest that CD44a is critical in mediating protective immune response against *E. piscicida* infection in zebrafish. Two variants of CD44a were identified in zebrafish. Since the seven basic modes of alternative splicing including exon skipping, intron retention, alternative 3’ss selection, alternative 5’ss selection, mutually exclusive exons, alternative promoters and alternative poly(A) are not suitable for the generation of zebrafish CD44a_tv1 and CD44a_tv2, we speculate that they are generated not by alternative splicing but by gene replication. According to the alignment, it was found that there was no significant difference in the composition of important domain between zebrafish CD44a_tv1 and CD44a_tv2. However the analysis of zebrafish CD44a_tv1 and CD44a_tv2 sequences revealed the existences of 1 N-glycosylated site for CD44a_tv1 and 2 N-glycosylated sites for CD44a_tv2. In mammals, many studies have shown that differential glycosylations of CD44 are sufficient to influence its recognition of hyaluronan, and have variable effects on CD44 function depending on the nature of the N-glycans^34,35^. How the difference of glycosylation levels between zebrafish CD44a_tv1 and CD44a_tv2 affects their antibacterial activity will need to be further investigated.

PRRs including Toll-like receptors (TLRs), NLRs and RIG-I-like receptors play pivotal roles in mounting innate immune responses against infections from various bacteria, fungi or virus^36,37^. In mammals, the correlation between CD44 and TLRs-induced inflammatory response has been revealed. CD44 can directly associate with TLR2, and suppress TLR-mediated inflammation by its cytoplasmic domain^38^. CD44 also prevents exaggerated inflammatory responses to LPS by the negative regulation of TLR4 signaling^39^. In teleost, our previous study showed that NOD1-RIPK2 signaling regulated the expression of CD44a^19–21^, however the role of the correlation between CD44 and NOD1 in resisting pathogen infections is unclear. In the present study, we found that CD44a deficiency impaired the expression of NOD1. However the loss of CD44a did not affect the antibacterial function of NOD1, and vice versa. All these data suggest that the antibacterial effects of CD44a and NOD1 are independent of each other, although mutual regulations exist between CD44a and NOD1.

**Fig. 9:**
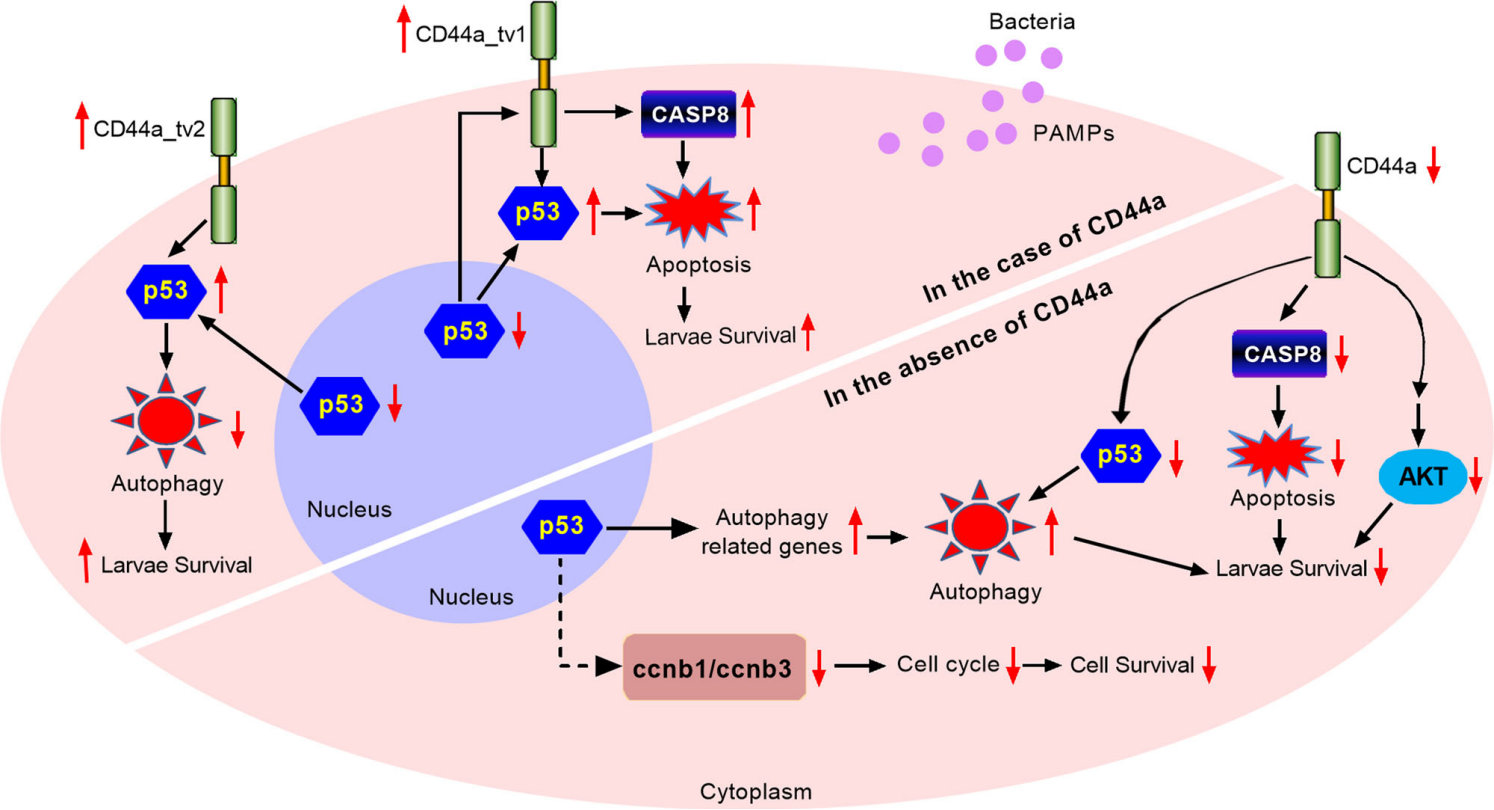
Proposed model illustrating the different antibacterial mechanisms of zebrafish CD44a variants during *E. piscicida* infection. CD44a deficiency regulates the expression levels of many genes involved in p53 signaling, apoptosis and autophagy, which leads to inhibition of apoptosis and induction of autophagy in the case of *E. piscicida* infection. In zebrafish, two CD44a variants including CD44a_tv1 and CD44a_tv2 are identified. The zebrafish CD44 variants exert protective effects against *E. piscicida* infection. Both zebrafish CD44a_tv1 and CD44a_tv2 can promote the nucleoplasmic translocation of p53 and interact with p53 in the cytoplasm. The antibacterial effect of zebrafish CD44a_tv1 is depending on the CASP8-mediated apoptosis, whereas the antibacterial effect of zebrafish CD44a_tv2 is dependent of the cytoplasmic p53-mediated inhibition of autophagy.

Many studies have shown that mammalian CD44 isoforms are co-receptors of Met, EGFR and TGFβ receptors to activate downstream signaling pathways including PI3K-Akt, Ras and MAPK to promote cell proliferation and survival^40^. Uncontrolled cell proliferation is a hallmark of cancer cells. In thyroid cancer cells, the intracellular protein fragment CD44-ICD released by CD44 proteolytic cleavage translocates to the nucleus, and enhances the binding of transcription factor CREB with the cyclin D1 promoter to promote cyclin D1 transcription and cell proliferation^41^. In gastric cancer, CD44 regulates stem cell proliferation by increasing cyclin D1 expression, and the expressions of CD44 and cyclin D1 are positively correlated with tumor differentiation^42^. The present study showed that CD44a deficiency impaired two cell cycle signaling pathways and the expressions of many genes including cell cycle proteins (*ccnb1*, *ccnb1*, *ccne1* and *ccne2*), cyclin-dependent kinases (*cdk1* and *cdk2*) and cell cycle checkpoint kinases (*chk1* and *chk2*). Ultimately, the proliferation abilities of CD44a^−/−^-14del cell lines or mitotic (pH3+) cells from CD44a^−/−^-14del zebrafish embryos were significantly decreased. The function of p53 as a tumor suppressor is intricate in permanently inhibiting cell proliferation, promoting cell death, or contributing to cell survival through the regulation of various signaling pathways^43^. Interesting, CD44a deficiency decreased the expression of p53, and the impaired proliferation abilities of CD44a^−/−^-14del zebrafish embryos and expression levels of *ccnb1* and *ccnb1* were rescued by p53 overexpression, which suggest that zebrafish CD44a influences cell survival via p53-mediated *ccnb1* and *ccnb3* (Fig. 9). A previous study showed that high CD44 expression may counteract p53’s antiproliferative action and serve as an important growth-promoting and survival factor in early stages of tumor progression^44^. When placed in the context of previous report, the present data suggest that in the existence of CD44 function, p53 contributes to inhibit cell survival at a disadvantage under pathogen infections or in tumorigenesis. It is worth noting that in the absence of CD44 function, overexpression of p53 promoted cell survival in the case of the uninfected and infected conditions, which indicate that p53 may compensate the function of CD44 in the regulation of cell growth and survival.

The pro-apoptotic or antiapoptotic signals mediated by mammalian CD44 and its variants are involved in many biological processes such as T-cell maturation, activation and tumorigenesis^16,45,46^. Many studies have suggested that apoptosis may be a defense mechanism of host cells or strategy for pathogen survival^47,48^. Caspases are central components of apoptotic machinery. The initiator caspases including caspase-2, -8, -9, and -10 activate the effector caspases including caspase-3, -6 and -7. Once activated, the effector caspases are responsible for the proteolytic cleavage of cellular targets, which ultimately lead to cell death^49^. In this study, zebrafish CD44a deficiency promoted the proliferation of *E. piscicida*, inhibited apoptosis and impaired the expression levels of many apoptosis-related genes including *casp8* (caspase-8), which were consistent with the results that treatment with the specific inhibitor of caspase-8 reduced the resistance of WT zebrafish against *E. piscicida* infection. p53 also regulates apoptosis in the transcription-dependent and -independent manners^50^. After the impaired expression of p53 was rescued in the CD44a-knockout zebrafish, the decreased transcription of *birc5*, *casp22*, *casp8* and *bax* in the CD44a-knockout zebrafish infected with *E. piscicida* was restored to normal levels, indicating that p53 was involved in the regulation of CD44a-mediated apoptosis signals through transcription-dependent manner (Fig. 9).

In addition to inhibit apoptosis, zebrafish CD44a deficiency also regulated the transcriptions of many autophagy-related genes and promoted the formation of autophagosomes. Autophagy is frequently a cytoprotective response that occurs in order to restore homeostasis and stave off apoptosis, such as under conditions of nutrient depletion^51^. In response to pathogens infection, autophagy may promote tolerance of pathogens or trigger immunosuppression. For example, a deficiency in the autophagy gene Atg16L1 mediated an enhanced immune response during extracellular intestinal *Citrobacter rodentium* infection, which was associated with the expression of NOD2 and monocyte function^52^. Deletion of autophagy genes including *Epg5*, *Atg14*, *FIP200*, *Atg5* and *Atg7* led to influenza resistance^53^. Previous studies have revealed that p53 regulates autophagy, which is directly driven by its subcellular localization^54^. It is generally believed that cytoplasmic p53 inhibits autophagy via protein-protein interactions in the mitochondria, whereas nuclear p53 induces autophagy by transactivating its target genes^55–57^. Similar to CD44a deficiency, blocking p53 transactivation in zebrafish induced the transcription of autophagy-related genes, indicating that both p53 and CD44a are involved in the regulation of autophagy-related genes. Further research is needed to investigate whether the regulation of CD44a on these autophagy-related genes is mediated by p53 in transcription-dependent manner.

The present study and our previous report^25^ revealed the protective effects of autophagy inhibition and apoptosis induction in resisting *E. piscicida* infection in zebrafish. This study further characterizes the exact mechanisms of zebrafish CD44a variants in resisting *E. piscicida* infection. In mammals, ligation of CD44 with the A3D8 antibody induced apoptosis, which was initiated principally through caspase-8 activation^58,59^. In zebrafish, overexpression of zebrafish CD44a_tv1 but not for CD44a_tv2 increases the transcription of *casp8* in the case of *E. piscicida* infection. Treatment with the specific inhibitor of caspase-8 completely abolishes the antibacterial effect of zebrafish CD44a_tv1 and weakened the apoptosis induced by zebrafish CD44a_tv1. Different from the specific inhibitor of caspase-8, treatments with the inhibitor of cytoplasmic p53 failed to block the antibacterial effect of zebrafish CD44a_tv1, although pifithrin-μ also significantly weakened the apoptosis induced by zebrafish CD44a_tv1. These data reveal that the cytoplasmic p53 is involved in the increased apoptosis induced by zebrafish CD44a_tv1, but the antibacterial effect of zebrafish CD44a_tv1 is depending on the CASP8-mediated apoptosis but not on the p53-mediated apoptosis. Furthermore, overexpression of zebrafish CD44a_tv1 results in the decreased protein levels of nuclear p53. The decreased nuclear p53 further increases the expression of zebrafish CD44a_tv1, thereby forming a positive feedback loop between the zebrafish CD44a_tv1 and p53. Similar to zebrafish CD44a_tv1, zebrafish CD44a_tv2 promotes the translocation of p53 from the nucleus to the cytoplasm, and leads to the accumulation of the cytoplasmic p53. However blocking cytoplasmic p53 effects completely abolishes the antibacterial effect of zebrafish CD44a_tv2 and autophagy inhibition caused by zebrafish CD44a_tv2, which suggest that the antibacterial effect of zebrafish CD44a_tv2 is depending on the cytoplasmic p53-mediated inhibition of autophagy.

In summary, the present report provides strong evidences that zebrafish CD44 variants exert protective effects against *E. piscicida* infection through different mechanisms. Both zebrafish CD44a_tv1 and CD44a_tv2 can promote the nucleoplasmic translocation of p53 and interact with p53 in the cytoplasm, however only the antibacterial effect of zebrafish CD44a_tv2 is dependent of the cytoplasmic p53-mediated inhibition of autophagy. Different from zebrafish CD44a_tv2, the antibacterial effect of zebrafish CD44a_tv1 is depending on the CASP8-mediated apoptosis (Fig. 9). Interestingly, in other studies, regulation of CD44 splicing and expression is vital for p53 function^40,60^. CD44 can control tumor initiation by terminating the p53-mediated DNA damage response^60^. Different from the antagonistic interplay between p53 and CD44 reported previously^44,61,62^, the present results demonstrate that p53 and p53 signaling pathway are vital for CD44 antibacterial function during *E. piscicida* infection. Furthermore, the present study firstly demonstrates that CD44 variants can promote p53 nuclear export and cytoplasmic accumulation of p53. Since proper regulation of p53 nuclear export has important functional consequences, it will be intriguing to further investigate the molecular mechanism by which CD44 variants localized at the plasma membrane facilitates p53 nuclear/cytoplasmic trafficking.

## Methods

### Generation of CD44a-knockout and NOD1-knockout mutants by CRISPR/Cas9 genome editing

Two CD44a-knockout mutants (CD44a^−/−^-14del and CD44a^−/−^-10IS) were generated by CRISPR/Cas9 system in the zebrafish strain AB/TU. The target site sequence (GGTGAACTGTGTCAGAGTTT) is located within the exon 2 of CD44a. The primers of detecting mutants were 5’-TGCACATGCACTGAATCATTAGG-3’ (forward) and 5’-GCTCTGGGCTAGGGCTATTGTAC-3’ (reverse). The NOD1-knockout mutants (NOD1-1IS^–/−^) were obtained from the China Zebrafish Resource Center^20^. The wild-type AB/TU (WT) zebrafish and the homozygotic mutants were bred, raised and maintained at 28 °C in cultivation systems according to standard protocols. All animal experiments were conducted in accordance with the Guiding Principles for the Care and Use of Laboratory Animals and were approved by the Institute of Hydrobiology, Chinese Academy of Sciences.

### Bacteria

The pathogenic bacterium used for zebrafish larvae and cell infection was *Edwardsiella piscicida* (PPD130/91 strain) or GFP-*E. piscicida*, which were cultivated at 28 °C in tryptic soy broth (TSB, BD Biosciences).

### Plasmid construction and sequence analysis

The ORFs of zebrafish CD44a variants and p53 were amplified by primers CD44a-F/ CD44a-R and p53-F/ p53-R, and cloned into the p3×FLAG-CMV-14 (Sigma-Aldrich) or pTurboGFP-N vector (Everogen). The primer sequences used for PCR amplification are listed in Supplementary Table 1.

Conserved domains of zebrafish CD44a variants were predicted using CD-Search (https://www.ncbi.nlm.nih.gov/Structure/cdd/wrpsb.cgi). N-glycosylated sites were predicted using NetNGlyc. Phylogenetic trees were constructed using the N-J method within the MEGA (version 4.1) package. Accession numbers of CD44 proteins from different species used for phylogenetic analysis are as follows: zebrafish CD44a_tv1, URX64905; zebrafish CD44a_tv2, URX64906; zebrafish CD44 antigen isoform X1, XP_017209790; zebrafish CD44 antigen isoform X2, XP_009296512; zebrafish CD44 antigen isoform X3, XP_017209791; zebrafish CD44 antigen isoform X4, XP_017209792; zebrafish CD44 antigen isoform X5, XP_017209793; zebrafish CD44c, MK396077; common carp CD44 antigen, XP_018937272; common carp CD44 antigen-like, XP_018963515; rainbow trout CD44 antigen, XP_021435669; rainbow trout CD44 antigen-like, XP_021456332; channel catfish CD44, XP_017341117; Atlantic salmon CD44 antigen-like, XP_014003799; African clawed frog CD44, ABA25895; chicken CD44, NP_990191.2; human CD44, ACI46596; mouse CD44, CAA46883.

### Antibodies and chemicals

Antibodies against the following proteins were used in this study: p62 (P0067, Sigma-Aldrich), LC3b (L7543, Sigma), GAPDH (60004-1-Ig, proteitech), FLAG (F3165, Sigma-Aldrich), GFP (AB513, Evrogen), p53 (ET1601–13, HuaAn Biotechnolog), phospho-Histone H3 (ab11477, abcam), phospho-Akt (#4060S, Cell Signaling), Akt (#9272, Cell Signaling), phospho-GSK-3β (#9323, Cell Signaling), GSK-3β (#9315, Cell Signaling), β-tubulin (ab6046, Abcam), HDAC1 (ab41407, Abcam) and zebrafish CD44a (25340-1hz, Abmart). Secondary antibodies for immunoblotting or immunofluorescence used in this study: goat anti-rabbit IgG and goat anti-mouse IgG (Prod #31460 and #31430, pierce), ReadyProbes™ Alexa Fluor® 594 Goat Anti-Mouse IgG Antibody (#R37121, Invitrogen), Alexa Fluor™ 488 Goat anti-Rabbit IgG (#A11008, Invitrogen) and Alexa Fluor™ 488 (#A110011, Invitrogen) Goat anti-Mouse IgG. The chemicals used in this study: Z-IETD-FMK (50 μm; #S7314, Selleck), pifithrin-μ (50 μm; #S2930, Selleck), pifithrin-α (5 μm; #S2929, Selleck), rapamycin (200 nm; #S1039, Selleck) and CQ (100 μm; #C6628, Sigma-Aldrich) and MitoTracker Deep red (Invitrogen™, M22426).

### Cell culture and transfection

Primary cell cultures were developed from caudal fins of WT and CD44a^−/−^-14del zebrafish at the age of 1 month, and were subcultured to the stable cell lines (beyond 40 passages) designated as the WT or CD44a^−/−^-14del caudal fin-derived cell lines by the tissue block adherent method. The cells were growing and passage in DMEM/F12 (1:1) medium (Life Technologies) supplemented with 10% FBS at 28 °C. EPC (*Epithelioma papulosum cyprini*) cells were grown in medium 199 (M199) supplemented with 10% FBS at 28 °C. For transient transfection, Lipofectamine 2000 (Invitrogen, Carlsbad, CA, USA) were used according to the manufacturer’s instructions.

### Cell viability analysis

Cell viability was measured with Cell Counting Kit-8 (CCK-8, Biyotime, China). WT and CD44a^−/−^-14del cells were plated at a density of 2 × 10^4^ per well in 96-well plate overnight and then infected with *E. piscicida* (MOI = 0.1) or left untreated. At 12, 24, 36 and 48 h for uninfected cells, and at 6, 12 and 24 hpi for infected cells, 10 μl CCK-8 was added to each well, and was incubated for 4 h. The absorbance at 450 nm was measured using a microplate reader (Synergy™ Neo2 Multi-Mode Microplate Reader, Bio-Tek, USA). To determine the role of p53 in CD44a-mediated cell viability and survival, WT and CD44a^−/−^-14del cells were transiently transfected with FLAG or p53-FLAG. After 24 h later, the transfected cells were plated at a density of 2 × 10^4^ per well in 96-well plate. Another 24 h later, the cells were infected with *E. piscicida* (MOI = 0.1) or left untreated. The viability of these cells was measured as described above.

### Bacterial infection in zebrafish larvae

To investigate the effect of *E. piscicida* infection on the expression of CD44a variants, the hatched WT zebrafish larvae at 4 dpf were infected with 2 × 10^8^ CFU/ml *E. piscicida*. To explore the possible effect of CD44a variants in bacterial infection, p3×FLAG, CD44a_tv1-FLAG and CD44a_tv2-FLAG were microinjected into one- or two-cell stage embryos from WT zebrafish. To explore whether the antibacterial effects of CD44a variants are associated with NOD1, rescue experiments were performed in the homozygotic NOD1 or CD44a mutants, respectively. These plasmids including p3×FLAG, CD44a_tv1-FLAG or CD44a_tv2-FLAG were microinjected into one- or two-cell stage embryos from the WT or NOD1-1IS^−/−^ zebrafish. The p3×FLAG or NOD1-FLAG plasmids were microinjected into one- or two-cell stage embryos from the WT or CD44a^−/−^-14del zebrafish. The hatched larvae at 4 dpf were infected with 2 × 10^8^ CFU/ml *E. piscicida*. To explore the effects of CD44a deficiency on the bacteria proliferation and larvae survival, the WT, CD44a^−/−^-14del and CD44a^−/−^-10IS zebrafish larvae at 4 dpf were infected with 2 × 10^8^ CFU/ml *E. piscicida*. To explore whether the inhibition of autophagy could rescue the CD44a^−/−^-mediated effects, the hatched larvae from WT and CD44a^−/−^-14del mutants were treated with the autophagy inhibitor CQ for 12 h, and then infected with 2 × 10^8^ CFU/ml *E. piscicida*. To explore whether p53 overexpression could rescue the expression levels of many genes involved in p53 signaling pathways and apoptosis, the p3×FLAG or p53-FLAG were microinjected into one- or two-cell stage embryos from the WT or CD44a^−/−^-14del zebrafish. The hatched larvae at 4 dpf were infected with 2 × 10^8^ CFU/ml *E. piscicida*. To explore the effects of rapamycin, pifithrin-μ and Z-IETD-FMK on the bacteria proliferation and larvae survival, the p3×FLAG, CD44a_tv1-FLAG or CD44a_tv2-FLAG were microinjected into one- or two-cell stage embryos from the WT zebrafish. The hatched larvae at 4 dpf were treated with rapamycin, pifithrin-μ and Z-IETD-FMK for 12 h, and then infected with 2 × 10^8^ CFU/ml *E. piscicida*.

For bacteria proliferation analysis, ten larvae per group were collected at 6, 24 and/or 48 hpi, and used for colony counting. For larval survival analysis, exposures were performed with three repetitions for each group, and the numbers of each repetition were 20–40 larvae. For expression analysis, ten larvae per group were collected at 24 and/or 48 hpi, and used for qRT-PCR.

### cDNA library construction and Illumina deep sequencing

The WT and CD44a^−/−^-14del zebrafish larvae at 4 dpf were infected with 2 × 10^8^ CFU/ml *E. piscicida* or left untreated. At 24 hpi, 48 hpi and 7 dpf, 150 larvae per group at each time point were collected, and used for Illumina deep sequencing. Total RNA was extracted using the TRIzol® Reagent (Invitrogen). The mRNA was isolated with the TruseqTM RNA sample prep Kit (Illumina, California, USA) following the manufacturer’s protocol. To identify DEGs between the WT and CD44a^−/−^-14del zebrafish, the expression levels were evaluated by using numbers of fragments per kilobase of transcript per million fragments sequenced (FPKM). The DEGs in each group were identified using DESeq2, based on false discovery rate <0.05, log_2_FC (fold change (CD44a^−/−^-14del /WT) for a gene) >1 or log_2_FC < −1.

### qRT-PCR

Total RNA from zebrafish larvae, collected at 5 and 6 dpf without bacterial infection or at 24 and/or 48 hpi with the *E. piscicida* infection, was extracted using Trizol reagent (Invitrogen). The first-strand cDNA was generated from total RNA using RevertAid First Stand cDNA Synthesis Kit (Thermo Fisher Scientific). Quantitative real-time PCR (qRT-PCR) was performed using SYBR^®^ Green Master Mix (Bio-RAD) on a BIO-RAD CFX96 Real-Time System according to the procedure as follows: 95 °C for 3 min, followed by 45 cycles of 95 °C for 10 s, 56–60 °C for 20 s, and 72 °C for 30 s. Reactions were performed in triplicate runs for each sample and were normalized to the level of glyceraldehyde 3-phosphate dehydrogenase (GAPDH). The sequences of primer pairs used in this study are listed in Supplementary Table 1 or our previous reports^19,20,63^. The relative gene expression levels were calculated based on the 2^−ΔΔCt^ method.

### Co-IP and western blotting

To determine the possible interaction between CD44 variants and p53, 1 × 10^6^ EPC cells were plated into 6-well plates overnight and then transfected with the indicated plasmids. The transfected cells were infected with *E. piscicida* at an MOI of 1. At 6 hpi, the cells were collected and lysed in IP lysis buffer (Thermo Scientific™) containing a Protease Inhibitor cocktail. Co-IP assay was performed using FLAG-tagged Protein Immunoprecipitation Kit (Sigma-Aldrich) according to the manufacturer’s protocol. The immunoprecipitate and input proteins were subjected to Western blotting using anti-FLAG (1:5000) and anti-pTurboGFP (1:5000) antibodies. To determine the endogenous interaction between zebrafish CD44a and p53, the WT larvae at 4 dpf were infected with *E. piscicida* or left untreated. Co-IP was carried out using the Thermo Scientific Pierce Co-IP Kit (#26149) according to the manufacturer’s manual. The zebrafish p53 antibody was first immobilized with AminoLink Plus Coupling Resin using Coupling Buffer overnight. The resin was then washed and incubated with the lysate from 50 larvae each group. After incubation, the resin was washed and protein eluted using Elution Buffer (E6150, Sigma-Aldrich). Total lysate and eluted proteins were analyzed by Western blot analysis with anti-CD44a (1: 500) and anti-p53 (1:1000) antibodies.

To investigate the regulation of CD44a variants on the total proteins of p53 in the case of *E. piscicida* infection, CD44a^−/−^-14del cell lines were used in order to eliminate the interference of CD44a variants with each other. CD44a^−/−^-14del cells plated at a density of 1 × 10^6^ per well into 6-well plates overnight were transfected with the indicated plasmids, and infected with *E. piscicida* at an MOI of 1. At 6 hpi, the cells were used for protein extraction. To investigate the regulation of CD44a variants on the nuclear and cytoplasmic proteins of p53 in the case of *E. piscicida* infection, CD44a^−/−^-14del cells transfected with the indicated plasmids were infected with *E. piscicida* at an MOI of 1. At 6 hpi, the cells were collected and used for preparation of nuclear and cytoplasmic extracts using a Subcellular Protein Fractionation Kit (Thermo Scientific™, #78840). Lysates were subjected to SDS-PAGE. For immunoblotting, the anti-HDAC1 (1:5000), anti-tubulin (1:5000), anti-FLAG (1:5000), and anti-pTurboGFP (1:5000) antibodies were used. The same amounts of lysates were loaded on each well and probed with anti-GAPDH antibody (1:5000) as a loading control.

To investigate the regulation of CD44a deficiency on the autophagy, 40 larvae per group collected at 24 hpi were used for protein extraction. To investigate the effects of CD44a variants on the autophagy, the p3×FLAG, CD44a_tv1-FLAG or CD44a_tv2-FLAG were microinjected into one- or two-cell stage embryos from the WT zebrafish. The hatched larvae at 4 dpf were treated with pifithrin-μ or left untreated. After 12 h treatment, these larvae were infected with *E. piscicida* at an MOI of 1. About 40 larvae per group collected at 24 hpi were used for protein extraction. Lysates were subjected to SDS-PAGE, and the protein expressions of p62, LC3b and GAPDH were examined using anti-p62 (1:2000), anti-LC3b (1:2000) and anti-GAPDH (1:5000) antibodies.

### Promoter construction and luciferase activity assay

Based on zebrafish p53 DNA sequences (ENSDARG00000035559), the 2045 bp of p53 promoter region (nt −1214 to +831 bp) were cloned by PCR with the primers PGL3-p53-F/PGL3-p53-R and inserted into pGL3-basic luciferase reporter vector (Promega). A bioinformatics tool ConSite (http://consite.genereg.net/cgi-bin/consite) was used to predict transcription factor binding sites. The primer sequences are listed in Supplementary Table 1.

To validate the activity of p53 promoter, 2.5 × 10^5^ EPC cells seeded in 24-well plates overnight were transfected with 25 ng Renilla (Promega) and 500 ng p53 promoter or pGL3-basic luciferase reporter vector. After 48 h, the cells were collected and lysed. The luciferase activity of lysates was assayed with the Dual-Luciferase reporter assay kit (Promega) by a Junior LB9509 luminometer (Berthold, Pforzheim, Germany). To determine the effect of CD44a variants on the p53 promoter activation, 2.5 × 10^5^ EPC cells seeded in 24-well plates overnight were transfected with 250 ng p53 promoter and 25 ng Renilla, together with 250 ng p3×FLAG, CD44a_tv1-FLAG or CD44a_tv2-FLAG. The transfected cells were infected with *E. piscicida* at an MOI of 0.1 or left untreated. At 6 hpi, the cells were lysed. The luciferase activity of lysates were measured with the Dual-luciferase Reporter Assay System.

### Apoptosis analysis

The WT and CD44a^−/−^-14del cells were seeded in 6-well plates overnight and then infected with *E. piscicida* at an MOI of 10. After 6 hpi, the cells were harvested, and then suspend in 1×Binding Buffer at a concentration of 1 × 10^6^ cells/ml. The cells were stained with annexin V FITC (BD Biosciences) according to the instructions of the manufacturer, and fluorescent cells were counting on a CytoFLEX LX Flow Cytometer (Beckman, USA). The data were analyzed using the software CytoExpert. Cells were identified as follows: early apoptotic cells (LR) if they were positive for annexin V-FITC but negative for PI, late apoptotic cells (UR) if they were positive for annexin V-FITC and PI, dead cells (UL) if they were negative for annexin V-FITC and positive for PI, and live cells (LL) if they were negative for annexin V-FITC and for PI. The LR, UR and UL were expressed as a percentage of the total cell counts.

To determine the inhibition of caspase-8 on the zebrafish CD44a_tv1-mediated apoptosis, EPC cells were seeded in 6-well plates overnight and then transfected with FLAG or CD44a_tv1-FLAG. After 48 h, the transfected cells were infected with *E. piscicida* for 1 h at an MOI of 10, and then treated with 20 μM Z-IETD-FMK or left untreated. To determine the inhibition of cytoplasmic p53 on the zebrafish CD44a_tv1-mediated or CD44a_tv2-mediated apoptosis, EPC cells seeded in 6-well plates overnight were transfected with FLAG, CD44a_tv1-FLAG or CD44a_tv2-FLAG. After 48 h, the transfected cells were infected with *E. piscicida* for 1 h at an MOI of 10, and then treated with 10 μM pifithrin-μ or left untreated. At 6 hpi, these cells were collected for apoptosis analysis using flow cytometry.

### Caspase-8 activity assay

To investigate the effect of zebrafish CD44a_tv1 and CD44a_tv2 on casp8 activity, EPC cells seeded overnight in 6-well plates at 1 × 10^6^ cells/well were transiently transfected with 2000 ng FLAG, CD44a-tv1-FLAG or CD44a-tv2-FLAG. After 24 h later, these cells were infected with *E. piscicida* at an MOI = 1. At 6 hpi and 24 hpi, the cells were harvest by trypsinisation and centrifuged at 600 × *g* for 5 min. The activities of casp8 were detected using Caspase-8 Activity Assay Kit (cat. C1151, Beyotime) according to the manufacturer’s protocols.

### Immunofluorescence assay

The CD44a^−/−^-14del cells were seeded on coverslips in 24-well plates overnight and then cotransfected with p3×FLAG + GFP, p3×FLAG + p53-GFP, CD44a_tv1-FLAG + p53-GFP, CD44a_tv2-FLAG + p53-GFP. The transfected cells were infected with *E. piscicida* at an MOI of 1. At 6 hpi, the cells were rinsed three times in PBS, and fixed with 4% formaldehyde at 37 °C for 15 min. Then the cells were permeabilized with PBS containing 0.01% Triton® X-100 for 10 min, and incubated with anti-FLAG antibody (1:5000) overnight. After three times wash with PBST, the cells were incubated with ReadyProbes™ Alexa Fluor® 594 Goat Anti-Mouse IgG Antibody (Invitrogen, #R37121) for 1 h. The nuclei were counter-stained with NucBlue^TM^ Fixed Cell ReadyProbes^TM^ (DAPI, Invitrogen, #R37606). Images were taken by a confocal microscope (SP8; Lecia, Wetzlar, Germany).

To determine the possible effect of zebrafish CD44a variants on the localization of p53 in the mitochondria, the CD44a^−/−^-14del cells were seeded on coverslips in 24-well plates overnight and then transfected with FLAG, CD44a_tv1 or CD44a_tv2. After 48 h, the transfected cells were infected with *E. piscicida* for 1 h at an MOI of 10 or left untreated. At 6 hpi, the cells were washed and then treated with 250 nM MitoTracker Deep red (Invitrogen™, M22426) for 30 min at 37 °C. The cells were rinsed three times in PBS, fixed with 4% formaldehyde at 37 °C for 15 min, permeabilized with PBS containing 0.01% Triton^®^ X-100 for 10 min, blocked with PBS containing 5% BSA for 1 h, and incubated with anti-p53 antibody (1: 1000) overnight. After washing, the cells were incubated with Alexa Fluor™ 488 Goat anti-Rabbit antibody for 1 h. The nuclei were counter-stained with DAPI. Images were taken by a confocal microscope.

### Phospho-histone 3 (pH3) immunostaining

To investigate the effect of CD44a deficiency on the cell proliferation in vivo, the WT and CD44a^−/−^-14del zebrafish embryos collected at 2 dpf were fixed with 4% paraformaldehyde overnight. To investigate the effect of p53 on the zebrafish CD44a -mediated cell proliferation, the p3×FLAG or p53-FLAG were microinjected into one- or two-cell stage embryos from the WT or CD44a^−/−^-14del zebrafish. Zebrafish embryos were collected at 2 dpf and fixed with 4% paraformaldehyde overnight. Embryos were blocked for 30 min using Blocking Reagent, and then incubated overnight at 4 °C with a polyclonal anti-phospho-Histone H3 antibody (1: 500). After the washing for four times, these embryos were incubated with the fluorescence-conjugated Alexa Fluor™ 488 Goat anti-Mouse IgG second antibody overnight at 4 °C. After the removal of excess antibody by similar washing, these embryos were mounted for imaging using confocal microscopy.

### Statistics and reproducibility

All data are representative of at least three independent experiments and are presented as means ± SD. Differences in larvae survival were assessed using the log-rank test in the GraphPad Prism. Two-group comparisons were made using Student’s *t* test. The *p* values less than 0.05 were considered to indicate statistical significance (*p < 0.05, **p < 0.01).

### Reporting summary

Further information on research design is available in the Nature Research Reporting Summary linked to this article.

## Supplementary information

Supplementary Information

Description of Additional Supplementary Files

Supplementary Data 1

Supplementary Data 2

Reporting Summary

## Data Availability

The raw sequences of Illumina deep sequencing were deposited at NCBI Gene Expression Omnibus (GEO) database under the accession number GSE180770. The source data behind the graphs can be found in Supplementary Data 1. The unedited and uncropped blots were included in Supplementary Data 2. All other data are available from the corresponding author on reasonable request.

## References

[1] Goodison S, Urquidi V, Tarin D (1999). CD44 cell adhesion molecules. Mol. Pathol..

[2] Suenaga N, Mori H, Itoh Y, Seiki M (2005). CD44 binding through the hemopexin-like domain is critical for its shedding by membrane-type 1 matrix metalloproteinase. Oncogene.

[3] Mythreye K, Blobe GC (2009). Proteoglycan signaling co-receptors: roles in cell adhesion, migration and invasion. Cell. Signal..

[4] Orian-Rousseau V, Sleeman J (2014). CD44 is a multidomain signaling platform that integrates extracellular matrix cues with growth factor and cytokine signals. Adv. Cancer Res..

[5] Thorne RF, Legg JW, Isacke CM (2004). The role of the CD44 transmembrane and cytoplasmic domains in co-ordinating adhesive and signalling events. J. Cell Sci..

[6] Dzwonek J, Wilczynski GM (2015). CD44: molecular interactions, signaling and functions in the nervous system. Front. Cell Neurosci..

[7] Jalkanen S (1990). Lymphocyte migration into the skin: the role of lymphocyte homing receptor (CD44) and endothelial cell antigen (HECA-452). J. Invest. Dermatol..

[8] Naor D, Sionov RV, Ish-Shalom D (1997). CD44: structure, function, and association with the malignant process. Adv. Cancer Res..

[9] Johnson P, Ruffell B (2009). CD44 and its role in inflammation and inflammatory diseases. Inflamm. Allergy Drug Targets.

[10] Orian-Rousseau V (2015). CD44 acts as a signaling platform controlling tumor progression and metastasis. Front. Immunol..

[11] Screaton GR (1992). Genomic structure of DNA encoding the lymphocyte homing receptor CD44 reveals at least 12 alternatively spliced exons. Proc. Natl Acad. Sci. USA.

[12] Fox SB (1994). Normal human tissues, in addition to some tumors, express multiple different CD44 isoforms. Cancer Res..

[13] Liu J, Jiang G (2006). CD44 and hematologic malignancies. Cell. Mol. Immunol..

[14] Puré E, Cuff CA (2001). A crucial role for CD44 in inflammation. Trends Mol. Med..

[15] Rudy W (1993). The two major CD44 proteins expressed on a metastatic rat tumor cell line are derived from different splice variants: each one individually suffices to confer metastatic behavior. Cancer Res..

[16] Chen C, Zhao S, Karnad A, Freeman JW (2018). The biology and role of CD44 in cancer progression: therapeutic implications. J. Hematol. Oncol..

[17] Forster-Horváth C (2001). Constitutive intracellular expression and activation-induced cell surface up-regulation of CD44v3 in human T lymphocytes. Eur. J. Immunol..

[18] Seiter S, Schmidt DS, Zöller M (2000). The CD44 variant isoforms CD44v6 and CD44v7 are expressed by distinct leukocyte subpopulations and exert non-overlapping functional activities. Int. Immunol..

[19] Cao L, Wu XM, Nie P, Chang MX (2019). The negative regulation of piscine CD44c in viral and bacterial infection. Dev. Comp. Immunol..

[20] Hu YW (2017). NOD1 deficiency impairs CD44a/Lck as well as PI3K/Akt pathway. Sci. Rep..

[21] Wu XM (2018). RIP2 is a critical regulator for NLRs signaling and MHC antigen presentation but not for MAPK and PI3K/Akt pathways. Front. Immunol..

[22] Kimura N (2017). Expression of autophagy-associated genes in skeletal muscle: an experimental model of chloroquine-induced myopathy. Pathobiology.

[23] Choi M (2022). Effect of hydroxychloroquine and chloroquine on syncytial differentiation and autophagy in primary human trophoblasts. Biomed. Pharmacother..

[24] Strom E (2006). Small-molecule inhibitor of p53 binding to mitochondria protects mice from gamma radiation. Nat. Chem. Biol..

[25] Cao L, Yan D, Xiao J, Feng H, Chang MX (2021). The zebrafish antiapoptotic protein BIRC2 promotes *Edwardsiella piscicid*a infection by inhibiting caspases and accumulating p53 in a p53 transcription-dependent and -independent manner. Front. Immunol..

[26] Günthert U (1995). Are CD44 variant isoforms involved in human tumour progression?. Cancer Surv..

[27] Prochazka L, Tesarik R, Turanek J (2014). Regulation of alternative splicing of CD44 in cancer. Cell. Signal..

[28] Chaudhry GE (2021). Understanding hyaluronan receptor (CD44) interaction, HA-CD44 activated potential targets in cancer therapeutics. Adv. Pharm. Bull..

[29] Jordan AR, Racine RR, Hennig MJ, Lokeshwar VB (2015). The role of CD44 in disease pathophysiology and targeted treatment. Front. Immunol..

[30] Eriksson E (2003). CD44-regulated intracellular proliferation of Listeria monocytogenes. Infect. Immun..

[31] Rouschop KM (2006). Urothelial CD44 facilitates *Escherichia coli* infection of the murine urinary tract. J. Immunol..

[32] van der Windt GJ (2010). CD44 deficiency is associated with increased bacterial clearance but enhanced lung inflammation during Gram-negative pneumonia. Am. J. Pathol..

[33] van der Windt GJ (2011). The role of CD44 in the acute and resolution phase of the host response during *pneumococcal pneumonia*. Lab. Invest..

[34] Katoh S, Zheng Z, Oritani K, Shimozato T, Kincade PW (1995). Glycosylation of CD44 negatively regulates its recognition of hyaluronan. J. Exp. Med..

[35] Guvench O (2015). Revealing the mechanisms of protein disorder and N-glycosylation in CD44-hyaluronan binding using molecular simulation. Front. Immunol..

[36] Dolasia K, Bisht MK, Pradhan G, Udgata A, Mukhopadhyay S (2018). TLRs/NLRs: shaping the landscape of host immunity. Int. Rev. Immunol..

[37] Rehwinkel J, Gack MU (2020). RIG-I-like receptors: their regulation and roles in RNA sensing. Nat. Rev. Immunol..

[38] Kawana H (2008). CD44 suppresses TLR-mediated inflammation. J. Immunol..

[39] Liang J (2007). CD44 is a negative regulator of acute pulmonary inflammation and lipopolysaccharide-TLR signaling in mouse macrophages. J. Immunol..

[40] Heldin P, Kolliopoulos C, Lin CY, Heldin CH (2020). Involvement of hyaluronan and CD44 in cancer and viral infections. Cell Signal..

[41] De Falco V (2012). CD44 proteolysis increases CREB phosphorylation and sustains proliferation of thyroid cancer cells. Cancer Res..

[42] Ibrahim HM (2019). Prognostic value of cyclin D1 and CD44 expression in gastric adenocarcinoma. J. Gastrointest. Cancer.

[43] Kruiswijk F, Labuschagne CF, Vousden KH (2015). p53 in survival, death and metabolic health: a lifeguard with a licence to kill. Nat. Rev. Mol. Cell Biol..

[44] Godar S (2008). Growth-inhibitory and tumor- suppressive functions of p53 depend on its repression of CD44 expression. Cell.

[45] Föger N, Marhaba R, Zöller M (2000). CD44 supports T cell proliferation and apoptosis by apposition of protein kinases. Eur. J. Immunol..

[46] Rajasagi M, Vitacolonna M, Benjak B, Marhaba R, Zöller M (2009). CD44 promotes progenitor homing into the thymus and T cell maturation. J. Leukoc. Biol..

[47] Häcker G (2018). Apoptosis in infection. Microbes Infect..

[48] Naderer T, Fulcher MC (2018). Targeting apoptosis pathways in infections. J. Leukoc. Biol..

[49] Shi Y (2002). Mechanisms of caspase activation and inhibition during apoptosis. Mol. Cell..

[50] Chen J (2016). The Cell-Cycle Arrest and Apoptotic Functions of p53 in Tumor Initiation and Progression. Cold Spring Harb. Perspect. Med..

[51] Boya P (2005). Inhibition of macroautophagy triggers apoptosis. Mol. Cell. Biol..

[52] Marchiando AM (2013). A deficiency in the autophagy gene Atg16L1 enhances resistance to enteric bacterial infection. Cell Host Microbe.

[53] Lu Q (2016). Homeostatic control of innate lung inflammation by Vici syndrome gene Epg5 and additional autophagy genes promotes influenza pathogenesis. Cell Host Microbe.

[54] Maiuri MC (2010). Autophagy regulation by p53. Curr. Opin. Cell Biol..

[55] Green DR, Kroemer G (2009). Cytoplasmic functions of the tumour suppressor p53. Nature.

[56] Eby KG (2010). ISG20L1 is a p53 family target gene that modulates genotoxic stress-induced autophagy. Mol. Cancer.

[57] White E (2016). Autophagy and p53. Cold Spring Harb. Perspect. Med..

[58] Maquarre E (2005). CD44 ligation induces apoptosis via caspase- and serine protease-dependent pathways in acute promyelocytic leukemia cells. Leukemia.

[59] Qian H, Xia L, Ling P, Waxman S, Jing Y (2012). CD44 ligation with A3D8 antibody induces apoptosis in acute myeloid leukemia cells through binding to CD44s and clustering lipid rafts. Cancer Biol. Ther..

[60] Muys BR (2021). The p53-induced RNA-binding protein ZMAT3 is a splicing regulator that inhibits the splicing of oncogenic CD44 variants in colorectal carcinoma. Genes Dev..

[61] Dhar D (2018). Liver cancer initiation requires p53 inhibition by CD44-enhanced growth factor signaling. Cancer Cell.

[62] Mandal CC, Ghosh-Choudhury N, Yoneda T, Choudhury GG, Ghosh-Choudhury N (2011). Simvastatin prevents skeletal metastasis of breast cancer by an antagonistic interplay between p53 and CD44. J. Biol. Chem..

[63] Cao L (2018). The discrepancy function of NLRC5 isoforms in antiviral and antibacterial immune responses. Dev. Comp. Immunol..

