# Supplementary material for: CD44a functions as a regulator of p53 signaling, apoptosis and autophagy in the antibacterial immune response
Source: Commun Biol. 2022 Aug 30;5:889. doi: 10.1038/s42003-022-03856-1 (PMC9427754; doi:10.1038/s42003-022-03856-1)
Supplement: Supplementary file 1 — Supplementary Information [file 42003_2022_3856_MOESM1_ESM.pdf]

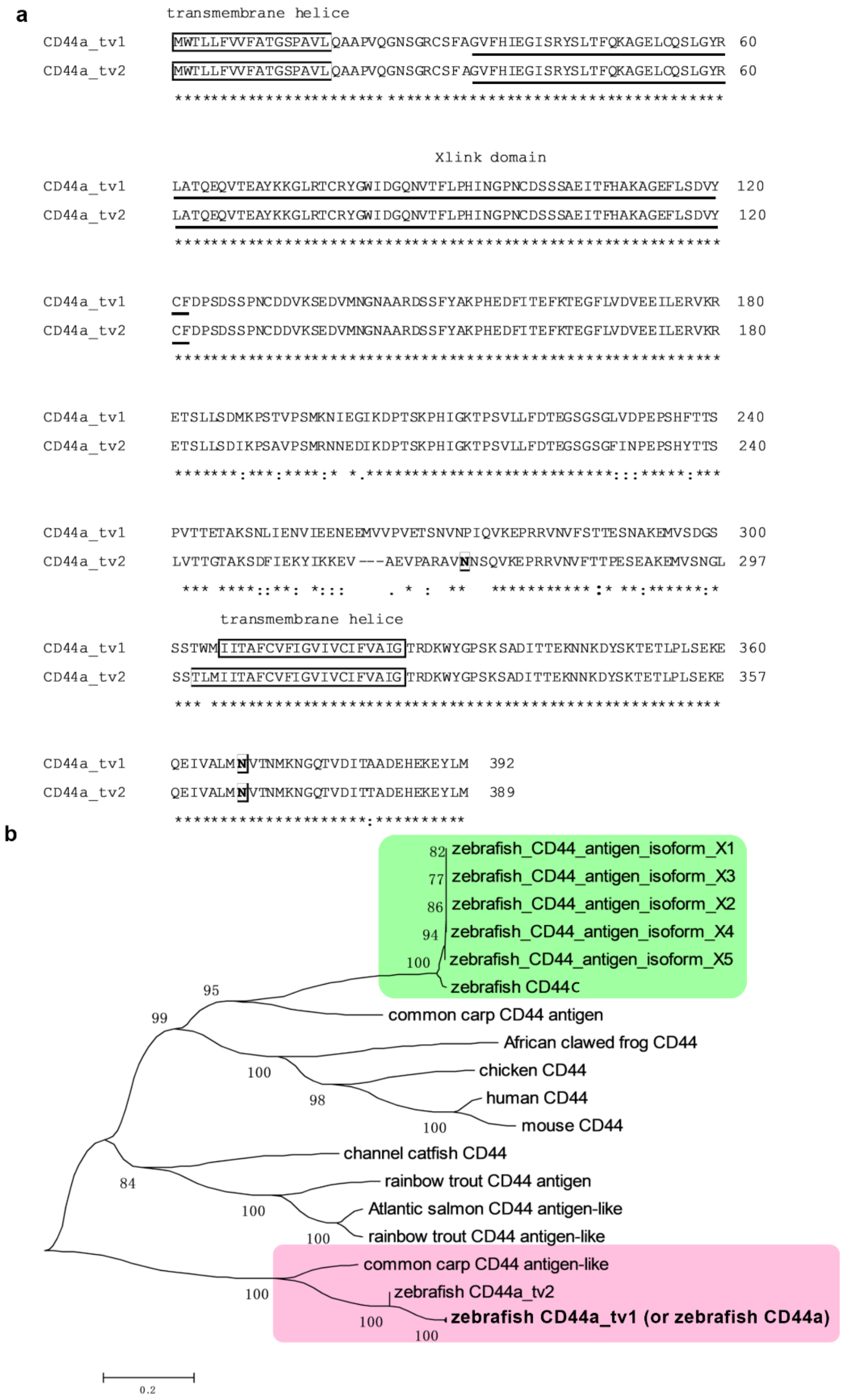

Supplementary Fig. 1 Bioinformatics analysis of zebrafish CD44a variants. a The sequence alignments of zebrafish CD44a_tv1 and CD44a_tv2. The N-glycosylated sites and transmembrane helices are boxed. The Xlink domain is underlined. **b** Phylogenetic tree analysis of vertebrate CD44. The CD44a_tv1 corresponds to the CD44a previously reported, which is highlighted in bold.

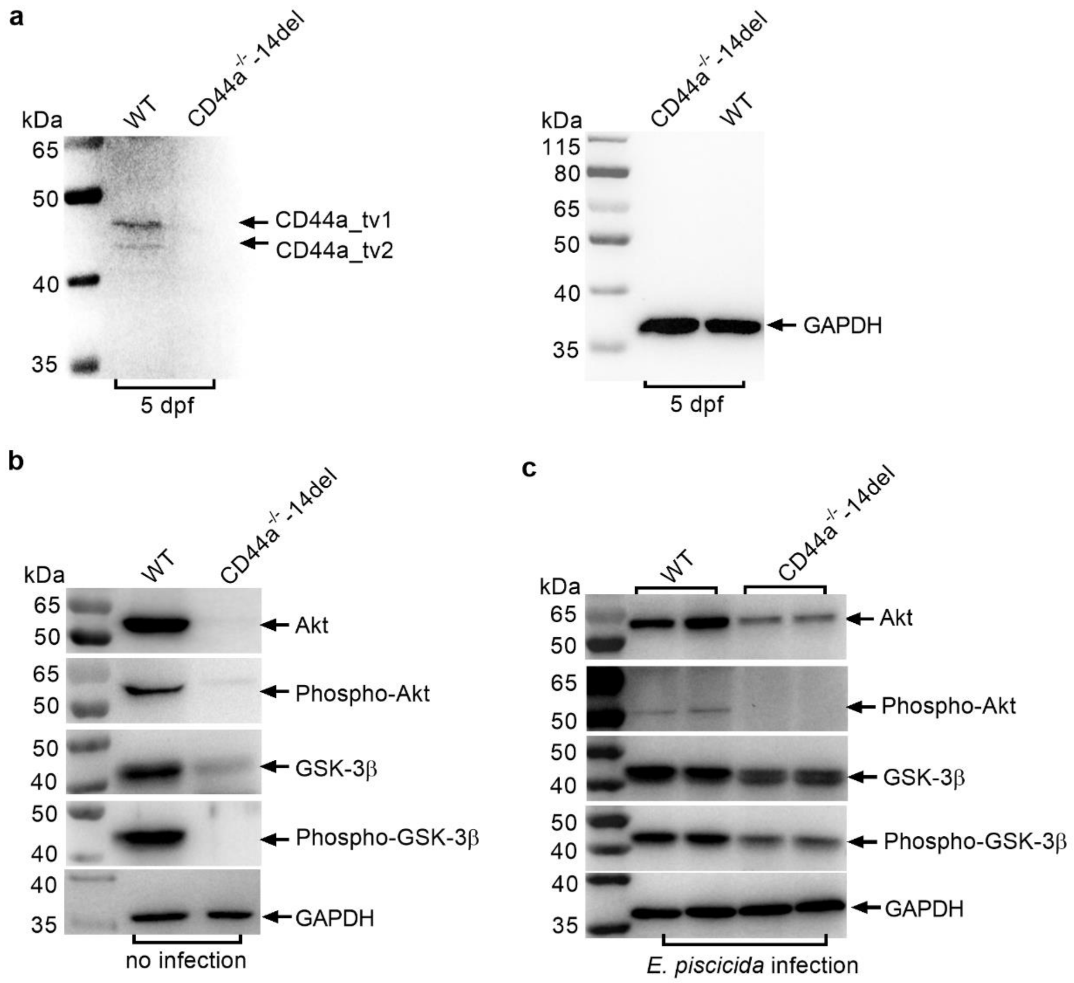

Supplementary Fig. 2 The effect of CD44a deficiency on the protein expressions of CD44a and Akt/GSK-3β axis. a The protein expression of CD44a variants in the WT or CD44a$^{-/-}$-14del zebrafish larvae collected at 5 dpf. **b** Immunoblot analysis of Akt, GSK-3β, phospho-Akt and phospho-GSK-3β in larvae homogenate from the WT or CD44a$^{-/-}$-14del zebrafish without the infection of *E. piscicida.* The larvae were collected at 5 dpf. **c** Immunoblot analysis of Akt, GSK-3β, phospho-Akt and phospho-GSK-3β in larvae homogenate from the WT or CD44a$^{-/-}$-14del zebrafish infected with *E. piscicida.* The larvae were collected at 24 hpi.

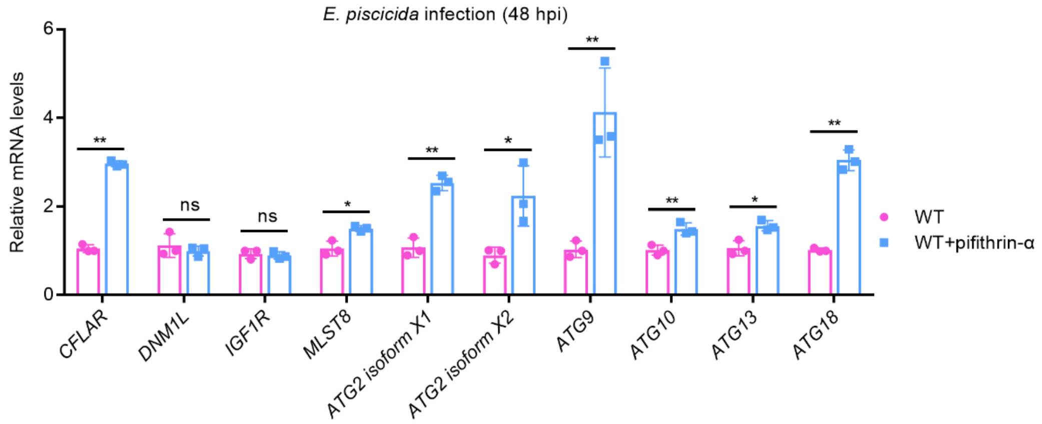

Supplementary Fig. 3 mRNA levels of autophagy-related genes in the WT zebrafish without or with the treatment of pifithrin-α. The larvae were collected at 48 hpi. Data are presented as mean values ± SD (n = 3). *$p < 0.05$, **$p < 0.01$; ns, not significant.

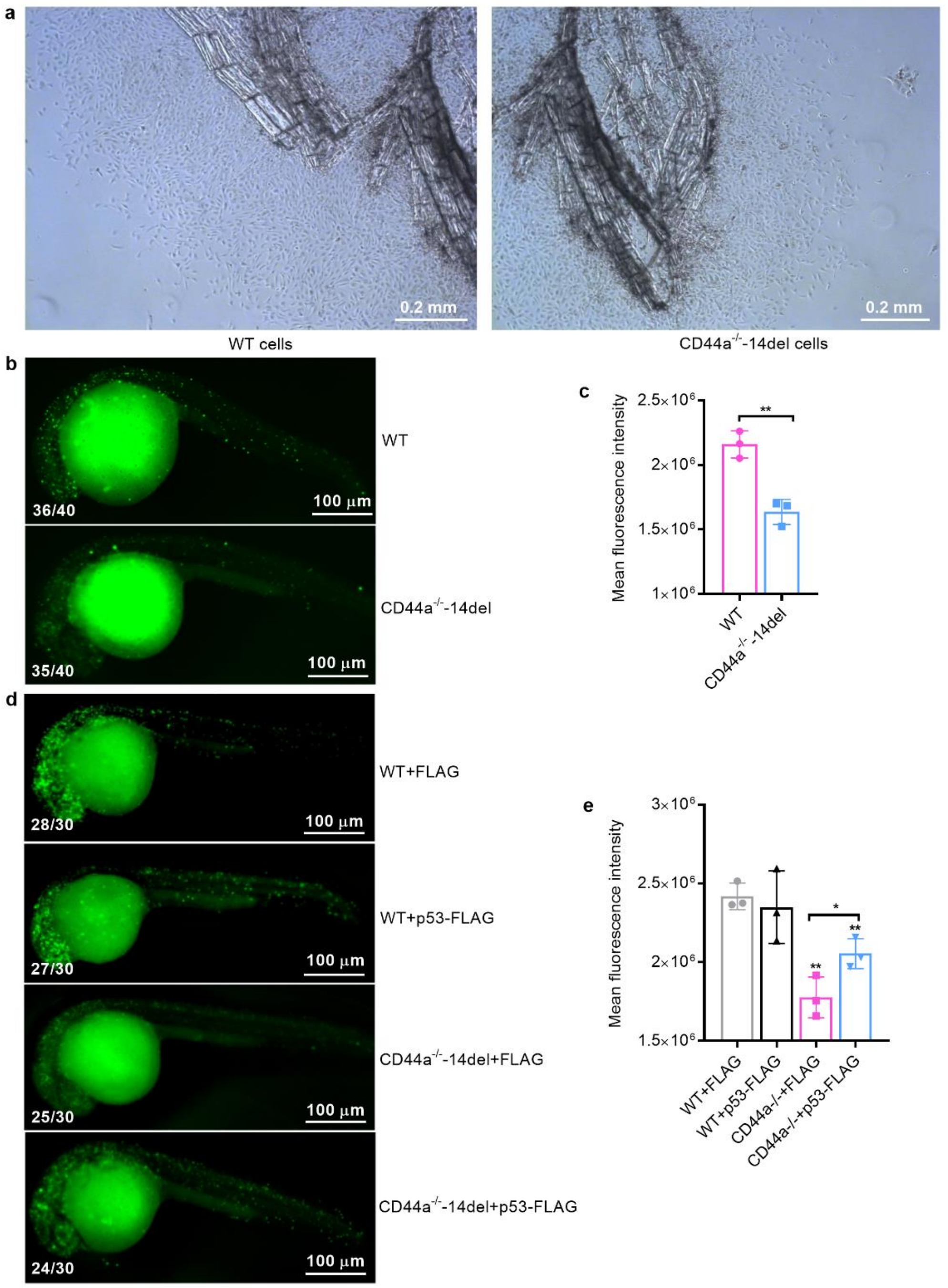

Supplementary Fig. 4 The cell lines and zebrafish of WT and CD44a deficiency. a The cellular morphology of primary cell cultures developed from caudal fins of the WT and CD44a$^{-/-}$-14del zebrafish at the age of 1 month. Scale bar, 0.2 mm. **b** Phospho-histone 3 (pH3) immunostaining of the WT and CD44a$^{-/-}$-14del zebrafish embryos collected at 2 dpf. Scale bar, 100 μm. **c** The average fluorescence intensity of pH3 immunostaining of the WT and CD44a$^{-/-}$-14del zebrafish embryos collected at 2 dpf. **d** Phospho-histone 3 (pH3) immunostaining of the WT and CD44a$^{-/-}$-14del zebrafish embryos microinjected with the FLAG or p53-FLAG, which were collected at 2 dpf. Scale bar, 100 μm. **e** The average fluorescence intensity of pH3 immunostaining of the WT and CD44a$^{-/-}$-14del zebrafish embryos microinjected with the FLAG or p53-FLAG, which were collected at 2 dpf. For **c** and **e**, data are presented as mean values ± SD (n = 3). *$p < 0.05$, **$p < 0.01$.

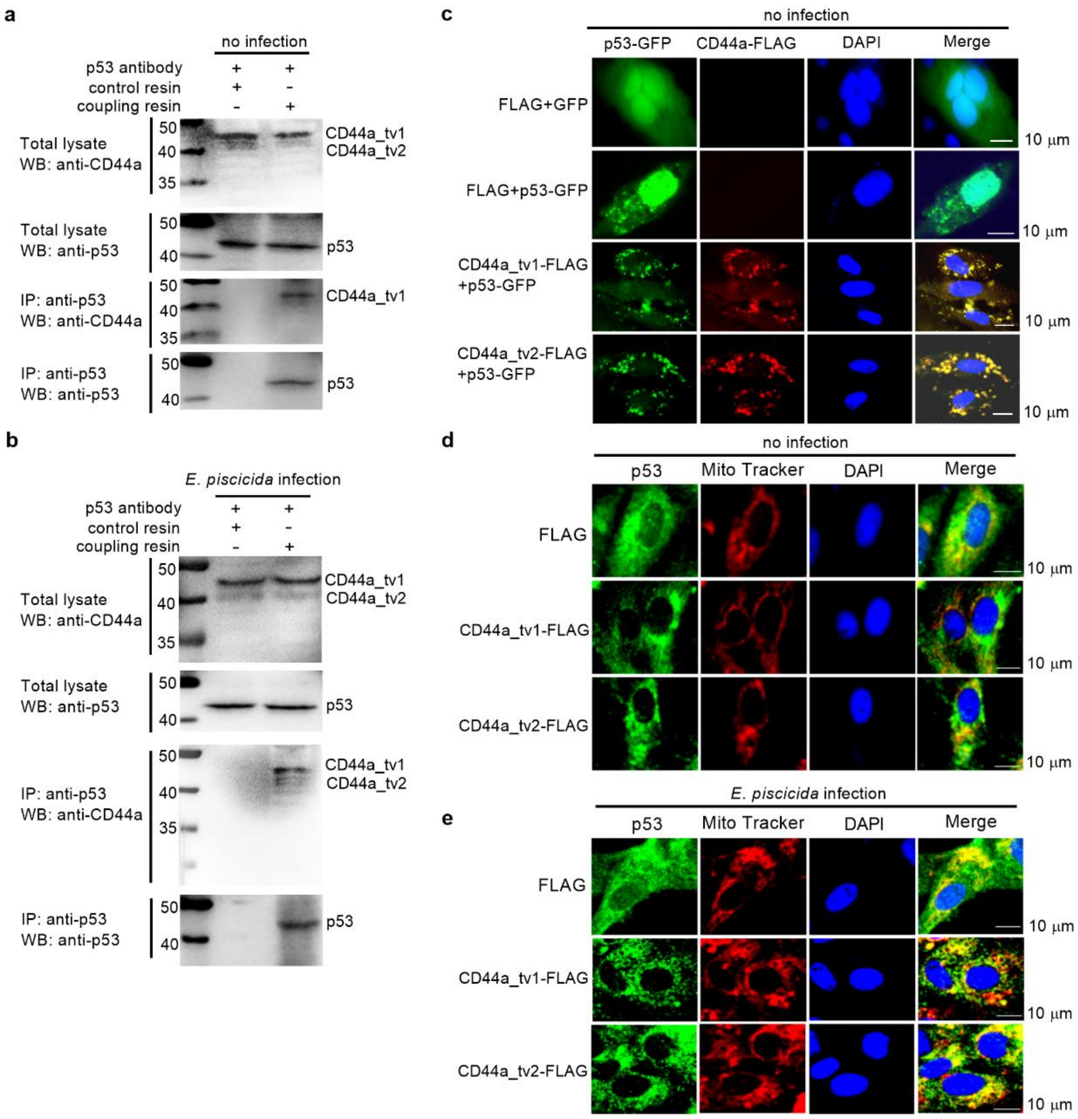

Supplementary Fig. 5 The interaction between zebrafish CD44a variants and p53. a The endogenous interaction between CD44a and p53 in zebrafish larvae without the infection of *E. piscicida.* **b** The endogenous interaction between CD44a and p53 in zebrafish larvae with the infection of *E. piscicida.* **c** The colocalization between zebrafish CD44a variants and p53 in the CD44a$^{-/-}$-14del cells in the absence of infection. Scale bar, 10 µm. **d** The co-staining between the endogenous p53 protein and mitochondria in the CD44a$^{-/-}$-14del cells transfected with FLAG, CD44a_tv1 or CD44a_tv2 without the infection of *E. piscicida.* Scale bar, 10 µm. **e** The co-staining between the endogenous p53 protein and mitochondria in the CD44a$^{-/-}$-14del cells transfected with FLAG, CD44a_tv1 or CD44a_tv2 with the infection of *E. piscicida.* Scale bar, 10 µm.

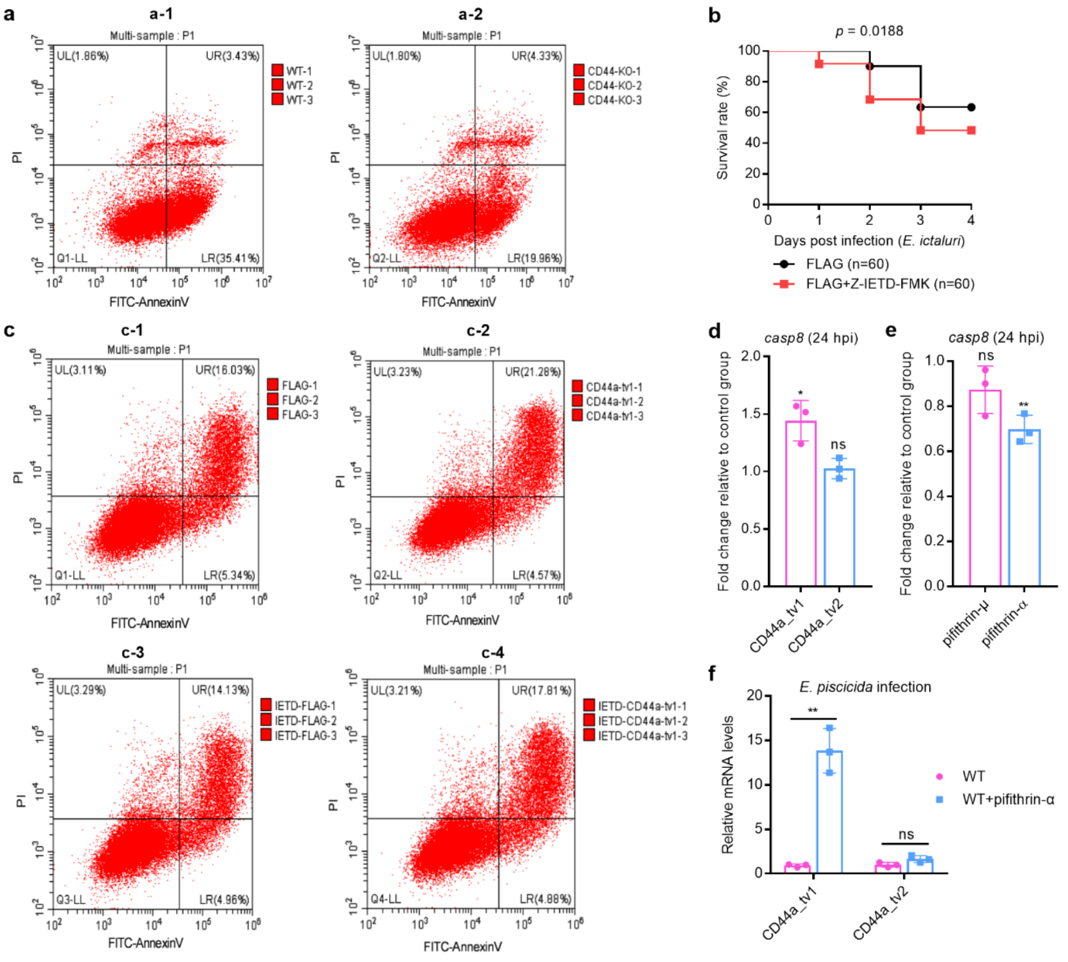

Supplementary Fig. 6 The effect of zebrafish CD44a variants in apoptosis. a dot plot of PI vs annexin V staining for the WT and CD44a$^{-/-}$-14del zebrafish cells. a-1: WT zebrafish cells; a-2: CD44a$^{-/-}$-14del zebrafish cells. UL: Dead cells; LL: live cells; UR: late apoptosis; LR: early apoptosis. **b** Larval survival analysis in the WT zebrafish larvae without or with the treatment of Z-IETD-FMK (n = 60 for each group). **c** dot plot of PI vs annexin V staining for EPC cells transfected with FLAG or CD44a_tv1 with or without the treatment of Z-IETD-FMK. c-1: EPC cells transfected with FLAG without the treatment of Z-IETD-FMK; c-2: EPC cells transfected with CD44a_tv1 without the treatment of Z-IETD-FMK; c-3: EPC cells transfected with FLAG with the treatment of Z-IETD-FMK; c-4: EPC cells transfected with CD44a_tv1 with the treatment of Z-IETD-FMK. UL: Dead cells; LL: live cells; UR: late apoptosis; LR: early apoptosis. **d** The effects of zebrafish CD44a variants on the transcription of *casp8* in the WT larvae infected with *E. piscicida*. **e** The effects of p53 inactivators pifithrin-μ and pifithrin-α on the transcription of *casp8* in the WT larvae infected with *E. piscicida*. **f** The effect of pifithrin-α on the transcription of zebrafish CD44a_tv1 and CD44a_tv2 in the WT larvae infected with *E. piscicida*. For **d-f**, data are presented as mean values ± SD (n = 3). *$p < 0.05$, **$p < 0.01$; ns, not significant.

Supplemental Table 1. Primer information

Name	Sequence	Application
CD44a-F	GTCAAGCTTACCATGTGGACTTTGTTATTTGTAG	Recombinant plasmids for CD44a_tv1_FLAG, CD44a_tv2_FLAG, CD44a_tv1-GFP and CD44a_tv2-GFP
CD44a-R	GAAGGTACCATTAAATATTCTTTTTCGTGTTCATC	
p53-F	CCAAGCTTATGGCGCAAAACGACAG	Recombinant plasmids for p53-FLAG and p53-GFP
p53-R	CCGGTACCGAATCAGAGTCGCTTCT	
PGL3-P53-F	CCCGGTACCATAAATGACTGCTGACACCTATTCT	Recombinant plasmids for p53-pGL3
PGL3-P53-R	CCCTCGAGTCTTGGCTGTCGTTTTGCGCCATT	
CD44aF	GAAAGTAATGCGAAGGAG	Quantitative real-time PCR
CD44aR	TCATCAGTGCCACAATCT	
p53F	CTCAGGTTCCCGCAGTC	
p53R	TCCATTCAGCACCAAGC	
casp8F	GTGTCTGTTGACGAAATACGA	
casp8R	GTGACTGAATAAACCAGGAGC	
ccnb1F	TATCTGGCAGGAAAGGA	
ccnb1R	AGAGGGAGGGTAGTGGA	
ccnb3F	TATTTGCGACGATGCCTAC	
ccnb3R	TCCTGATGACAGCGAGTTTA	
cdk1F	AACTCGCCACCAAGAAACC	
cdk1R	TTGTCGTGCCGAAATCCTC	
cdk2F	CGAGGTTGTAACTTTGTGGT	
cdk2R	AAGGAGGGTTTGTAGTCTGG	
ccne1F	TACCCACAGGCTACATT	
ccne1R	TCATCTGCTGCTATTCC	
ccne2F	GAAGGTGATGTTAGTGCCAGTG	
ccne2R	TTTGAGGCTATGAAGAGTGAGG	
chk1F	ACACCGCTGGTTTAGCAGAAGTT	
chk1R	GACCCAAAGCAATACAAGAATCACG	
chk2F	GAGTCTGGGAGTCCTACTGTTCAT	
chk2R	TTGCGTTTCCTTGTTGCTTC	
rrm2F	TGCCTGGACTCACCTTCT	
rrm2R	GTCGGTCAGCCACAAACT	
baxF	CTCATCAGCACCGTTCAGT	
baxR	TAGCGAGTTCTTCTCCAGTAA	
birc5F	CGGAGGATGACCCTGAGAA	
birc5R	AAGGACCACAGCCAAATGC	
birc6F	CACAGTGTCCGCTTCAACCC	
birc6R	CGCTCGTAACCTGGCTCATT	
egfrF	GCATACGCACTGGAAACAAC	
egfrR	TGGGTCACAAGCCAAACAC	

pdcd8F	TAAAGCCACAGCCAAGGACAC	
pdcd8R	CACAGAACGATACCAACCACCA	
htra2F	AGGGATTTCCTTCGCTATTC	
htra2R	TAACATCTCCTGGCTTCATT	
ptpn13F	GGGAGGAAAGGTCAAGTGT	
ptpn13R	GCAGTGAGTGATGATGGGT	
casp22F	TTCTACGGATAAGACCAACACCAT	
casp22R	AAAGCCCAGCCATTCAAACAA	
cflarF	GATGGACACGGAGGACC	
cflarR	CTGCTTCAGTGGCGATG	
dnm1LF	GATTCAATAGTGGAGGTGGTC	
dnm1LR	ACGGCATCTTCTCATTCTTC	
IGF1RF	CGTCTGGTCGTTTGGTGT	
IGF1RR	GCTTGTTCTCCTCGCTGT	
ATG2 isoform X1F	TCGTTTGGCACCTCCACAGC	
ATG2 isoform X1R	TGATTTGGTTCCTCATTCCTCCC	
ATG2 isoform X2F	GTAAGAGTGGCAGCAGACGG	
ATG2 isoform X2R	GGAGATTGAAGCAGGAGGGA	
ATG9F	GTCTGAGGCTCCGCTCTGCT	
ATG9R	TGTCCGTCGTGTTGCTGTCG	
ATG10F	ACTGAACTTCCGACAGACGA	
ATG10R	GACGACAGGACCCACAACAC	
ATG13F	CCTCTGGGAACTGTGGATTT	
ATG13R	TTACCTGAGTGGTGGCTTTG	
ATG18F	TTCGCCACTGTTCACCTCA	
ATG18R	AACTCCTGCTCATCATCCA	
MLST8F	TCAGCCCAGACTCCACATT	
MLST8R	ACACCACAGACGAGCAAGG	
TBC1D15F	TCGGGCTCCTGAGTTAGA	
TBC1D15R	CCCATTCCTCCATTGTTA	
TFE3bF	GCCTCAGAGCCACCTCAACAT	
TFE3bR	GGGAGAAGAGCACATCCGAAC	
PGRP6F	GTGGTGCTGACTTTGGAT	
PGRP6R	GTTGCTCTGCTGGTGGTA	
NOD2F	AGTTTCTGGGATTATGGGGT	
NOD2R	ACTGCCCACACCATTATCCA	
