# Supplementary material for: CD44a functions as a regulator of p53 signaling, apoptosis and autophagy in the antibacterial immune response
Source: Commun Biol. 2022 Aug 30;5:889. doi: 10.1038/s42003-022-03856-1 (PMC9427754; doi:10.1038/s42003-022-03856-1)
Supplement: Supplementary file 2 — Description of Additional Supplementary Files [file 42003_2022_3856_MOESM2_ESM.pdf]

Description of Additional Supplementary Files

File name: Supplementary Data 1
Description: The source data behind the graphs in the paper.

File name: Supplementary Data 2
Description: The unedited and uncropped blots.
