# Supplementary figures and images for: CD44a functions as a regulator of p53 signaling, apoptosis and autophagy in the antibacterial immune response (part 1 of 2)
Source: Commun Biol. 2022 Aug 30;5:889. doi: 10.1038/s42003-022-03856-1 (PMC9427754; doi:10.1038/s42003-022-03856-1)

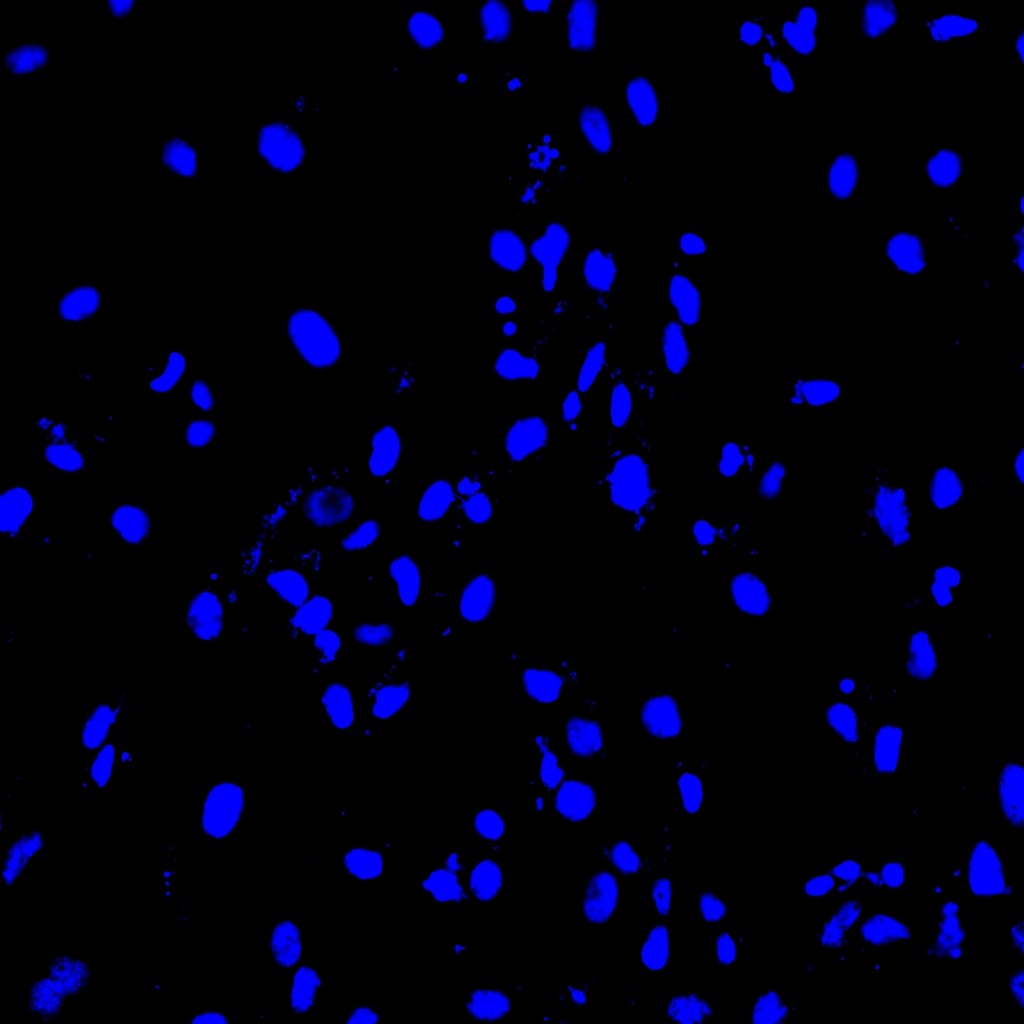

Supplement: Supplementary file 3 — Supplementary Data 1 [file 42003_2022_3856_MOESM3_ESM.zip › Figure 6/Fig. 6h/FLAG+GFP-P53/DAPI.jpg]

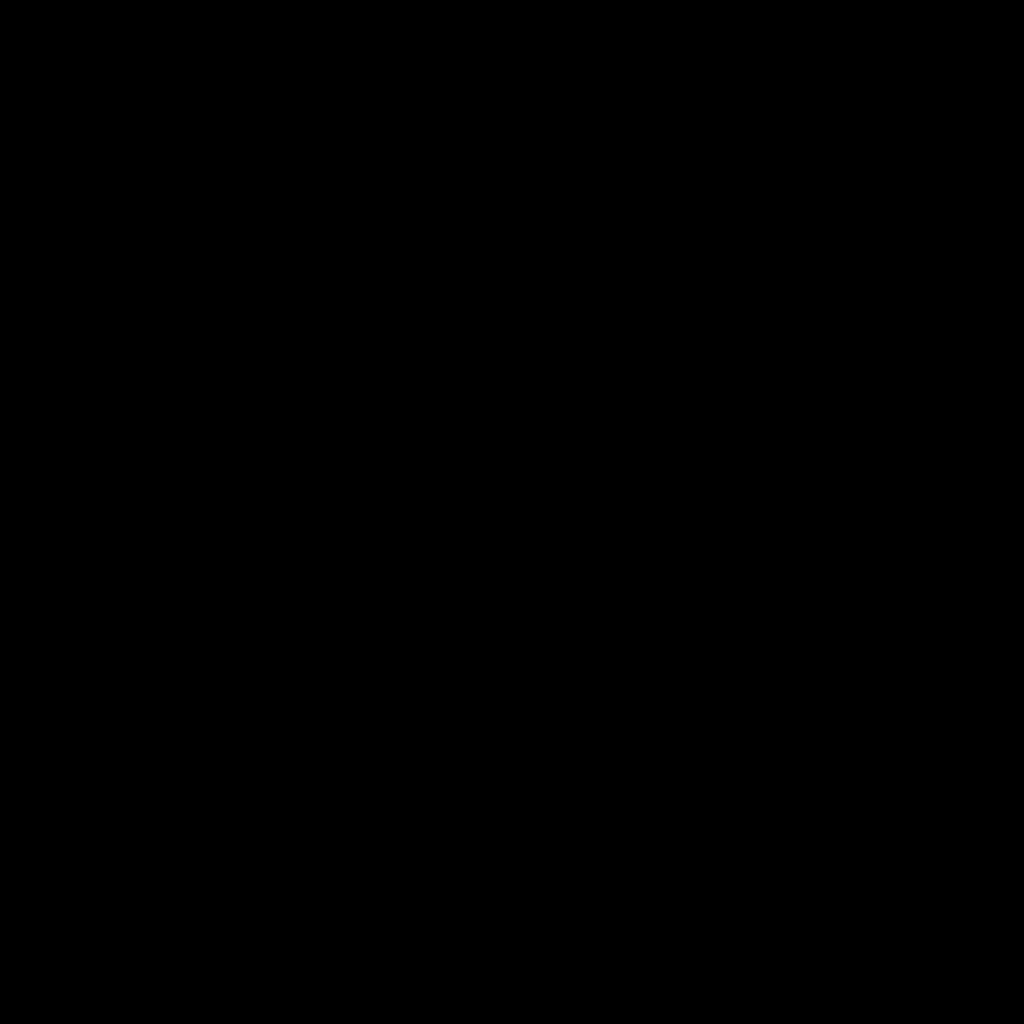

Supplement: Supplementary file 3 — Supplementary Data 1 [file 42003_2022_3856_MOESM3_ESM.zip › Figure 6/Fig. 6h/FLAG+GFP-P53/FLAG.jpg]

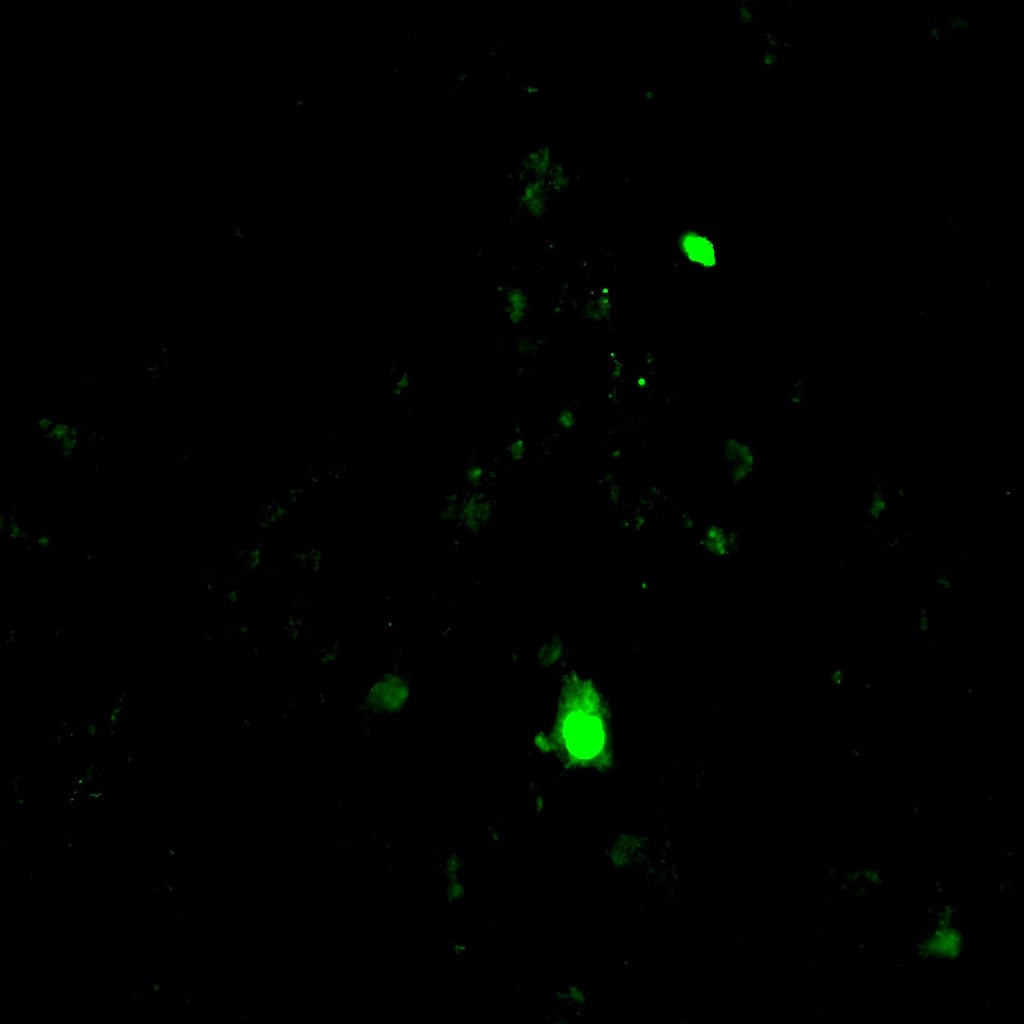

Supplement: Supplementary file 3 — Supplementary Data 1 [file 42003_2022_3856_MOESM3_ESM.zip › Figure 6/Fig. 6h/FLAG+GFP-P53/GFP-P53.jpg]

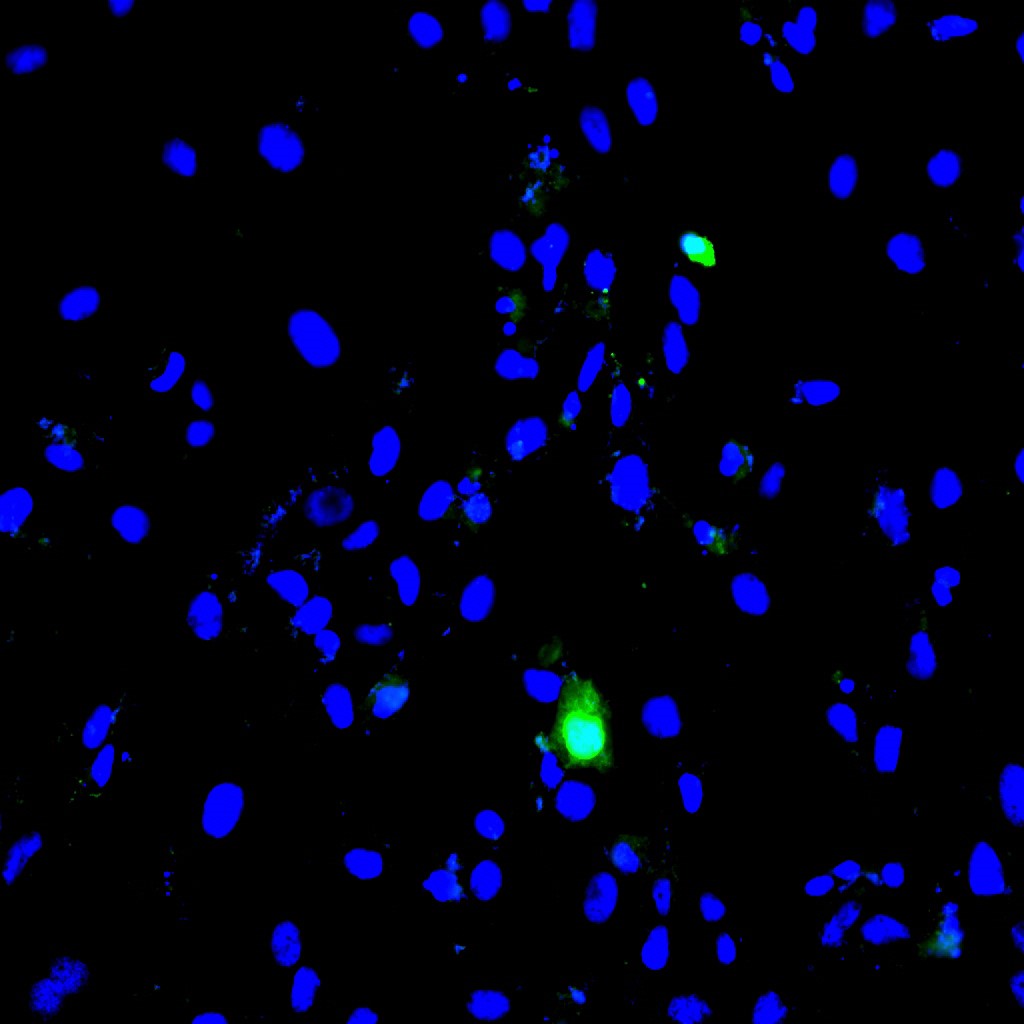

Supplement: Supplementary file 3 — Supplementary Data 1 [file 42003_2022_3856_MOESM3_ESM.zip › Figure 6/Fig. 6h/FLAG+GFP-P53/Merge.jpg]

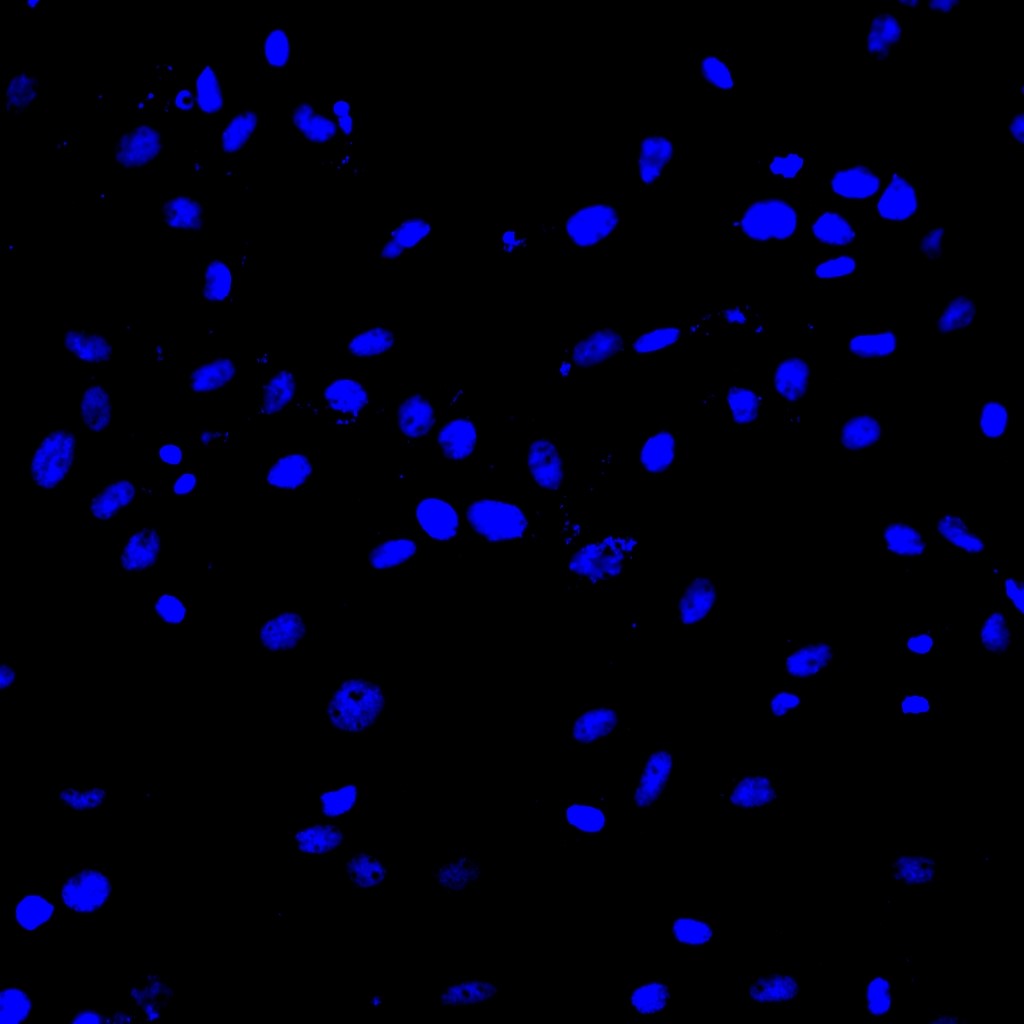

Supplement: Supplementary file 3 — Supplementary Data 1 [file 42003_2022_3856_MOESM3_ESM.zip › Figure 6/Fig. 6h/FLAG+GFP/DAPI.jpg]

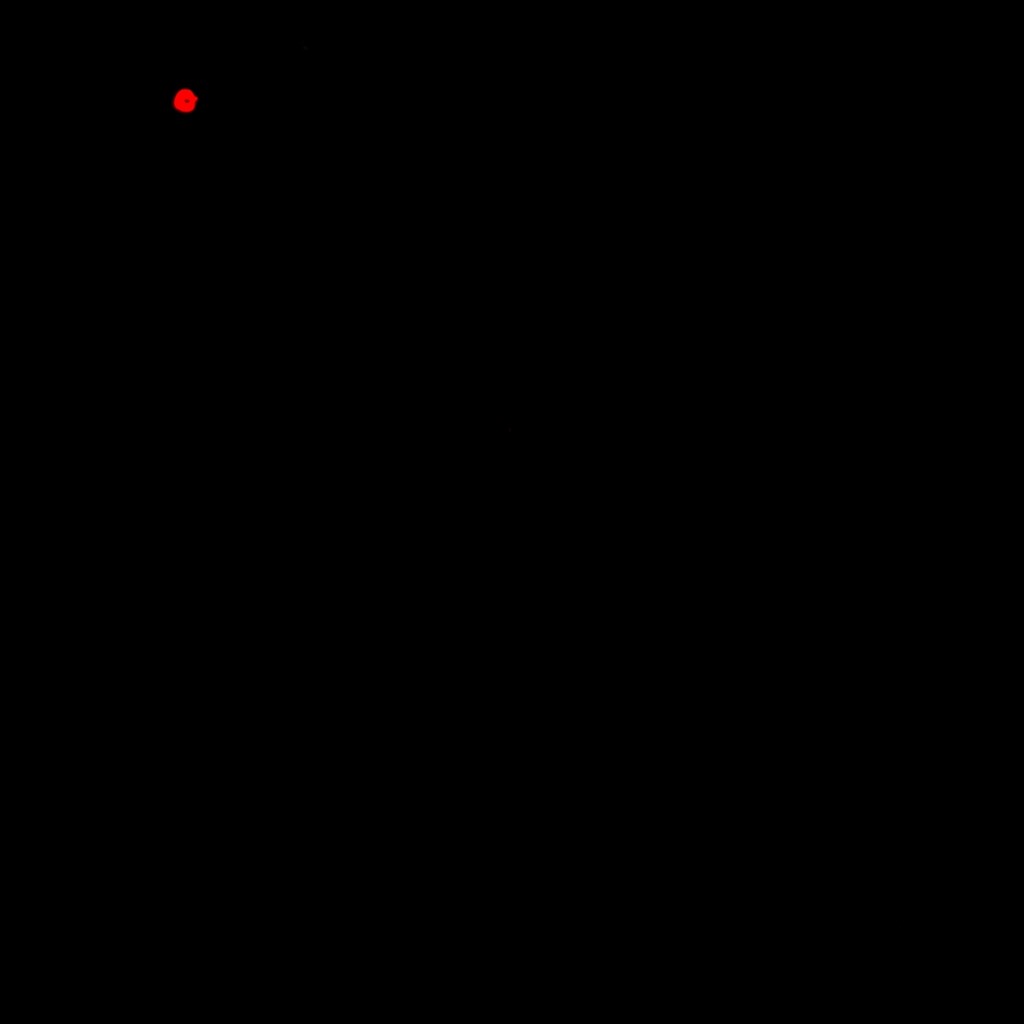

Supplement: Supplementary file 3 — Supplementary Data 1 [file 42003_2022_3856_MOESM3_ESM.zip › Figure 6/Fig. 6h/FLAG+GFP/flag.jpg]

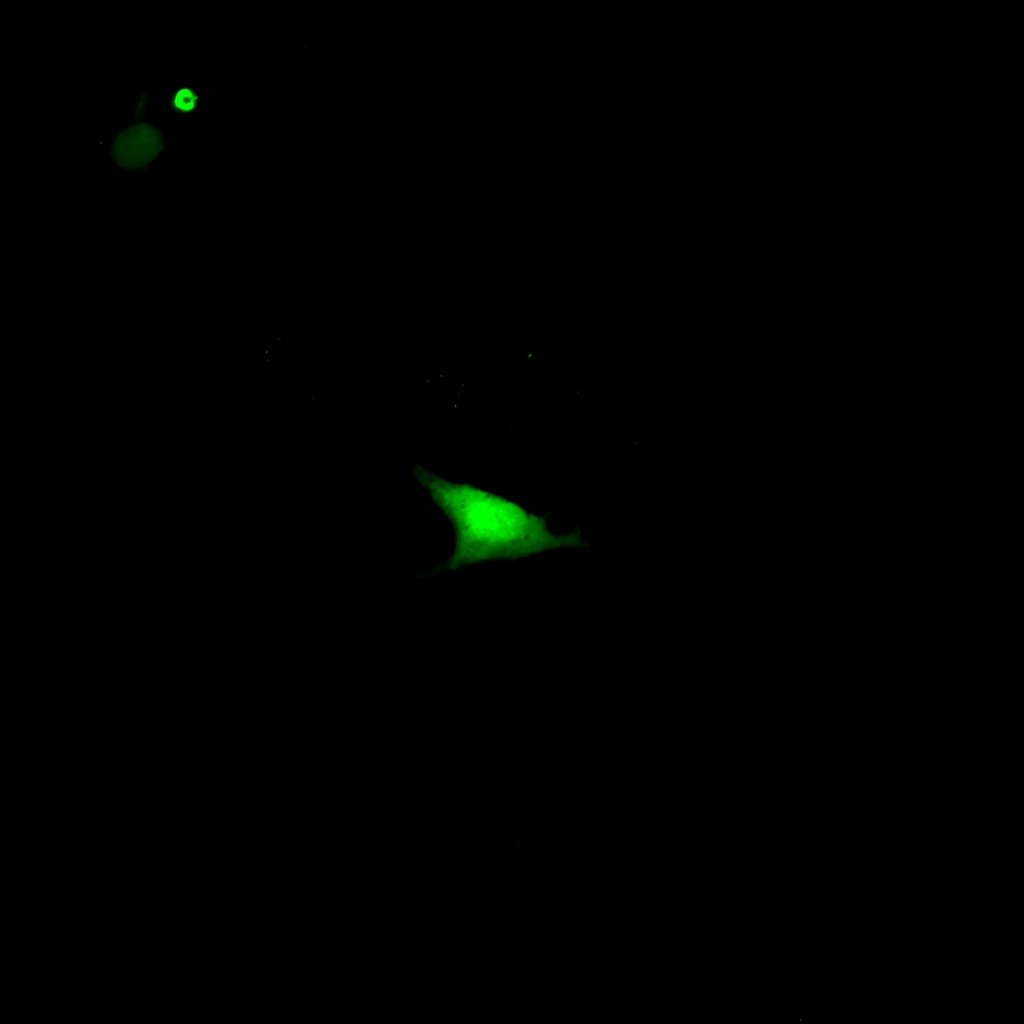

Supplement: Supplementary file 3 — Supplementary Data 1 [file 42003_2022_3856_MOESM3_ESM.zip › Figure 6/Fig. 6h/FLAG+GFP/gfp.jpg]

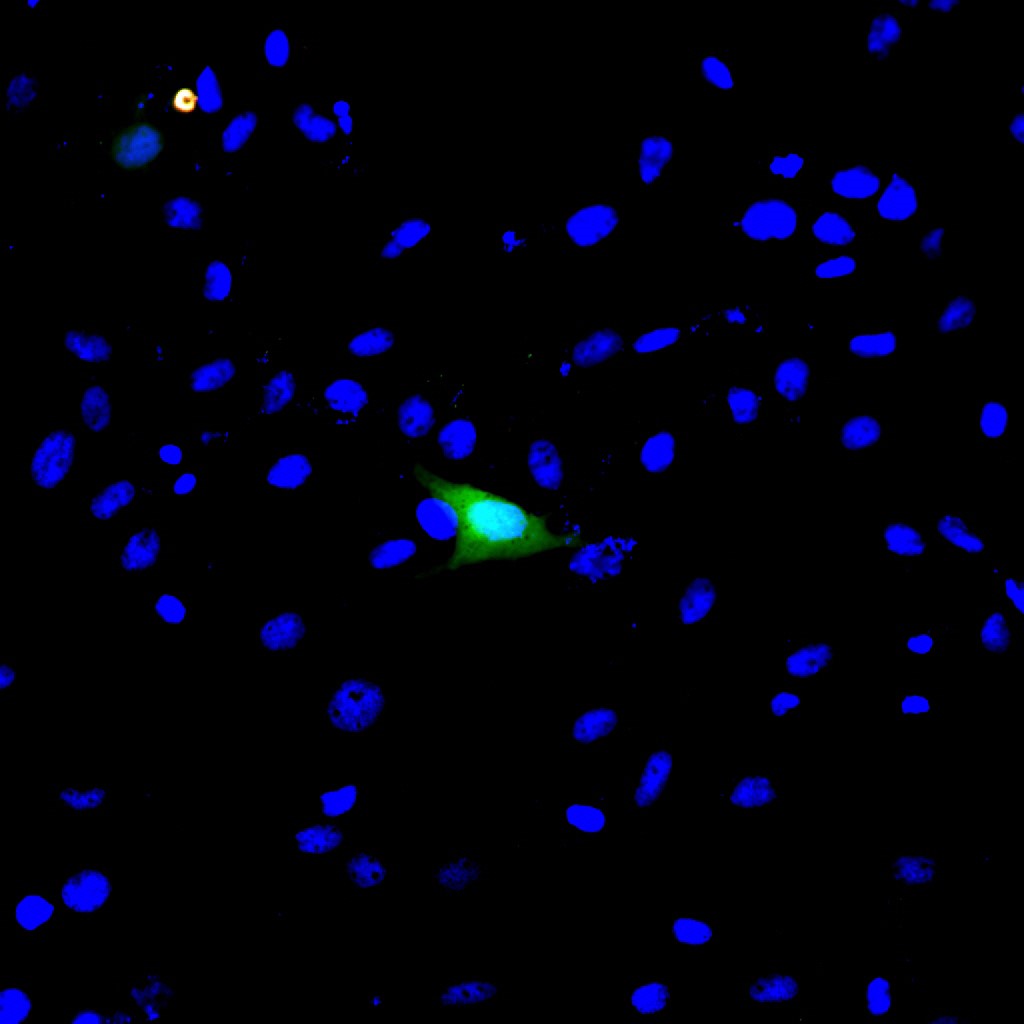

Supplement: Supplementary file 3 — Supplementary Data 1 [file 42003_2022_3856_MOESM3_ESM.zip › Figure 6/Fig. 6h/FLAG+GFP/Merge.jpg]

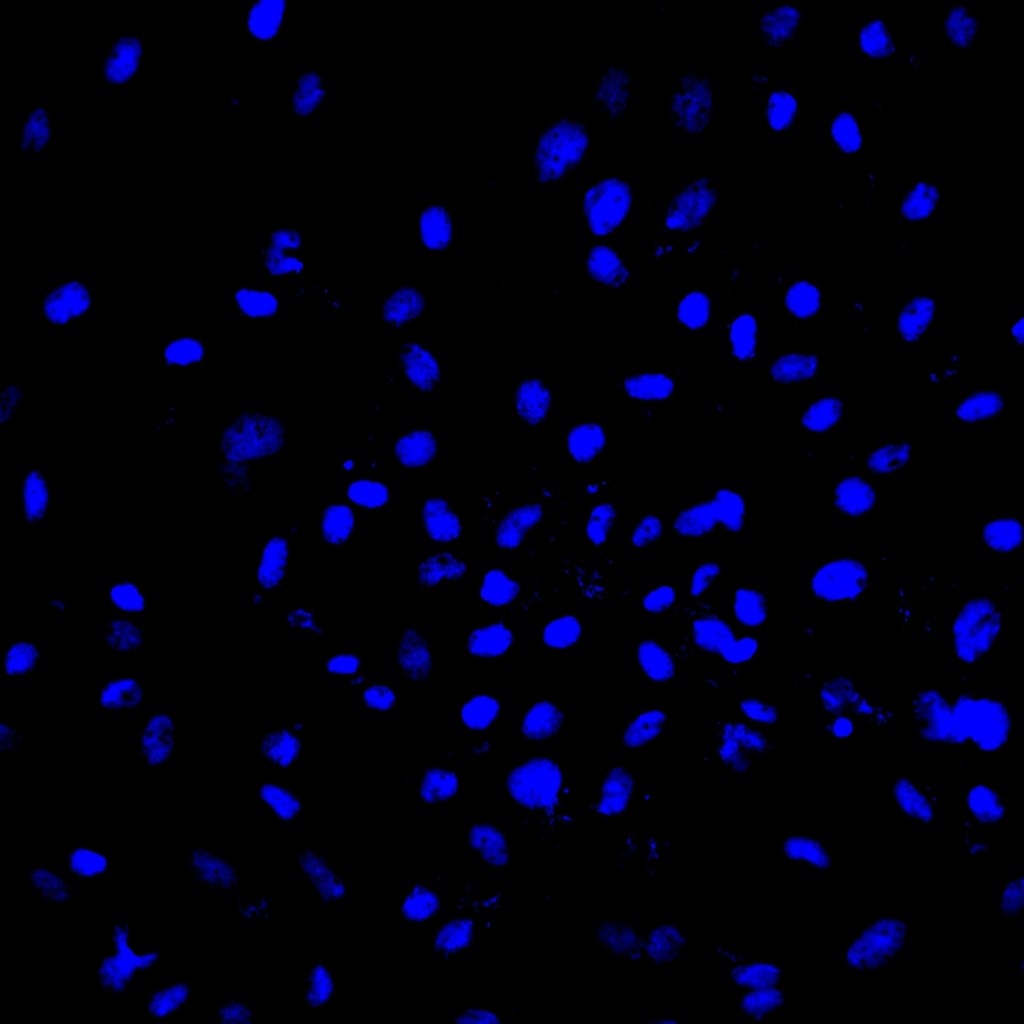

Supplement: Supplementary file 3 — Supplementary Data 1 [file 42003_2022_3856_MOESM3_ESM.zip › Figure 6/Fig. 6h/FLAG-CD44a-tv1+GFP-P53/dapi.jpg]

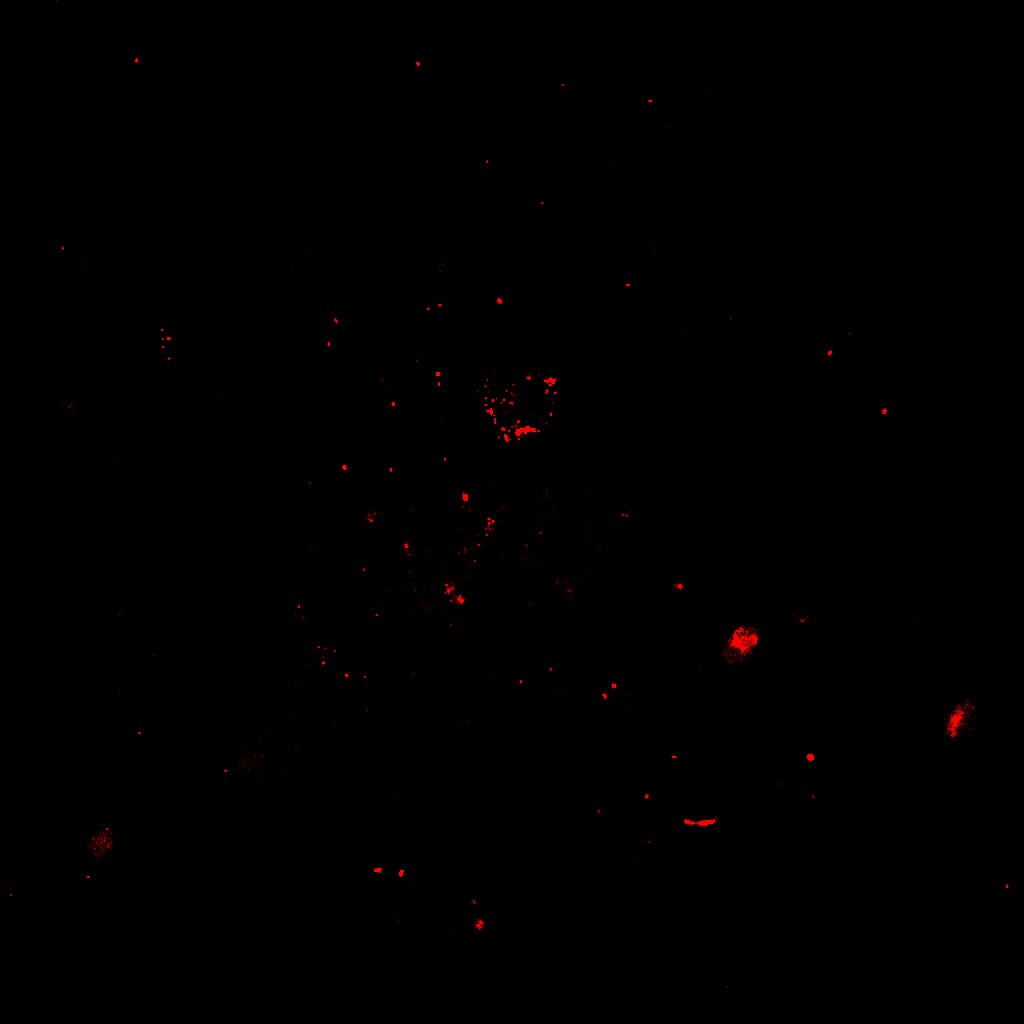

Supplement: Supplementary file 3 — Supplementary Data 1 [file 42003_2022_3856_MOESM3_ESM.zip › Figure 6/Fig. 6h/FLAG-CD44a-tv1+GFP-P53/FLAG-CD44a-tv1.jpg]

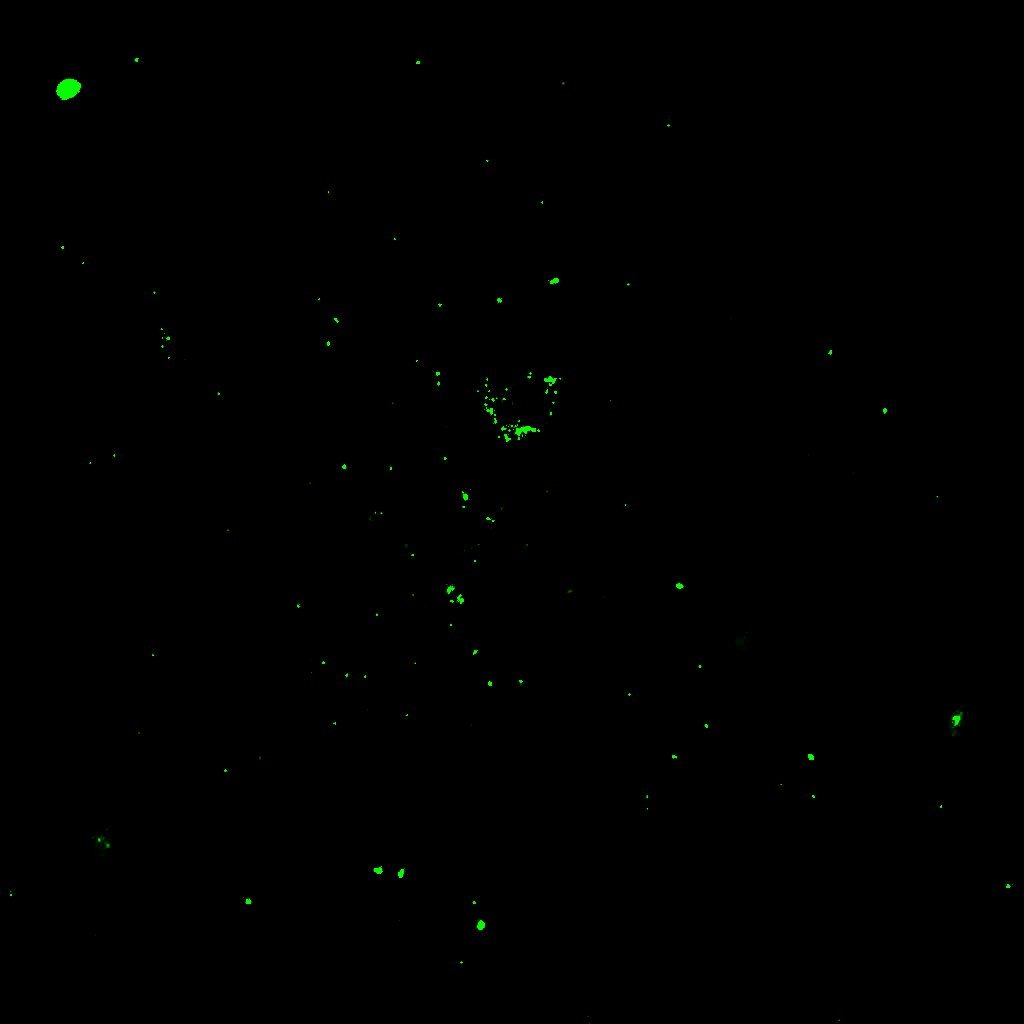

Supplement: Supplementary file 3 — Supplementary Data 1 [file 42003_2022_3856_MOESM3_ESM.zip › Figure 6/Fig. 6h/FLAG-CD44a-tv1+GFP-P53/GFP-P53.jpg]

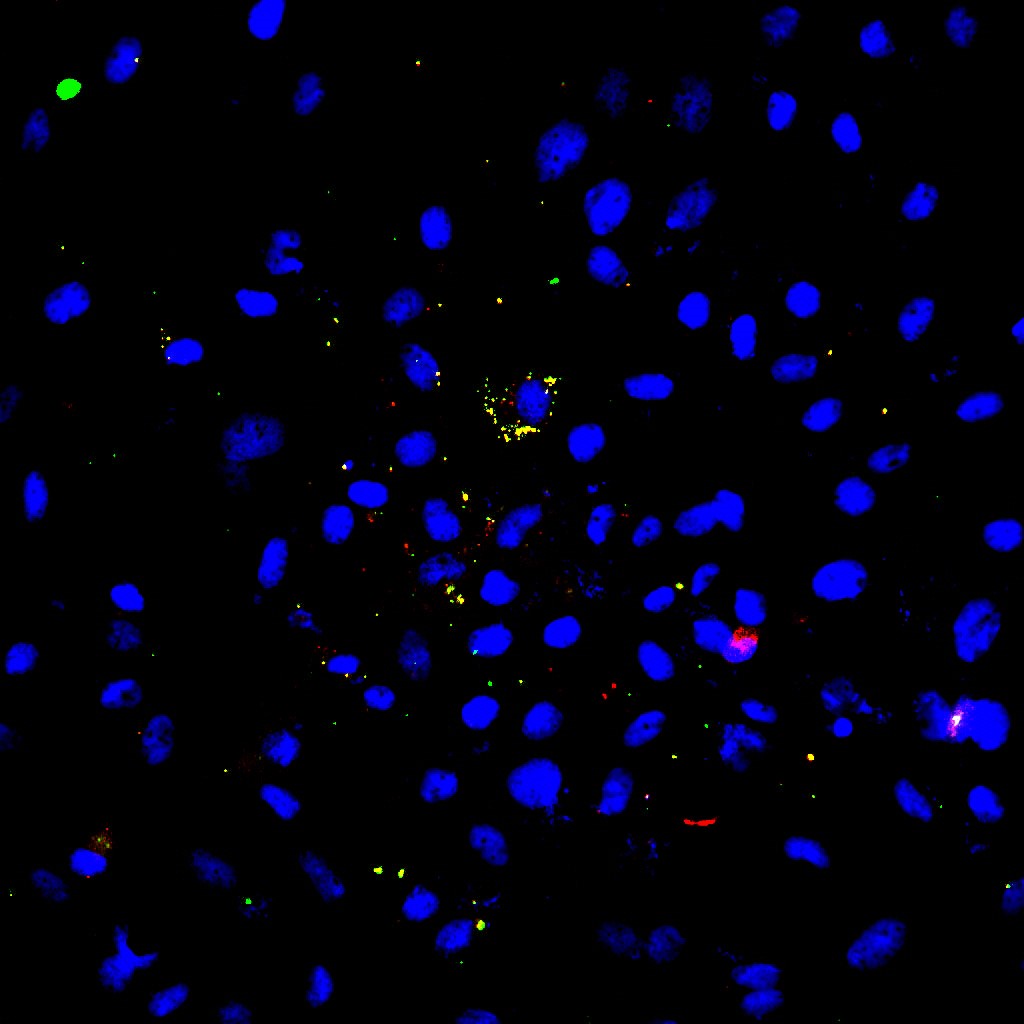

Supplement: Supplementary file 3 — Supplementary Data 1 [file 42003_2022_3856_MOESM3_ESM.zip › Figure 6/Fig. 6h/FLAG-CD44a-tv1+GFP-P53/merge.jpg]

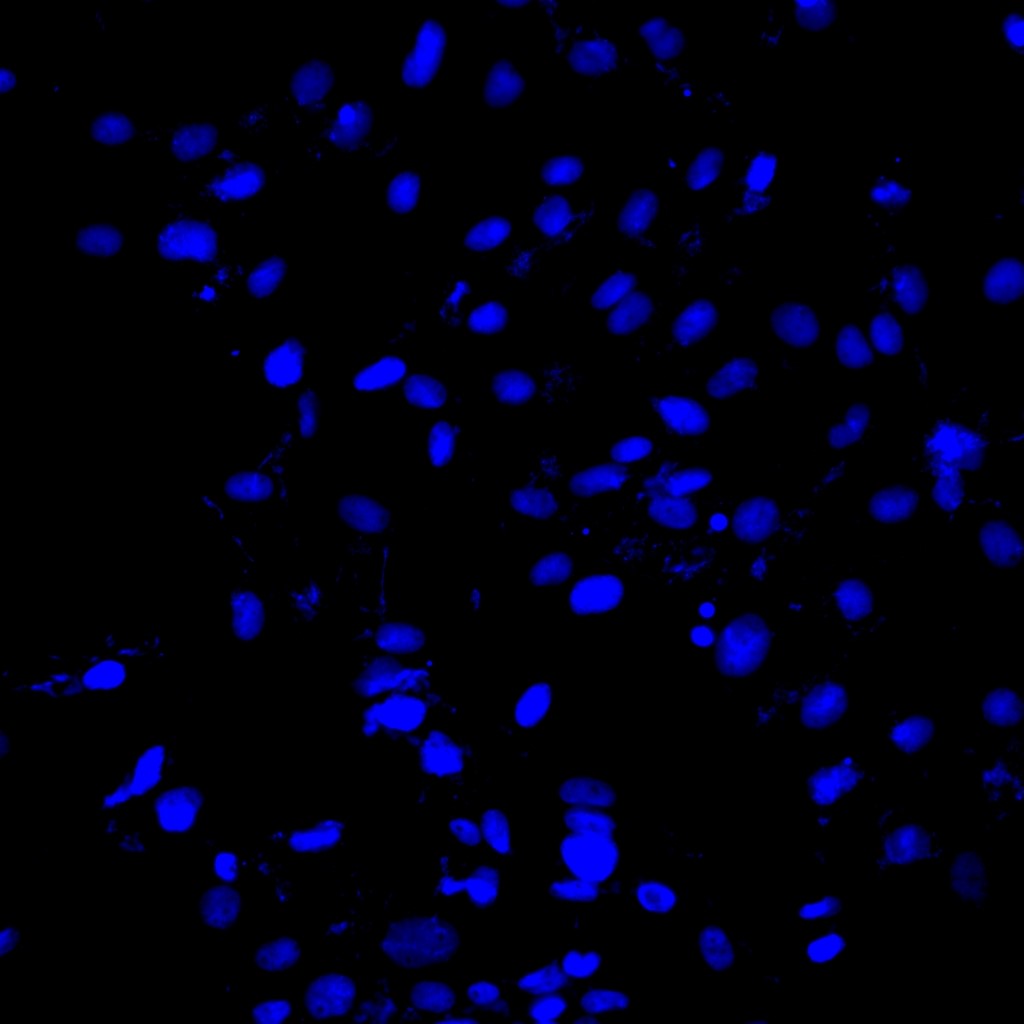

Supplement: Supplementary file 3 — Supplementary Data 1 [file 42003_2022_3856_MOESM3_ESM.zip › Figure 6/Fig. 6h/FLAG-CD44a-tv2+GFP-P53/DAPI.jpg]

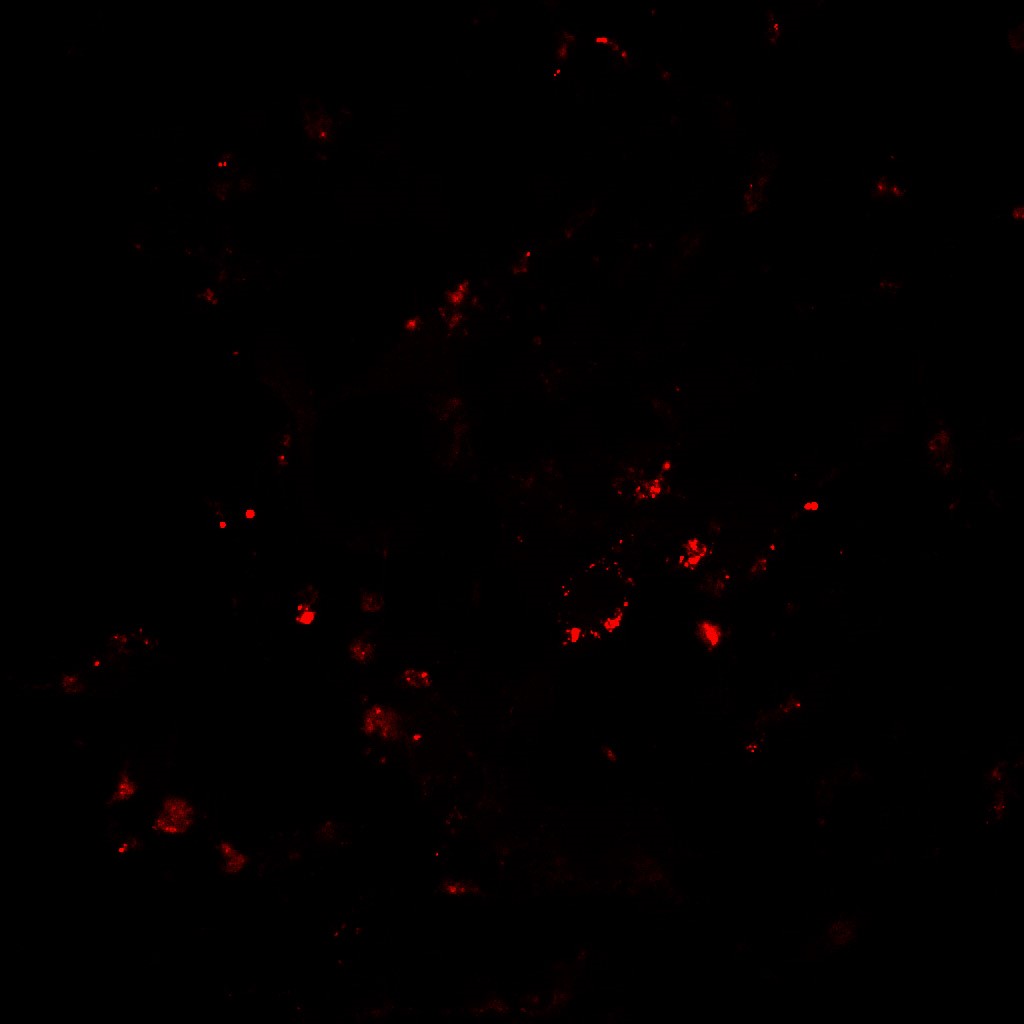

Supplement: Supplementary file 3 — Supplementary Data 1 [file 42003_2022_3856_MOESM3_ESM.zip › Figure 6/Fig. 6h/FLAG-CD44a-tv2+GFP-P53/flag-cd44a-tv2.jpg]

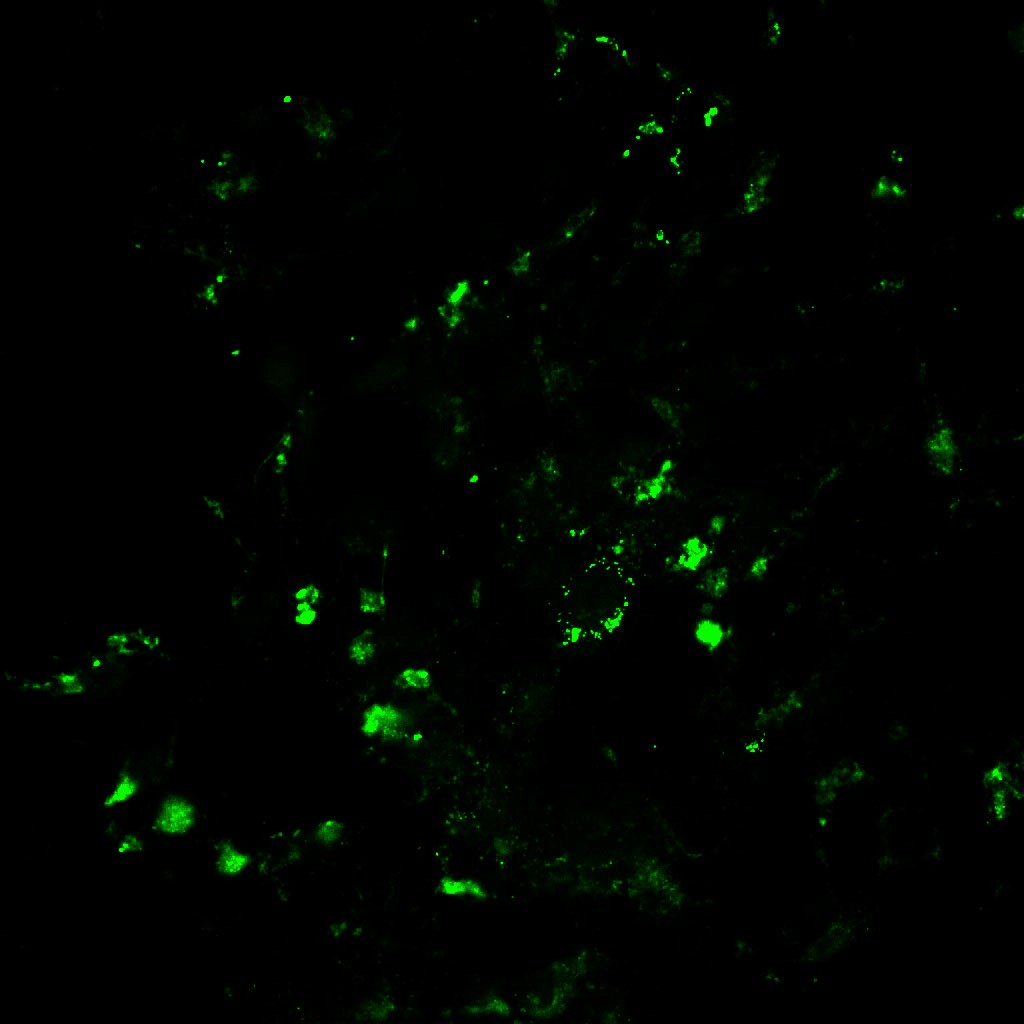

Supplement: Supplementary file 3 — Supplementary Data 1 [file 42003_2022_3856_MOESM3_ESM.zip › Figure 6/Fig. 6h/FLAG-CD44a-tv2+GFP-P53/gfp-p53.jpg]

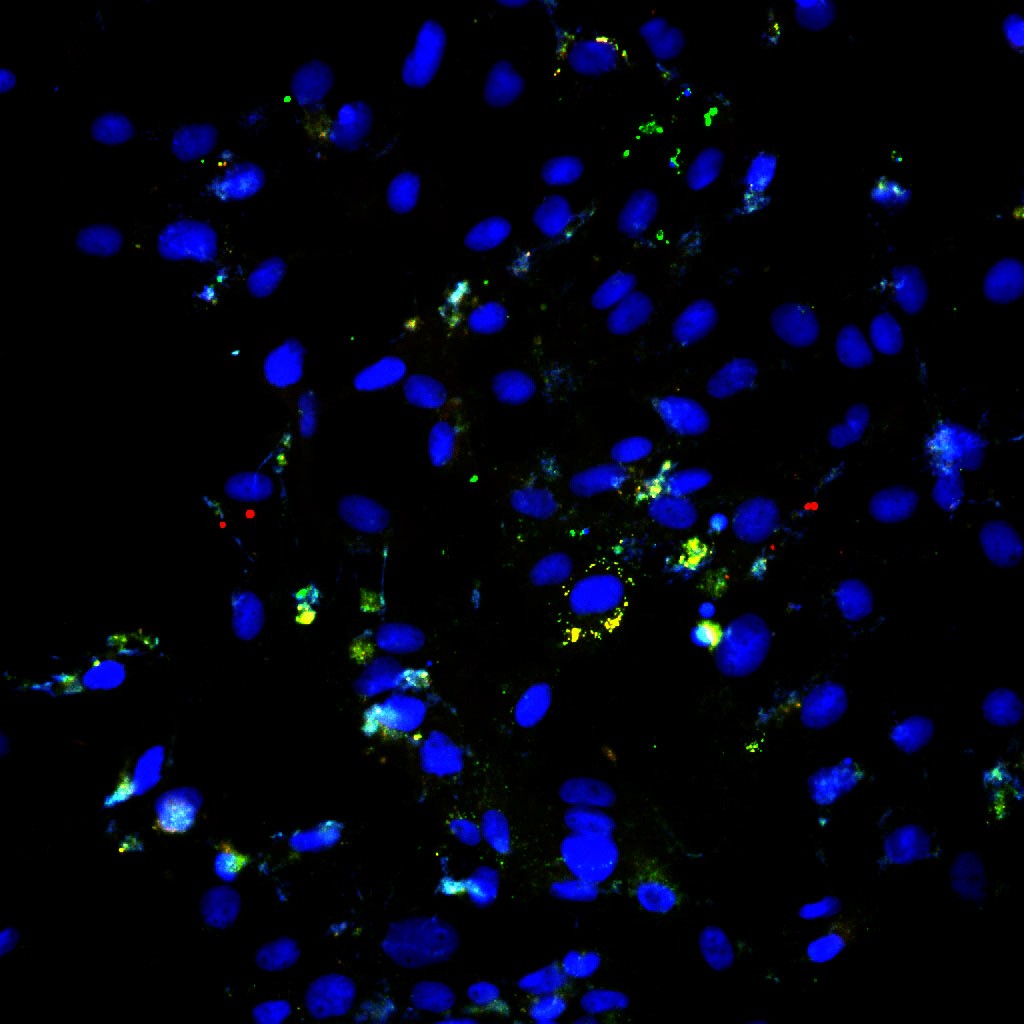

Supplement: Supplementary file 3 — Supplementary Data 1 [file 42003_2022_3856_MOESM3_ESM.zip › Figure 6/Fig. 6h/FLAG-CD44a-tv2+GFP-P53/Merge.jpg]

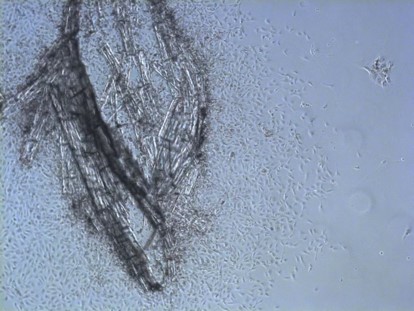

Supplement: Supplementary file 3 — Supplementary Data 1 [file 42003_2022_3856_MOESM3_ESM.zip › Supplementary Fig. 4/a/CD44a-14del.jpg]

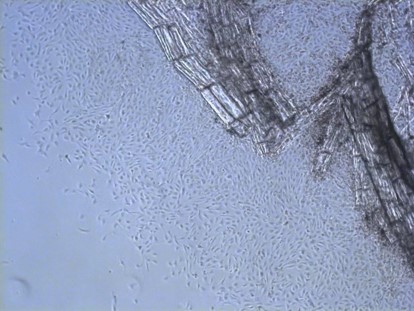

Supplement: Supplementary file 3 — Supplementary Data 1 [file 42003_2022_3856_MOESM3_ESM.zip › Supplementary Fig. 4/a/WT.jpg]

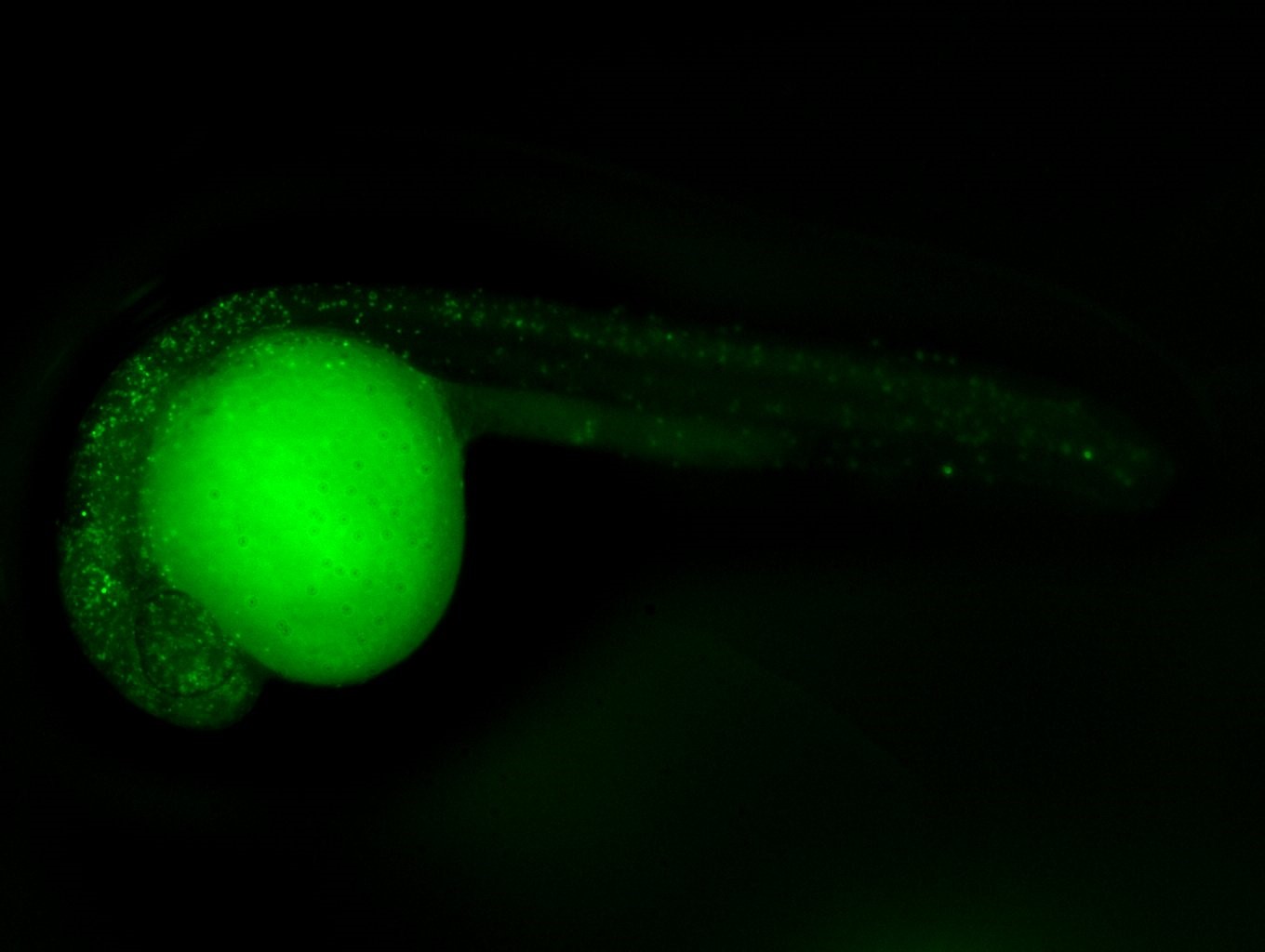

Supplement: Supplementary file 3 — Supplementary Data 1 [file 42003_2022_3856_MOESM3_ESM.zip › Supplementary Fig. 4/b and d/CD44a-14del+FLAG.jpg]

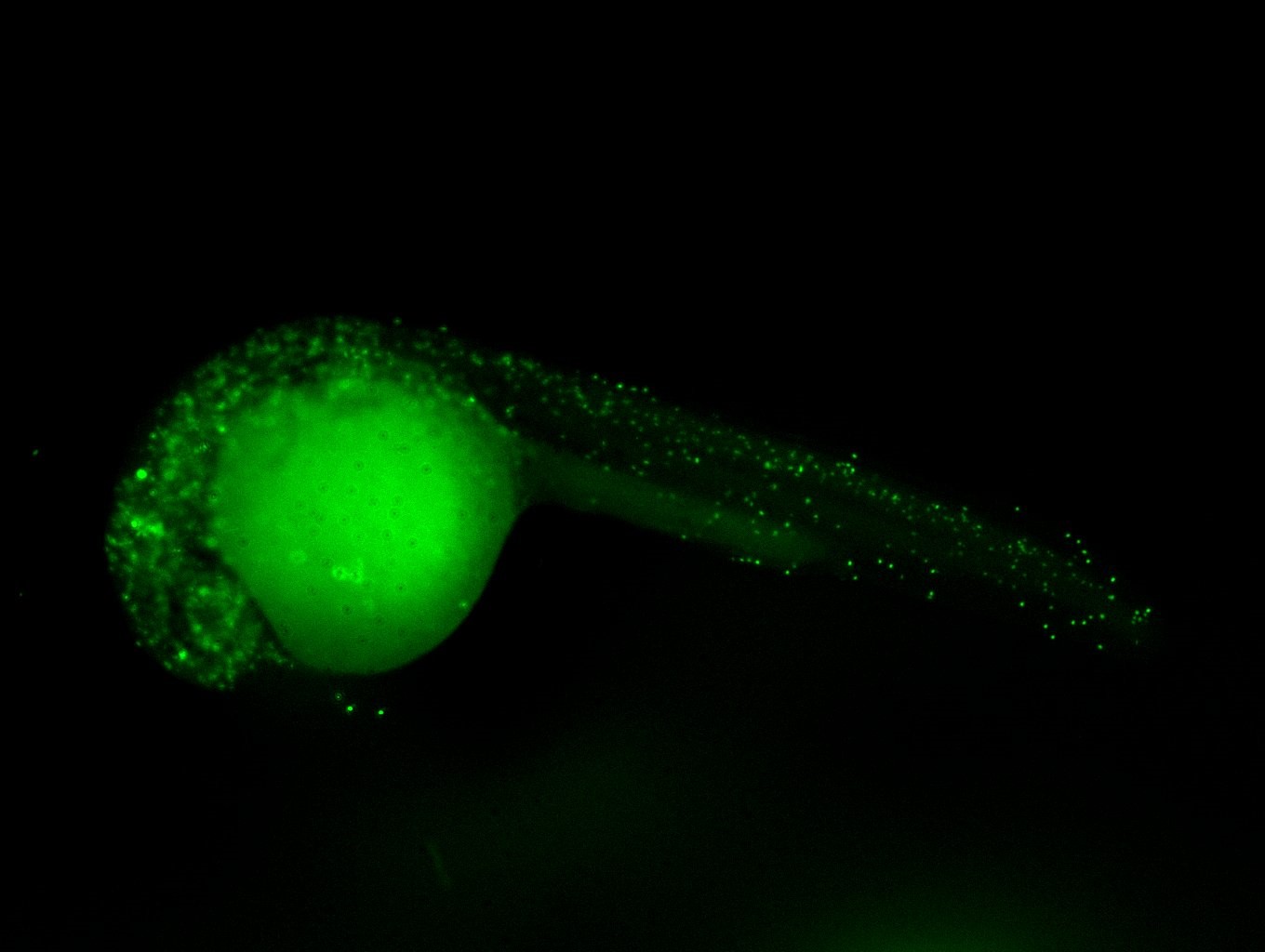

Supplement: Supplementary file 3 — Supplementary Data 1 [file 42003_2022_3856_MOESM3_ESM.zip › Supplementary Fig. 4/b and d/CD44a-14del+P53-FLAG.jpg]

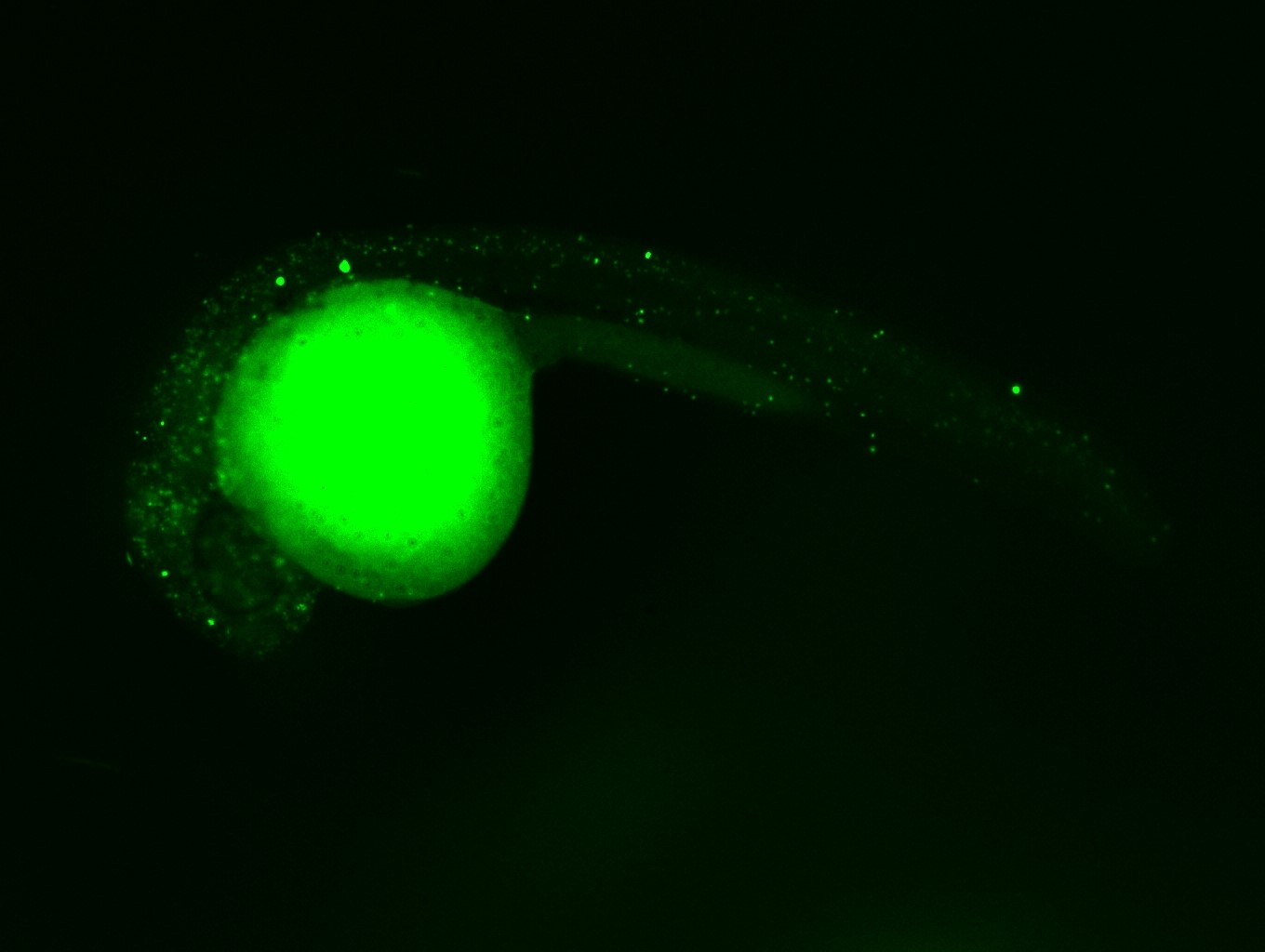

Supplement: Supplementary file 3 — Supplementary Data 1 [file 42003_2022_3856_MOESM3_ESM.zip › Supplementary Fig. 4/b and d/CD44a-14del.jpg]

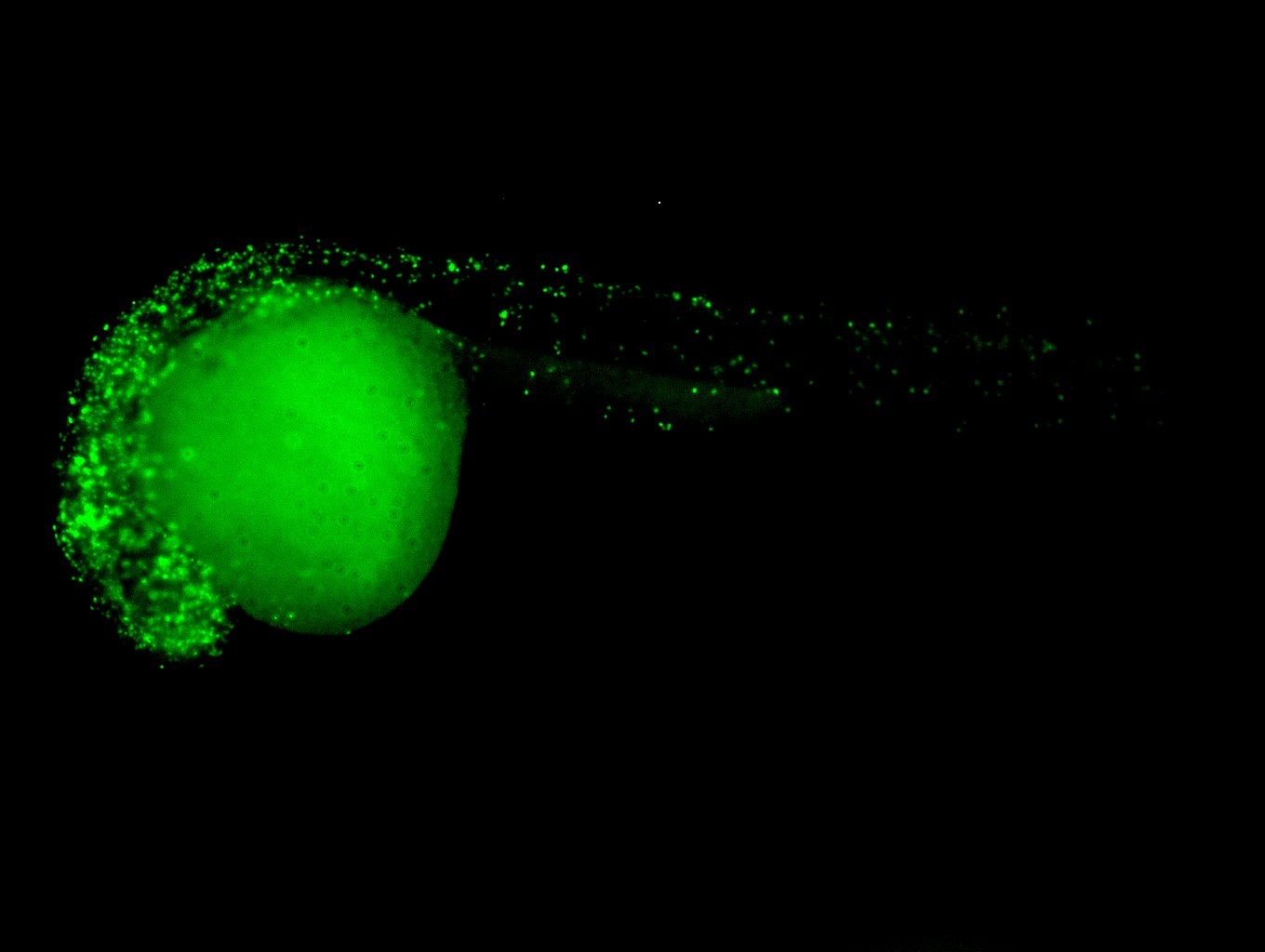

Supplement: Supplementary file 3 — Supplementary Data 1 [file 42003_2022_3856_MOESM3_ESM.zip › Supplementary Fig. 4/b and d/WT+FLAG.jpg]

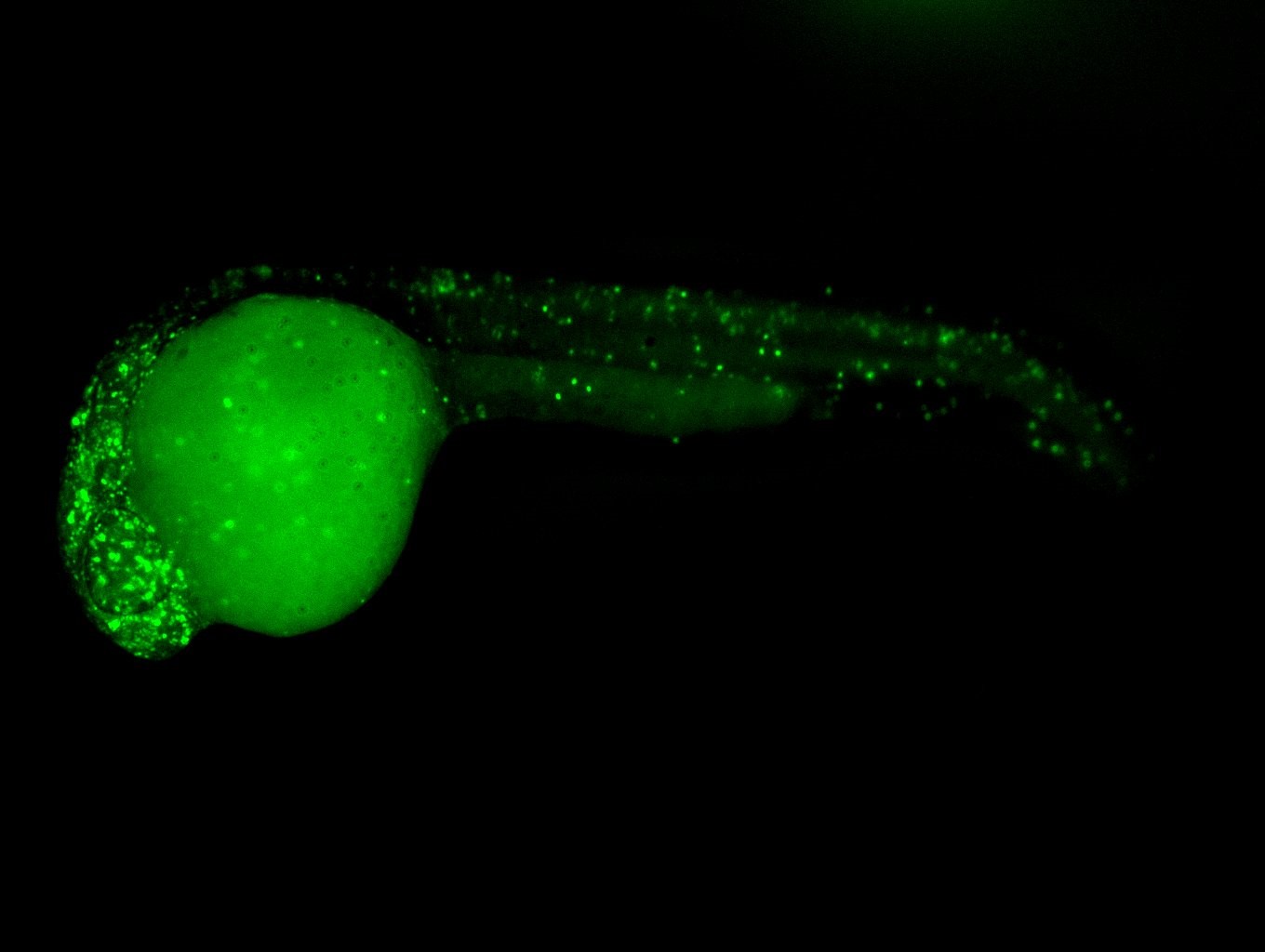

Supplement: Supplementary file 3 — Supplementary Data 1 [file 42003_2022_3856_MOESM3_ESM.zip › Supplementary Fig. 4/b and d/WT+P53-FLAG.jpg]

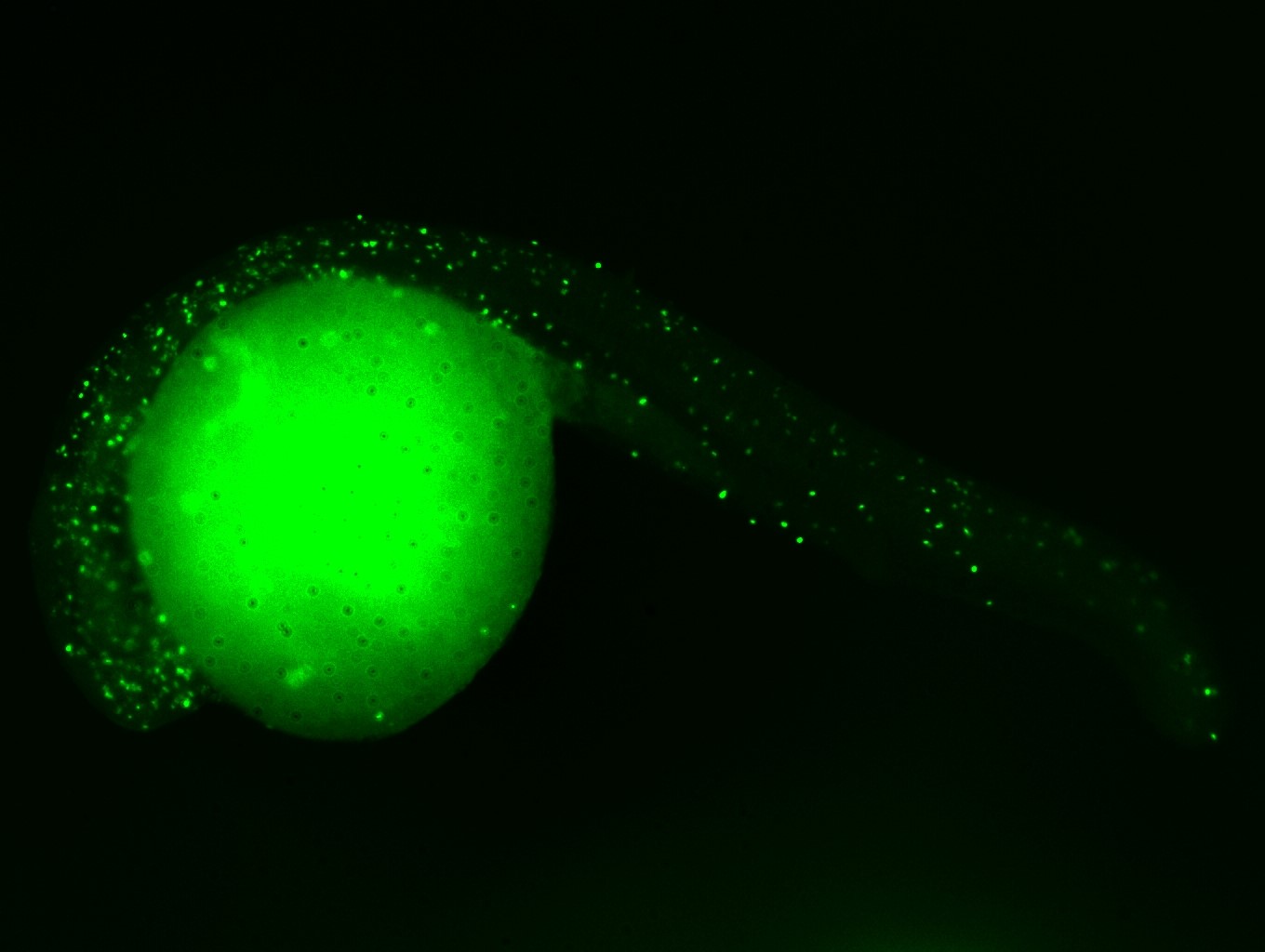

Supplement: Supplementary file 3 — Supplementary Data 1 [file 42003_2022_3856_MOESM3_ESM.zip › Supplementary Fig. 4/b and d/WT.jpg]

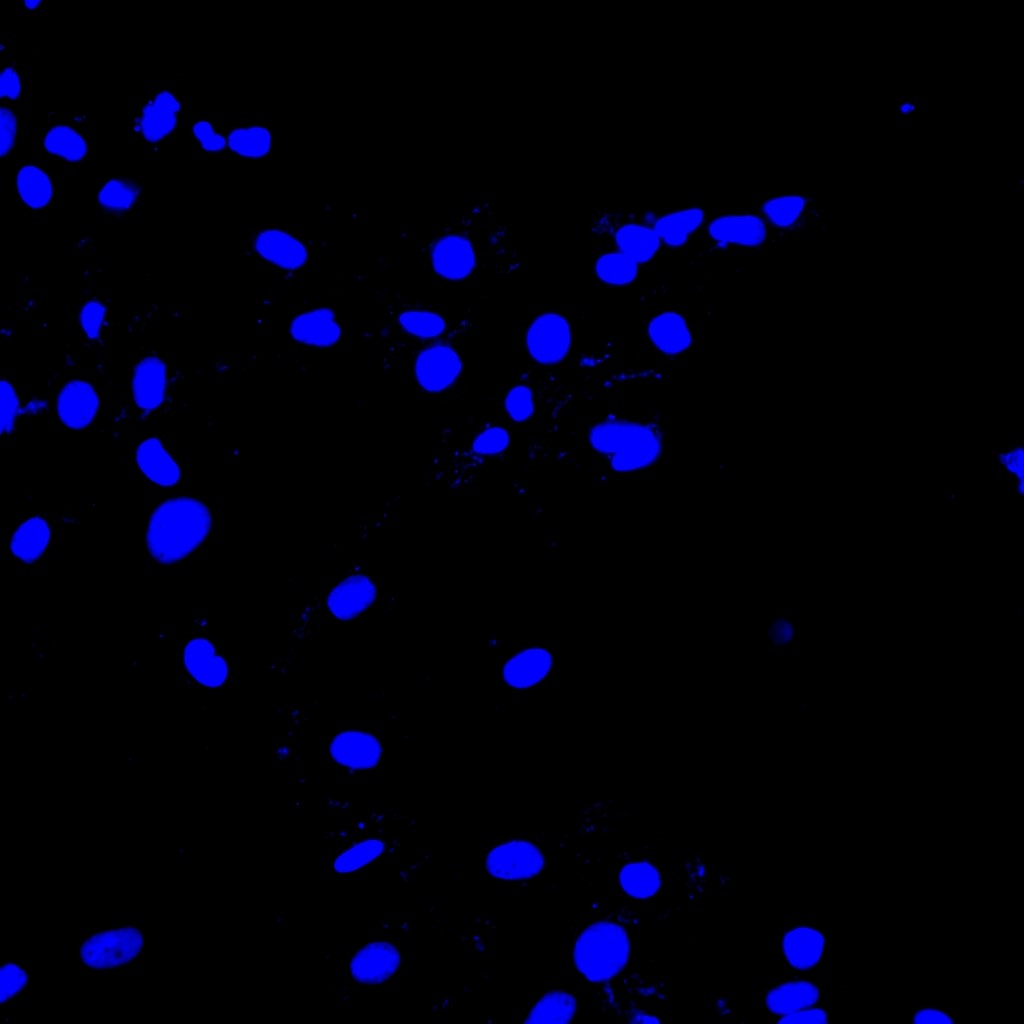

Supplement: Supplementary file 3 — Supplementary Data 1 [file 42003_2022_3856_MOESM3_ESM.zip › Supplementary Fig. 5/c/FLAG+GFP-p53/DAPI.jpg]

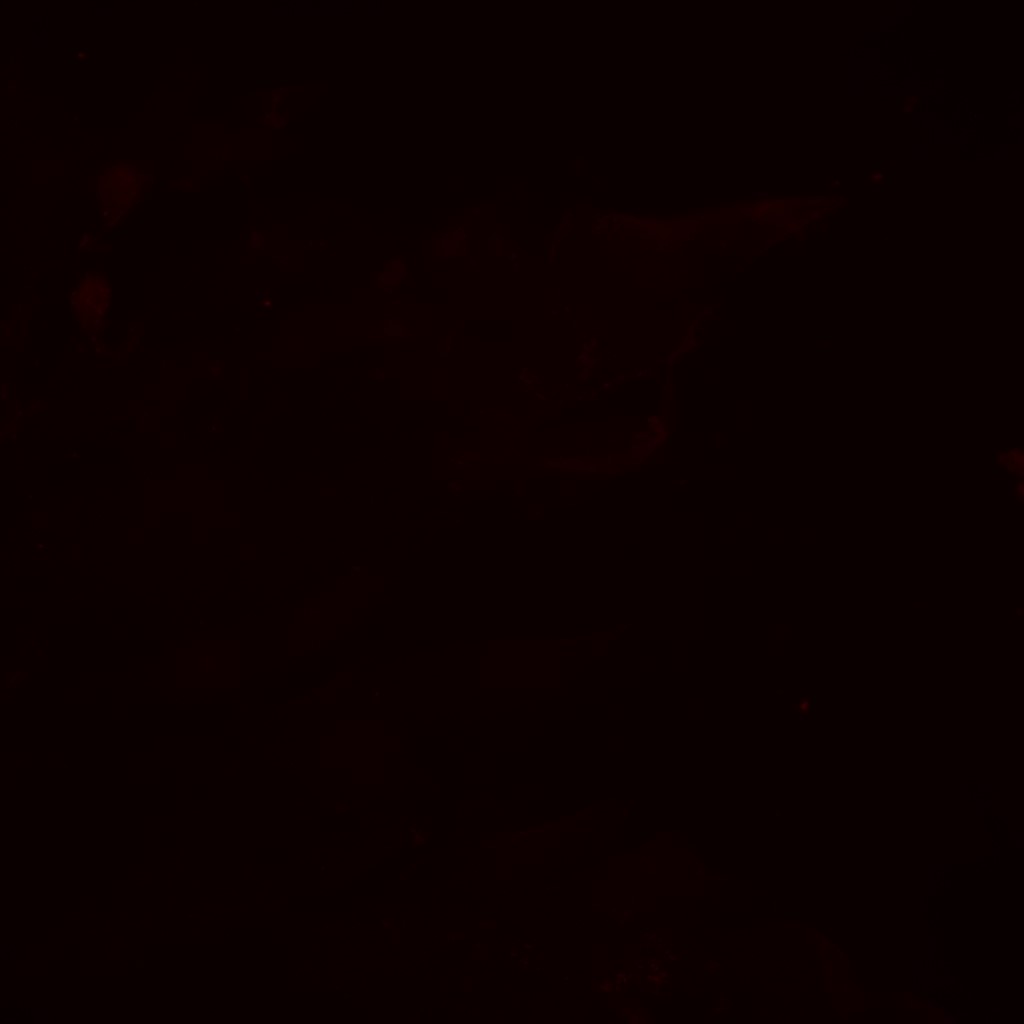

Supplement: Supplementary file 3 — Supplementary Data 1 [file 42003_2022_3856_MOESM3_ESM.zip › Supplementary Fig. 5/c/FLAG+GFP-p53/FLAG.jpg]

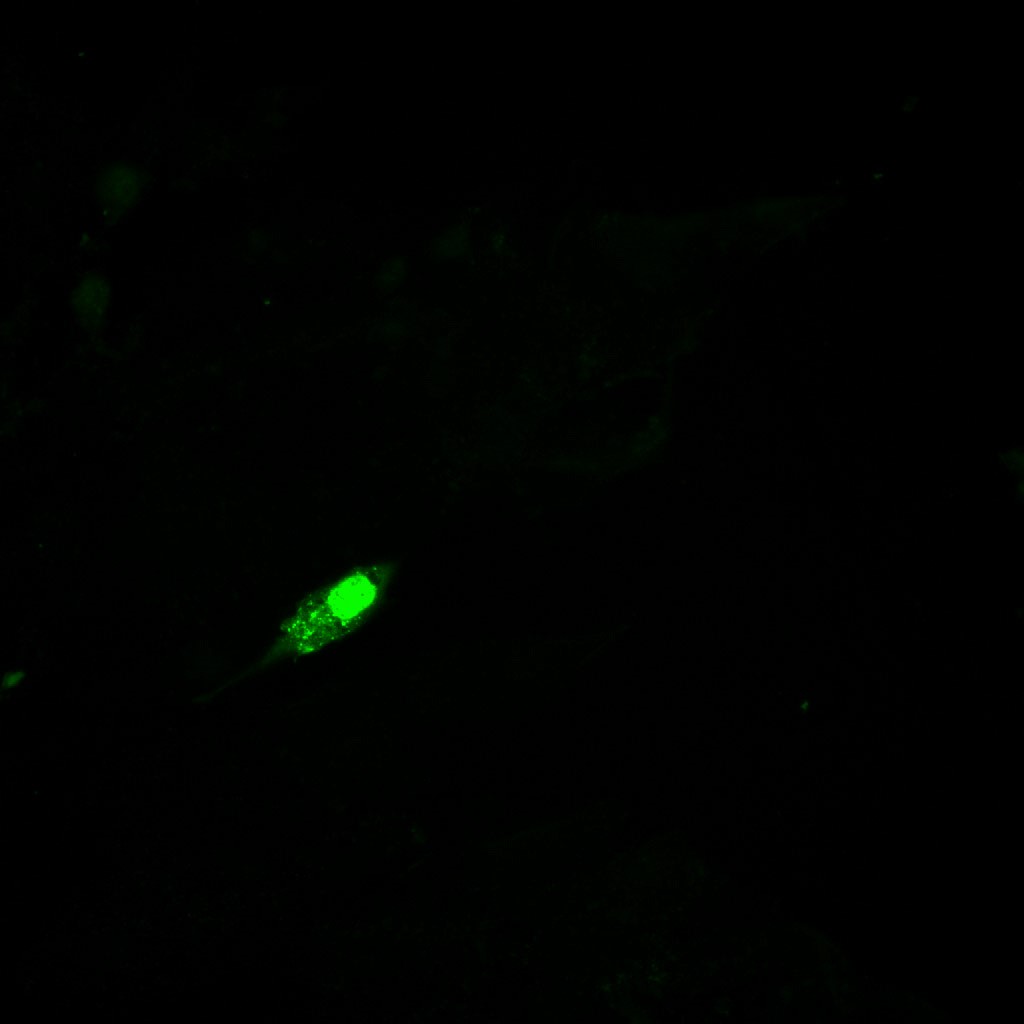

Supplement: Supplementary file 3 — Supplementary Data 1 [file 42003_2022_3856_MOESM3_ESM.zip › Supplementary Fig. 5/c/FLAG+GFP-p53/GFP-P53.jpg]

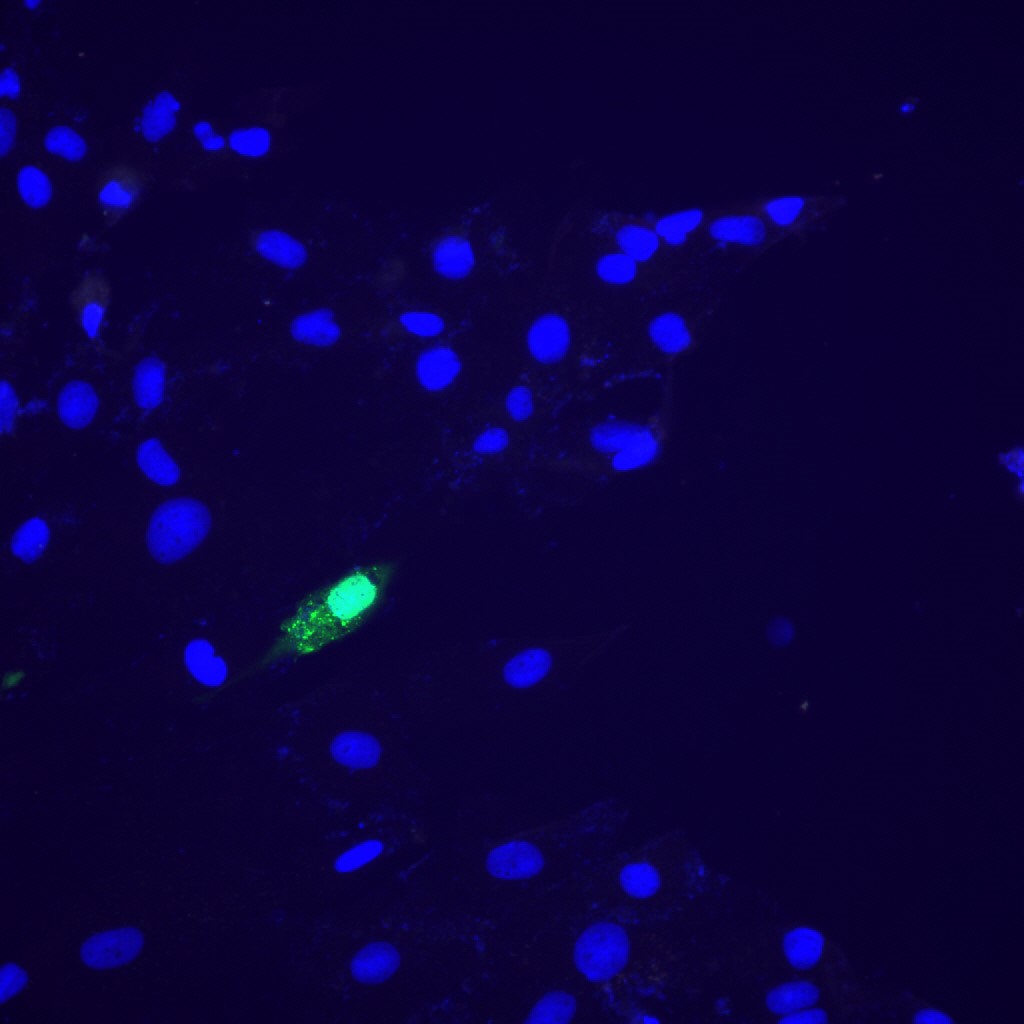

Supplement: Supplementary file 3 — Supplementary Data 1 [file 42003_2022_3856_MOESM3_ESM.zip › Supplementary Fig. 5/c/FLAG+GFP-p53/Merge.jpg]

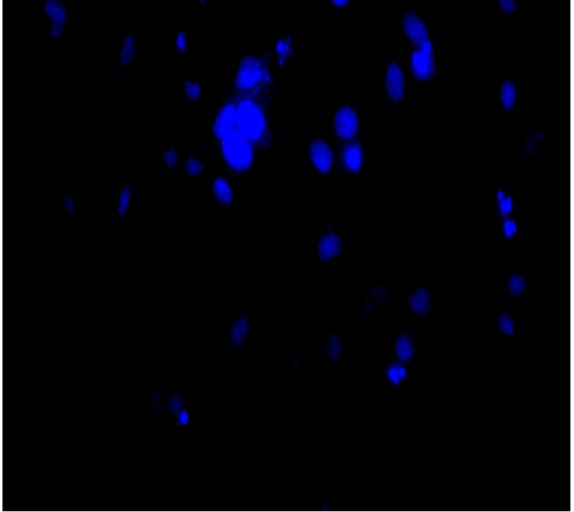

Supplement: Supplementary file 3 — Supplementary Data 1 [file 42003_2022_3856_MOESM3_ESM.zip › Supplementary Fig. 5/c/FLAG+GFP/DAPI.jpg]

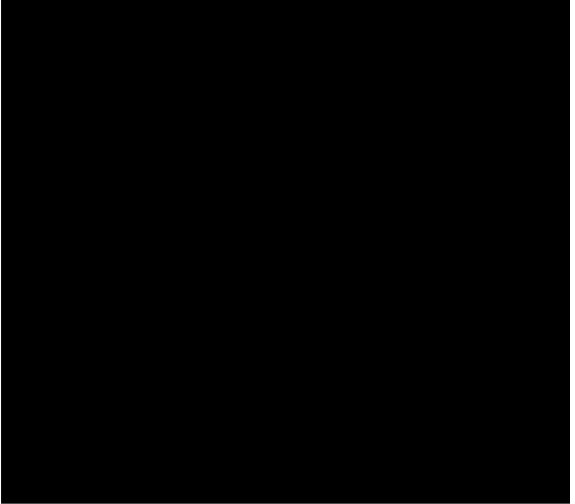

Supplement: Supplementary file 3 — Supplementary Data 1 [file 42003_2022_3856_MOESM3_ESM.zip › Supplementary Fig. 5/c/FLAG+GFP/FLAG.jpg]

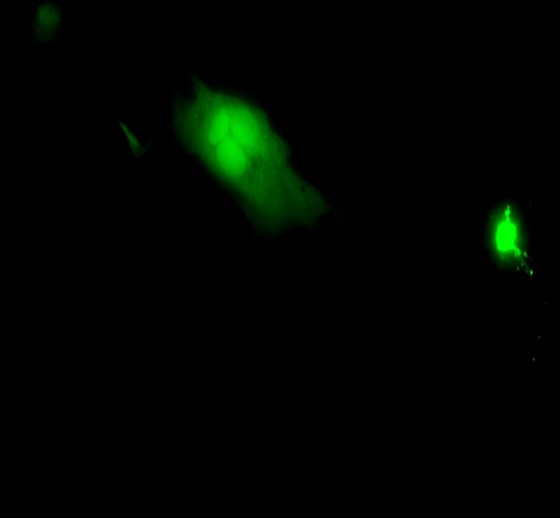

Supplement: Supplementary file 3 — Supplementary Data 1 [file 42003_2022_3856_MOESM3_ESM.zip › Supplementary Fig. 5/c/FLAG+GFP/gfp.jpg]

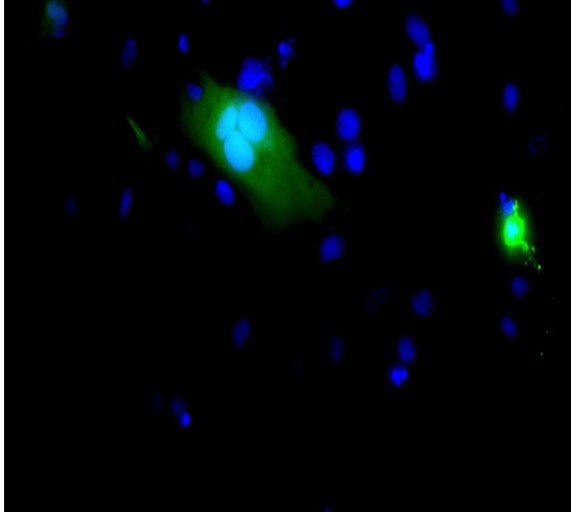

Supplement: Supplementary file 3 — Supplementary Data 1 [file 42003_2022_3856_MOESM3_ESM.zip › Supplementary Fig. 5/c/FLAG+GFP/merge.jpg]

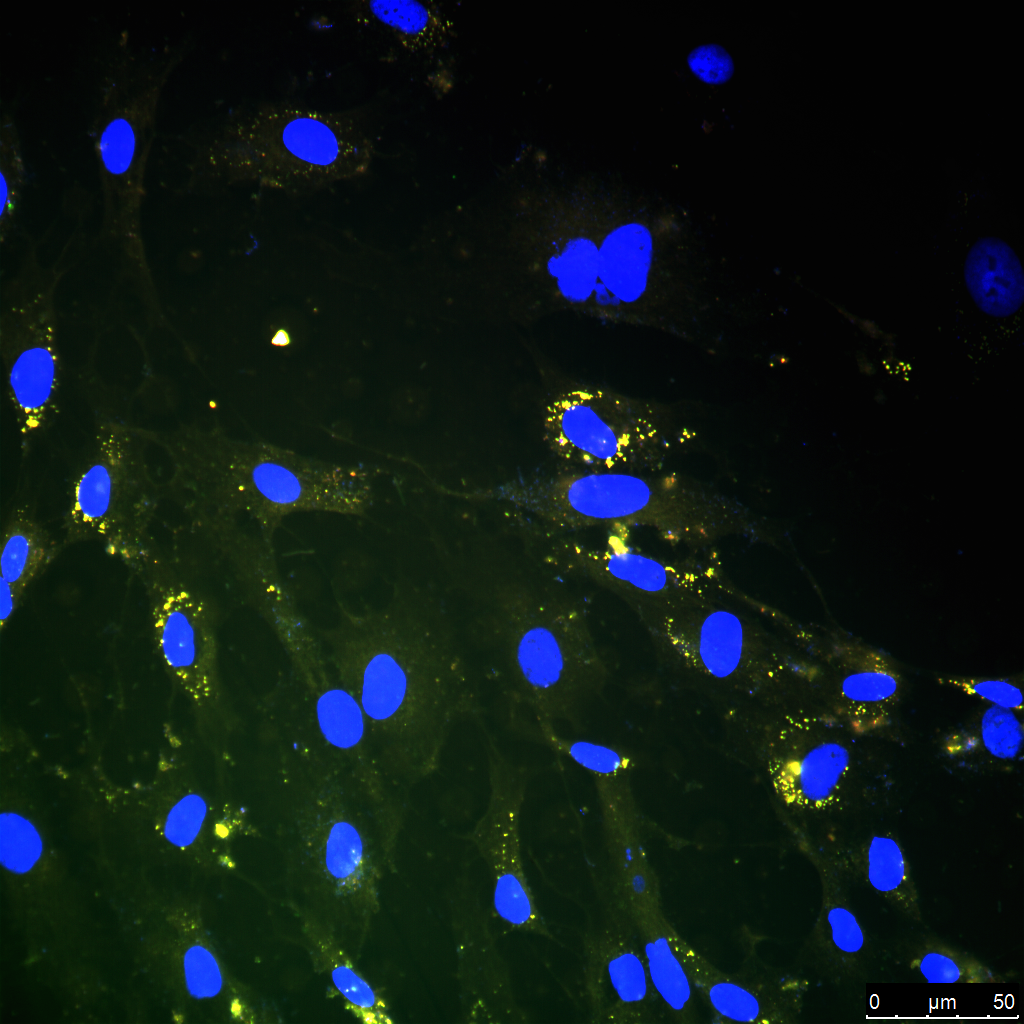

Supplement: Supplementary file 3 — Supplementary Data 1 [file 42003_2022_3856_MOESM3_ESM.zip › Supplementary Fig. 5/c/FLAG-CD44a-tv1+GFP-p53/CD44a1-FLAG +p53-FLAG.lif_Series001.tif]

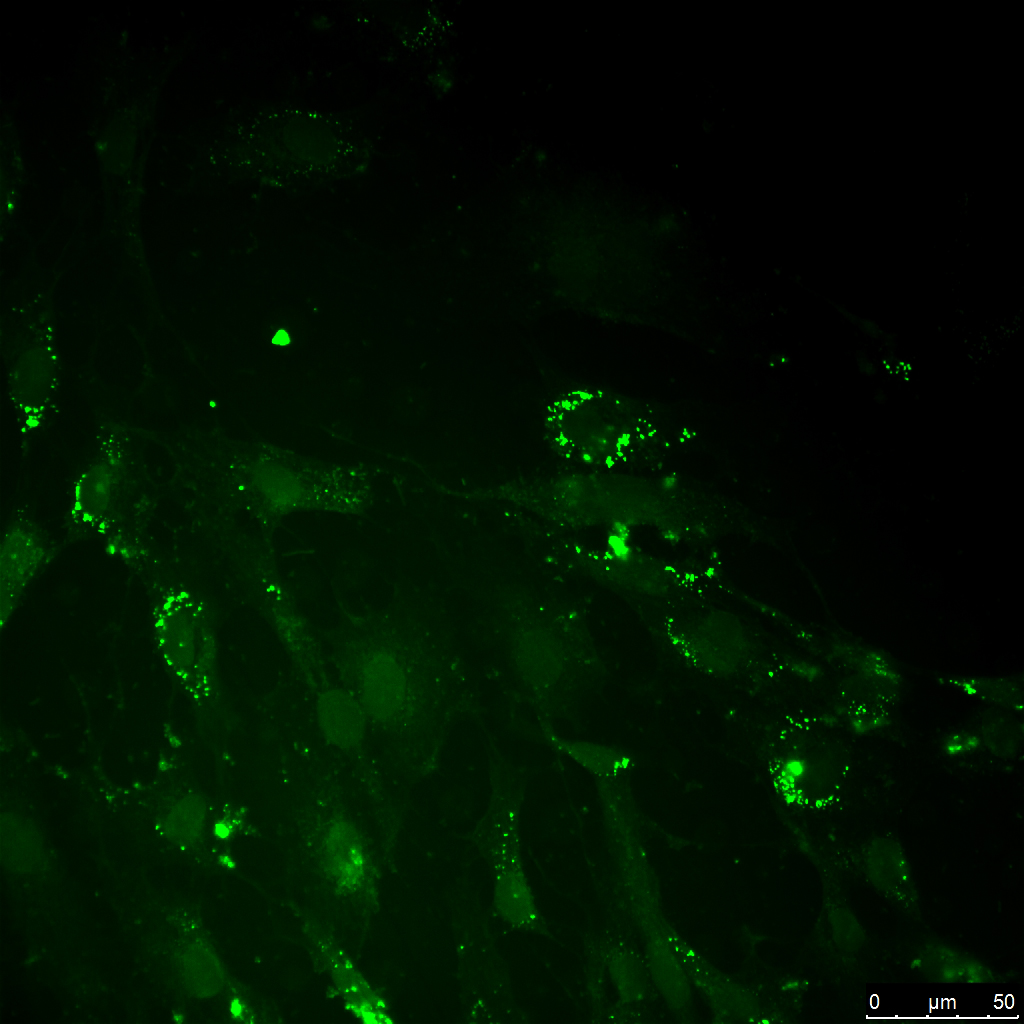

Supplement: Supplementary file 3 — Supplementary Data 1 [file 42003_2022_3856_MOESM3_ESM.zip › Supplementary Fig. 5/c/FLAG-CD44a-tv1+GFP-p53/CD44a1-FLAG +p53-FLAG.lif_Series001_ch01.jpg]

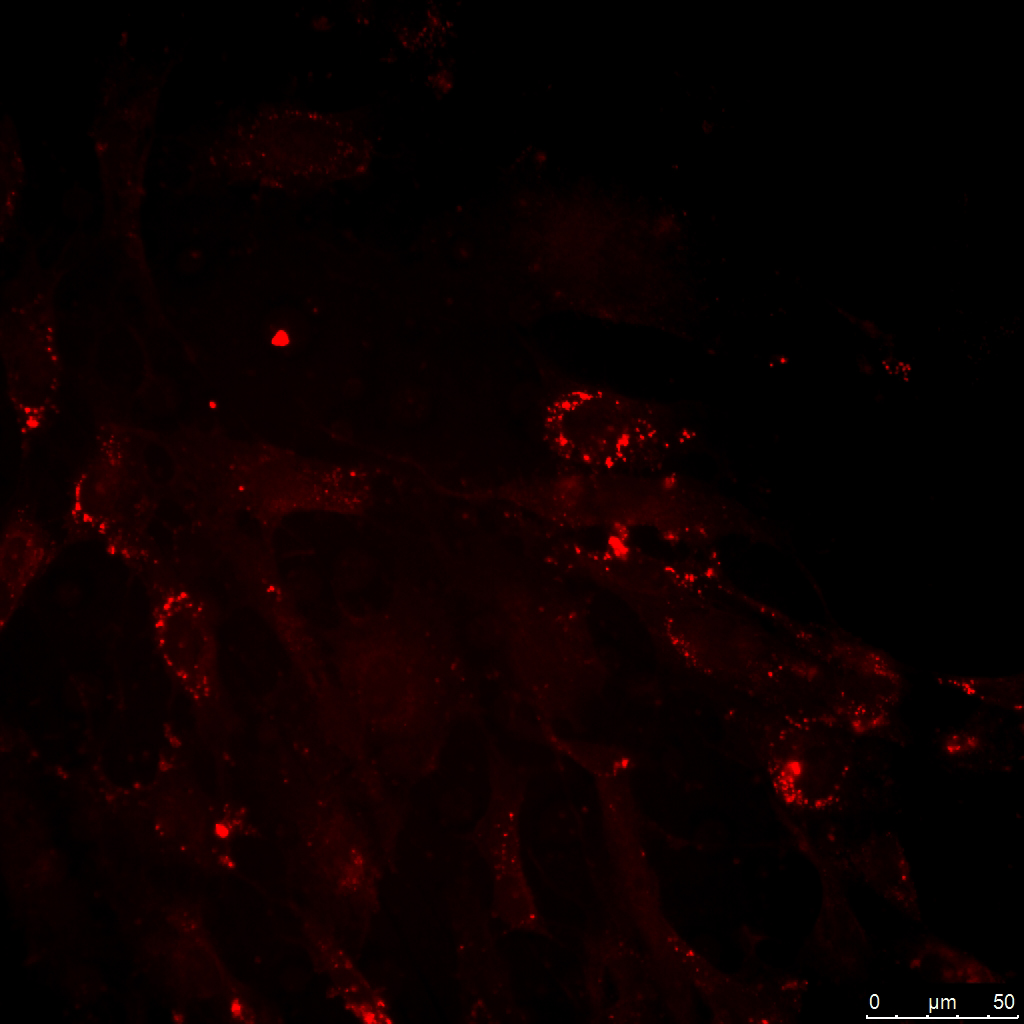

Supplement: Supplementary file 3 — Supplementary Data 1 [file 42003_2022_3856_MOESM3_ESM.zip › Supplementary Fig. 5/c/FLAG-CD44a-tv1+GFP-p53/CD44a1-FLAG +p53-FLAG.lif_Series001_ch02.jpg]

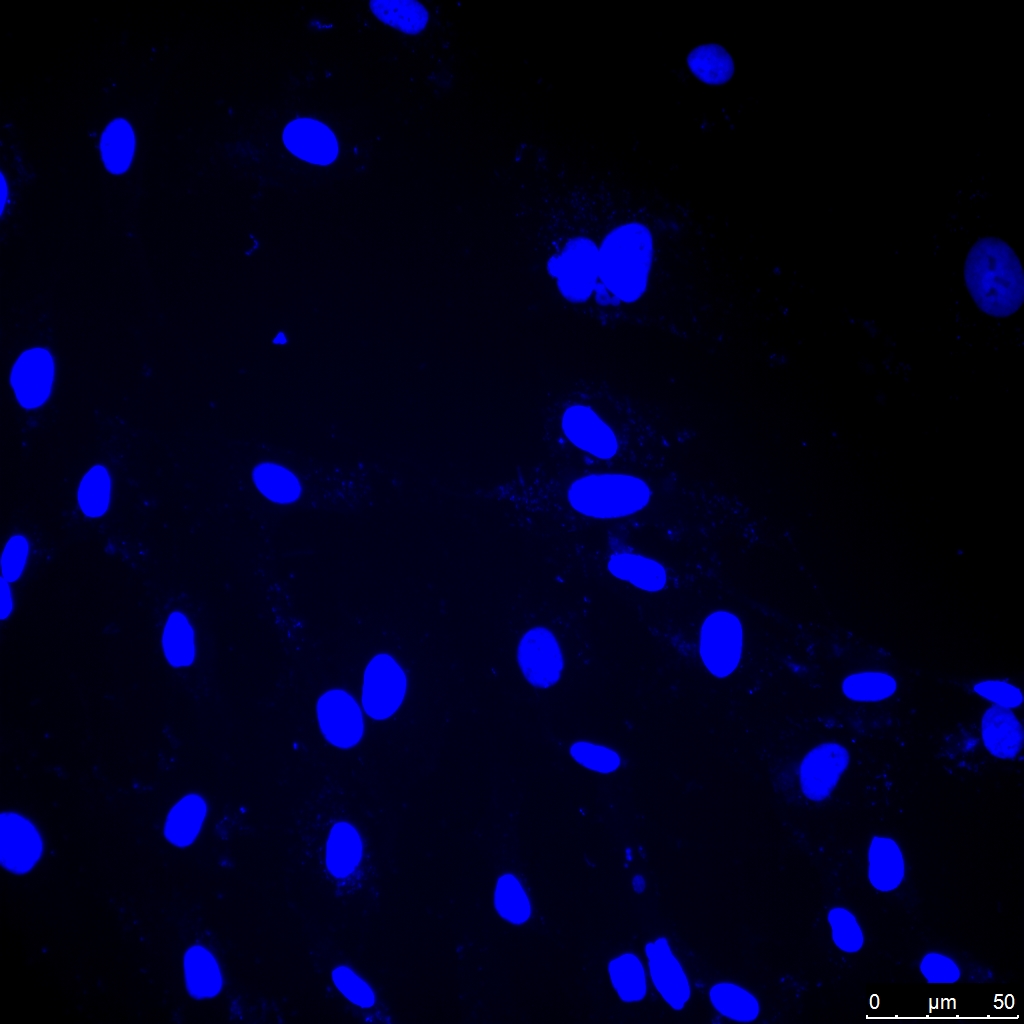

Supplement: Supplementary file 3 — Supplementary Data 1 [file 42003_2022_3856_MOESM3_ESM.zip › Supplementary Fig. 5/c/FLAG-CD44a-tv1+GFP-p53/CD44a1-FLAG +p53-FLAG.lif_Series001_ch03.jpg]

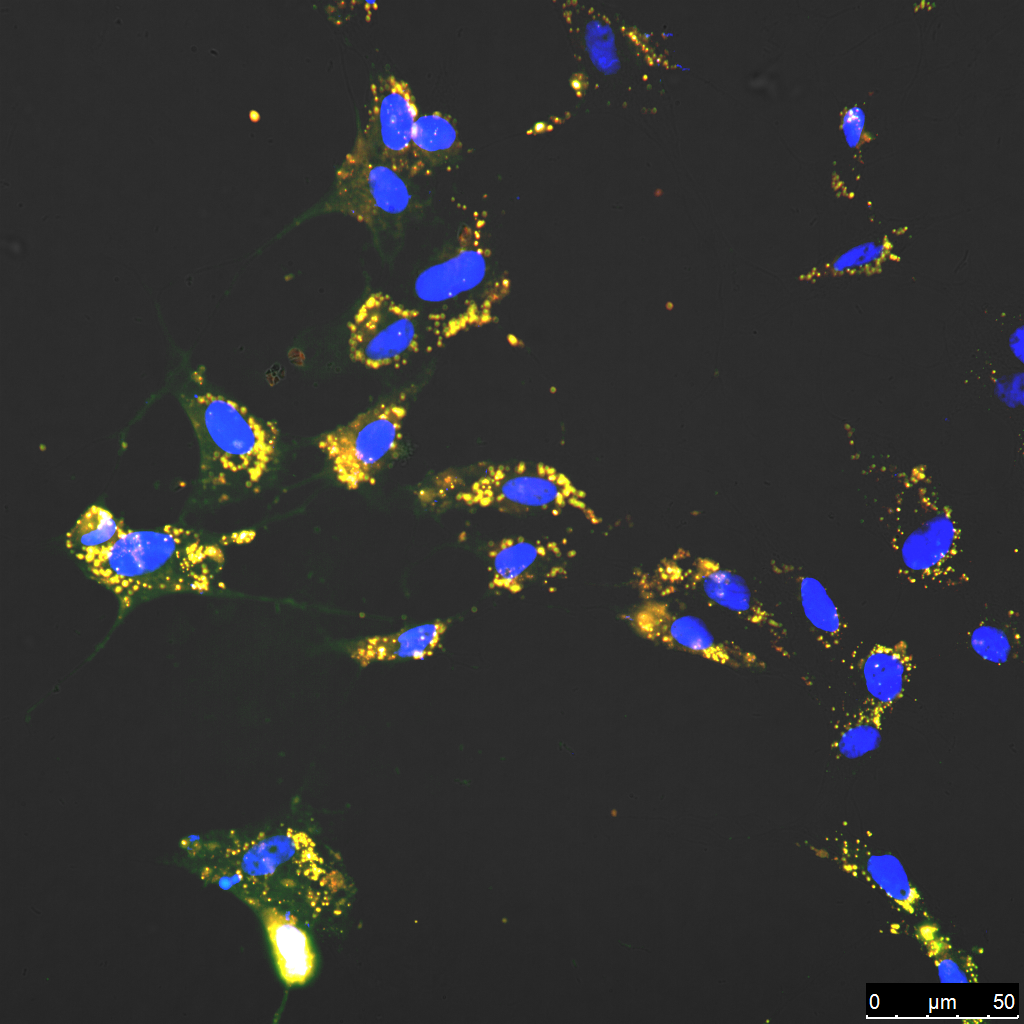

Supplement: Supplementary file 3 — Supplementary Data 1 [file 42003_2022_3856_MOESM3_ESM.zip › Supplementary Fig. 5/c/FLAG-CD44a-tv2+GFP-p53/cd44a2+p53-gfp.lif_Series007.tif]

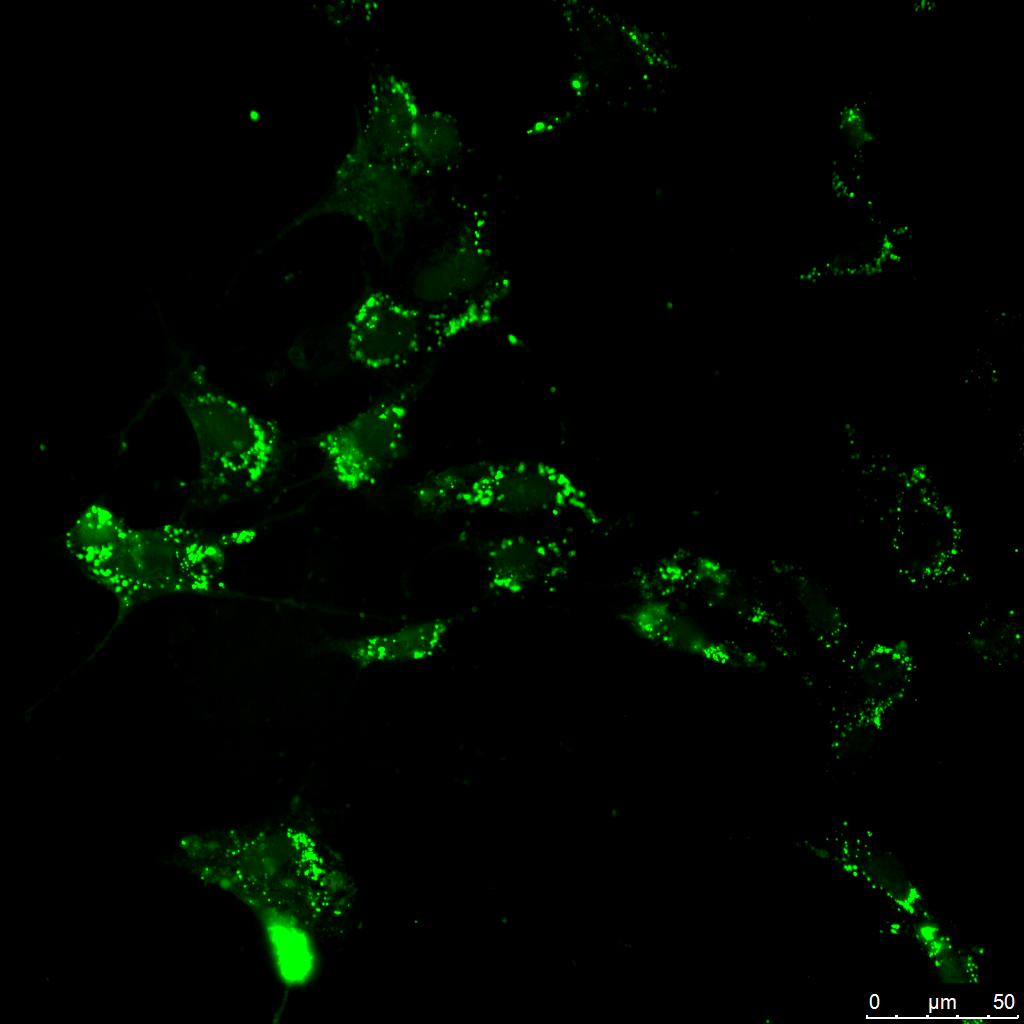

Supplement: Supplementary file 3 — Supplementary Data 1 [file 42003_2022_3856_MOESM3_ESM.zip › Supplementary Fig. 5/c/FLAG-CD44a-tv2+GFP-p53/cd44a2+p53-gfp.lif_Series007_ch01.tif]

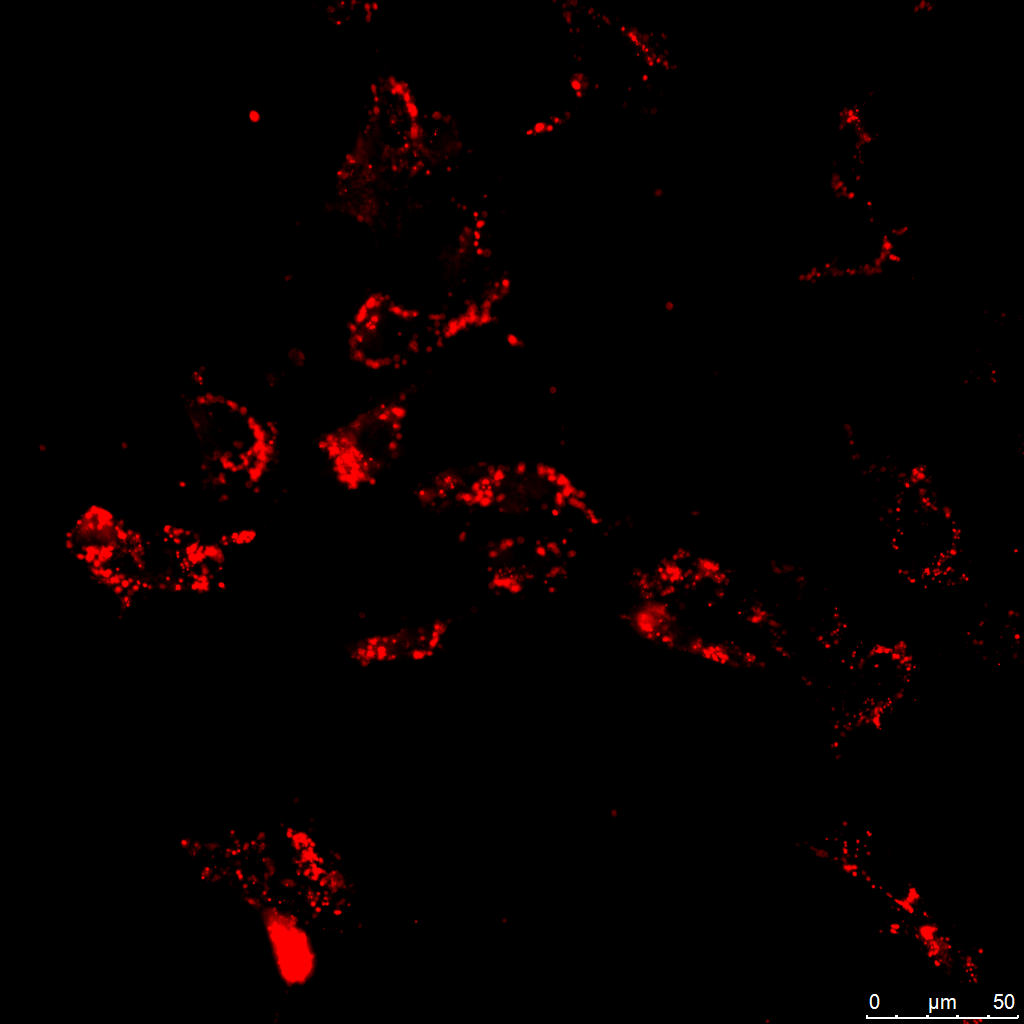

Supplement: Supplementary file 3 — Supplementary Data 1 [file 42003_2022_3856_MOESM3_ESM.zip › Supplementary Fig. 5/c/FLAG-CD44a-tv2+GFP-p53/cd44a2+p53-gfp.lif_Series007_ch02.tif]

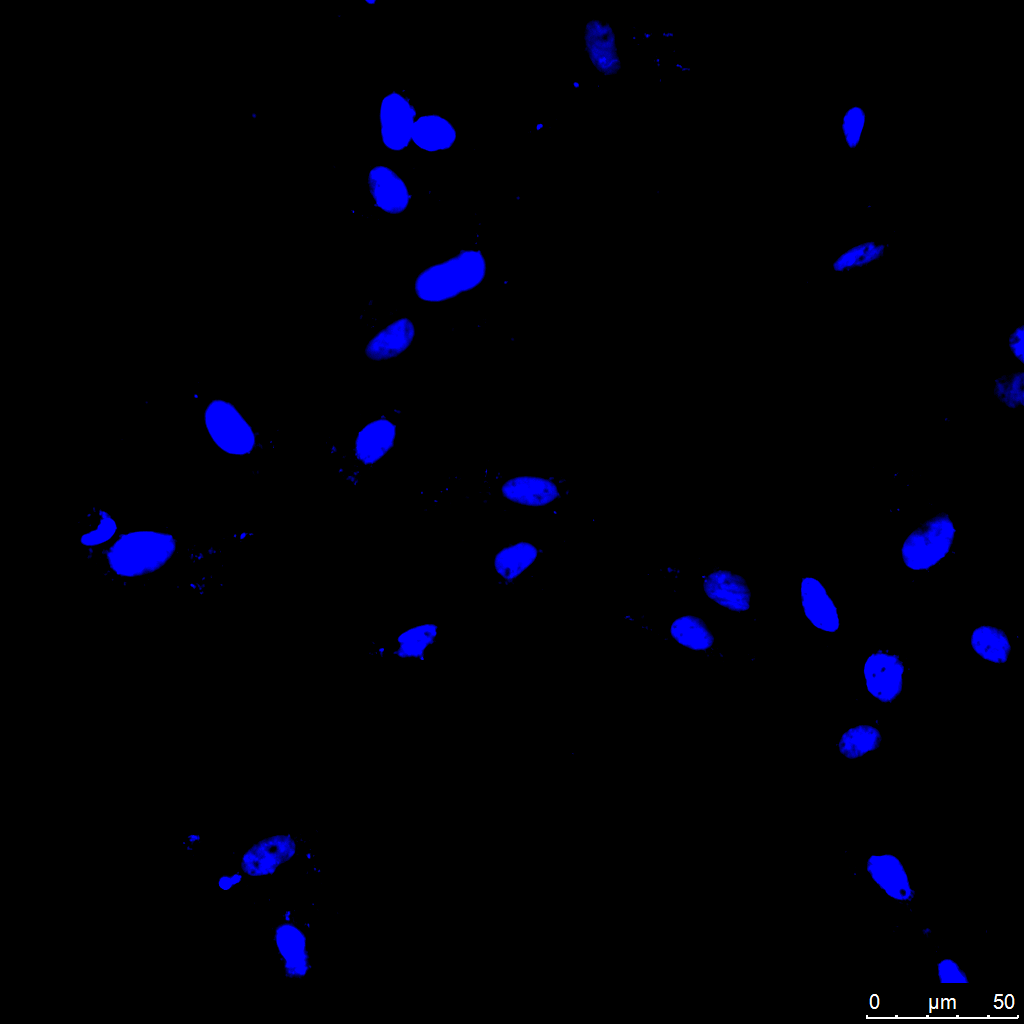

Supplement: Supplementary file 3 — Supplementary Data 1 [file 42003_2022_3856_MOESM3_ESM.zip › Supplementary Fig. 5/c/FLAG-CD44a-tv2+GFP-p53/cd44a2+p53-gfp.lif_Series007_ch03.tif]

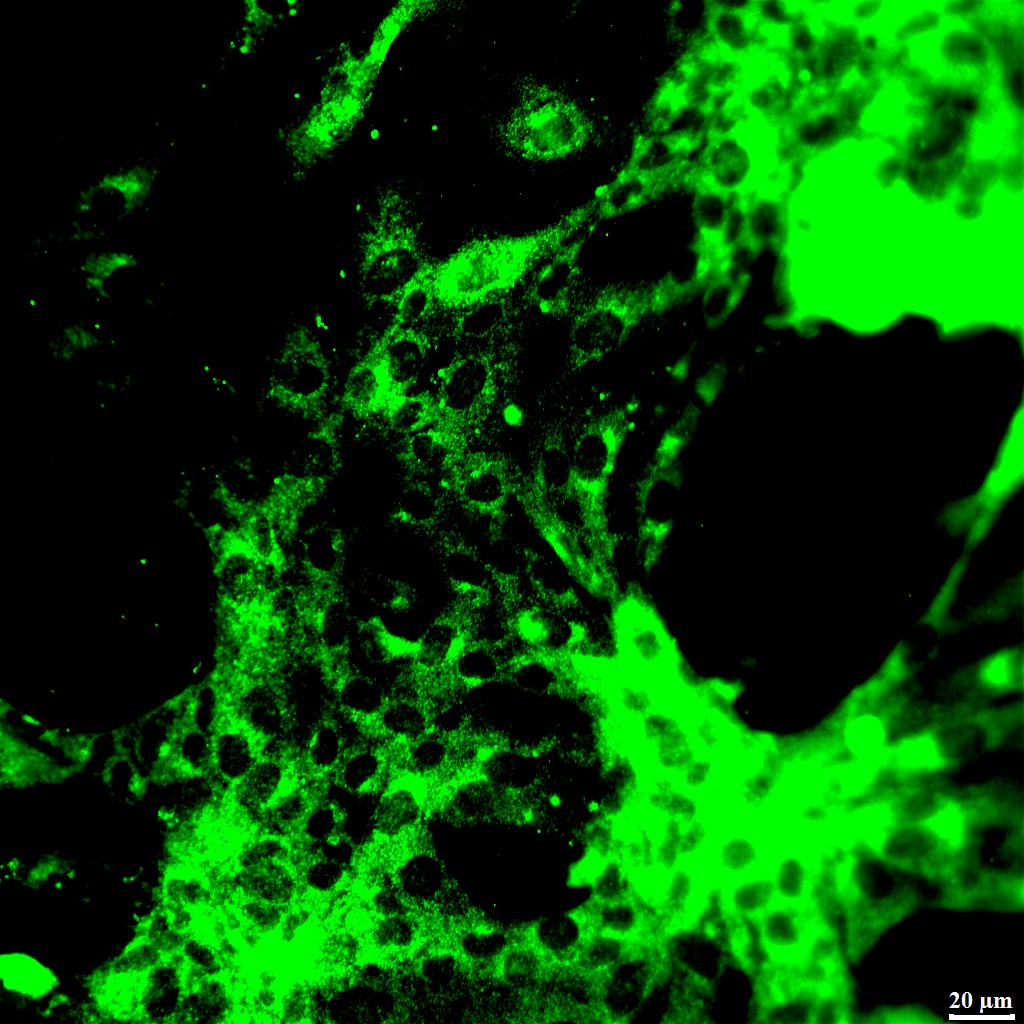

Supplement: Supplementary file 3 — Supplementary Data 1 [file 42003_2022_3856_MOESM3_ESM.zip › Supplementary Fig. 5/d/CD44a-tv1-FLAG/anti-p53.jpg]

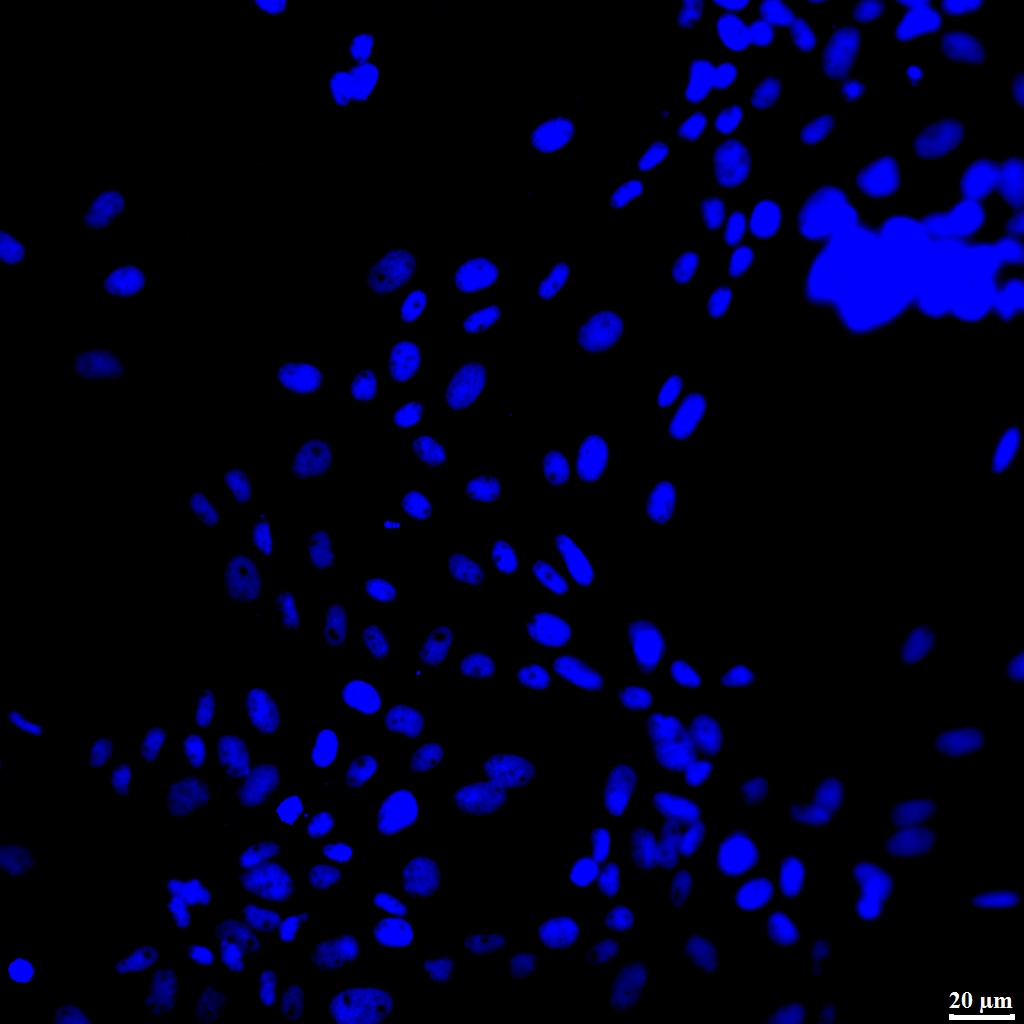

Supplement: Supplementary file 3 — Supplementary Data 1 [file 42003_2022_3856_MOESM3_ESM.zip › Supplementary Fig. 5/d/CD44a-tv1-FLAG/dapi.jpg]

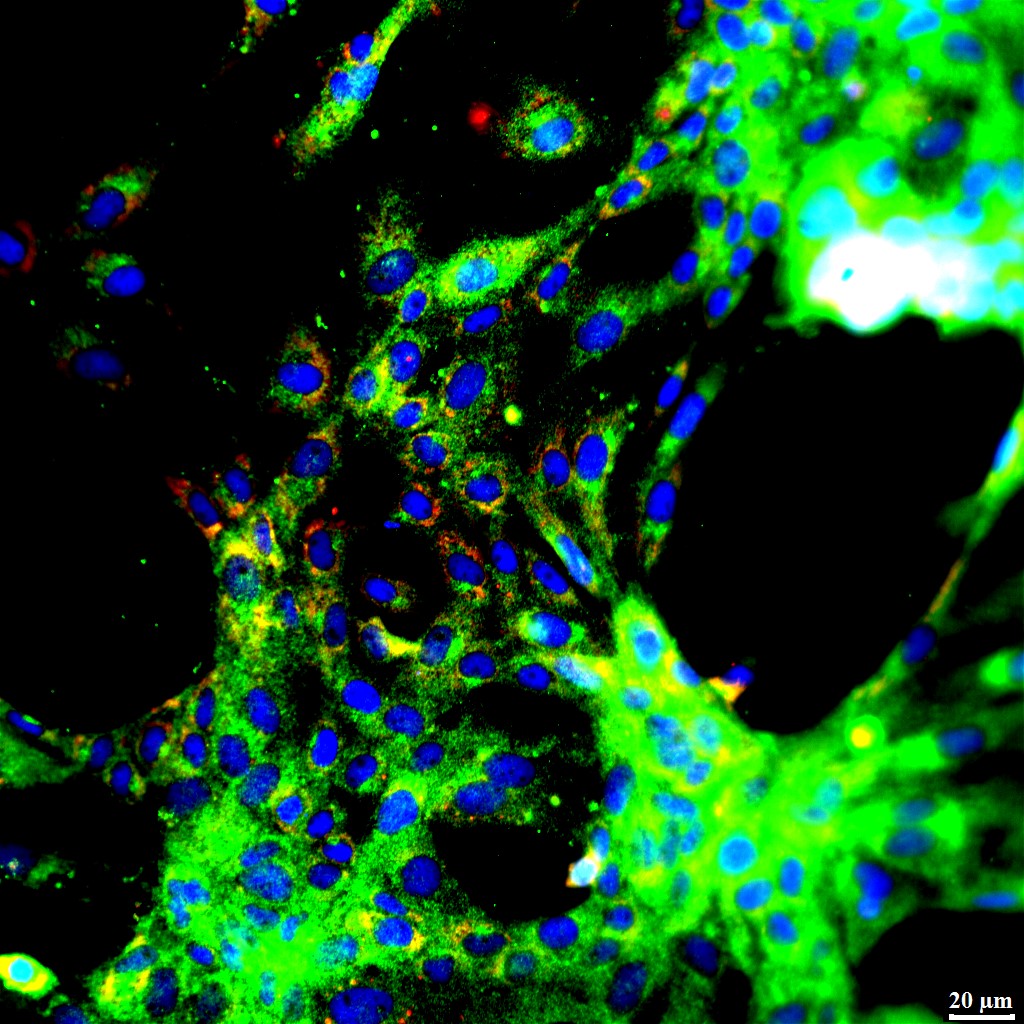

Supplement: Supplementary file 3 — Supplementary Data 1 [file 42003_2022_3856_MOESM3_ESM.zip › Supplementary Fig. 5/d/CD44a-tv1-FLAG/merge.jpg]

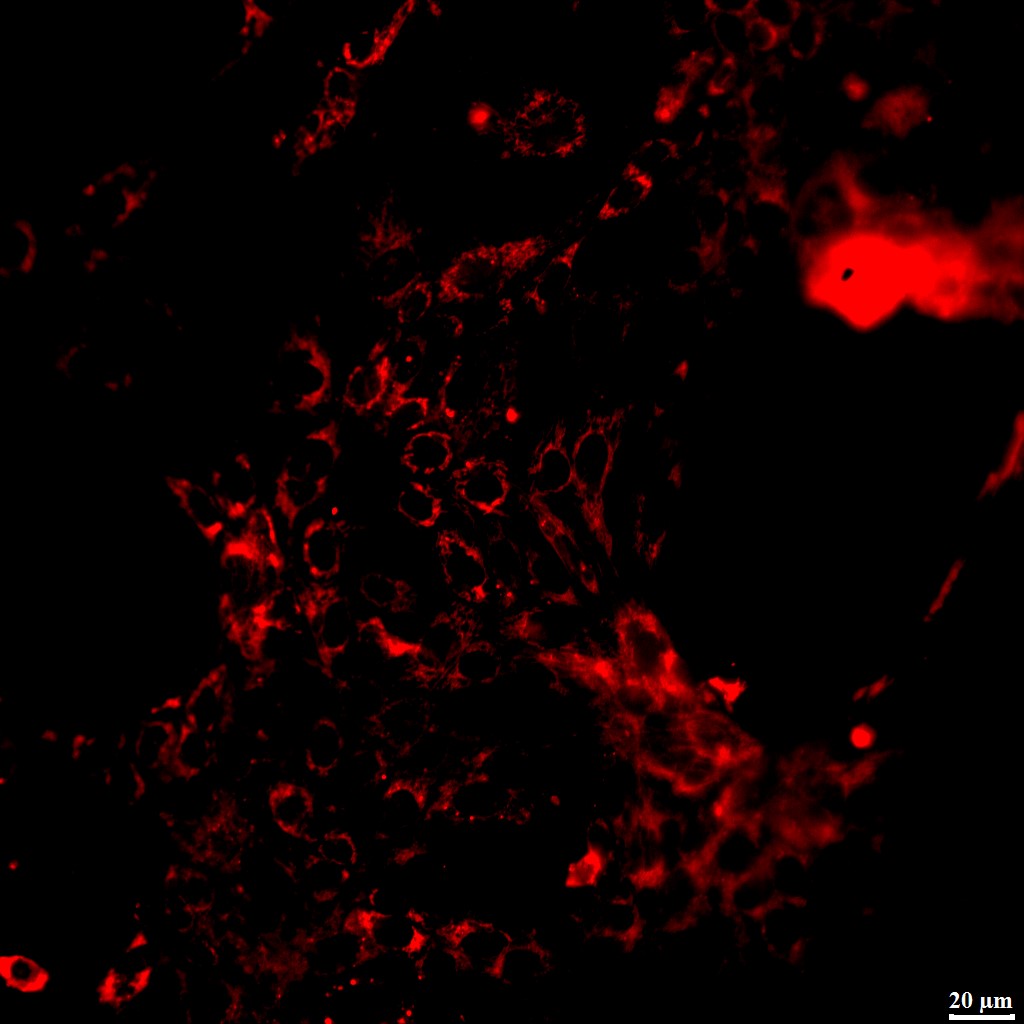

Supplement: Supplementary file 3 — Supplementary Data 1 [file 42003_2022_3856_MOESM3_ESM.zip › Supplementary Fig. 5/d/CD44a-tv1-FLAG/mito tracker.jpg]

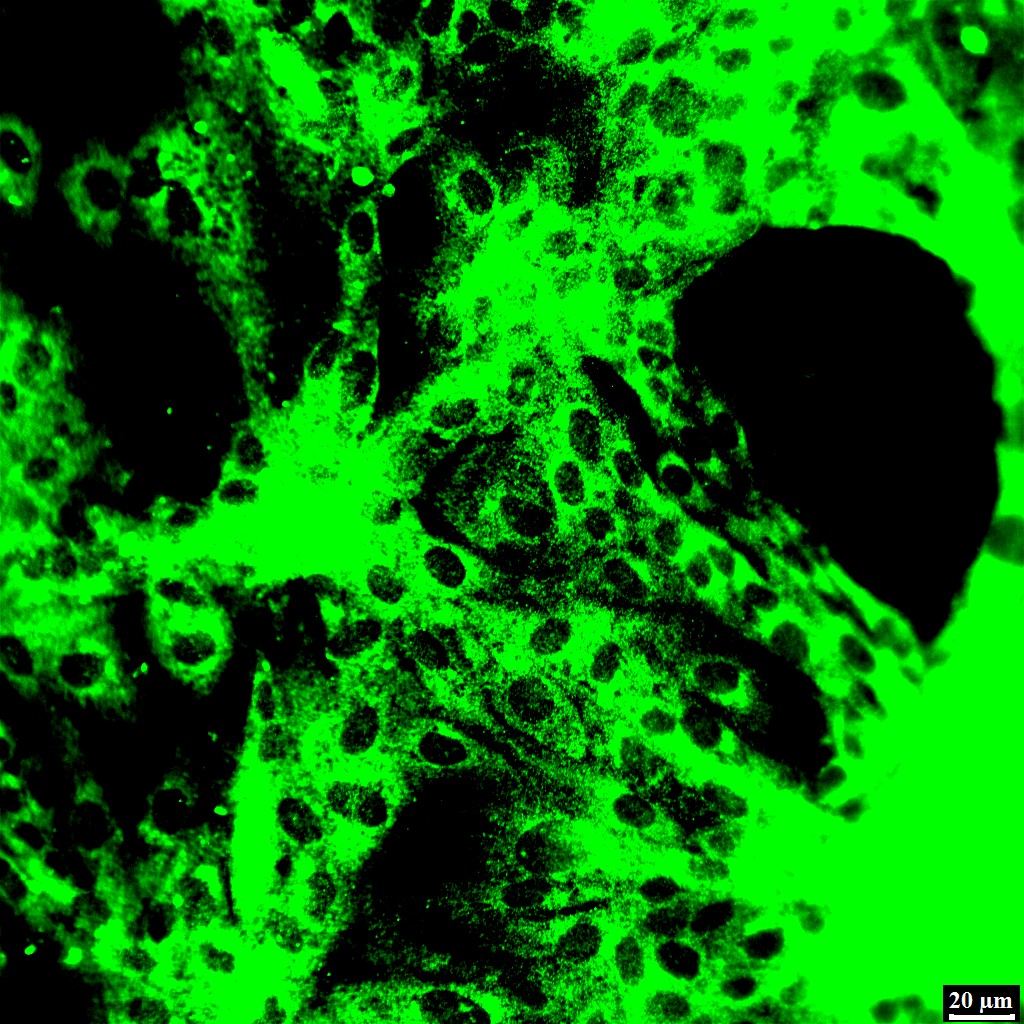

Supplement: Supplementary file 3 — Supplementary Data 1 [file 42003_2022_3856_MOESM3_ESM.zip › Supplementary Fig. 5/d/CD44a-tv2-FLAG/anti-p53.jpg]

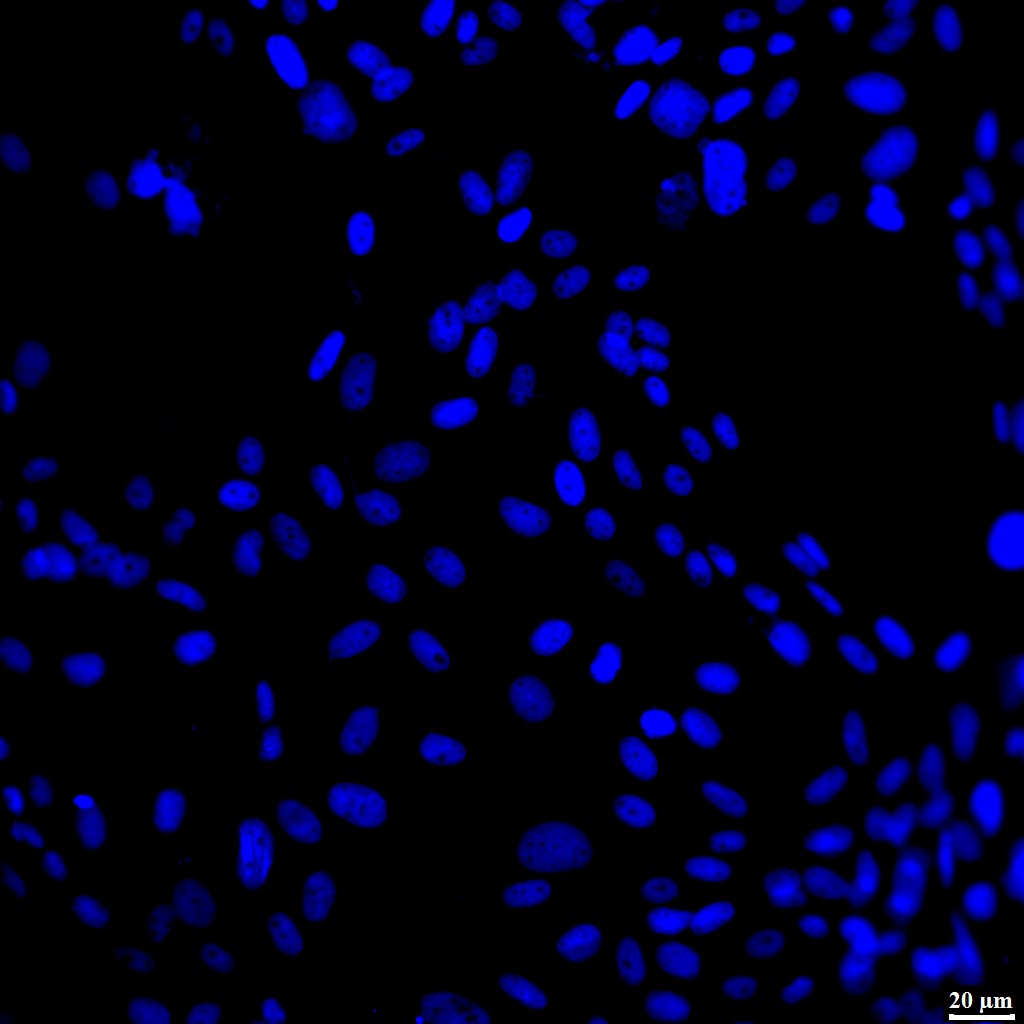

Supplement: Supplementary file 3 — Supplementary Data 1 [file 42003_2022_3856_MOESM3_ESM.zip › Supplementary Fig. 5/d/CD44a-tv2-FLAG/dapi.jpg]

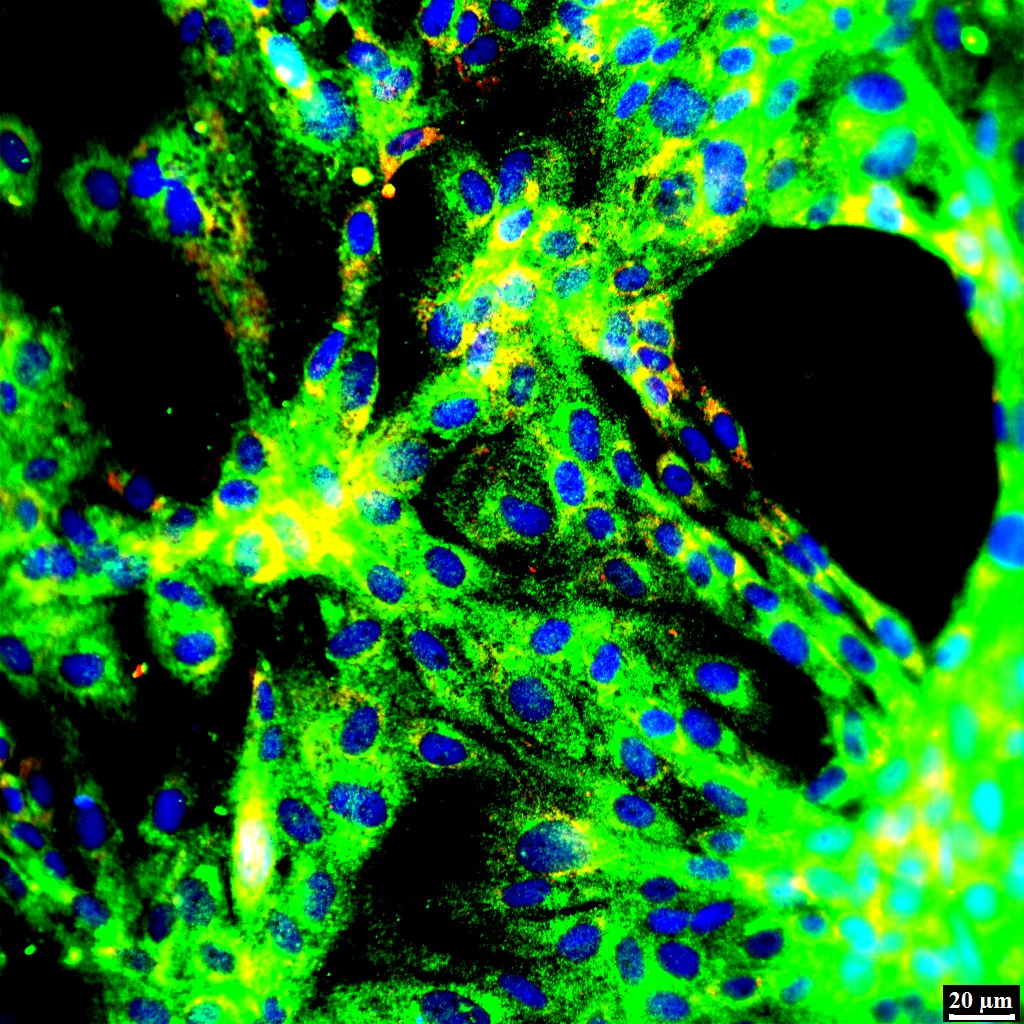

Supplement: Supplementary file 3 — Supplementary Data 1 [file 42003_2022_3856_MOESM3_ESM.zip › Supplementary Fig. 5/d/CD44a-tv2-FLAG/merge.jpg]

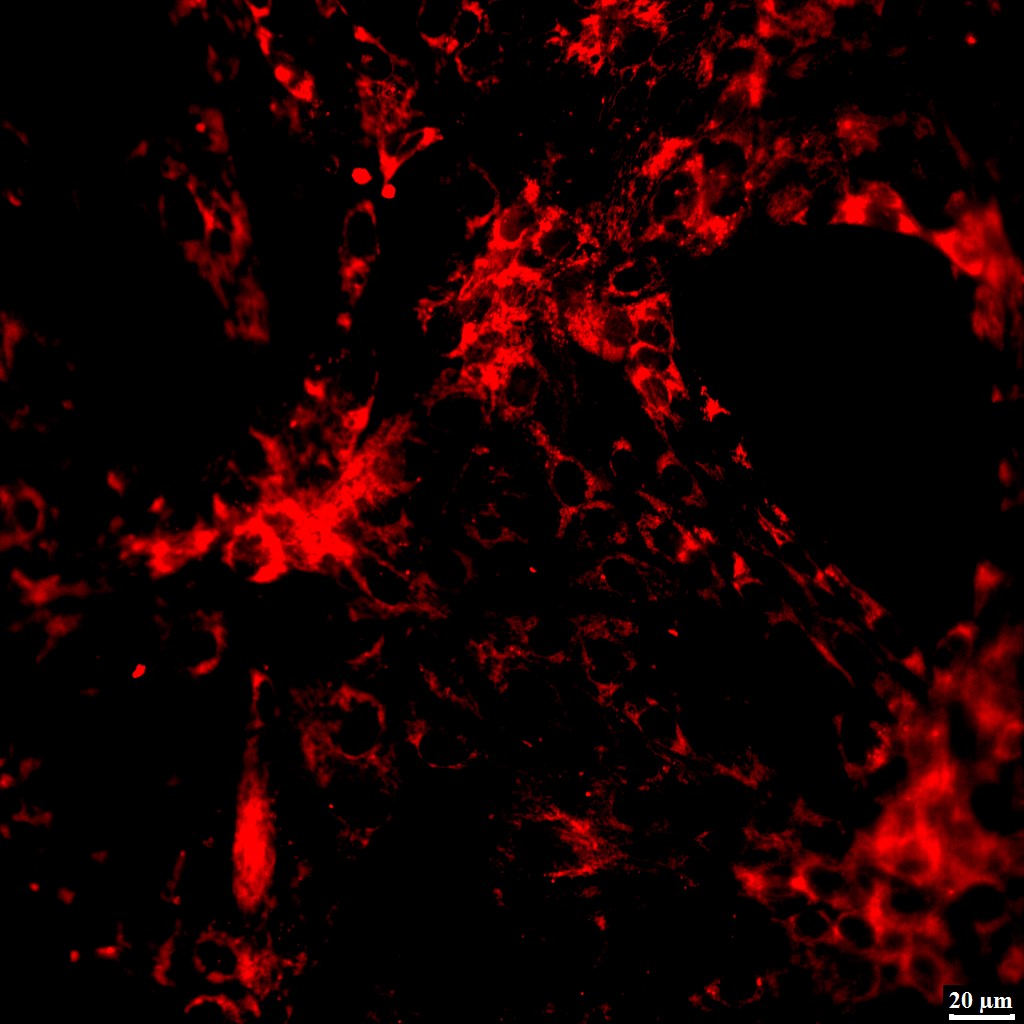

Supplement: Supplementary file 3 — Supplementary Data 1 [file 42003_2022_3856_MOESM3_ESM.zip › Supplementary Fig. 5/d/CD44a-tv2-FLAG/mito tracker.jpg]

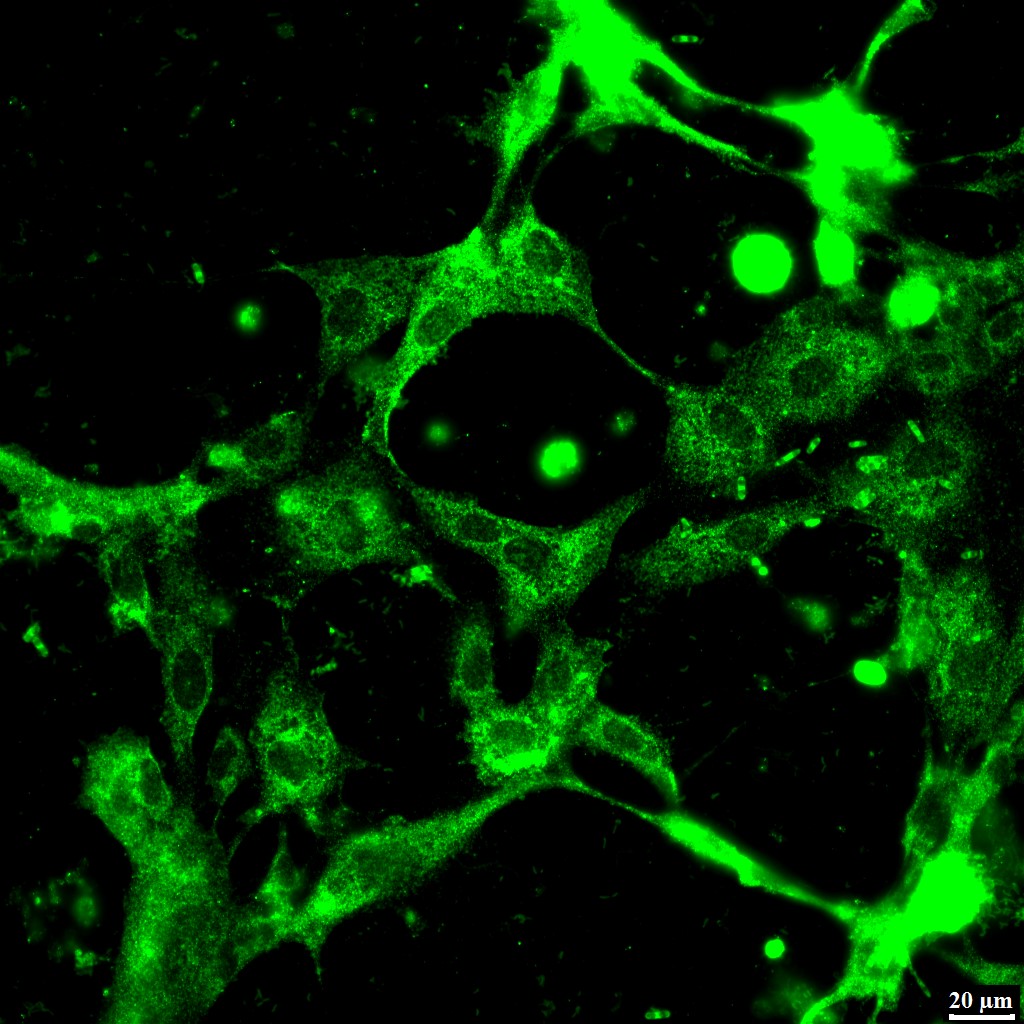

Supplement: Supplementary file 3 — Supplementary Data 1 [file 42003_2022_3856_MOESM3_ESM.zip › Supplementary Fig. 5/d/FLAG/anti-p53.jpg]

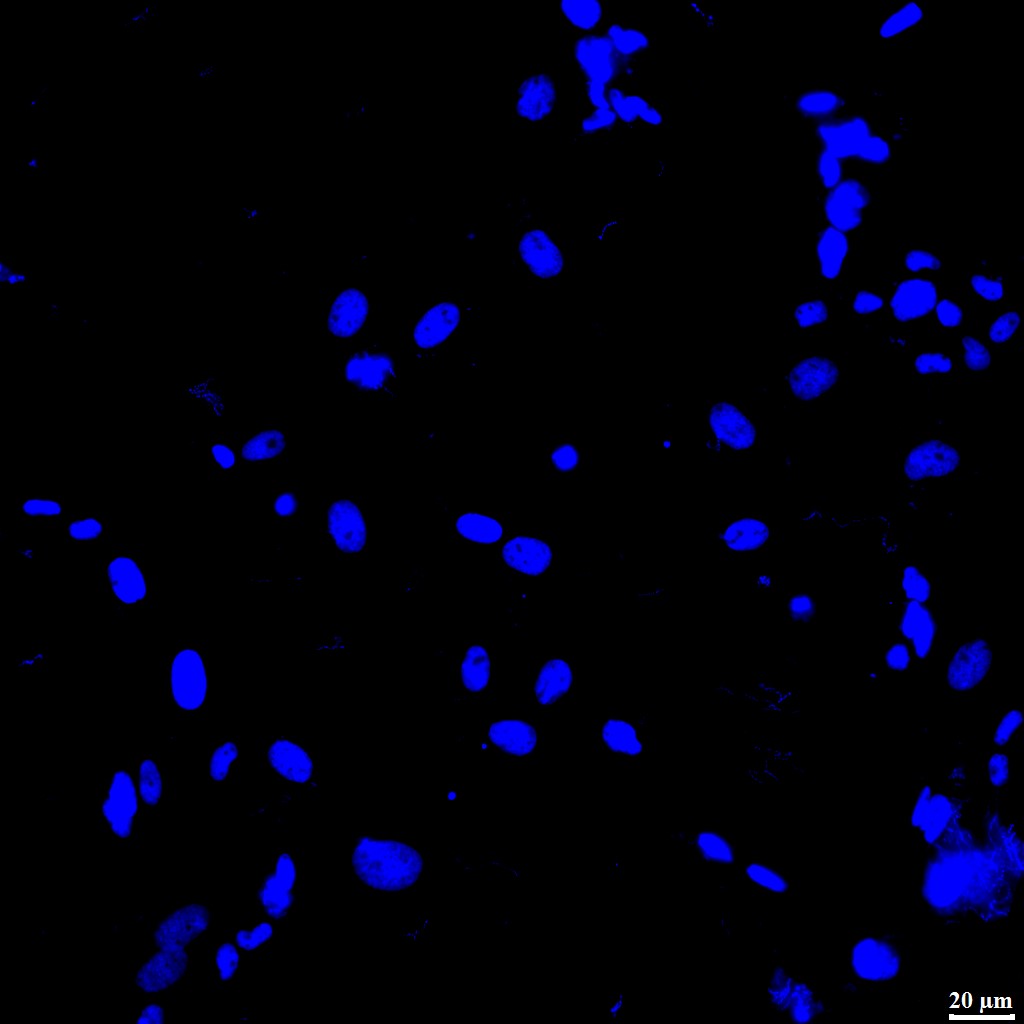

Supplement: Supplementary file 3 — Supplementary Data 1 [file 42003_2022_3856_MOESM3_ESM.zip › Supplementary Fig. 5/d/FLAG/dapi.jpg]

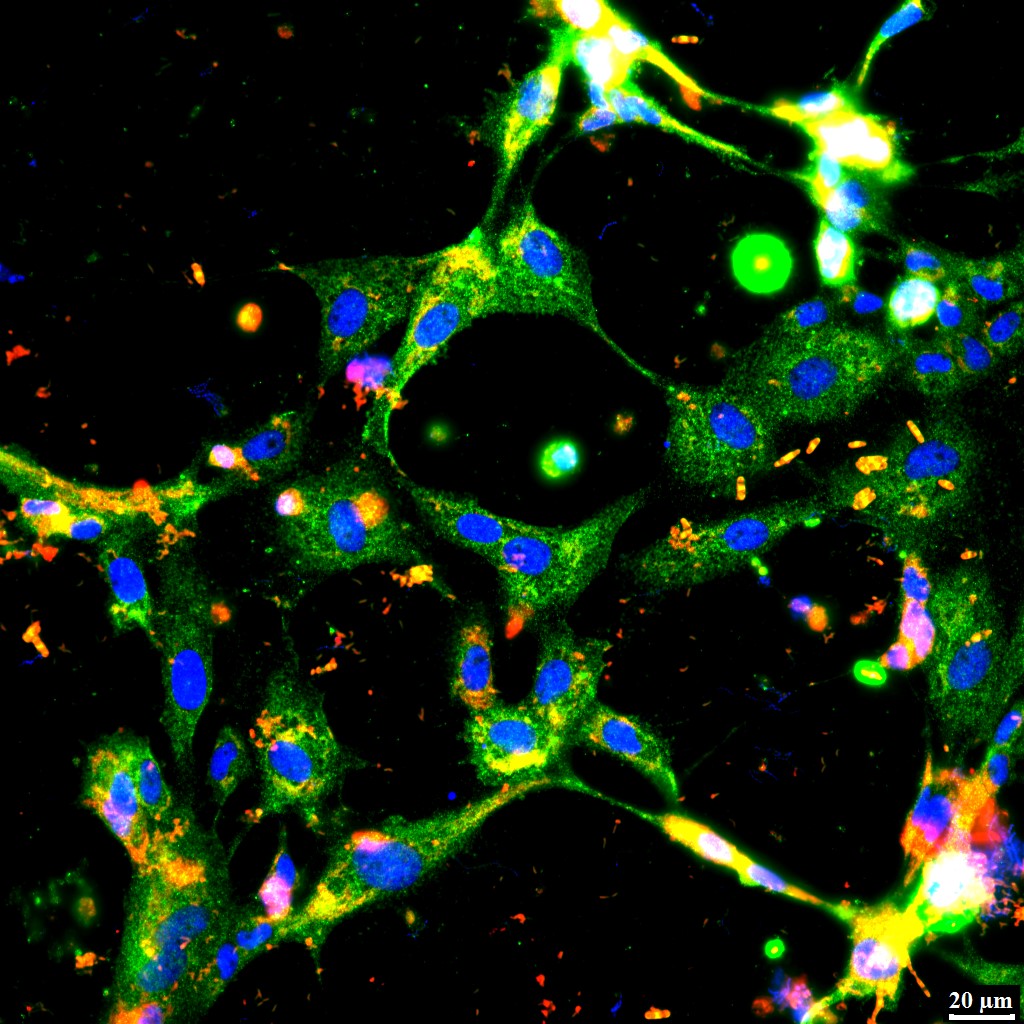

Supplement: Supplementary file 3 — Supplementary Data 1 [file 42003_2022_3856_MOESM3_ESM.zip › Supplementary Fig. 5/d/FLAG/merge.jpg]

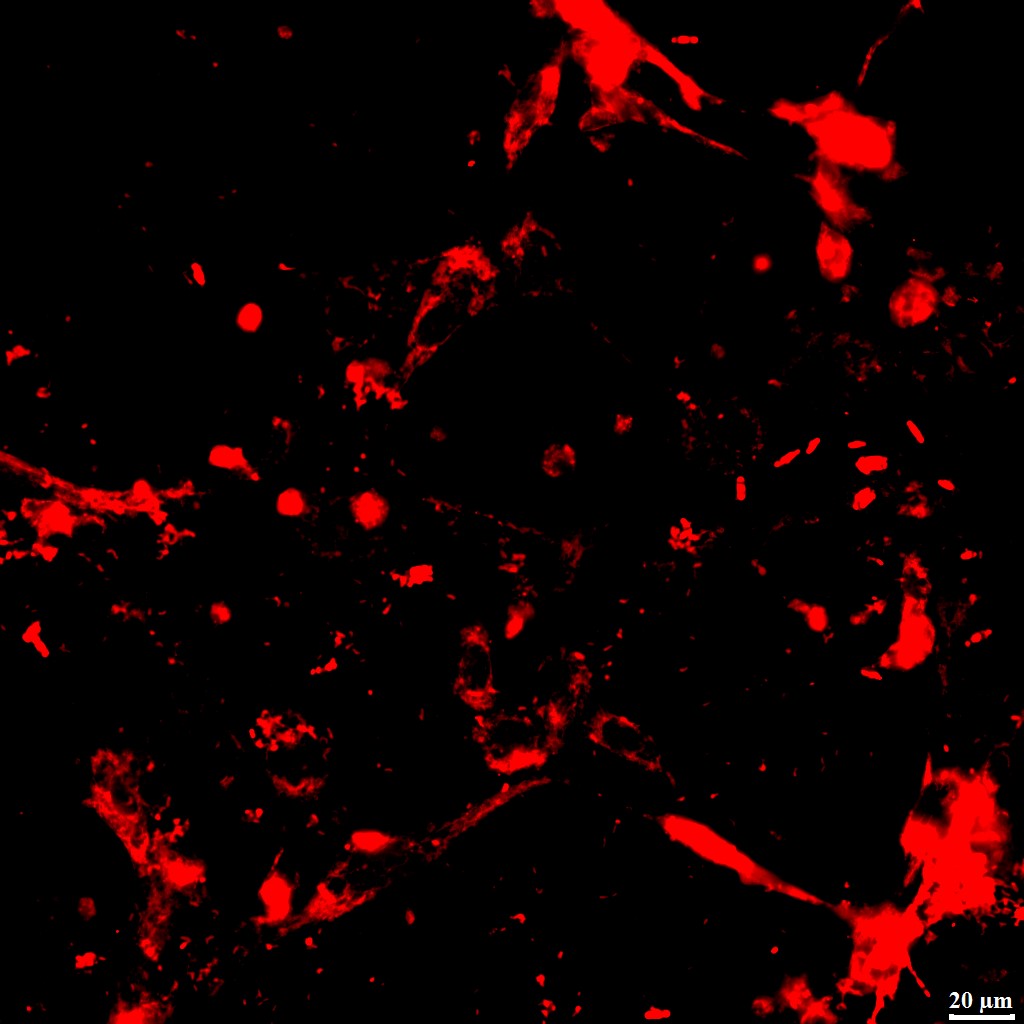

Supplement: Supplementary file 3 — Supplementary Data 1 [file 42003_2022_3856_MOESM3_ESM.zip › Supplementary Fig. 5/d/FLAG/mito tracker.jpg]

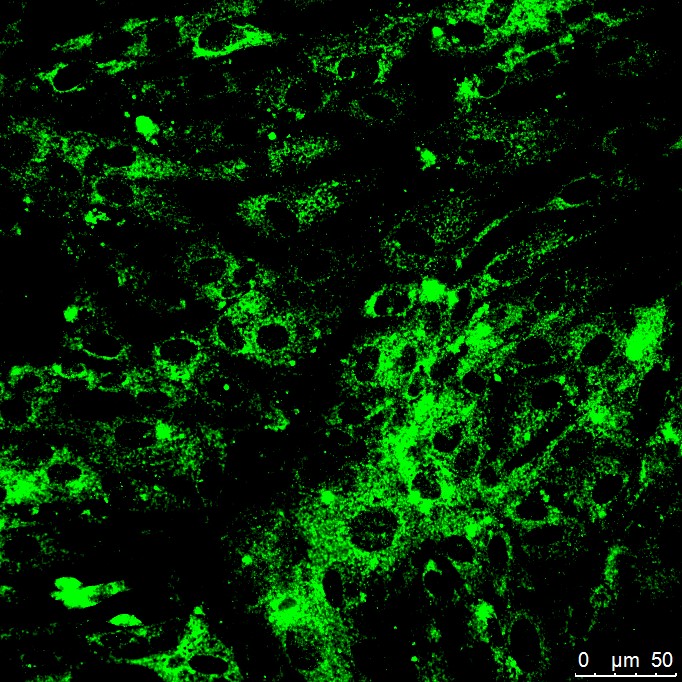

Supplement: Supplementary file 3 — Supplementary Data 1 [file 42003_2022_3856_MOESM3_ESM.zip › Supplementary Fig. 5/e/CD44a-tv1-FLAG/anti-p53.jpg]

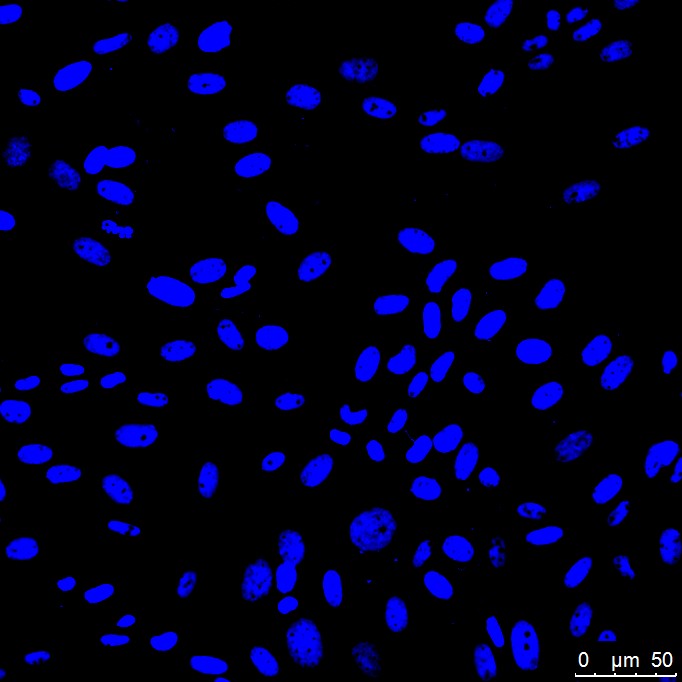

Supplement: Supplementary file 3 — Supplementary Data 1 [file 42003_2022_3856_MOESM3_ESM.zip › Supplementary Fig. 5/e/CD44a-tv1-FLAG/dapi.jpg]

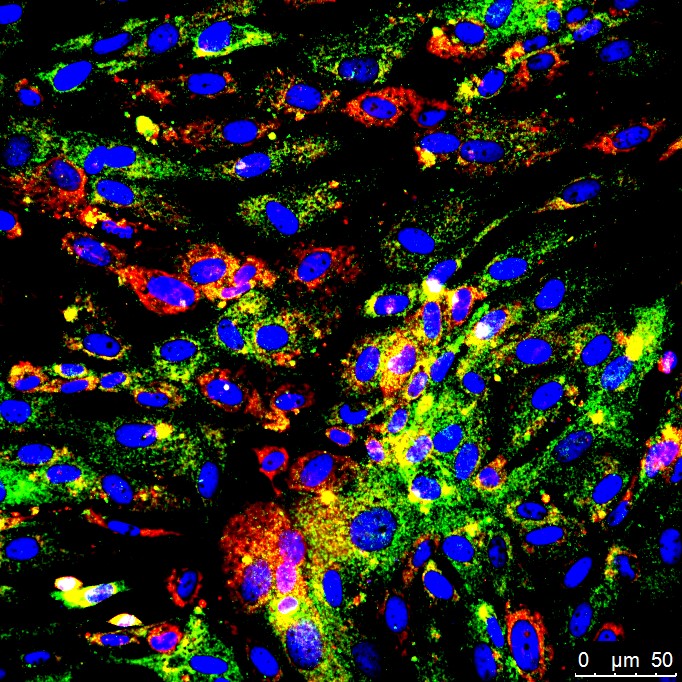

Supplement: Supplementary file 3 — Supplementary Data 1 [file 42003_2022_3856_MOESM3_ESM.zip › Supplementary Fig. 5/e/CD44a-tv1-FLAG/merge.jpg]

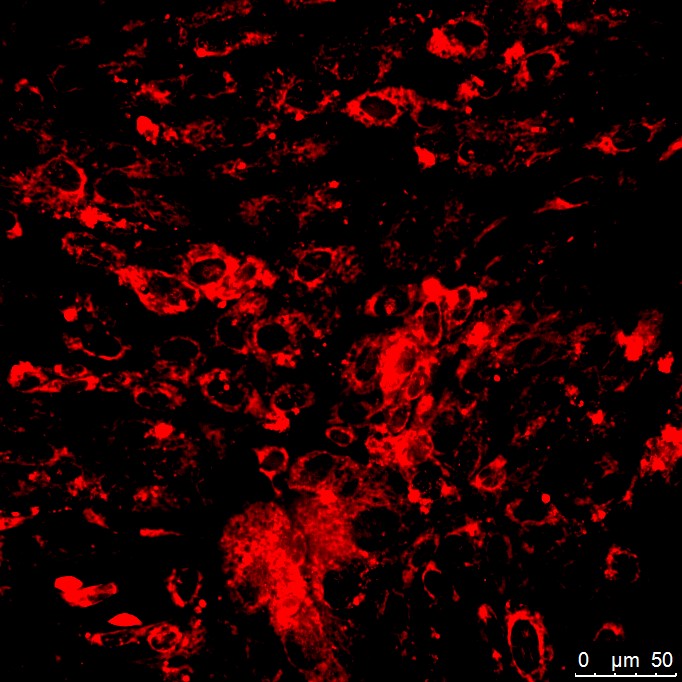

Supplement: Supplementary file 3 — Supplementary Data 1 [file 42003_2022_3856_MOESM3_ESM.zip › Supplementary Fig. 5/e/CD44a-tv1-FLAG/mito tracker.jpg]

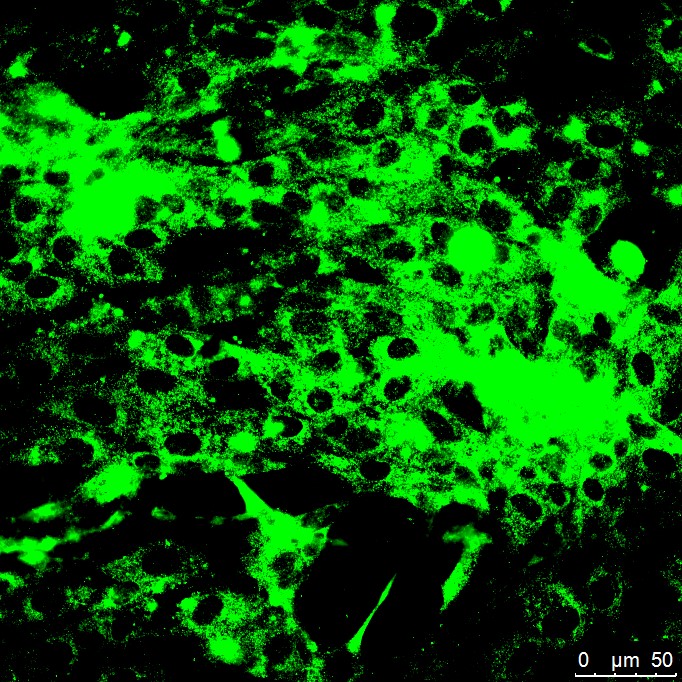

Supplement: Supplementary file 3 — Supplementary Data 1 [file 42003_2022_3856_MOESM3_ESM.zip › Supplementary Fig. 5/e/CD44a-tv2-FLAG/anti-p53.jpg]

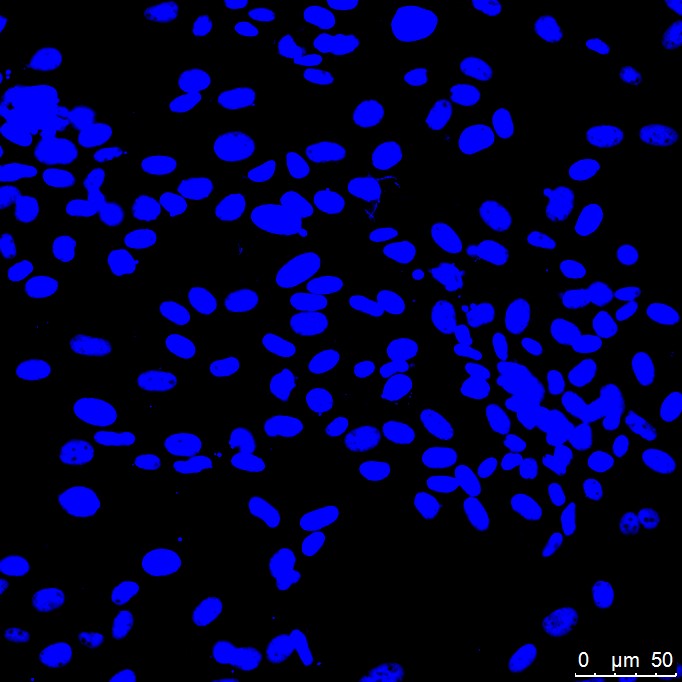

Supplement: Supplementary file 3 — Supplementary Data 1 [file 42003_2022_3856_MOESM3_ESM.zip › Supplementary Fig. 5/e/CD44a-tv2-FLAG/dapi.jpg]

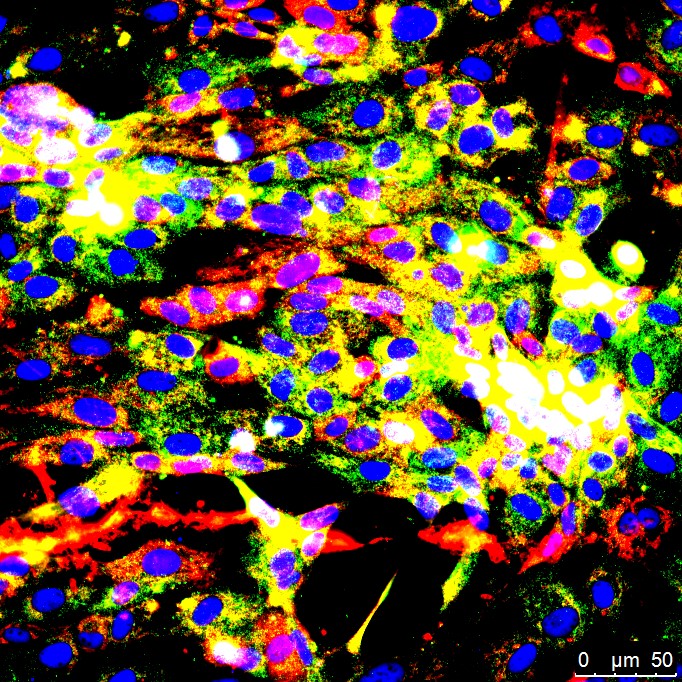

Supplement: Supplementary file 3 — Supplementary Data 1 [file 42003_2022_3856_MOESM3_ESM.zip › Supplementary Fig. 5/e/CD44a-tv2-FLAG/merge.jpg]

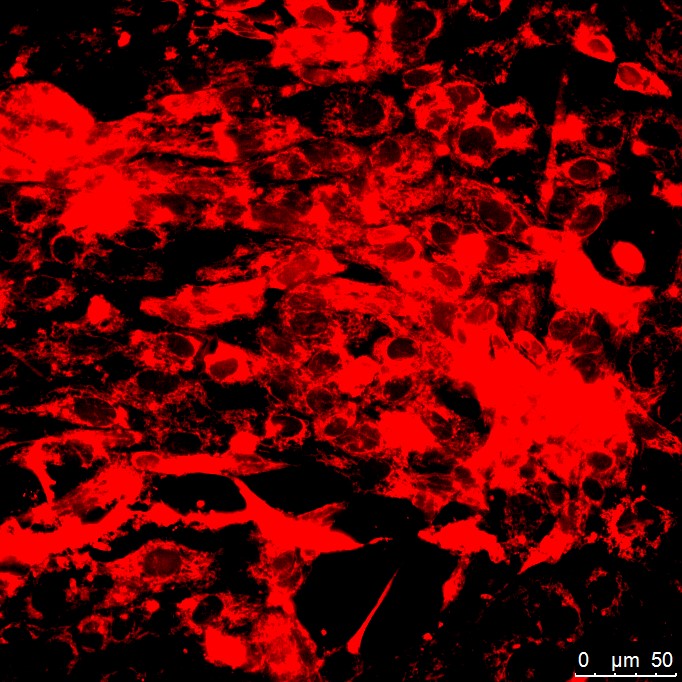

Supplement: Supplementary file 3 — Supplementary Data 1 [file 42003_2022_3856_MOESM3_ESM.zip › Supplementary Fig. 5/e/CD44a-tv2-FLAG/mito tracker.jpg]

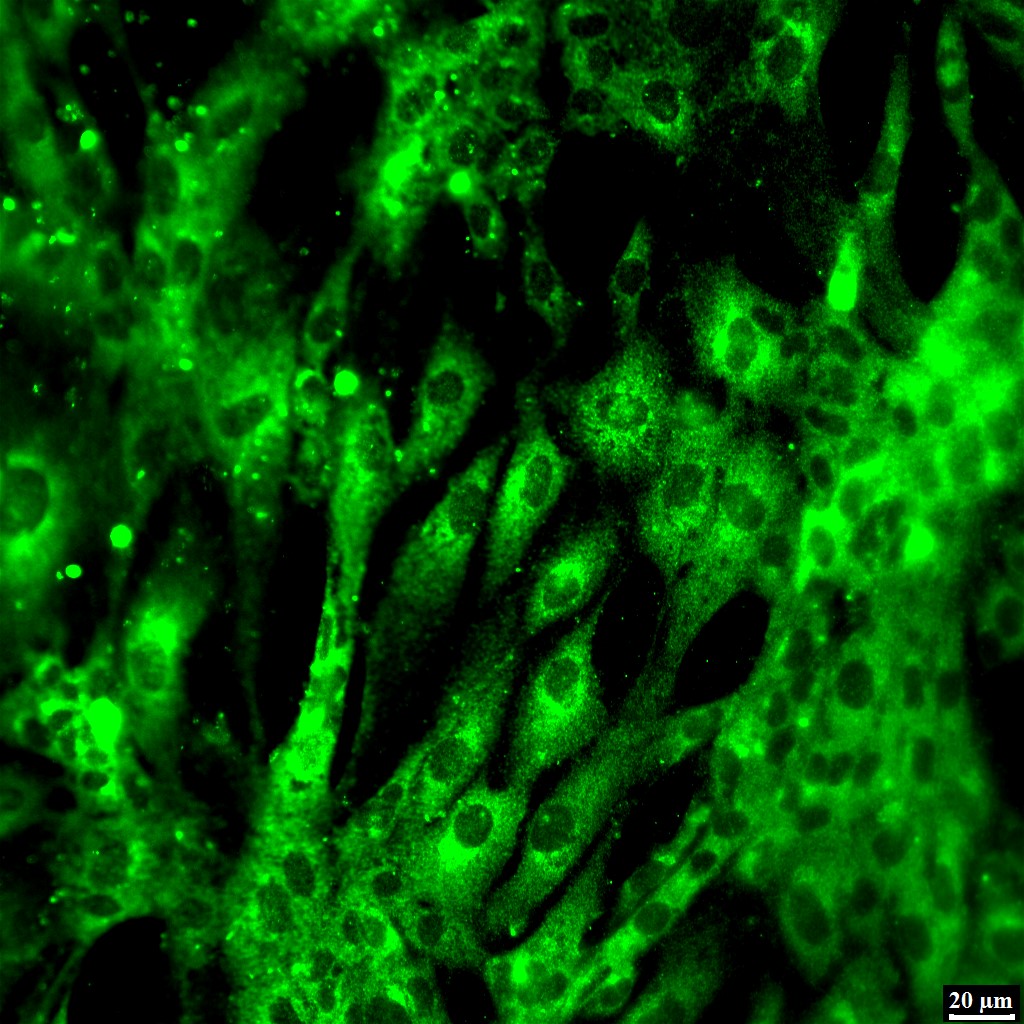

Supplement: Supplementary file 3 — Supplementary Data 1 [file 42003_2022_3856_MOESM3_ESM.zip › Supplementary Fig. 5/e/FLAG/anti-p53.jpg]

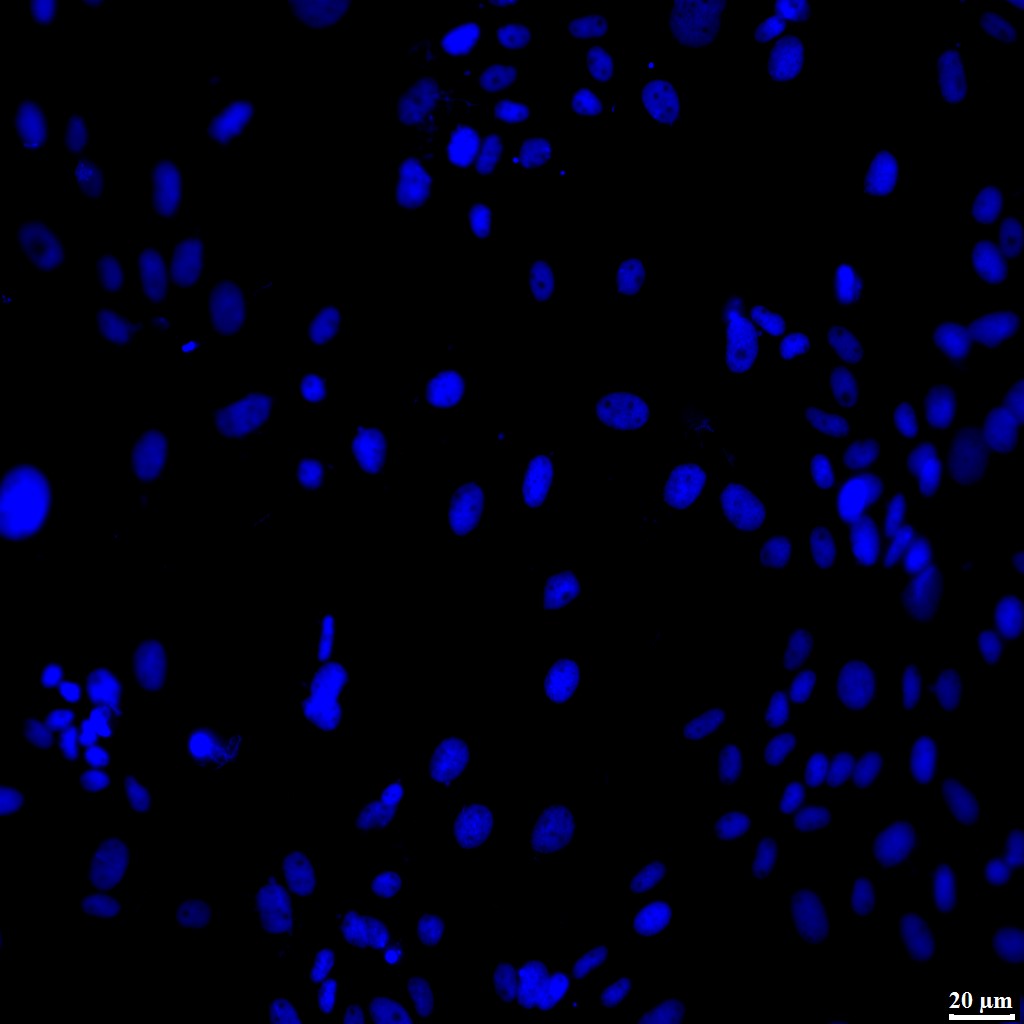

Supplement: Supplementary file 3 — Supplementary Data 1 [file 42003_2022_3856_MOESM3_ESM.zip › Supplementary Fig. 5/e/FLAG/dapi.jpg]

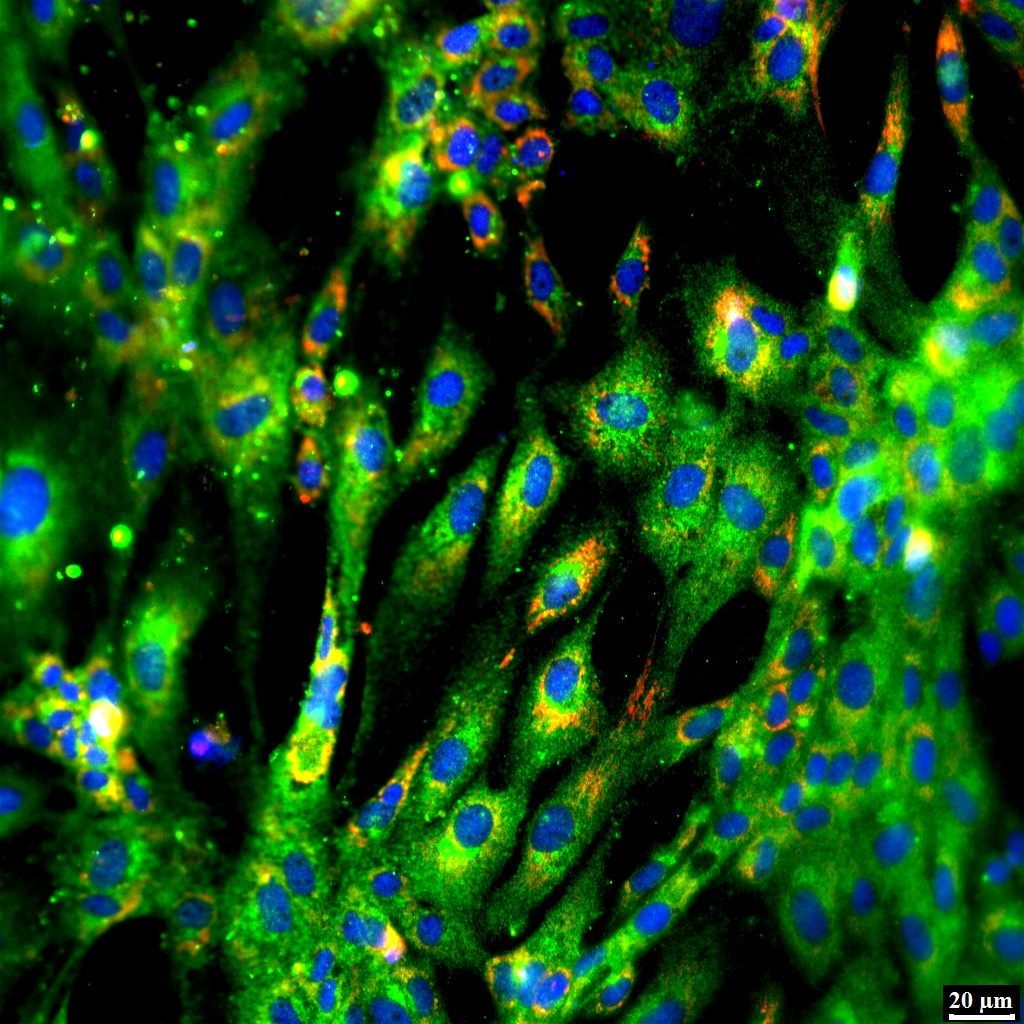

Supplement: Supplementary file 3 — Supplementary Data 1 [file 42003_2022_3856_MOESM3_ESM.zip › Supplementary Fig. 5/e/FLAG/merge.jpg]

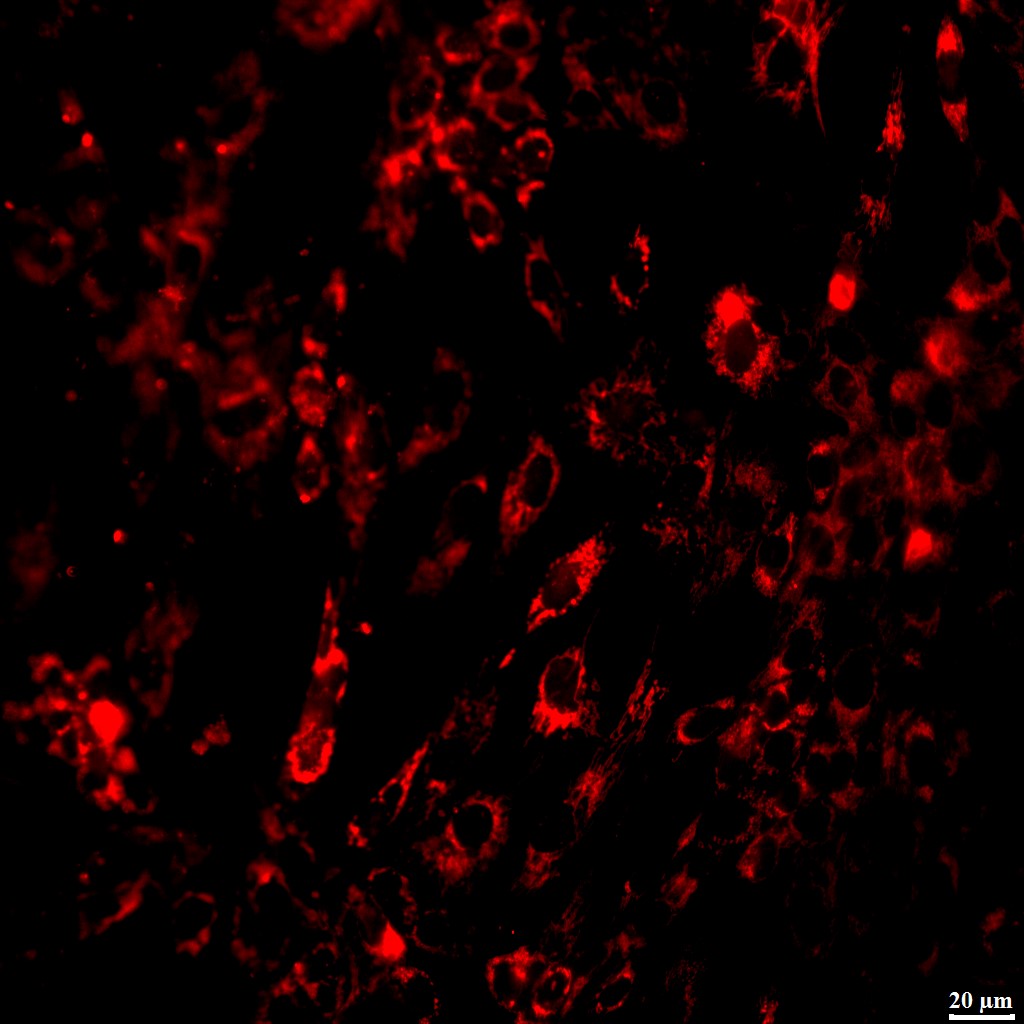

Supplement: Supplementary file 3 — Supplementary Data 1 [file 42003_2022_3856_MOESM3_ESM.zip › Supplementary Fig. 5/e/FLAG/mito tracker.jpg]

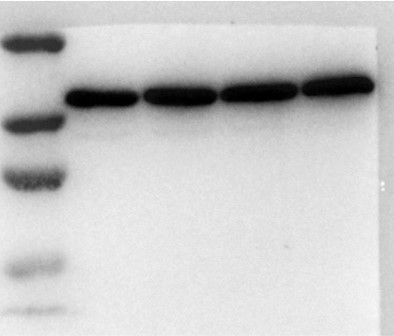

Supplement: Supplementary file 4 — Supplementary Data 2 [file 42003_2022_3856_MOESM4_ESM.zip › Fig. 4c/GAPDH.jpg]

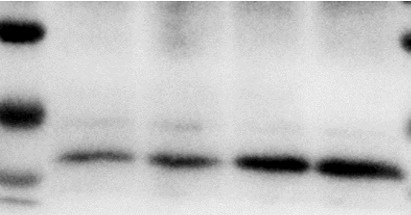

Supplement: Supplementary file 4 — Supplementary Data 2 [file 42003_2022_3856_MOESM4_ESM.zip › Fig. 4c/LC3b.jpg]

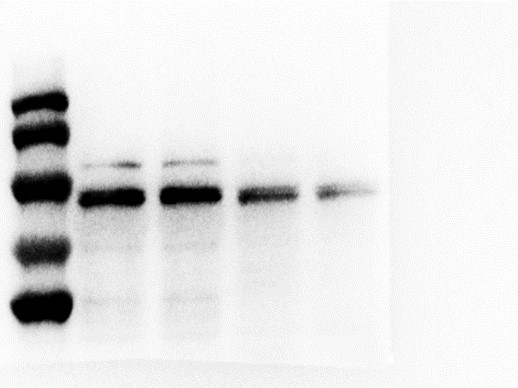

Supplement: Supplementary file 4 — Supplementary Data 2 [file 42003_2022_3856_MOESM4_ESM.zip › Fig. 4c/p62.jpg]

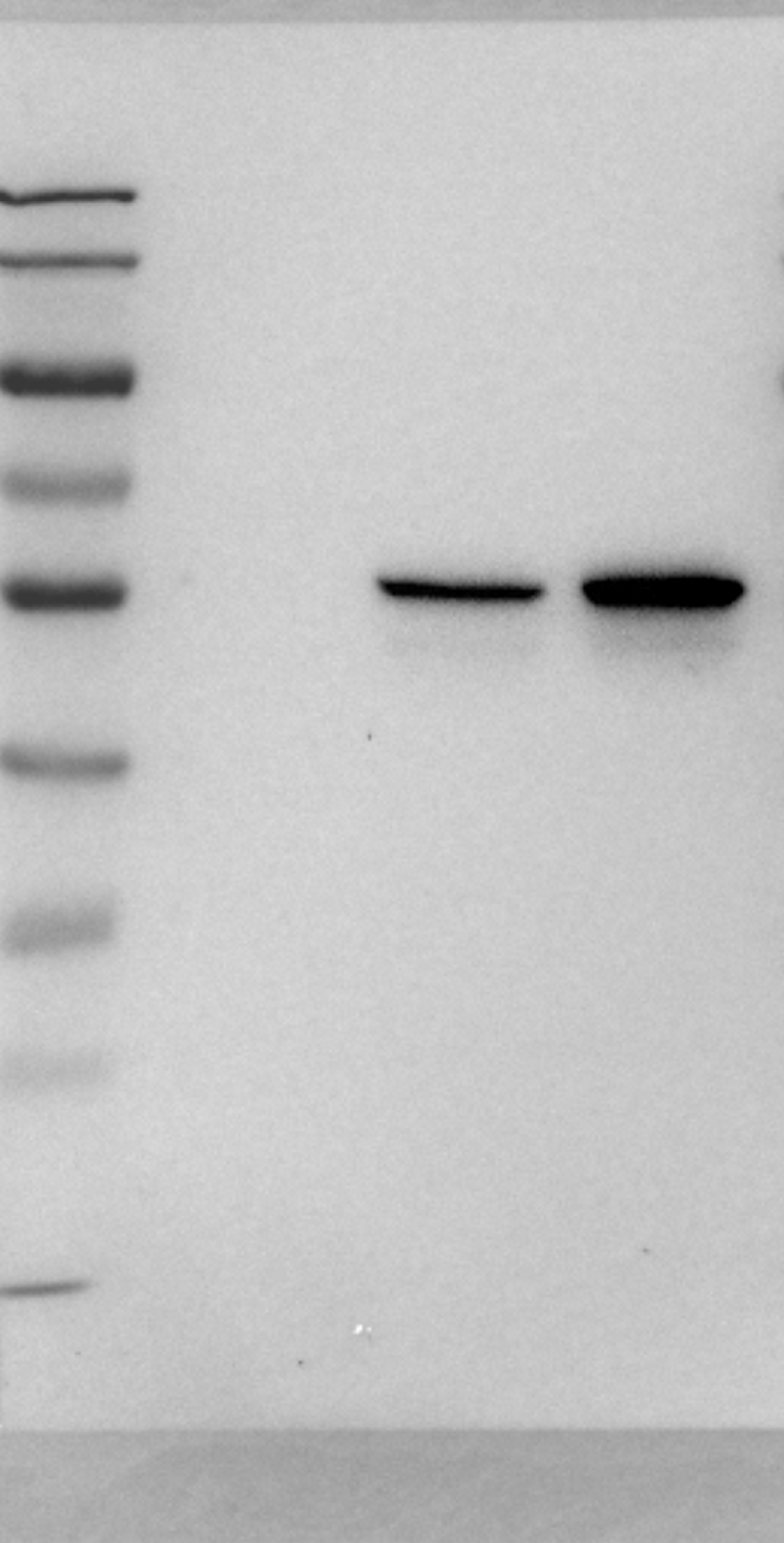

Supplement: Supplementary file 4 — Supplementary Data 2 [file 42003_2022_3856_MOESM4_ESM.zip › Fig. 6a/anti-flag-p53-1.tif]

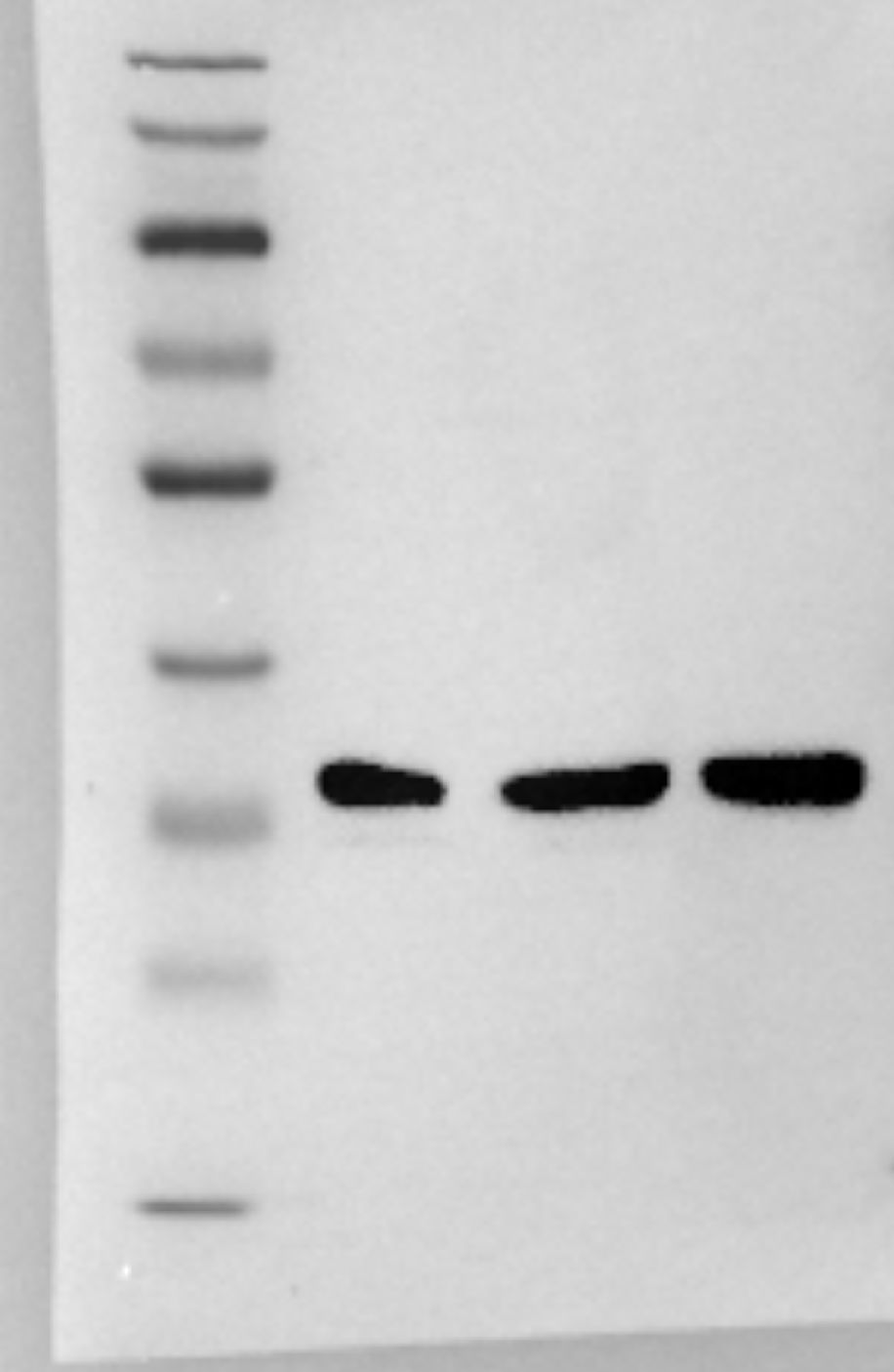

Supplement: Supplementary file 4 — Supplementary Data 2 [file 42003_2022_3856_MOESM4_ESM.zip › Fig. 6a/anti-GAPDH (1).tif]

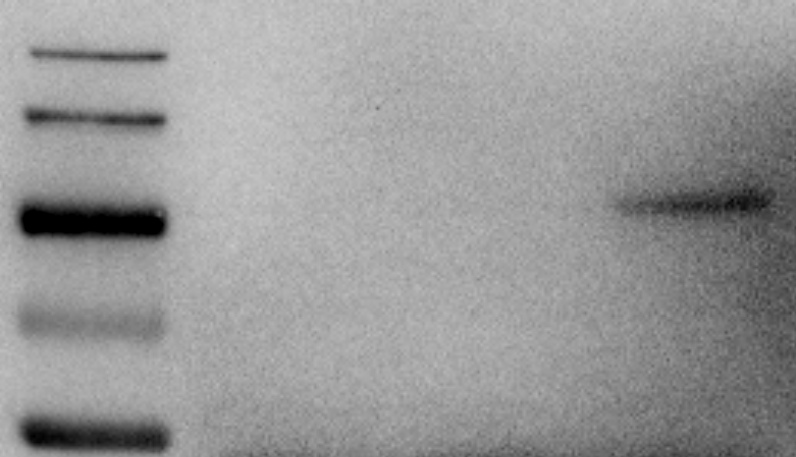

Supplement: Supplementary file 4 — Supplementary Data 2 [file 42003_2022_3856_MOESM4_ESM.zip › Fig. 6a/anti-GFP-CD44a1.tif]

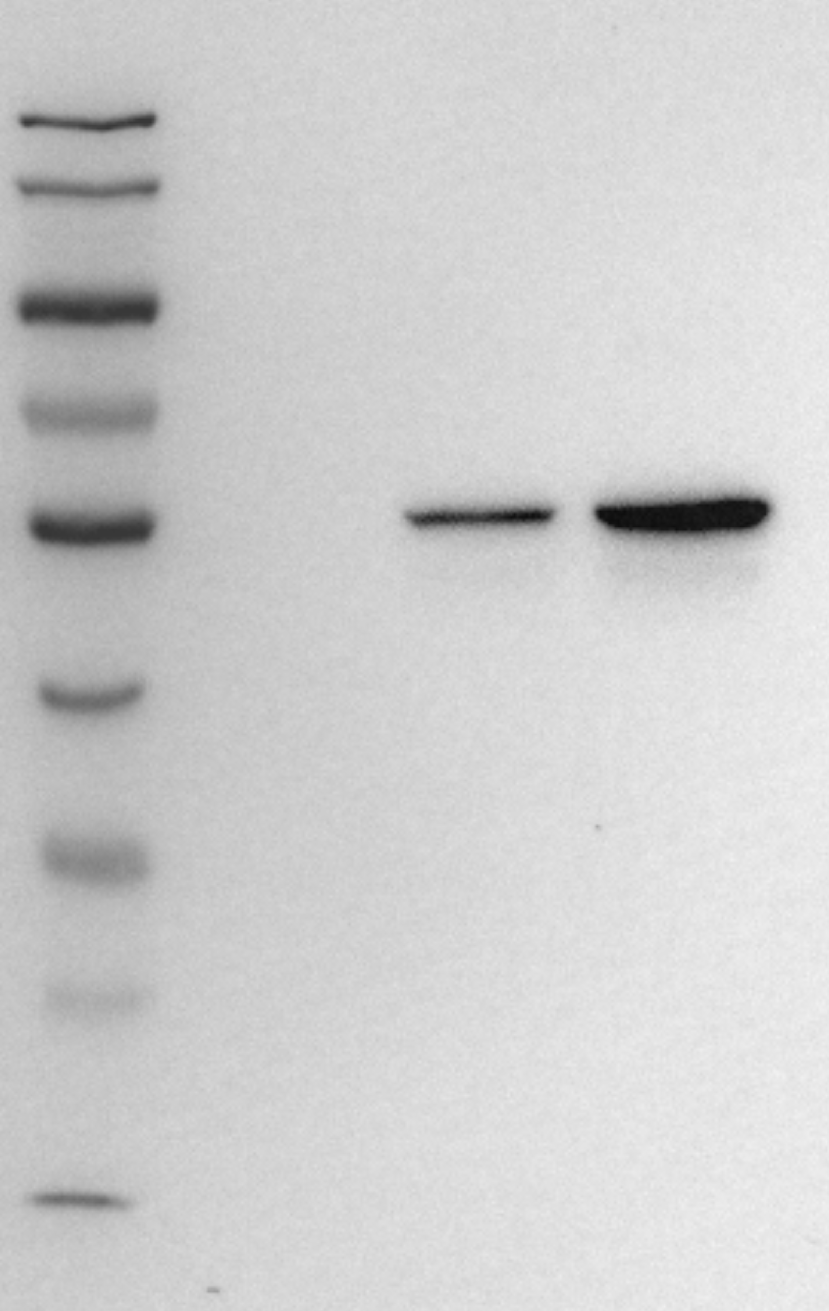

Supplement: Supplementary file 4 — Supplementary Data 2 [file 42003_2022_3856_MOESM4_ESM.zip › Fig. 6b/anti-flag-p53.tif]

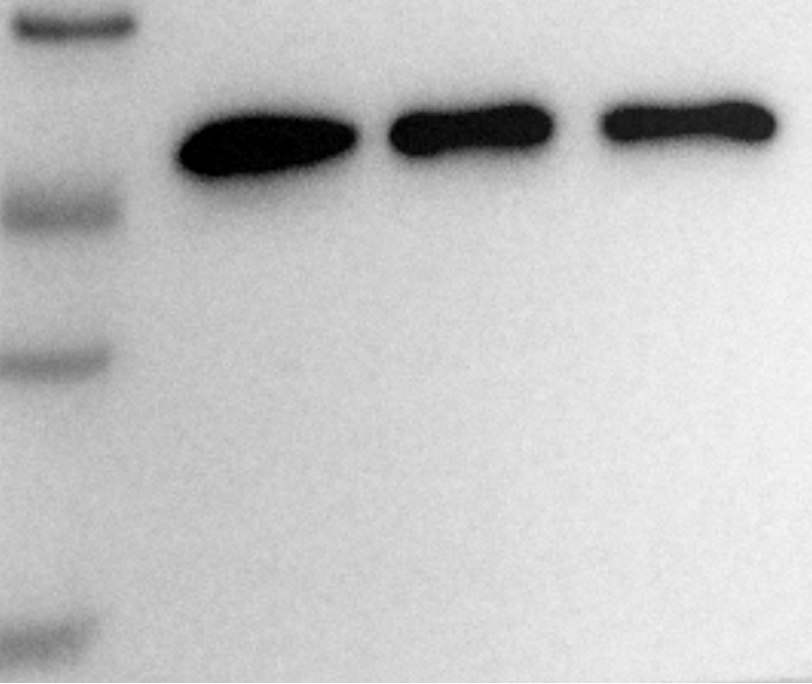

Supplement: Supplementary file 4 — Supplementary Data 2 [file 42003_2022_3856_MOESM4_ESM.zip › Fig. 6b/anti-GAPDH-2.tif]

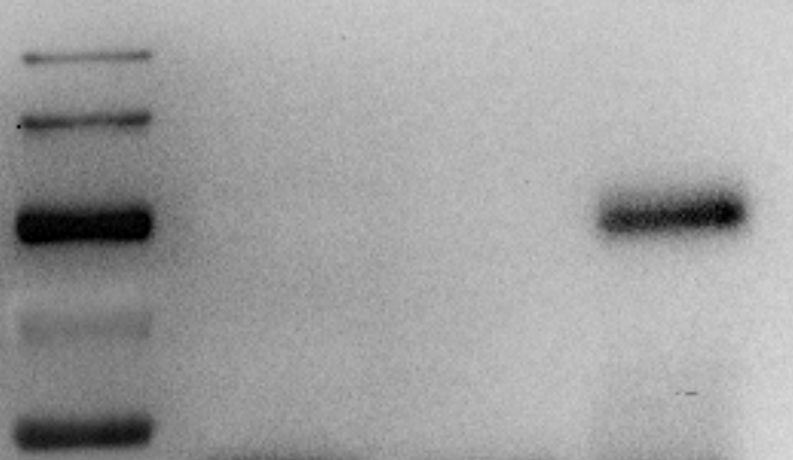

Supplement: Supplementary file 4 — Supplementary Data 2 [file 42003_2022_3856_MOESM4_ESM.zip › Fig. 6b/anti-GFP-CD44a2.tif]

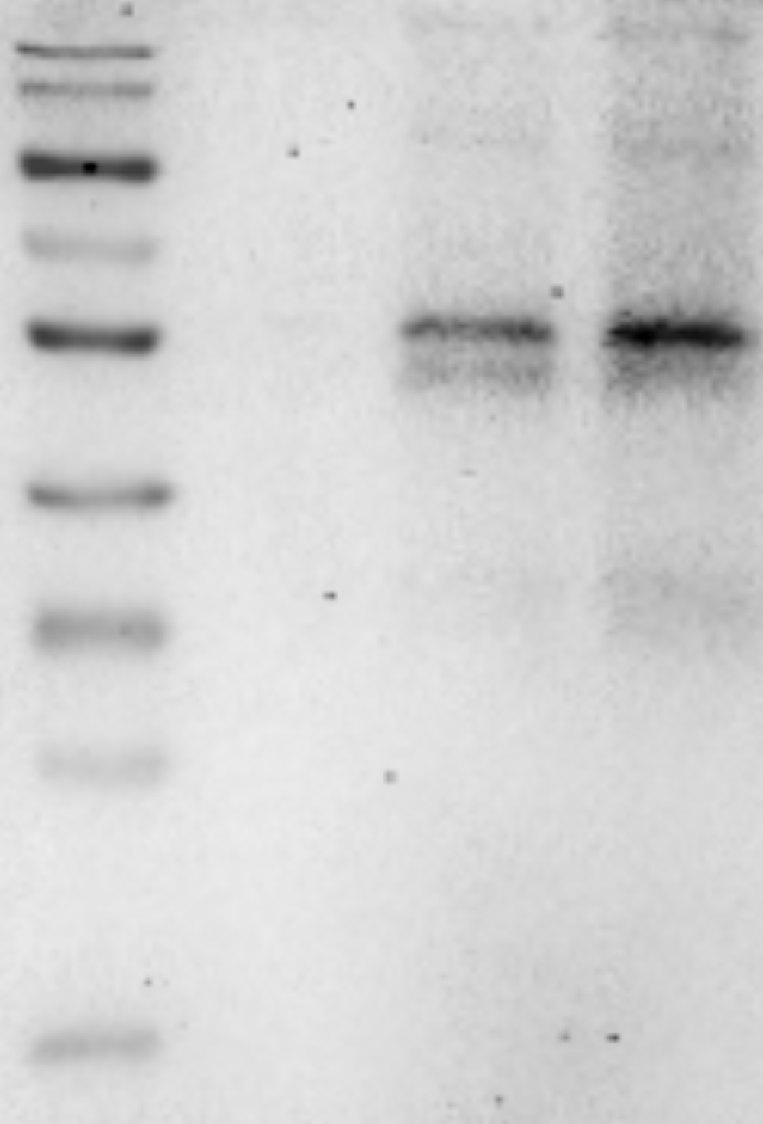

Supplement: Supplementary file 4 — Supplementary Data 2 [file 42003_2022_3856_MOESM4_ESM.zip › Fig. 6c/anti-flag-p53-3.tif]

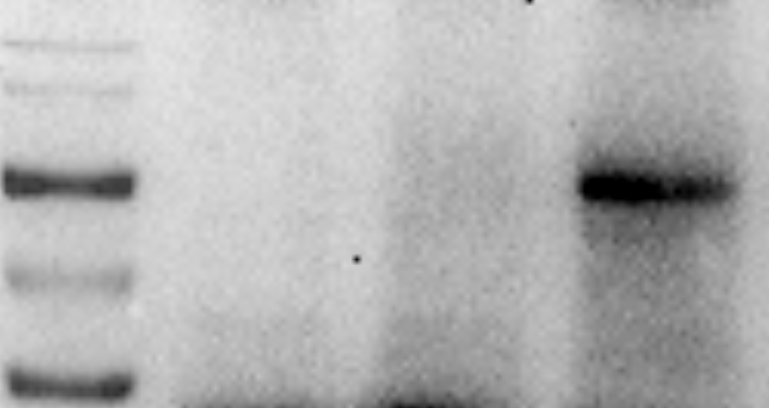

Supplement: Supplementary file 4 — Supplementary Data 2 [file 42003_2022_3856_MOESM4_ESM.zip › Fig. 6c/anti-GFP-CD44a1-2.tif]

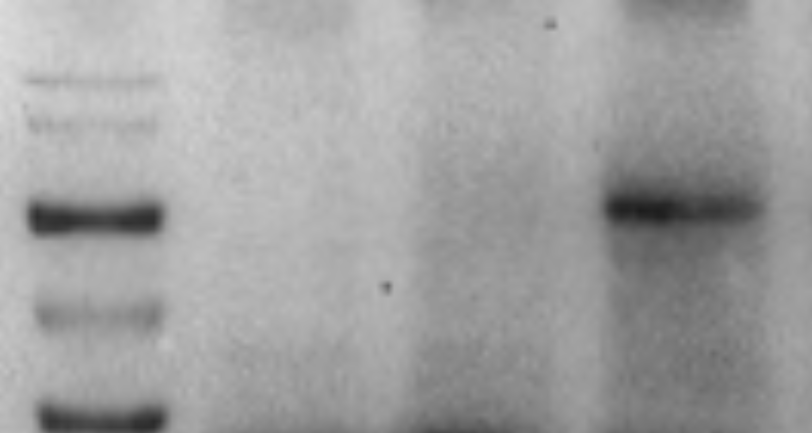

Supplement: Supplementary file 4 — Supplementary Data 2 [file 42003_2022_3856_MOESM4_ESM.zip › Fig. 6c/anti-GFP-CD44a1.tif]

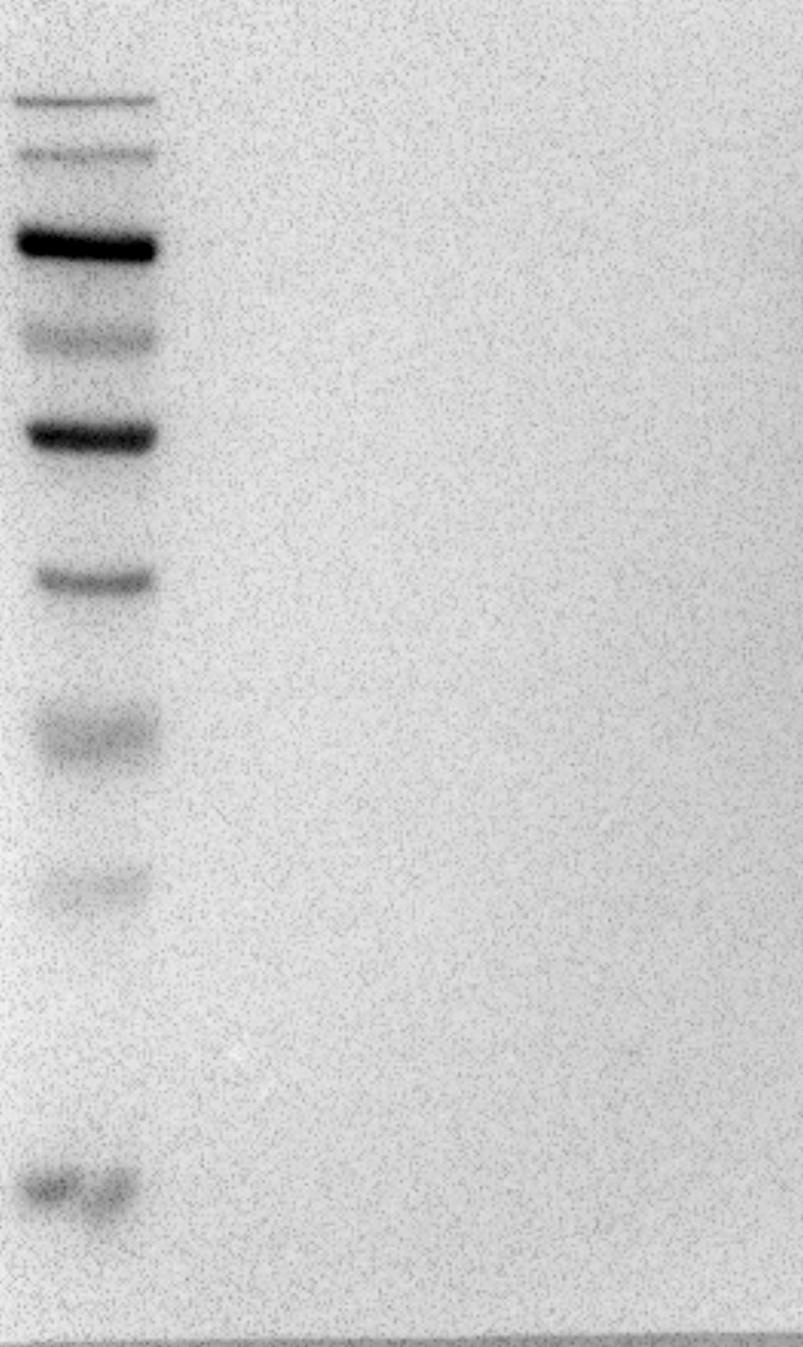

Supplement: Supplementary file 4 — Supplementary Data 2 [file 42003_2022_3856_MOESM4_ESM.zip › Fig. 6c/anti-HDAC1.tif]

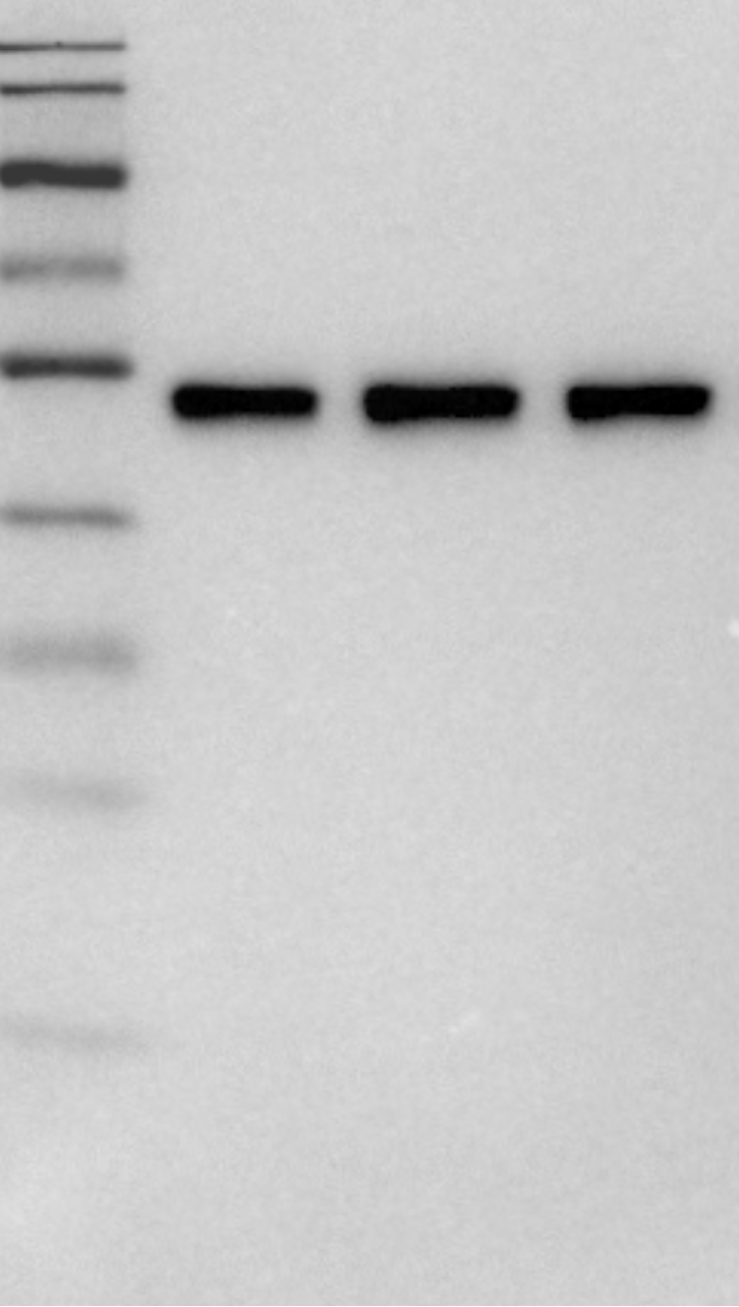

Supplement: Supplementary file 4 — Supplementary Data 2 [file 42003_2022_3856_MOESM4_ESM.zip › Fig. 6c/anti-tubulin a1-1.tif]

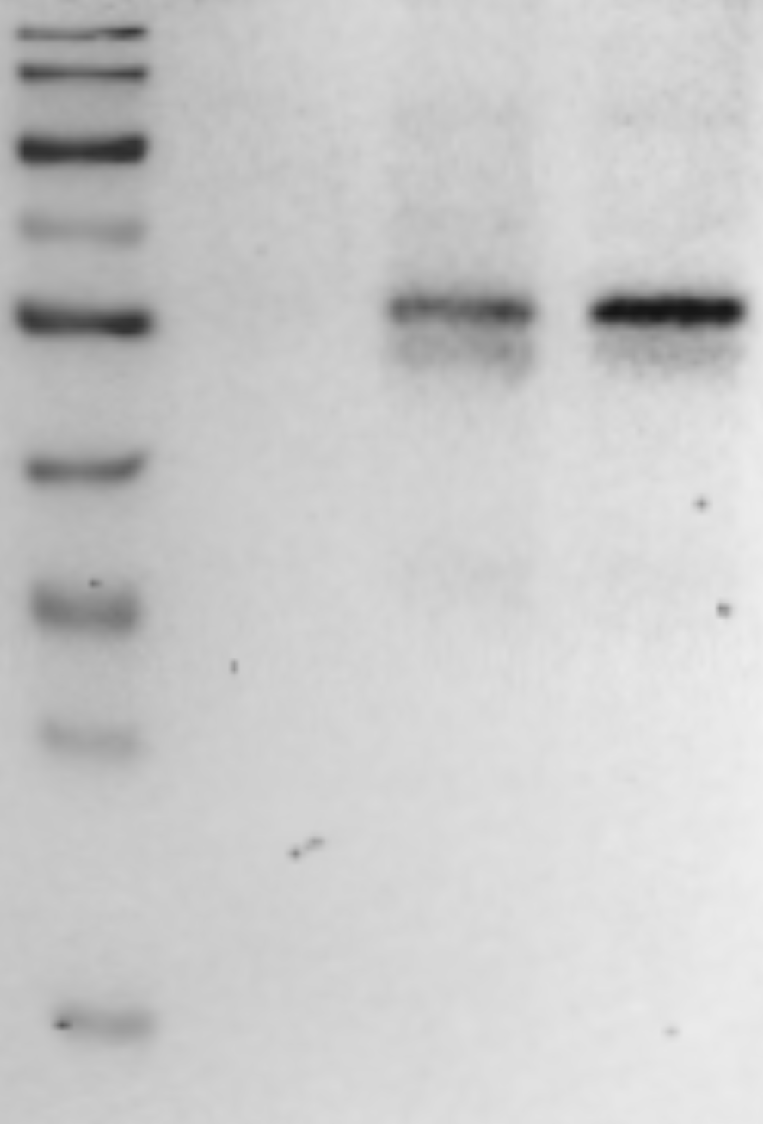

Supplement: Supplementary file 4 — Supplementary Data 2 [file 42003_2022_3856_MOESM4_ESM.zip › Fig. 6d/anti-flag-p53.tif]

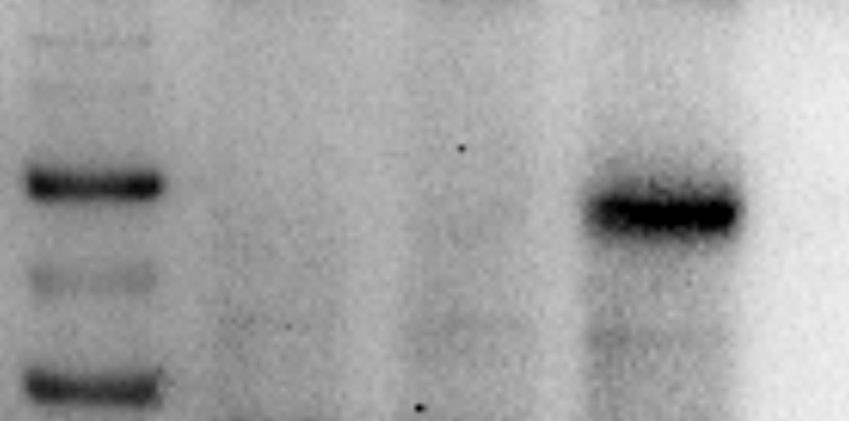

Supplement: Supplementary file 4 — Supplementary Data 2 [file 42003_2022_3856_MOESM4_ESM.zip › Fig. 6d/anti-GFP-cd44a2-2.tif]

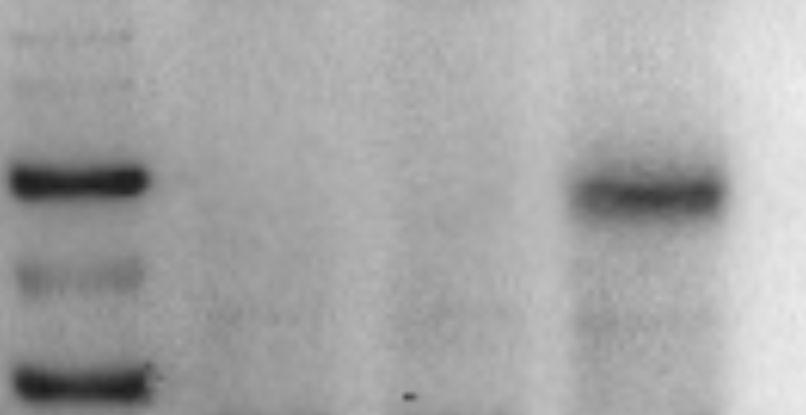

Supplement: Supplementary file 4 — Supplementary Data 2 [file 42003_2022_3856_MOESM4_ESM.zip › Fig. 6d/anti-GFP-cd44a2.tif]

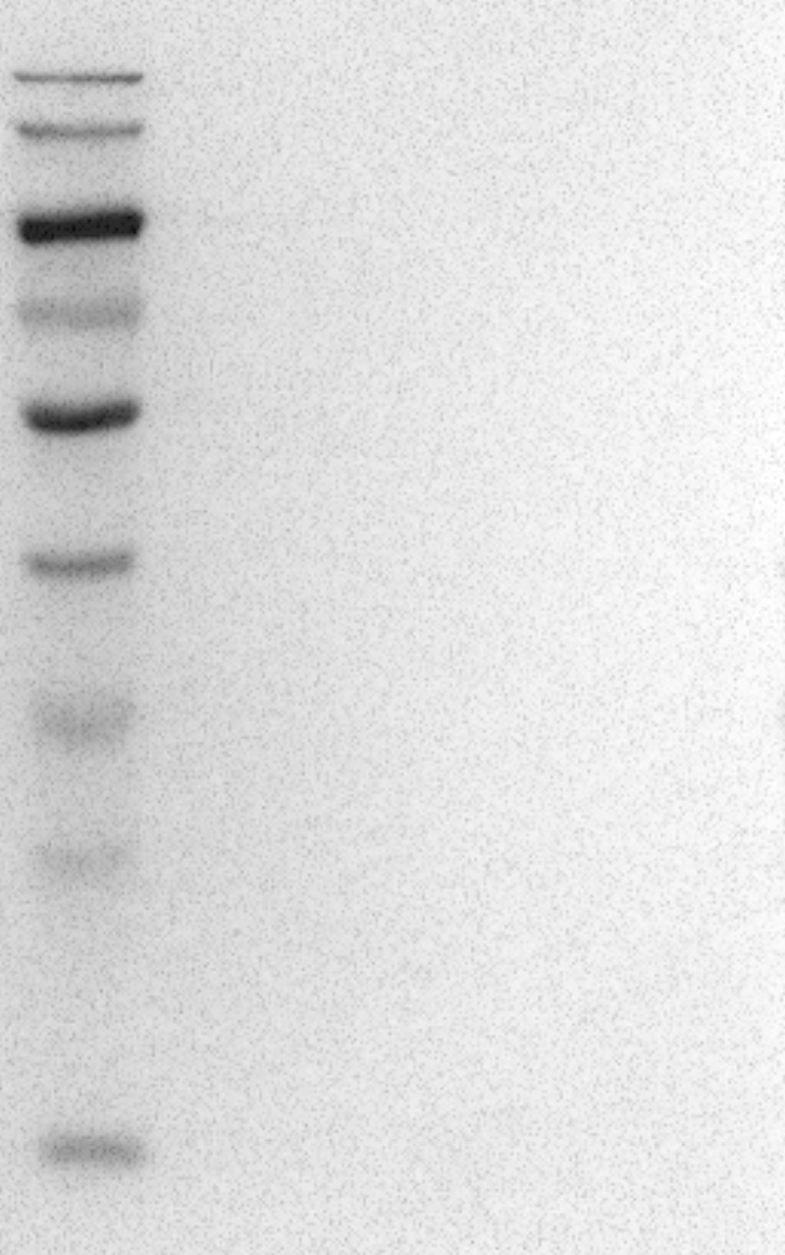

Supplement: Supplementary file 4 — Supplementary Data 2 [file 42003_2022_3856_MOESM4_ESM.zip › Fig. 6d/anti-HDAC1.tif]

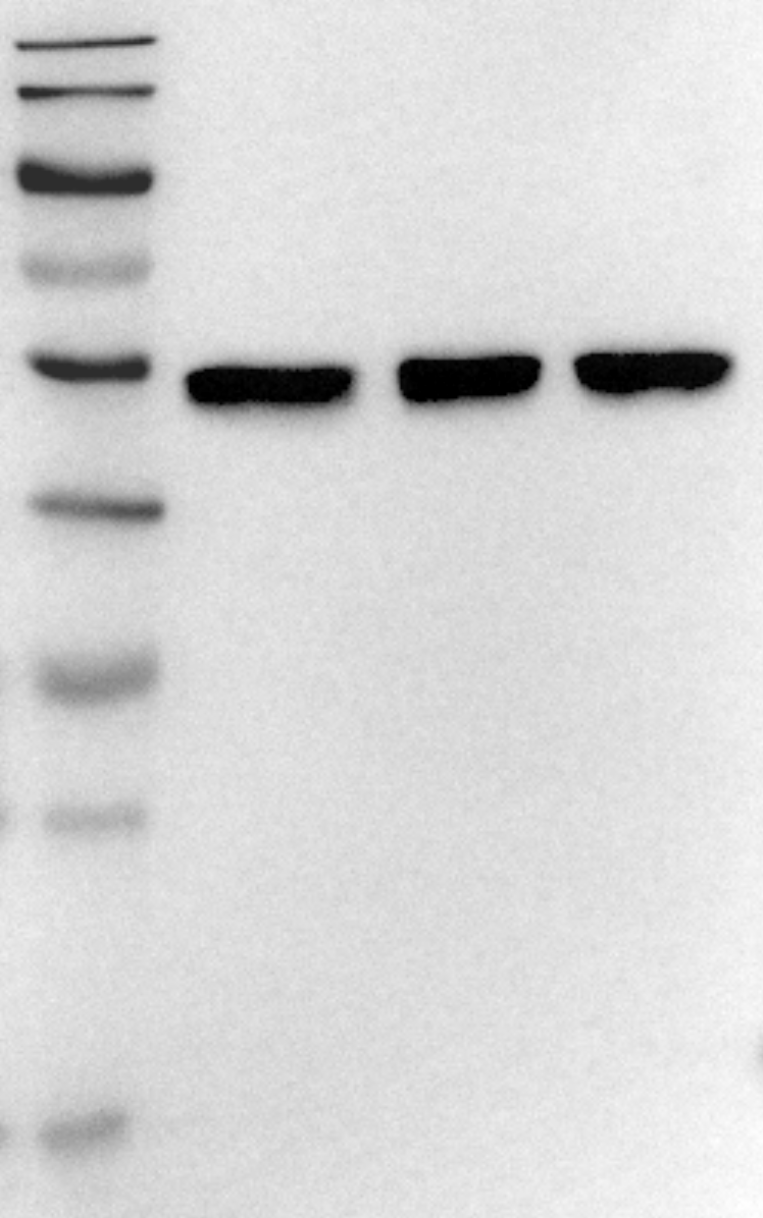

Supplement: Supplementary file 4 — Supplementary Data 2 [file 42003_2022_3856_MOESM4_ESM.zip › Fig. 6d/anti-tublin a2-1.tif]

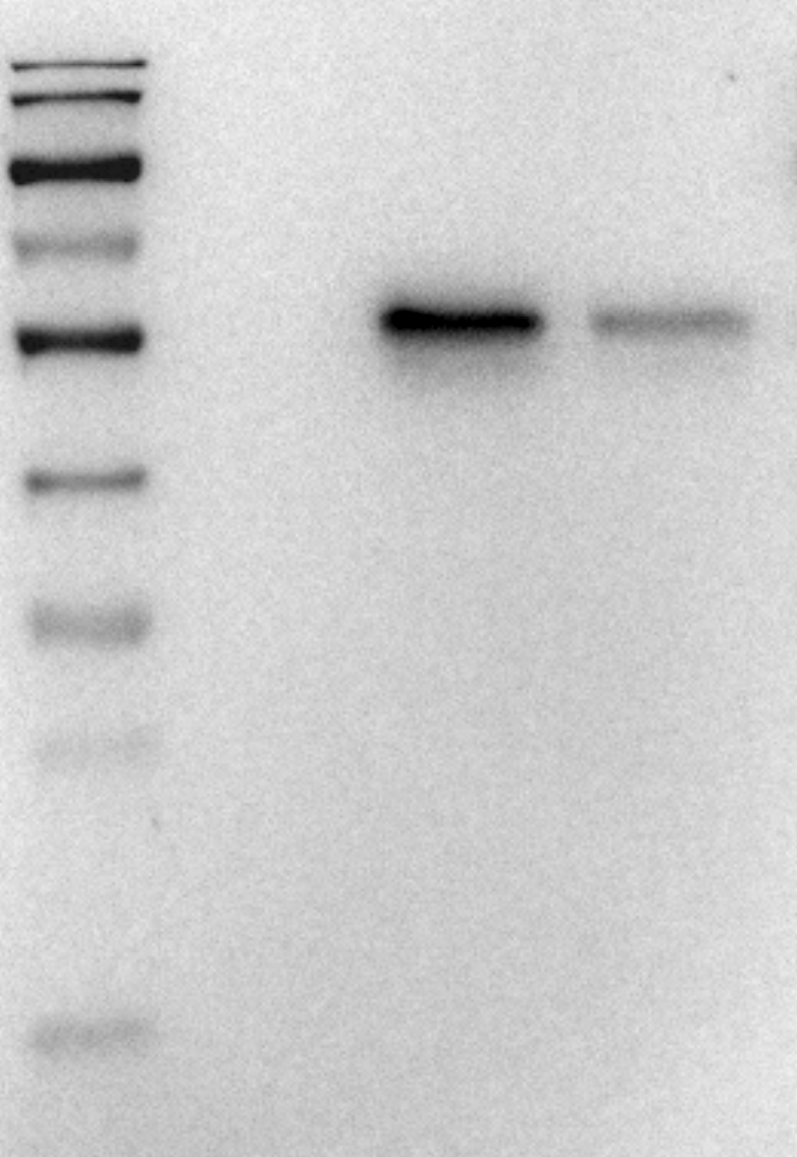

Supplement: Supplementary file 4 — Supplementary Data 2 [file 42003_2022_3856_MOESM4_ESM.zip › Fig. 6e/anti-FLAG-P53-3.tif]

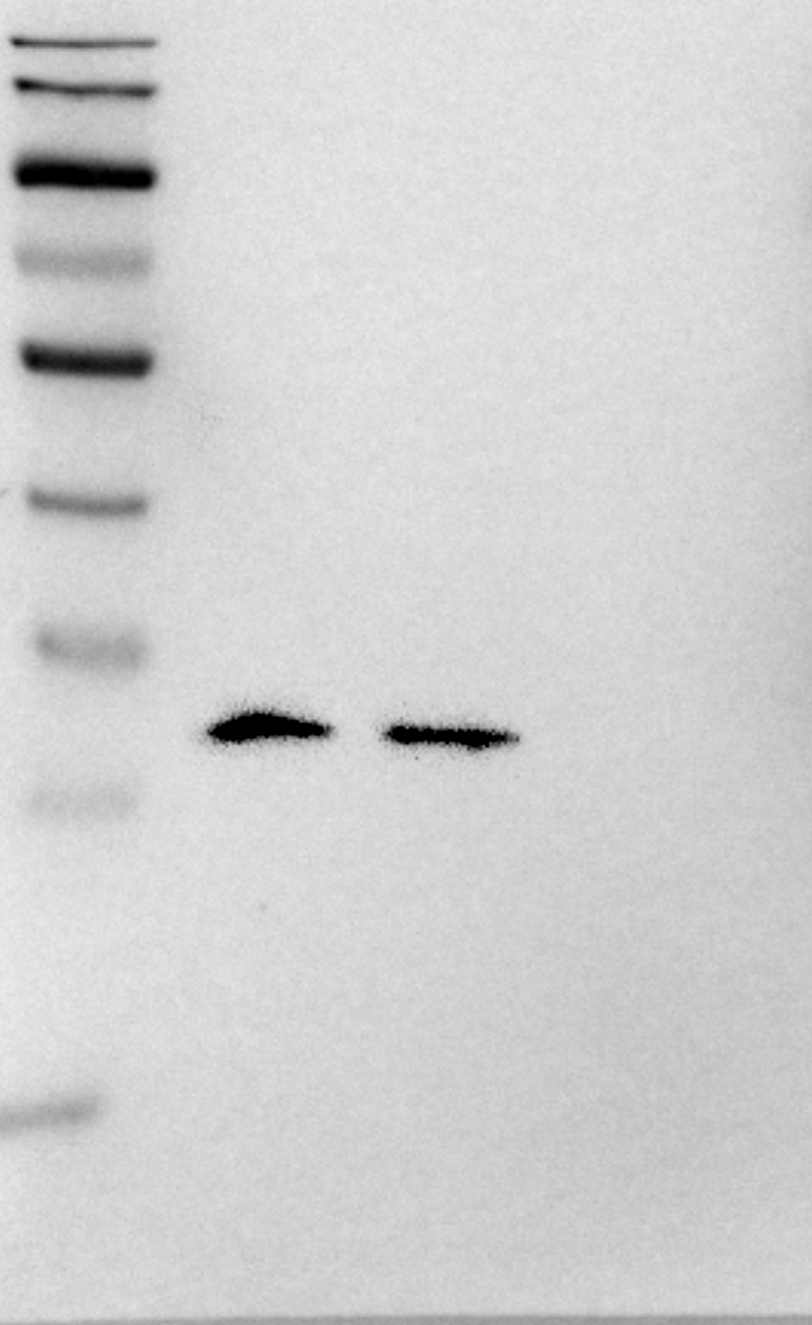

Supplement: Supplementary file 4 — Supplementary Data 2 [file 42003_2022_3856_MOESM4_ESM.zip › Fig. 6e/anti-GFP.tif]

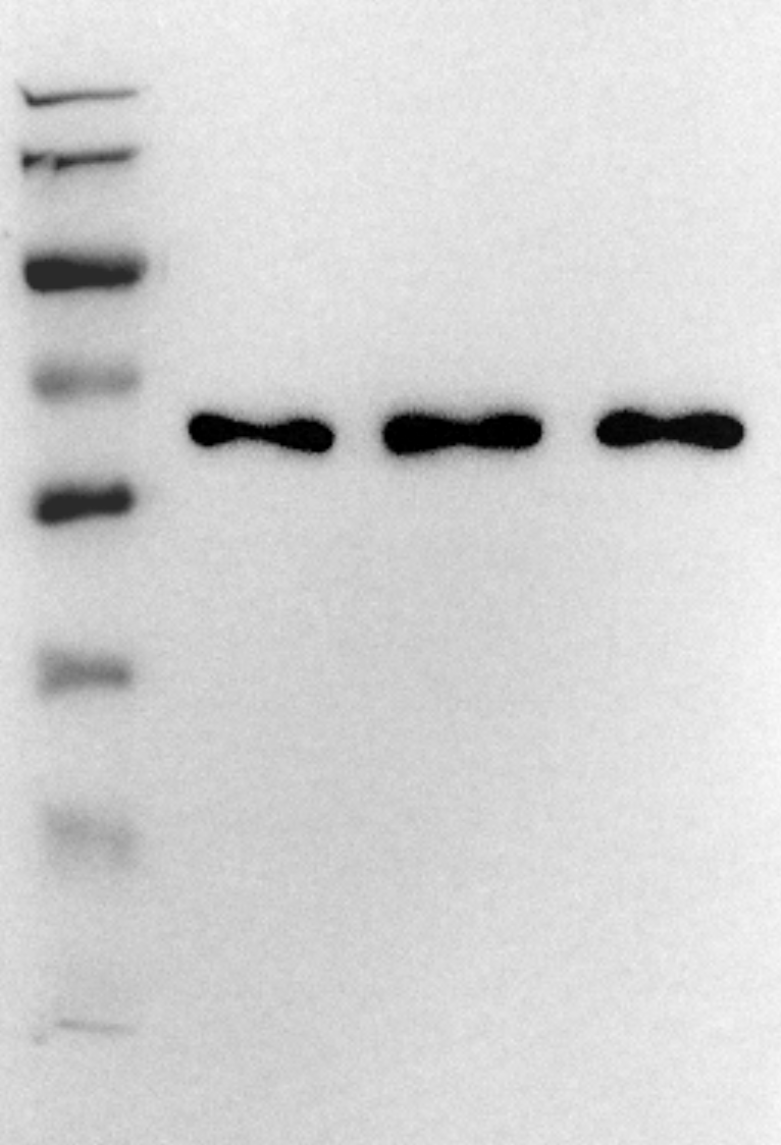

Supplement: Supplementary file 4 — Supplementary Data 2 [file 42003_2022_3856_MOESM4_ESM.zip › Fig. 6e/anti-HDAC1-A1-1.tif]

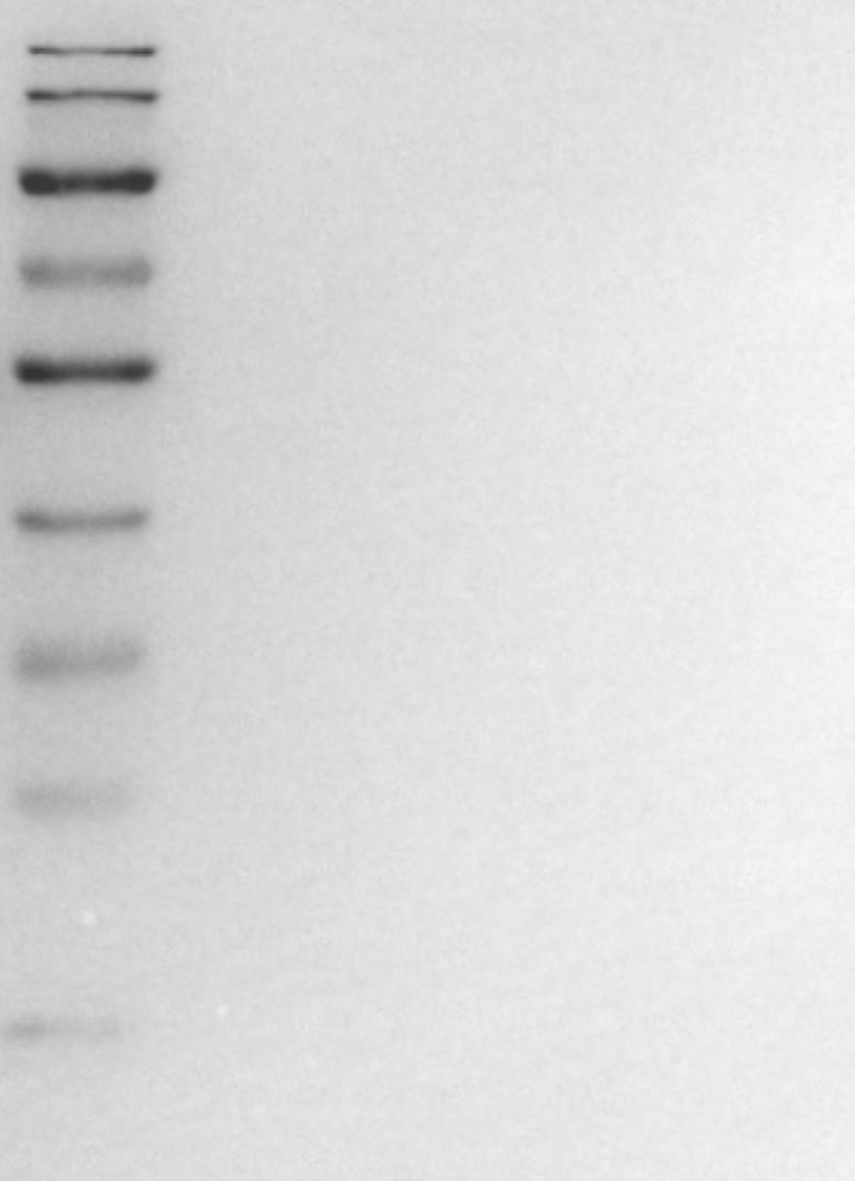

Supplement: Supplementary file 4 — Supplementary Data 2 [file 42003_2022_3856_MOESM4_ESM.zip › Fig. 6e/anti-tubulin.tif]

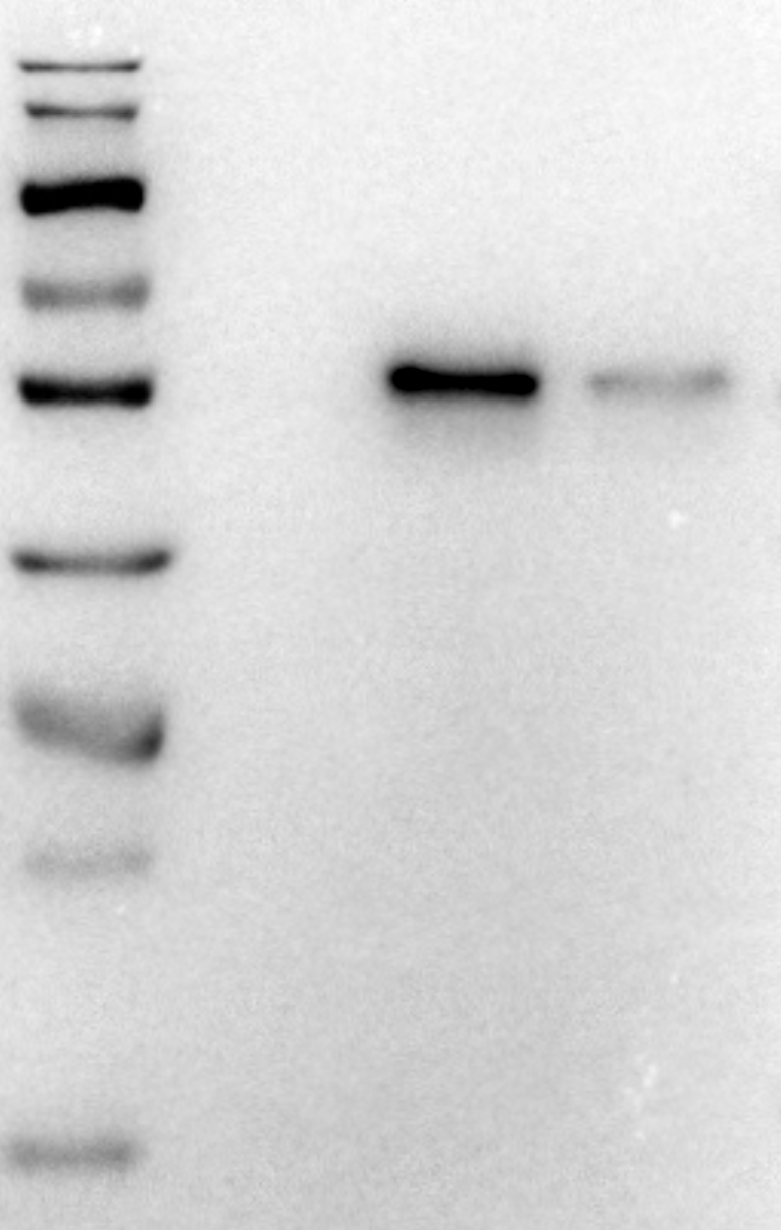

Supplement: Supplementary file 4 — Supplementary Data 2 [file 42003_2022_3856_MOESM4_ESM.zip › Fig. 6f/anti-FLAG-P53-2.tif]

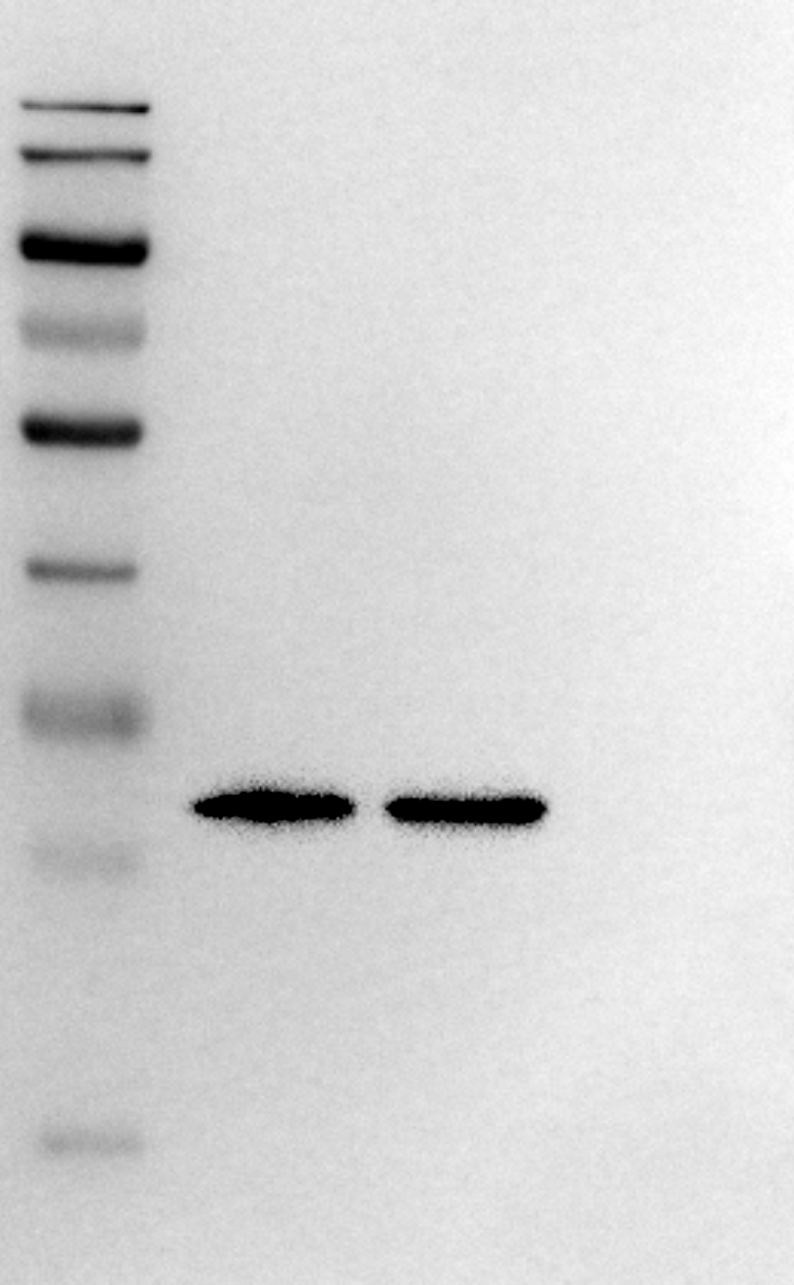

Supplement: Supplementary file 4 — Supplementary Data 2 [file 42003_2022_3856_MOESM4_ESM.zip › Fig. 6f/anti-GFP.tif]

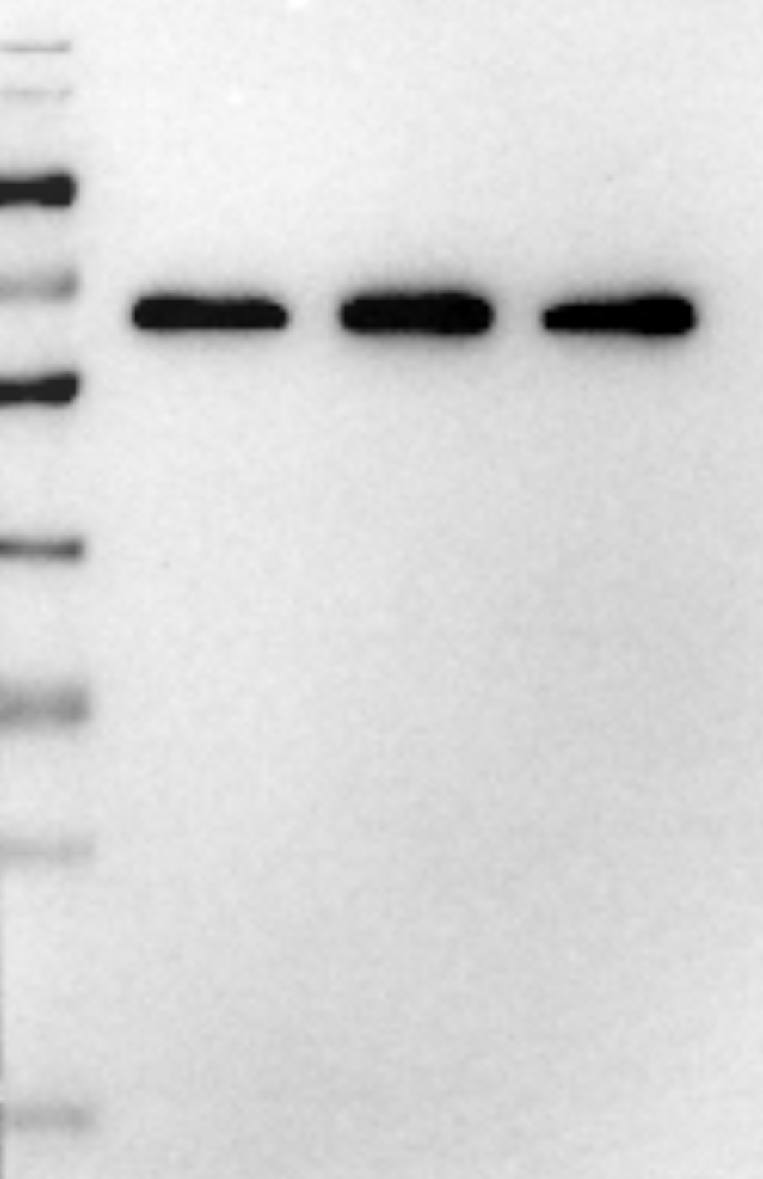

Supplement: Supplementary file 4 — Supplementary Data 2 [file 42003_2022_3856_MOESM4_ESM.zip › Fig. 6f/anti-HDAC1-2.tif]

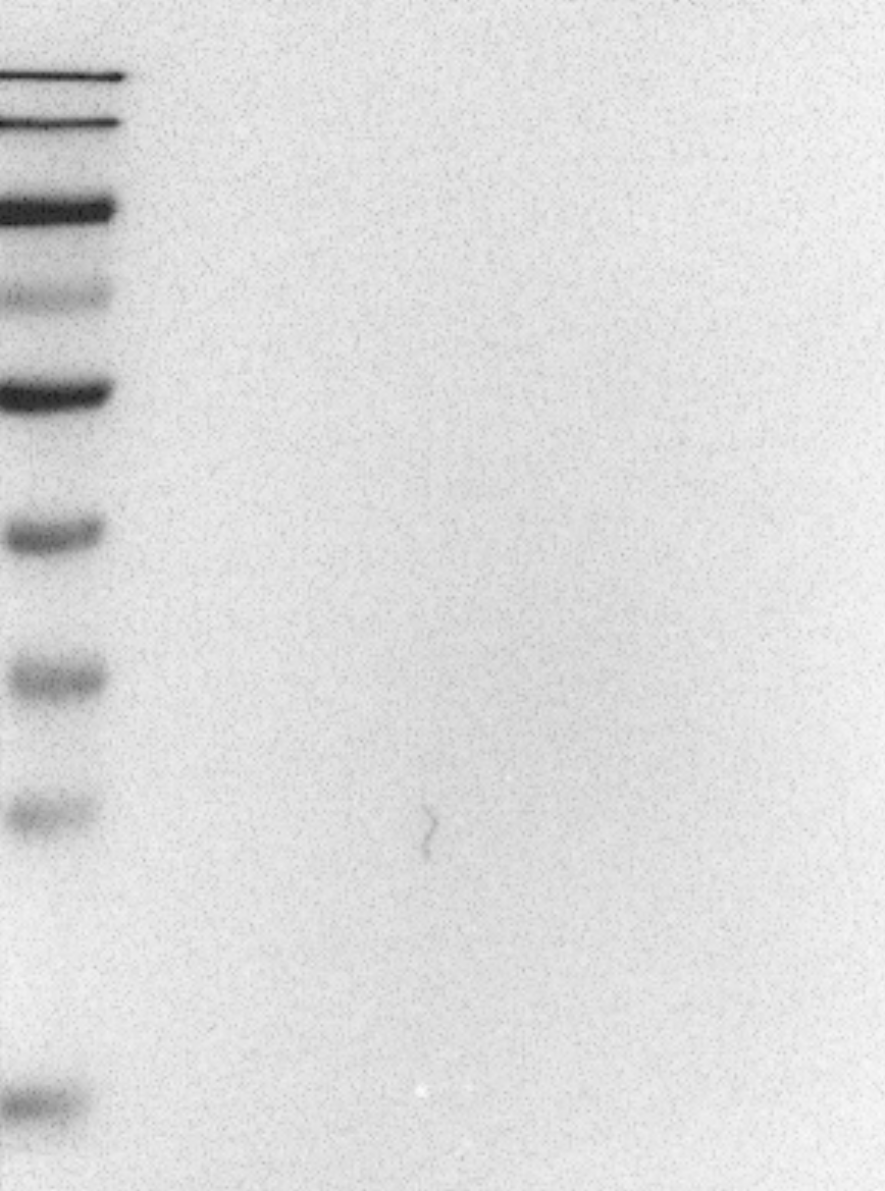

Supplement: Supplementary file 4 — Supplementary Data 2 [file 42003_2022_3856_MOESM4_ESM.zip › Fig. 6f/anti-tubulin.tif]

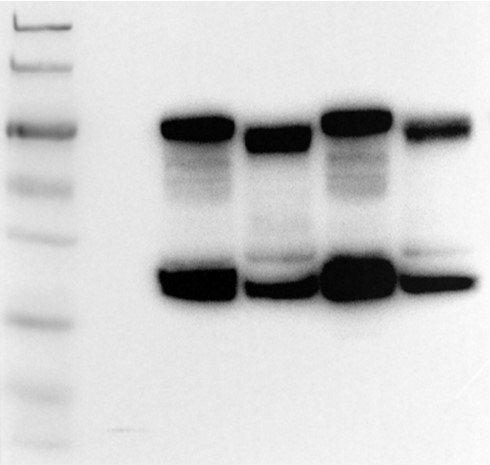

Supplement: Supplementary file 4 — Supplementary Data 2 [file 42003_2022_3856_MOESM4_ESM.zip › Fig. 6g/INPUT/GFP-CD44a-tv1 CD44a-tv2.jpg]

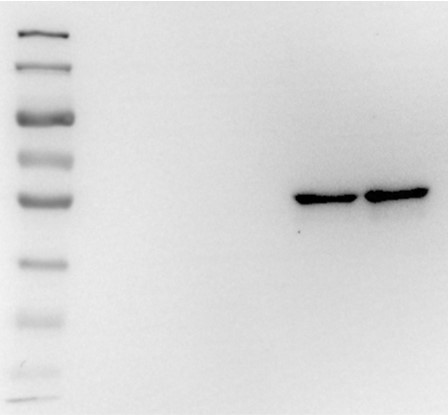

Supplement: Supplementary file 4 — Supplementary Data 2 [file 42003_2022_3856_MOESM4_ESM.zip › Fig. 6g/INPUT/P53-FLAG.jpg]

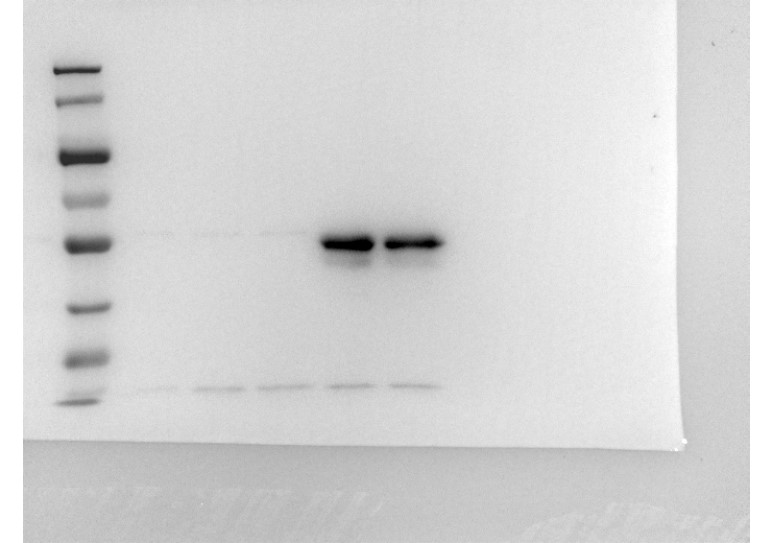

Supplement: Supplementary file 4 — Supplementary Data 2 [file 42003_2022_3856_MOESM4_ESM.zip › Fig. 6g/IP/anti-FLAG.jpg]

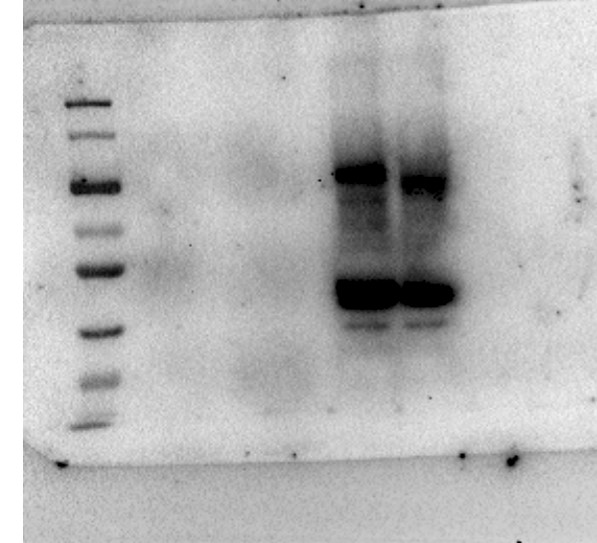

Supplement: Supplementary file 4 — Supplementary Data 2 [file 42003_2022_3856_MOESM4_ESM.zip › Fig. 6g/IP/anti-GFP.jpg]

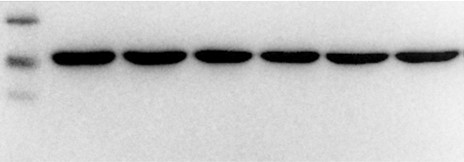

Supplement: Supplementary file 4 — Supplementary Data 2 [file 42003_2022_3856_MOESM4_ESM.zip › Fig. 8g/anti-GAPDH.jpg]

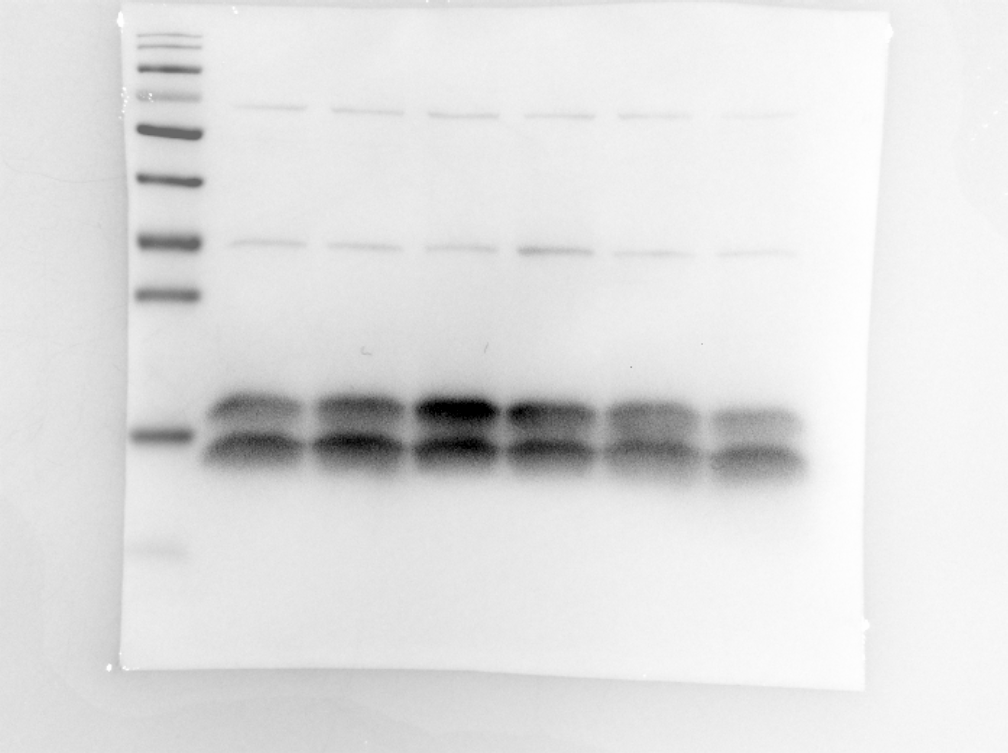

Supplement: Supplementary file 4 — Supplementary Data 2 [file 42003_2022_3856_MOESM4_ESM.zip › Fig. 8g/anti-LC3.tif]

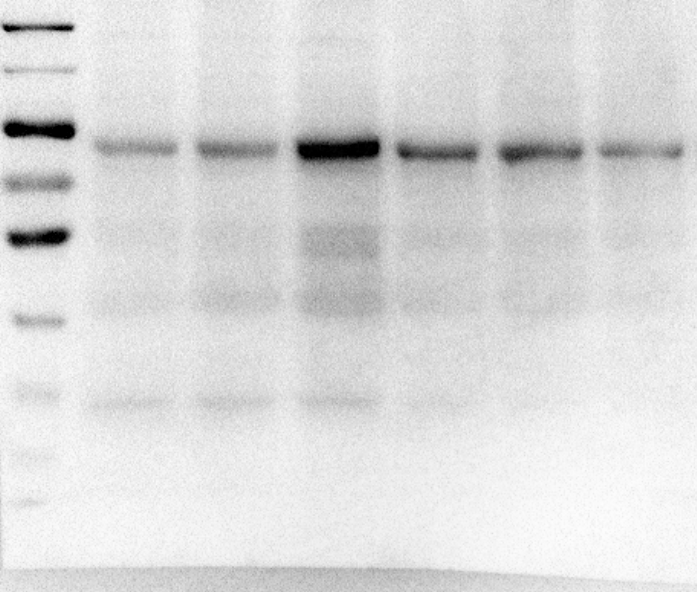

Supplement: Supplementary file 4 — Supplementary Data 2 [file 42003_2022_3856_MOESM4_ESM.zip › Fig. 8g/anti-p62.tif]

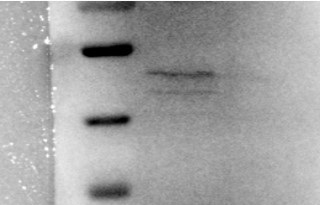

Supplement: Supplementary file 4 — Supplementary Data 2 [file 42003_2022_3856_MOESM4_ESM.zip › Supplementary Fig. 2/a/anti-CD44a.jpg]

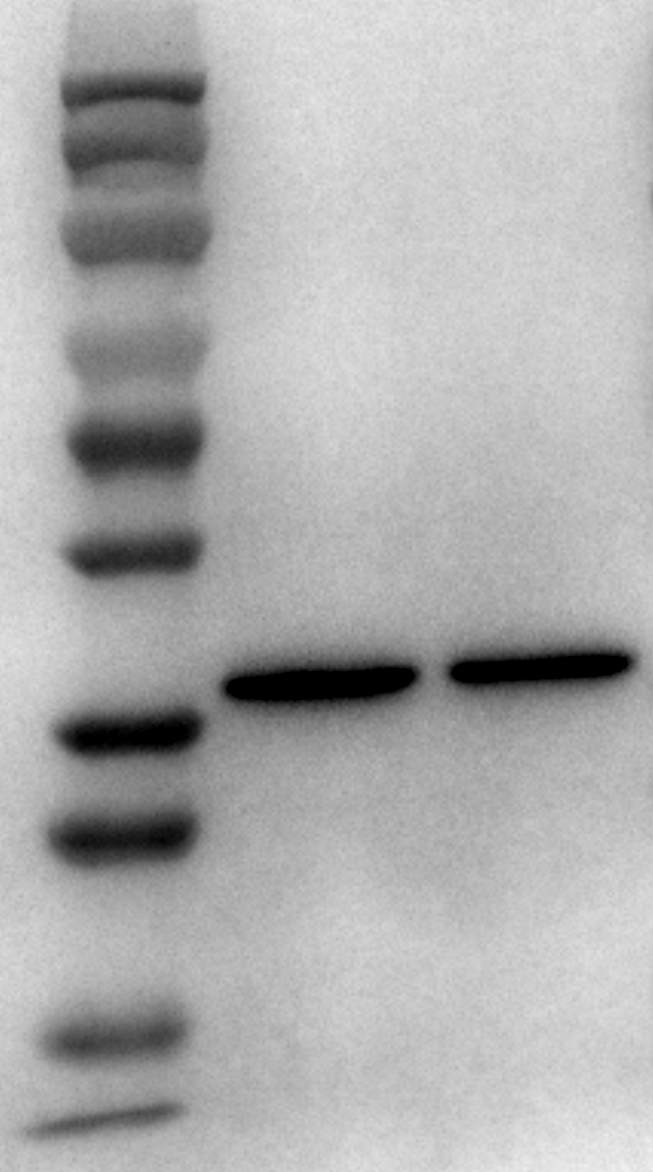

Supplement: Supplementary file 4 — Supplementary Data 2 [file 42003_2022_3856_MOESM4_ESM.zip › Supplementary Fig. 2/a/anti-GADPH.tif]
