# Supplementary figures and images for: CD44a functions as a regulator of p53 signaling, apoptosis and autophagy in the antibacterial immune response (part 2 of 2)
Source: Commun Biol. 2022 Aug 30;5:889. doi: 10.1038/s42003-022-03856-1 (PMC9427754; doi:10.1038/s42003-022-03856-1)

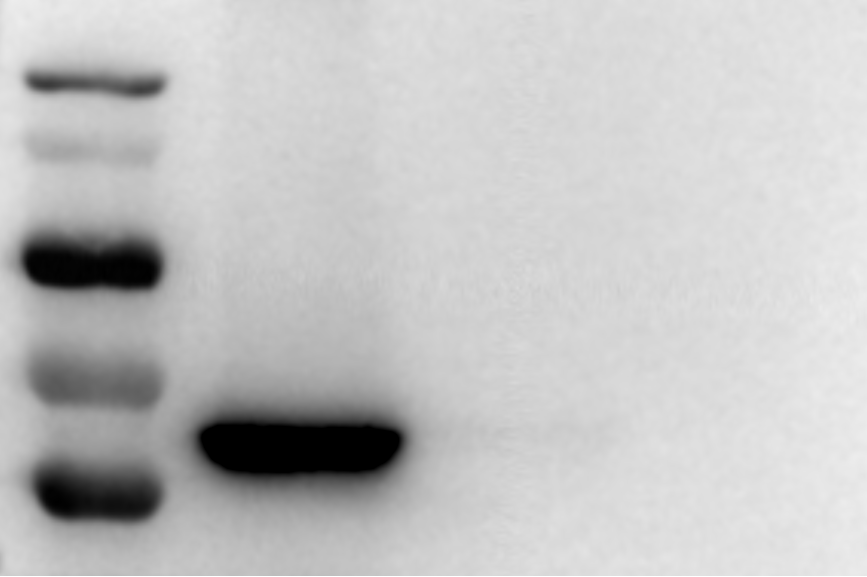

Supplement: Supplementary file 4 — Supplementary Data 2 [file 42003_2022_3856_MOESM4_ESM.zip › Supplementary Fig. 2/b/anti-AKT.tif]

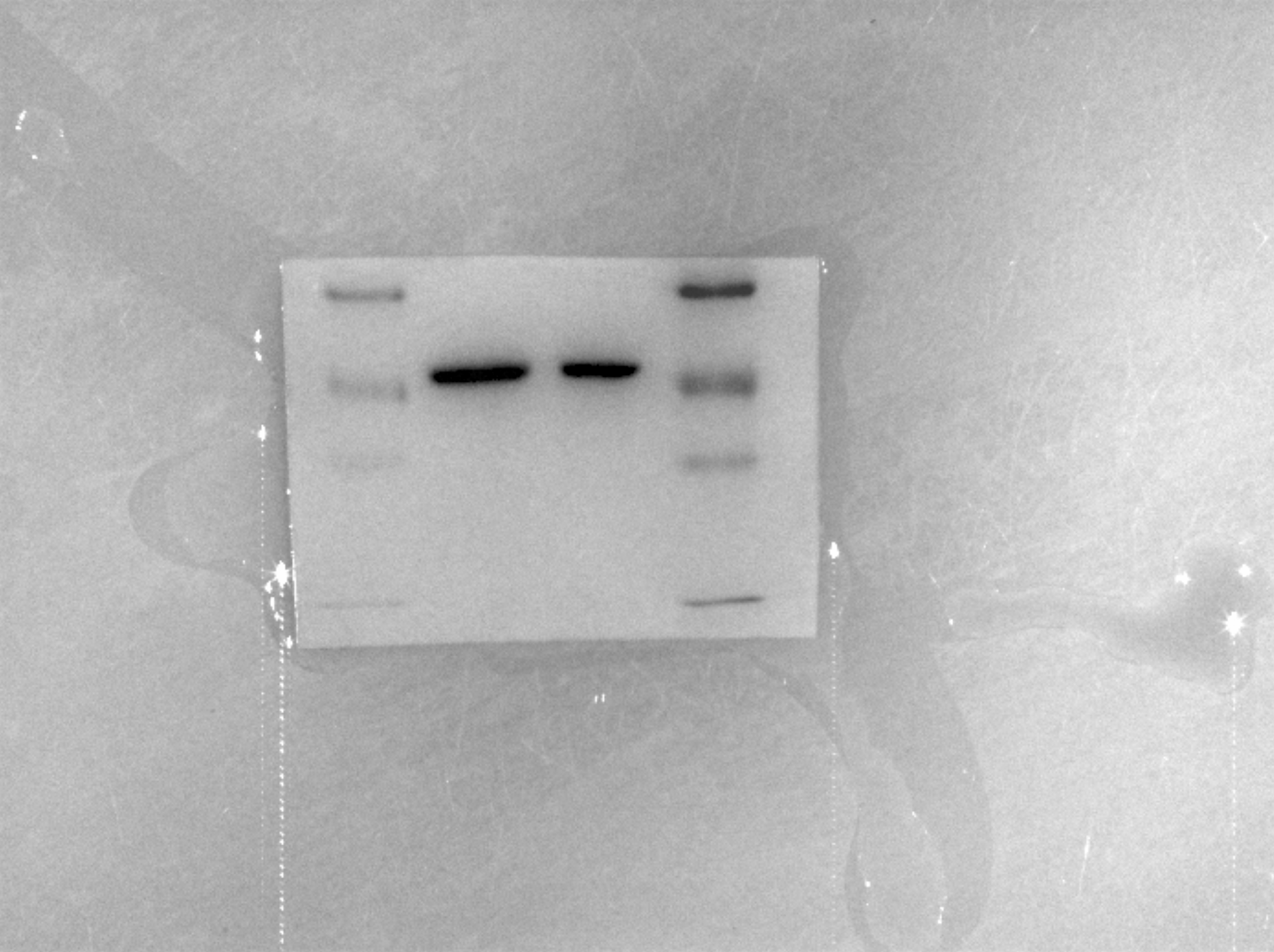

Supplement: Supplementary file 4 — Supplementary Data 2 [file 42003_2022_3856_MOESM4_ESM.zip › Supplementary Fig. 2/b/anti-gadph.tif]

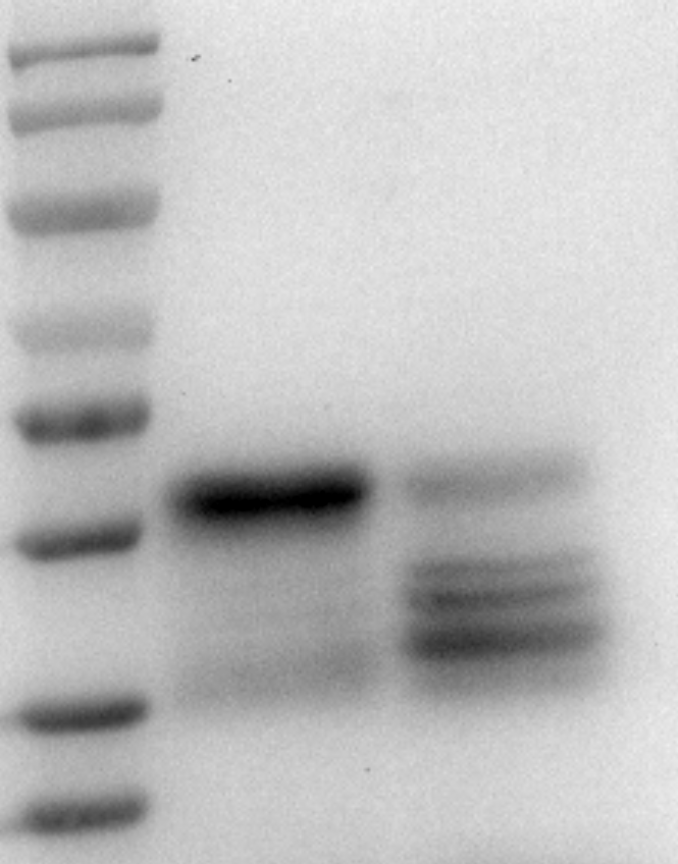

Supplement: Supplementary file 4 — Supplementary Data 2 [file 42003_2022_3856_MOESM4_ESM.zip › Supplementary Fig. 2/b/anti-GSK-3β.tif]

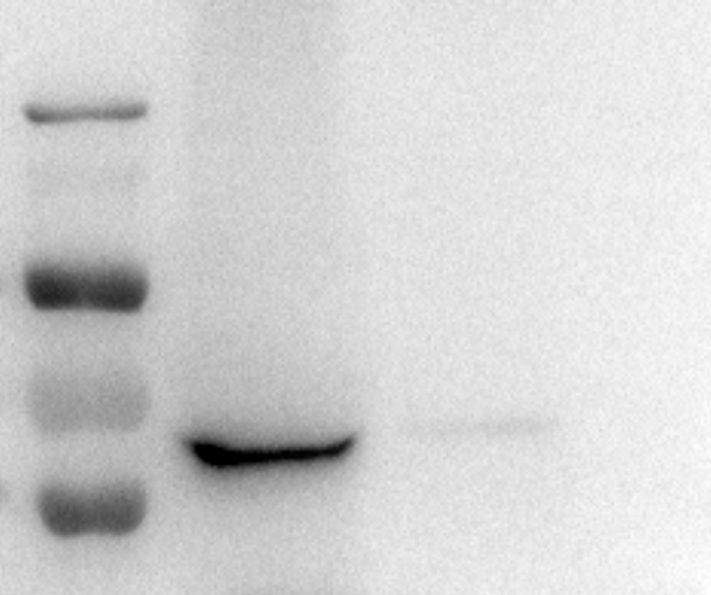

Supplement: Supplementary file 4 — Supplementary Data 2 [file 42003_2022_3856_MOESM4_ESM.zip › Supplementary Fig. 2/b/anti-phospho-AKT.tif]

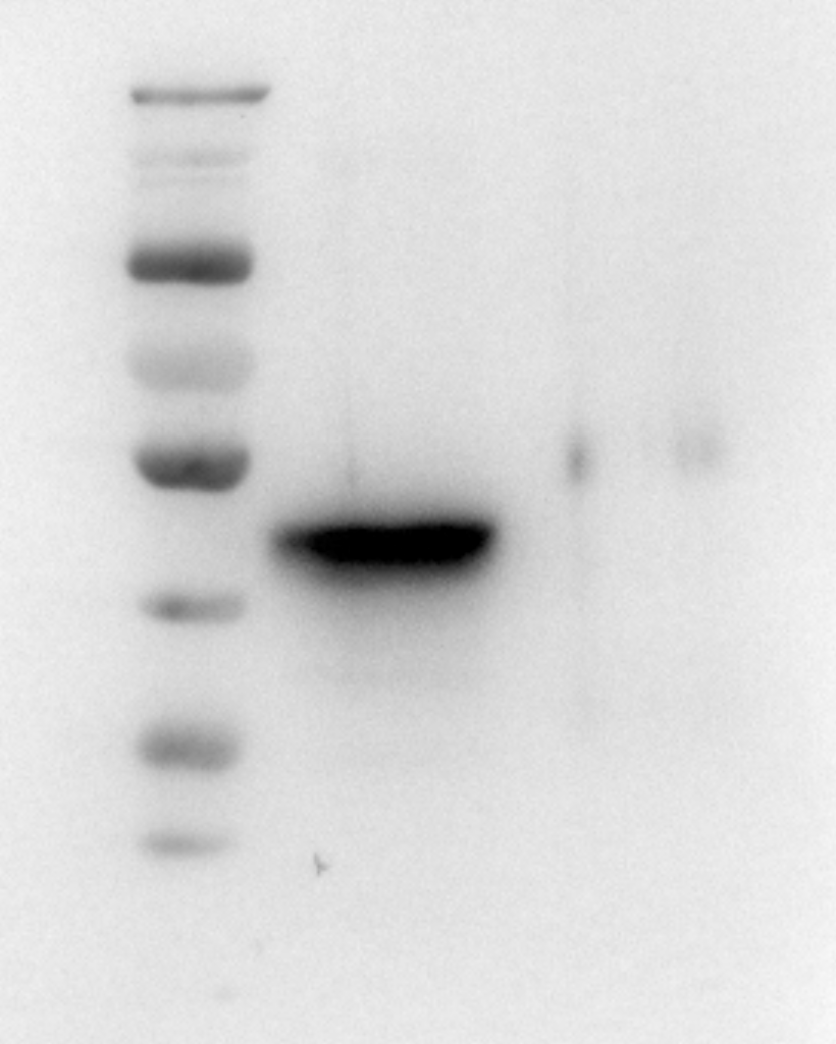

Supplement: Supplementary file 4 — Supplementary Data 2 [file 42003_2022_3856_MOESM4_ESM.zip › Supplementary Fig. 2/b/anti-phospho-gsk-3β.tif]

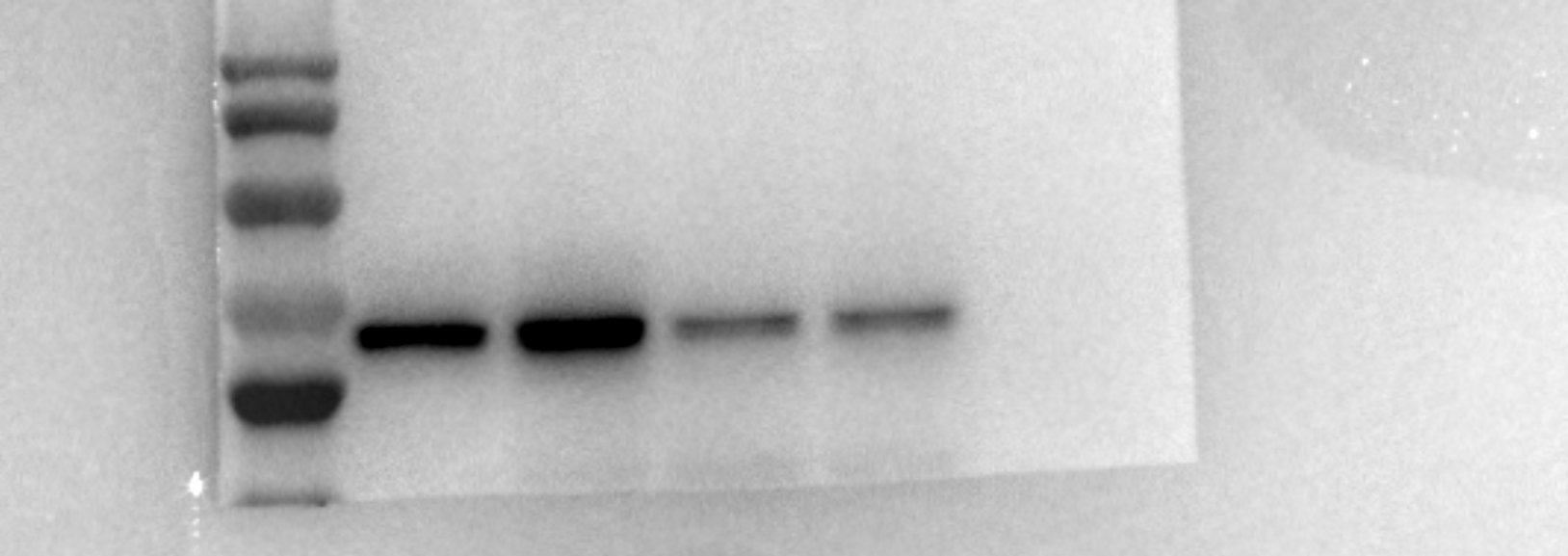

Supplement: Supplementary file 4 — Supplementary Data 2 [file 42003_2022_3856_MOESM4_ESM.zip › Supplementary Fig. 2/c/anti-akt.tif]

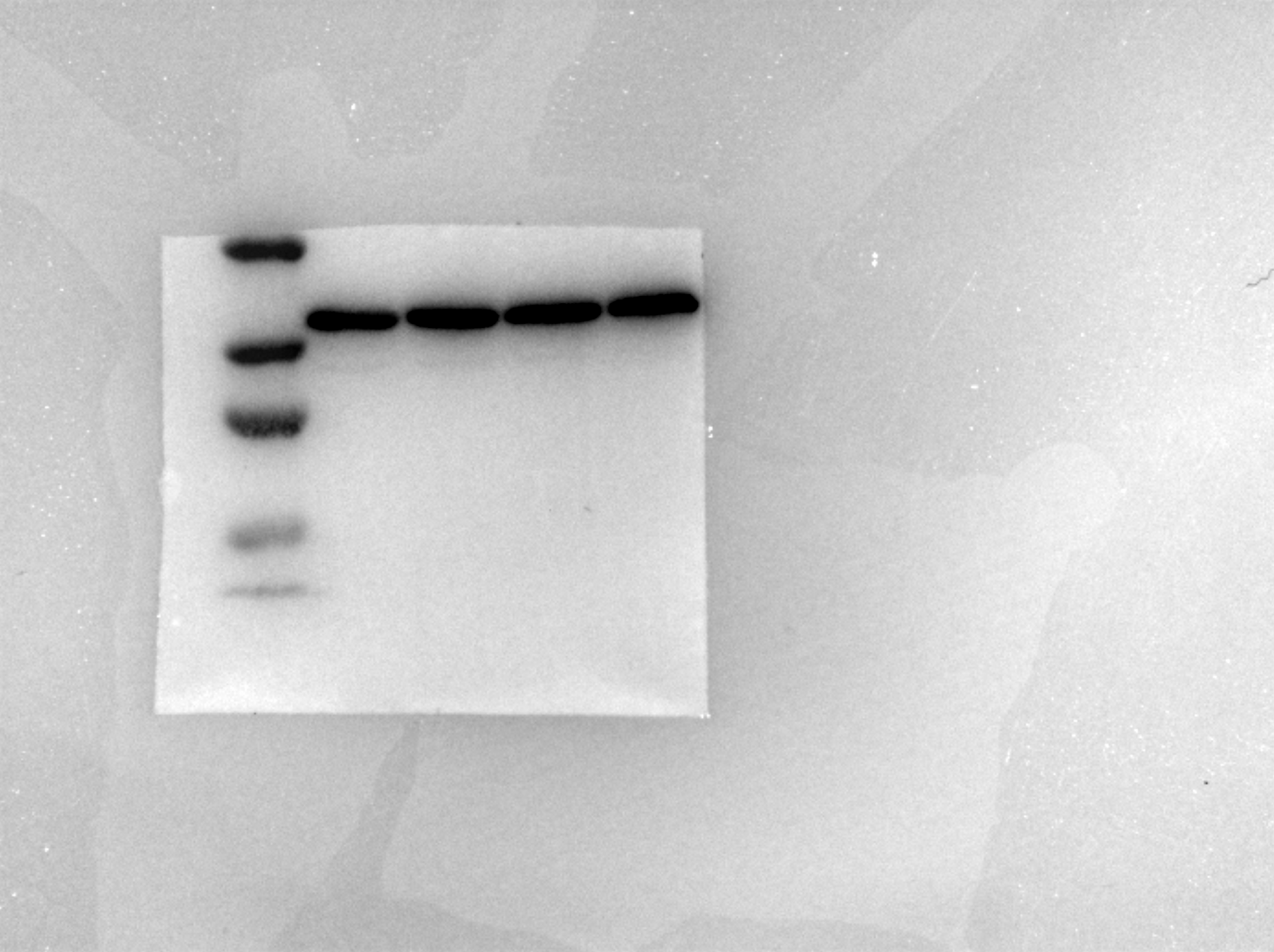

Supplement: Supplementary file 4 — Supplementary Data 2 [file 42003_2022_3856_MOESM4_ESM.zip › Supplementary Fig. 2/c/anti-gadph.tif]

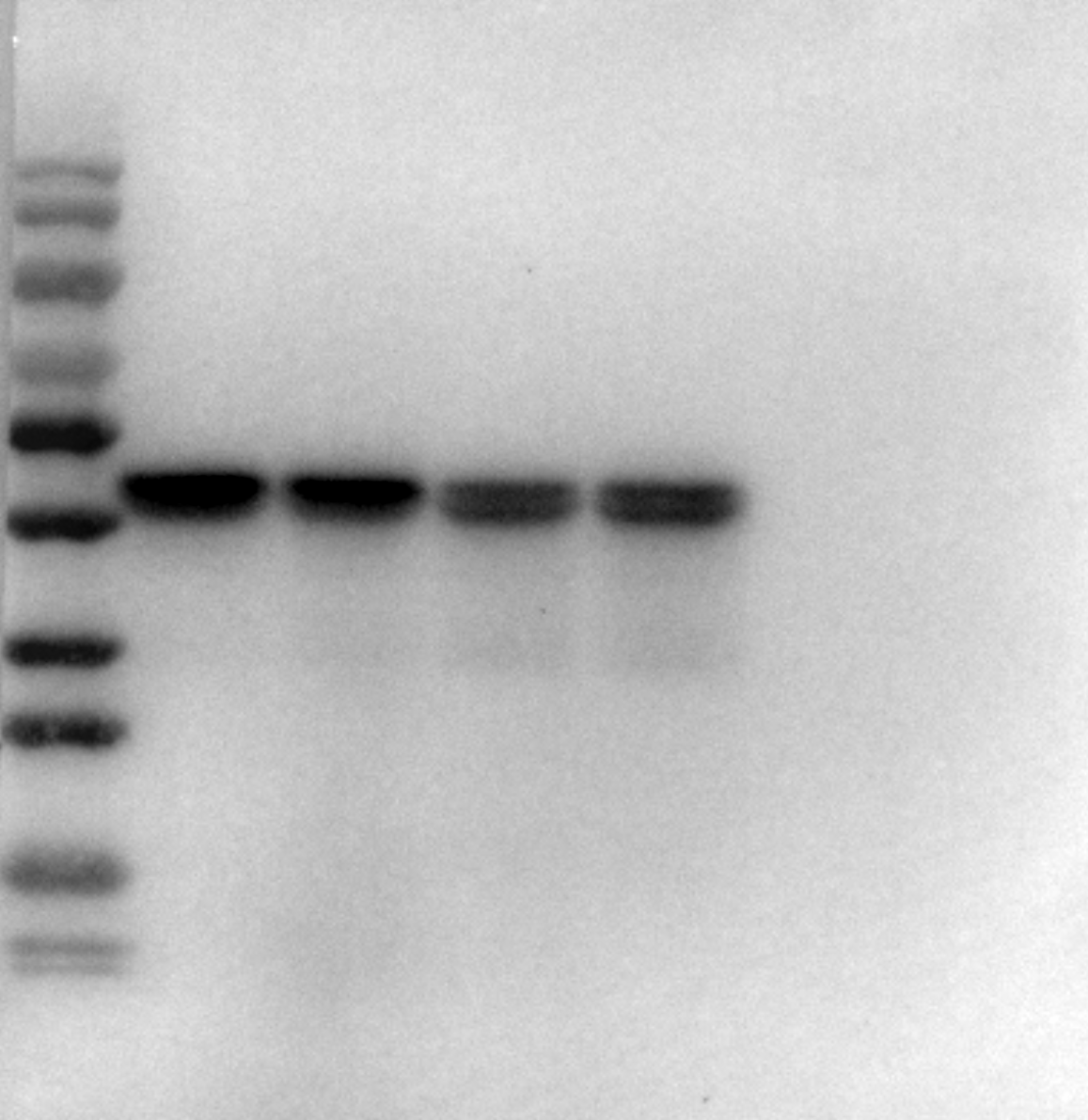

Supplement: Supplementary file 4 — Supplementary Data 2 [file 42003_2022_3856_MOESM4_ESM.zip › Supplementary Fig. 2/c/anti-gsk-3β.tif]

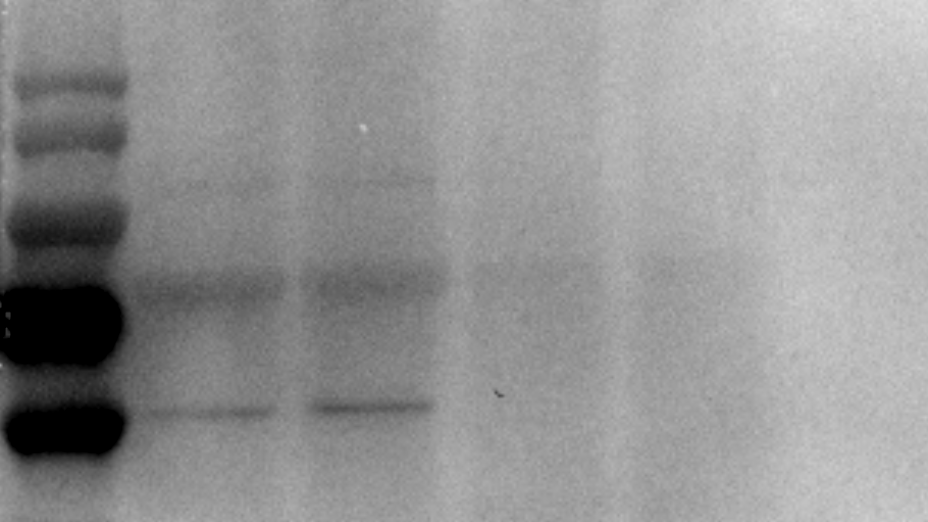

Supplement: Supplementary file 4 — Supplementary Data 2 [file 42003_2022_3856_MOESM4_ESM.zip › Supplementary Fig. 2/c/anti-phospho-akt.tif]

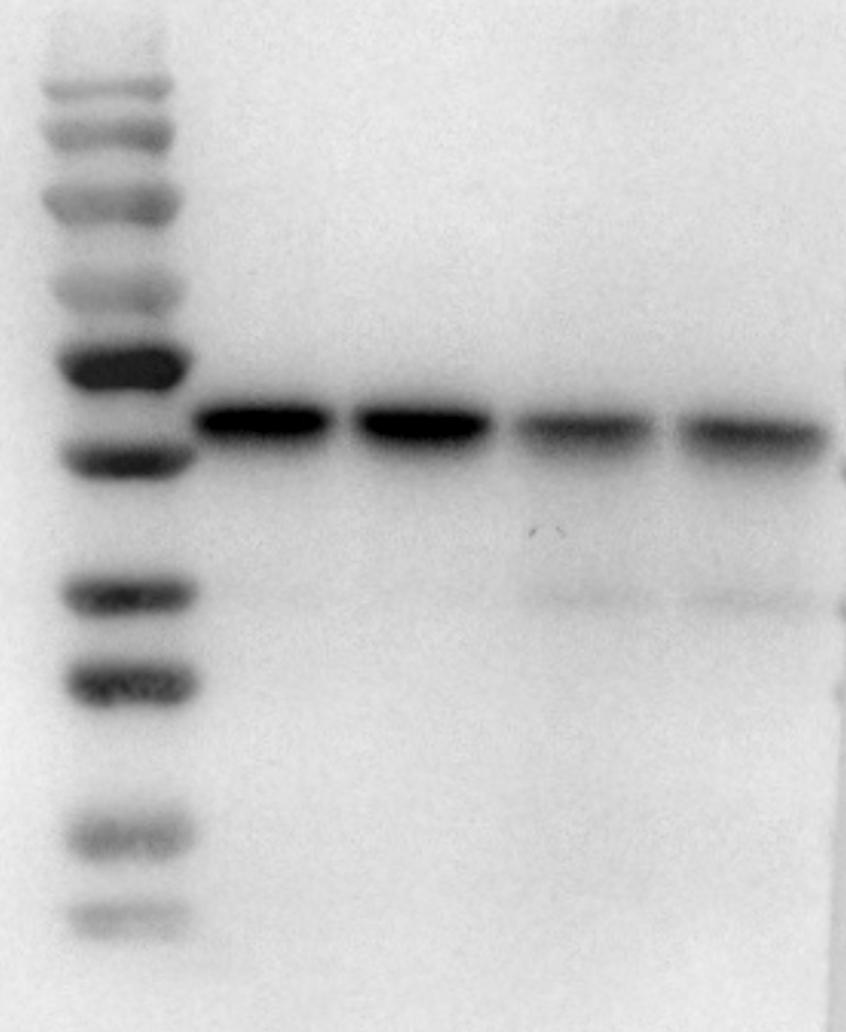

Supplement: Supplementary file 4 — Supplementary Data 2 [file 42003_2022_3856_MOESM4_ESM.zip › Supplementary Fig. 2/c/anti-phospho-gsk-3β.tif]

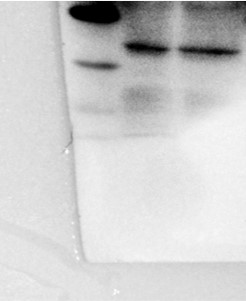

Supplement: Supplementary file 4 — Supplementary Data 2 [file 42003_2022_3856_MOESM4_ESM.zip › Supplementary Fig. 5/a/input/anti-p53.jpg]

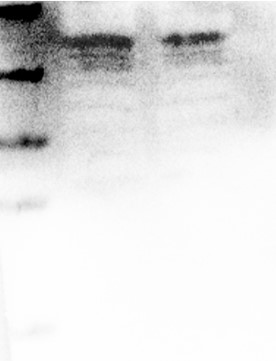

Supplement: Supplementary file 4 — Supplementary Data 2 [file 42003_2022_3856_MOESM4_ESM.zip › Supplementary Fig. 5/a/input/anti.CD44a.jpg]

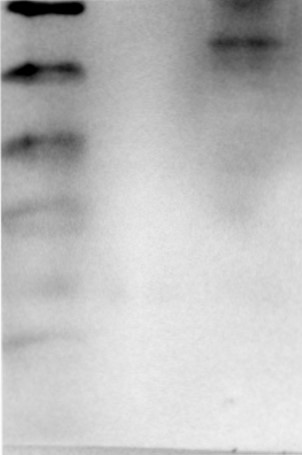

Supplement: Supplementary file 4 — Supplementary Data 2 [file 42003_2022_3856_MOESM4_ESM.zip › Supplementary Fig. 5/a/IP/anti-CD44a.jpg]

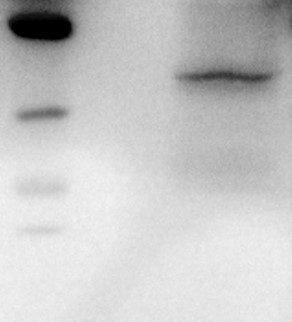

Supplement: Supplementary file 4 — Supplementary Data 2 [file 42003_2022_3856_MOESM4_ESM.zip › Supplementary Fig. 5/a/IP/anti-p53.jpg]

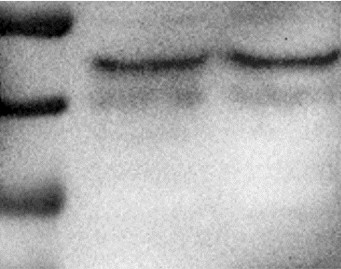

Supplement: Supplementary file 4 — Supplementary Data 2 [file 42003_2022_3856_MOESM4_ESM.zip › Supplementary Fig. 5/b/input/anti-CD44a.jpg]

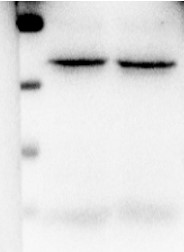

Supplement: Supplementary file 4 — Supplementary Data 2 [file 42003_2022_3856_MOESM4_ESM.zip › Supplementary Fig. 5/b/input/anti-p53.jpg]

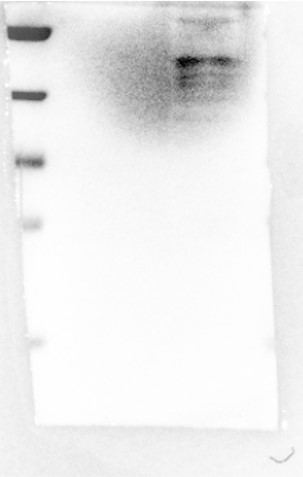

Supplement: Supplementary file 4 — Supplementary Data 2 [file 42003_2022_3856_MOESM4_ESM.zip › Supplementary Fig. 5/b/IP/anti-CD44a.jpg]

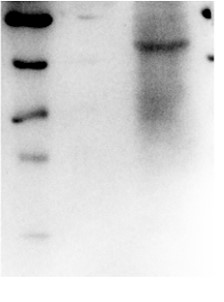

Supplement: Supplementary file 4 — Supplementary Data 2 [file 42003_2022_3856_MOESM4_ESM.zip › Supplementary Fig. 5/b/IP/anti-p53.jpg]
