# Supplementary material for: CD44a functions as a regulator of p53 signaling, apoptosis and autophagy in the antibacterial immune response
Source: Commun Biol. 2022 Aug 30;5:889. doi: 10.1038/s42003-022-03856-1 (PMC9427754; doi:10.1038/s42003-022-03856-1)
Supplement: Supplementary file 5 — Reporting Summary [file 42003_2022_3856_MOESM5_ESM.pdf]

nature portfolio

Corresponding author(s): Mingxian Chang

Last updated by author(s): Jul 22, 2022

Reporting Summary

Nature Portfolio wishes to improve the reproducibility of the work that we publish. This form provides structure for consistency and transparency in reporting. For further information on Nature Portfolio policies, see our Editorial Policies and the Editorial Policy Checklist.

Statistics

For all statistical analyses, confirm that the following items are present in the figure legend, table legend, main text, or Methods section.

n/a	Confirmed	
☐	☒	The exact sample size (n) for each experimental group/condition, given as a discrete number and unit of measurement
☐	☒	A statement on whether measurements were taken from distinct samples or whether the same sample was measured repeatedly
☐	☒	The statistical test(s) used AND whether they are one- or two-sided<br>*Only common tests should be described solely by name; describe more complex techniques in the Methods section.*
☒	☐	A description of all covariates tested
☒	☐	A description of any assumptions or corrections, such as tests of normality and adjustment for multiple comparisons
☐	☒	A full description of the statistical parameters including central tendency (e.g. means) or other basic estimates (e.g. regression coefficient) AND variation (e.g. standard deviation) or associated estimates of uncertainty (e.g. confidence intervals)
☐	☒	For null hypothesis testing, the test statistic (e.g. F, t, r) with confidence intervals, effect sizes, degrees of freedom and P value noted<br>*Give P values as exact values whenever suitable.*
☒	☐	For Bayesian analysis, information on the choice of priors and Markov chain Monte Carlo settings
☒	☐	For hierarchical and complex designs, identification of the appropriate level for tests and full reporting of outcomes
☒	☐	Estimates of effect sizes (e.g. Cohen's d, Pearson's r), indicating how they were calculated

Our web collection on statistics for biologists contains articles on many of the points above.

Software and code

Policy information about availability of computer code

Data collection	All quantitative PCR (qPCR) data was collected by CFX Manager (version 2.1). All flow cytometry data was collected by CytExpert(version 2.3.1.22). All Immunofluorescence data were collected by Leica Application Suite X (LAS X). Western blotting results were collected by Image Lab software (version 4.1.0.2177). Caspase8 activity assay and Cell Counting Kit-8 assay data were collected by Gen5 Software(version 3.04). All luciferase activity assay data were collected by GloMax®-Multi Detection System Software (version 1.10).
Data analysis	All statistical analyses were performed on Graphpad Prism (version 7.04, La Jolla, CA). All flow cytometry data were analyzed on Cytoexpert (version 2.3.1.22, Beckman Coulter). Phylogenetic trees were constructed using the neighbor-joining (N-J) method within the MEGA (version 4.1) package. For Western blotting and immunofluorescence, images were analyzed with ImageJ (version 1.52r).

For manuscripts utilizing custom algorithms or software that are central to the research but not yet described in published literature, software must be made available to editors and reviewers. We strongly encourage code deposition in a community repository (e.g. GitHub). See the Nature Portfolio guidelines for submitting code & software for further information.

Data

Policy information about availability of data

All manuscripts must include a data availability statement. This statement should provide the following information, where applicable:

- Accession codes, unique identifiers, or web links for publicly available datasets
- A description of any restrictions on data availability
- For clinical datasets or third party data, please ensure that the statement adheres to our policy

The longer CD44a transcript variant was named as CD44a_tv1 (GenBank accession number: MW674927) and the shorter CD44a transcript variant was named as

CD44a_tv2 (GenBank accession number: MW674928).The raw sequences of Illumina deep sequencing were deposited at NCBI Gene Expression Omnibus (GEO) database under the accession number GSE180770.

Field-specific reporting

Please select the one below that is the best fit for your research. If you are not sure, read the appropriate sections before making your selection.

☒ Life sciences ☐ Behavioural & social sciences ☐ Ecological, evolutionary & environmental sciences

For a reference copy of the document with all sections, see nature.com/documents/nr-reporting-summary-flat.pdf

Life sciences study design

All studies must disclose on these points even when the disclosure is negative.

Sample size	Samples were taken from at least three repeat experiments. Sample sizes were determined according to a pilot study as well as on the basis of previous experimental experience.
Data exclusions	No data were excluded.
Replication	Each experiment was performed with at least 3 biological replicates. All the reported results are from experiments in which every repeat gave similar results.
Randomization	All samples were randomly allocated into experimental groups.
Blinding	No formal blinding was used.

Reporting for specific materials, systems and methods

We require information from authors about some types of materials, experimental systems and methods used in many studies. Here, indicate whether each material, system or method listed is relevant to your study. If you are not sure if a list item applies to your research, read the appropriate section before selecting a response.

Materials & experimental systems

n/a	Involved in the study	
☐	☒	Antibodies
☐	☒	Eukaryotic cell lines
☒	☐	Palaeontology and archaeology
☐	☒	Animals and other organisms
☒	☐	Human research participants
☒	☐	Clinical data
☒	☐	Dual use research of concern

Methods

n/a	Involved in the study	
☒	☐	ChIP-seq
☐	☒	Flow cytometry
☒	☐	MRI-based neuroimaging

Antibodies

Antibodies used	Antibody: Rabbit Anti-p62 / SQSTM1 Antibody (Cat# P0067, Sigma-Aldrich), Anti-LC3B antibody produced in rabbit (Cat# L7543, Sigma-Aldrich), Mouse GAPDH monoclonal antibody (Cat# 60004-1-Ig, proteitech), Monoclonal ANTI-FLAG® M2 antibody (Cat# F3165, Sigma-Aldrich), Anti-TurboGFP polyclonal antibody (Cat# AB513, Evrogen), Anti-beta Tubulin antibody (Cat# ab6046, Abcam), Anti-HDAC1 antibody (Cat# ab41407, Abcam), p53 (ET1601-13, HuaAn Biotechnolog), phospho-Histone H3 (ab11477, abcam), phospho-Akt (#4060S, Cell Signaling), Akt (#9272, Cell Signaling), phospho-GSK-3β (#9323, Cell Signaling), GSK-3β (#9315, Cell Signaling), zebrafish CD44a (25340-1hz, Abmart),Goat anti-Rabbit IgG (H+L) Secondary Antibody (Cat# 31460, Thermo Fisher Scientific), Goat anti-Mouse IgG (H+L) Secondary Antibody (Cat# 31430, Thermo Fisher Scientific), ReadyProbes™ Alexa Fluor® 594 Goat Anti-Mouse IgG Antibody (Cat#R37121, Invitrogen), Alexa Fluor™ 488 Goat anti-Rabbit IgG (#A11008, Invitrogen) and Alexa Fluor™ 488 (#A110011, Invitrogen)
Validation	All antibodies were validated by their source company. The antibodies used in this study have been previously described and used in previously published research.

Eukaryotic cell lines

Policy information about cell lines

Cell line source(s)	EPC cells (ATCC CRL-2872) was purchased from ATCC. WT or CD44a deficiency caudal fin-derived cell lines were generated by our laboratory using the tissue block adherent method.

Authentication	EPC cells (ATCC CRL-2872) was authenticated by the supplier.
Mycoplasma contamination	All cell lines were tested for mycoplasma contamination. No mycoplasma contamination was found.
Commonly misidentified lines (See ICLAC register)	This study did not use commonly misidentified cell lines.

Animals and other organisms

Policy information about studies involving animals; ARRIVE guidelines recommended for reporting animal research

Laboratory animals	Zebrafish strain used in this study was AB strain. Wild-type and NOD1 mutant zebrafish were obtained from the China Zebrafish Resource Center (CZRC).Two CD44a-knockout mutants were generated in our laboratory using CRISPR/Cas9 technique. 2 days post fertilization zebrafish larvae were used for Phospho-histone 3 (pH3) immunostaining.4 days post fertilization zebrafish larvae were used for infectious experiments.
Wild animals	This study did not involve wild animals
Field-collected samples	This study did not involve field-collected asmples
Ethics oversight	All animal experiments were conducted in accordance with the Guiding Principles for the Care and Use of Laboratory Animals and were approved by the Institute of Hydrobiology, Chinese Academy of Sciences (Approval ID: IHB 2013724).

Note that full information on the approval of the study protocol must also be provided in the manuscript.

Flow Cytometry

Plots

Confirm that:

☒ The axis labels state the marker and fluorochrome used (e.g. CD4-FITC).

☒ The axis scales are clearly visible. Include numbers along axes only for bottom left plot of group (a 'group' is an analysis of identical markers).

☒ All plots are contour plots with outliers or pseudocolor plots.

☒ A numerical value for number of cells or percentage (with statistics) is provided.

Methodology

Sample preparation	The cells were harvested, and then resuspend in 1 × Binding Buffer at a concentration of 1 ×10(6) cells/mL. The cells were stained with annexin V FITC (BD Biosciences) according to the instructions of the manufacturer.
Instrument	CytoFLEX S(Beckman,USA)
Software	CytExpert Software (version 2.3.1.22, Beckman Coulter)
Cell population abundance	No sorting was performed.
Gating strategy	Cells were gated on FSC-A/SSC-A.

☒ Tick this box to confirm that a figure exemplifying the gating strategy is provided in the Supplementary Information.
